# Vibration-mediated long-wavelength photolysis of electronegative bonds beyond S_0_–S_1_ and S_0_–T_1_ transitions

**DOI:** 10.1038/s42004-024-01208-0

**Published:** 2024-06-04

**Authors:** Antônio Junio Araujo Dias, Atsuya Muranaka, Masanobu Uchiyama, Ken Tanaka, Yuki Nagashima

**Affiliations:** 1 Department of Chemical Science and Engineering, Tokyo Institute of Technology, O-okayama, Meguro-ku, Tokyo 152-8550 Japan; 2 Molecular Structure Characterization Unit, RIKEN Center for Sustainable Resource Science, 2-1 Hirosawa, Wako, Saitama 351-0198 Japan; 3 Graduate School of Pharmaceutical Sciences, The University of Tokyo, 7-3-1 Hongo, Bunkyo-ku, Tokyo 113-0033 Japan

**Keywords:** Synthetic chemistry methodology, Photochemistry, Synthetic chemistry methodology

## Abstract

Photolysis is an attractive method in organic synthesis to produce free radicals through direct bond cleavage. However, in this method, specific irradiation wavelengths of light have been considered indispensable for excitation through S_0_–S_n_ or S_0_–T_n_ transitions. Here we report the photoinduced homolysis of electronegative interelement bonds using light at wavelengths much longer than theoretically and spectroscopically predicted for the S_0_–S_n_ or S_0_–T_n_ transitions. This long-wavelength photolysis proceeds in N–Cl, N–F, and O–Cl bonds at room temperature under blue, green, and red LED irradiation, initiating diverse radical reactions. Through experimental, spectroscopic, and computational studies, we propose that this “hidden” absorption is accessible via electronic excitations from naturally occurring vibrationally excited ground states to unbonded excited states and is due to the electron-pair repulsion between electronegative atoms.

## Abstract (web summary)

Photolysis can induce the homolytic cleavage of chemical bonds through the excited state under specific irradiation wavelengths, producing free radical species. Here, the authors report the photoinduced homolysis of electronegative interelement bonds (N–Cl, N–F, and O–Cl bonds) using light with wavelengths longer than that predicted for the S_0_–S_n_ or S_0_–T_n_ transitions, initiating diverse radical reactions.

## Introduction

Photolysis, the homolytic cleavage of chemical bonds through the excited state, offers an attractive method to produce free radical species using traceless photons^1–7^. The first report of an organic reaction initiated by photoinduced homolysis dates back to the accidental discovery of N–Cl bond cleavage of nitrosyl chloride under sunlight irradiation in 1919^8^. Since then, by using ultraviolet light, light-emitting diodes (LEDs) or sunlight, this strategy has evolved to be elegantly applied to many organic reactions, including radical addition reactions, radical-mediated functionalizations, and radical-initiated polymerizations^9–15^.

**Fig. 1:**
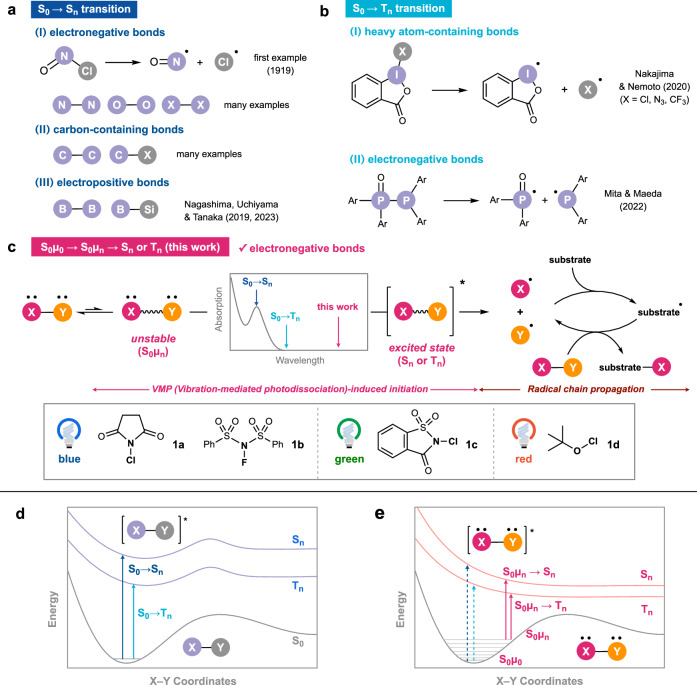
Photolysis via conventional direct transitions and vibration-mediated transition in organic synthesis. **a** Photolysis of electronegative, carbon-containing, or electropositive bonds via S_0_–S_n_ transition. **b** Photolysis of heavy atom-containing or electronegative bonds via S_0_–T_n_ transition. **c** Long-wavelength photolysis of electronegative bonds via S_0_μ_0_–S_0_μ_n_–S_n_ or T_n_ transition (this work). **d** Potential energy surface and S_0_–S_n_ or T_n_ transition. **e** Potential energy surface of electronegative bonds and S_0_μ_0_–S_0_μ_n_–S_n_ or T_n_ transition (this work).

In photolysis, there are two routes to the excited state from the ground state (S_0_), and the use of an appropriate wavelength of light is necessary for the electronic excitation of the target molecules. The first route is a transition between the singlet excited states (S_0_–S_n_), which may be followed by internal conversion to another singlet excited state or intersystem crossing (ISC) to the triplet excited state (T_n_). Many examples of photolysis of electronegative bonds, such as O–O, O–X, N–X, and X–X (X = halogen) bonds (Fig. 1a-(I))^16–22^, as well as carbon-containing C–C or C–X bonds, via the excitation to the S_n_ state have been reported (Fig. 1a-(II))^23–26^. Our group has also developed borylation and silylation reactions based on the photolysis of electropositive interelement B–B and B–Si bonds via S_0_–S_n_ transition (Fig. 1a-(III))^27,28^.

The second route to the excited state is a direct excitation to the triplet state (T_n_), although S_0_–T_n_ transition is generally restricted due to the intrinsic need for a spin inversion^29,30^. In 2020, Nakajima and Nemoto established that photoinduced reactions of heavy-atom-containing molecules, such as hypervalent iodine or bismuth compounds, proceed via direct S_0_–T_n_ transition, with the aid of the relaxation of spin restrictions based on the internal heavy-atom effect (Fig. 1b-(I))^31,32^. Subsequently, in 2022, Mita and Maeda also featured the homolysis of P–P bonds via light absorption through direct S_0_–T_n_ transition (Fig. 1b-(II))^33^. In these cases, the potential energy of the triplet state (T_n_) is usually lower than that of the singlet state (S_n_), and thus longer wavelengths of light can be employed in these reactions (Fig. 1d). Thus, when designing photolysis-driven organic reactions, the appropriate irradiation wavelength has conventionally been determined by investigating the absorption spectrum of the target molecule or by predicting S_0_–S_n_ as well as S_0_–T_n_ transition energies with theoretical calculations.

Here we report the photoinduced homolysis of electronegative interelement bonds using light at wavelengths much longer than theoretically and spectroscopically predicted for the S_0_–S_n_ or S_0_–T_n_ transitions (Fig. 1c). Specifically, this report includes the photohomolysis of the following interelement bonds: (1) the N–Cl bond in *N*-chlorosuccinimide (NCS, **1a**) and the N–F bond in *N*-fluorobenzenesulfonimide (NFSI, **1b**), (2) the N–Cl bond in *N*-chlorosaccharin (NCSA, **1c**), and (3) the O–Cl bond in *t*-butyl hypochlorite (**1d**), under blue, green, and red LEDs irradiation, respectively. Through experimental, spectroscopic, and computational studies, we propose that this “hidden” absorption occurs via an electronic transition from a thermally populated vibrational excited state (S_0_μ_0_–S_0_μ_n_–S_n_ or T_n_, Fig. 1e), referred to as vibration-mediated photodissociation (VMP) in physical chemistry^34–36^. VMPs of small molecules such as water, acetylene, ammonia, nitric acid, hydrogen peroxide, pyrrole, and methanethiol have been detected by laser-based double-resonance techniques^34–39^. VMP has also been used to investigate intramolecular vibrational redistribution dynamics^40–42^, but not for organic synthesis. The proposed mechanism is shown in Fig. 1c. Molecules bearing N–Cl, N–F, and O–Cl bonds are stabilized in the excited state as their bond lengths increase, reducing the energy necessary for S_0_μ_n_–S_n_ or T_n_ transitions and allowing their photolysis and “hidden” absorption in the long-wavelength region. Hence, the electronic excitation of a small fraction of molecules, specifically those in the excited vibrational state (S_0_μ_n_) at thermal equilibrium, would allow direct access to the excited state (S_0_μ_n_–S_n_ or T_n_). Although such long-wavelength photolysis generates only trace amounts of the radical species, combining it with a chain process enables reactions on a bulk scale. VMP first meets organic synthesis in this paper to enable the C(sp^2^)–H chlorination/fluorination of aldehydes and allylic C(sp^3^)–H chlorination/aminochlorination of alkenes using long wavelengths of light beyond S_0_–S_1_ and S_0_–T_1_ transitions.

## Results and discussion

### Computational analysis

We have previously reported the photolysis of electropositive interelement B–B and B–Si bonds by the formation of light-absorbing complexes under irradiation with light of longer wavelengths than the absorption of B–B and B–Si reagents^27,28^. During the application of this complexation strategy to the electronegative N–Cl bond of **1a**, we unexpectedly found the blue-light-induced photolysis of **1a** without any complexations (Supplementary Fig. 1a). However, our measurement of the absorption spectrum of **1a** showed that the major absorption peaks were observed below 250 nm, and the absorption tail cannot reach even UV-A (<320 nm) under standard measurement conditions (Supplementary Fig. 1b).

Considering that the spin-restricted S_0_–T_n_ transition might enable the absorption of such longer wavelength^31–33^, we theoretically predicted the S_0_–S_n_ and S_0_–T_n_ transition wavelengths and bond dissociation energies (BDEs) for N–Cl bonds by density functional theory (DFT) and time-dependent DFT (TD-DFT) calculations^43–46^ at the (U)M06/6-31 + G* or (U)M06/6-311 + G* levels of theory. Typical compounds involving **1a** are shown in Table 1 (for the detailed list of calculated *N*-chloroamines, see: Supplementary Table 2). For such small molecules with only N–Cl bonds, carbonyl groups, and phenyl groups, the S_0_–S_1_ transition is limited to wavelengths below 320 nm, as expected, and the spin-restricted S_0_–T_1_ transition was assigned to UV-A (320–400 nm) for **1e,** **1** **f,** **1** **h** and **1i** (*N*–chlorophthalimide, NCP), or visible light (>400 nm) for **1c**. These calculations well explained previous reports where photolysis occurs in **1a** and **1c**–**i** at the above-predicted irradiation wavelengths^19,47–49^, but the present visible-light-induced photolysis of **1a** remained inexplicable. In addition, according to our prediction, two reports of catalyst-free blue-light-induced N–Cl excitation reactions for **1e**^50^ and **1** **h**^51^, where the absorption tails can reach visible light, may not be explained by S_0_–S_n_ and S_0_–T_n_ transition. On the basis of these contradictions between experimental and theoretical results, we hypothesized that the electron-pair repulsions in electronegative bonds could facilitate photolysis at much longer irradiation wavelengths than both the theoretically predicted S_0_–S_n_ or S_0_–T_n_ transitions and the spectroscopically observed absorption tails to enable diverse radical reactions using “hidden” absorption.

**Table 1:** DFT and TD-DFT calculated transitions from the ground state (S_0_) to the singlet (S_1_) and triplet (T_1_) excited states and BDEs of compounds with N–Cl bond


Compound	S_0_ → S_1_ [nm]	S_0_ → T_1_ [nm]	BDE [kcal mol^-1^]
**1e**	294	333	46.7
**1f**	294	363	50.2
**1c** (NCSA)	277	408	58.2
**1g**	270	316	60.1
**1h**	253	333	62.5
**1i** (NCP)	313 (311)	434 (380)	69.5 (75.3)
**1a** (NCS)	242 (240)	275 (251)	73.2 (73.2)

DFT and TD-DFT calculations were performed at the (U)M06/6-31 + G* levels of theory. The values given in parentheses are the wavelengths and BDEs calculated at the (U)M06/6-311 + G** levels of theory. *BDE* bond dissociation energy.

### Experimental analysis

To test the validity of our hypothesis, we first conducted C(sp^2^)–H chlorination of benzaldehyde (**2a**), which proceeded using **1** **h** under the irradiation wavelength corresponding to absorption tails reported by Luca^51^, with the most stable N–Cl reagent **1a** under irradiation wavelengths beyond S_0_–S_1_ and S_0_–T_1_ transitions (Table 2). The efficacy of the halogenation step was evaluated by derivatizing the unstable acyl chloride produced to the corresponding stable amide using dimethylamine. Without light irradiation, the chlorination of **2a** did not proceed with **1a** (entry 1). In contrast, irradiation with blue LEDs (390–470 nm, *λ*_max_ = 450 nm) in a wavelength range much longer than the theoretically estimated for the S_0_–S_1_ (242 nm, Table 1) or the S_0_–T_1_ absorption peaks (275 nm, Table 1) of **1a**, could promote the C(sp^2^)–H chlorination of **2a** to give the corresponding acyl chloride, which was subjected to amidation to form *N*,*N*–dimethylbenzamide **3a** in 83% yield (entry 2). However, green LEDs (490–570 nm, *λ*_max_ = 525 nm) could not activate the present chlorination (entry 3). Notably, heating could not promote this reaction either, indicating that this reaction proceeded via photoinduced homolysis of the N–Cl bond (entry 4). This reaction also proceeded under air (entry 5), showing that the bond dissociation might occur faster than the oxygen quenching of the excited state. Furthermore, the yields correlate with the irradiance of the used blue LEDs (entries 6–8), and the present “hidden” absorption is accessible even with lower irradiances. Hence, two-photon excitation is unlikely.

**Table 2:** Optimization of reaction conditions of the C(sp^2^)–H chlorination of aldehydes


Entry	N–Cl reagent	Light source	Temperature	Yield of 3a (%)
1	**1a**	none	r.t.	0
**2**	**1a**	**blue LEDs**	**r.t**.	**83 (76)**
3	**1a**	green LEDs	r.t.	0
4	**1a**	none	60 °C	0
5^a^	**1a**	blue LEDs	r.t.	80
6^b^	**1a**	blue LEDs ( > 2000 W m^-2^)	r.t.	62
7^b^	**1a**	blue LEDs (776 W m^-2^)	r.t.	47
8^b^	**1a**	blue LEDs (221 W m^-2^)	r.t.	20
9	**1i**	blue LEDs	r.t.	54
10	**1i**	green LEDs	r.t.	10
**11**	**1c**	**green LEDs**	**r.t**.	**86 (87)**

Reactions were conducted using **1** (0.10 mmol), **2a** (0.11 mmol), and CCl_4_ (0.20 mL) under argon at room temperature (r.t.) for 18 h under irradiation with light source, and then stirred for 2 h after the addition of Et_3_N (0.30 mL) and a 2.0 M THF solution of HNMe_2_ (0.50 mL). Yields were determined by ^1^H NMR of the crude mixture using mesitylene as the internal standard. Yields in parenthesis are of isolated products. Blue LEDs (wavelength range: 390–470 nm, *λ*_max_ = 450 nm, >2000 W m^-2^) or green LEDs (wavelength range: 490–570 nm, *λ*_max_ = 525 nm, >2000 W m^-2^) were used. ^a^ Under air. ^b^ Run for 2 h instead of 18 h.

Next, we examined the present reaction using **1i** and **1c** (entries 9–11). Reagent **1i** gave a similar result to **1a** under blue LEDs irradiation, but it was also activatable with green LEDs, albeit with low product yield. In the case of **1c**, the product was obtained in high yield under green LEDs (entry 11) irradiation with wavelengths much longer than the theoretically estimated for the S_0_–S_1_ (277 nm, Table 1) or S_0_–T_1_ (408 nm, Table 1) absorption peaks. It is worth mentioning that previous reports have used light-driven photocatalytic radical initiators^52–54^, and thus this is the first example of a catalyst-free visible-light-induced C(sp^2^)–H chlorination of aldehydes by NCS (**1a**).

### Substrate scope

**Fig. 2:**
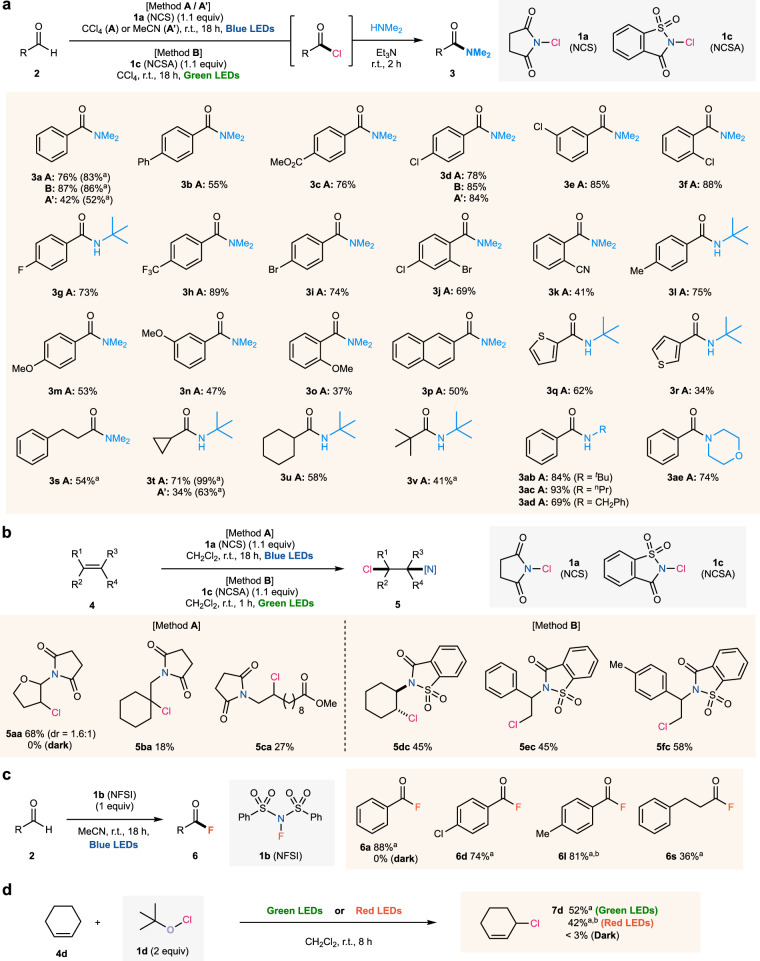
Photolysis-driven halogenation reactions. **a** C(sp^2^)–H chlorination of aldehydes. **2** (0.10 mmol), **1a** (0.11 mmol), and CCl_4_ (0.20 mL) or MeCN (0.10 mL) were used [Method **A** or **A’**]. **2** (0.10 mmol), **1c** (0.11 mmol), and CCl_4_ (0.20 mL) were used [Method **B**]. **b** Aminochlorination of alkenes. **4** (0.50 mmol), **1a** (0.55 mmol), and CH_2_Cl_2_ (1.0 mL) were used [Method **A**], and **4** (0.10 mmol), **1c** (0.11 mmol), and CH_2_Cl_2_ (0.2 mL) were used [Method **B**]. **c**, C(sp^2^)–H fluorination of aldehydes. **2** (0.20 mmol), **1b** (0.20 mmol), and MeCN (0.4 mL) were used. **d**, Allylic C(sp^3^)–H chlorination. **4d** (0.5 mmol), **1d** (1.0 mmol), and CH_2_Cl_2_ (1.0 mL) were used. Cited yields are of isolated products. Blue LEDs (*λ*_max_ = 450 nm, >2000 W m^-2^), green LEDs (*λ*_max_ = 525 nm, >2000 W m^-2^), or red LEDs (*λ*_max_ = 640 nm, <771 W m^-2^) were used. ^a^ Yields determined by ^1^H or ^19^F NMR of the crude mixture using mesitylene as the internal standard. ^b^ Product was isolated after amidation or amination.

The substrate scope of the photoinduced C(sp^2^)–H chlorination of aldehydes followed by amidation is shown in Fig. 2a. For blue LEDs irradiation using **1a** (Method **A**, Fig. 2a), aromatic aldehydes bearing various electron-withdrawing groups (*p*-Ph, *p*-CO_2_Me, *p*-CF_3_, *o*-/*m*-/*p*-Cl, *p*-F, *p*-Br, and *m*-cyano) as well as electron-donating groups (*p*-Me and *o*-/*m*-/*p*-OMe) could be used to give the corresponding amide products in good to excellent yields (**3b–3o**). π-extended arenes- (**2p**) and heteroarenes-substituted (**2q** and **2r**) aldehydes were also compatible. Importantly, not only aromatic aldehydes but aliphatic aldehydes can participate in the present chlorination to afford the amide products **3s–3** **v** in moderate yields, indicating that the formation of an NCS (**1a**)-substrate complex that allows longer wavelength absorption is unlikely^11,12^. Additionally, sequential one-pot amidation with various amines was possible, giving the amide products **3ab–3ae** without isolation of the acyl chlorides. Similarly, under green LEDs irradiation (525 nm), NCSA (**1c**) could also afford the amide products **3a** and **3d** (Method **B**, Fig. 2a). According to solvent screening experiments (Supplementary Table 1), the present C(sp^2^)–H chlorination proceeds in not only CCl_4_ but also other solvents, such as MeCN and CH_2_Cl_2_, or even under solventless conditions, to afford the desired products **3a,** **3d**, and **3t** (Method **A’** for MeCN, Fig. 2a).

This visible-light-induced activation mode also enabled the aminochlorination of alkenes **4** (Fig. 2b). In previous reports, a combination of short wavelength UV light irradiation and stable N–Cl reagents such as **1a**^47,48^ and **1i**^19^ or fluorescent lamp irradiation and an unstable N–Cl reagent such as **1c**^49^ enabled this transformation. In contrast, aminochlorination with **1a** under blue LEDs irradiation afforded the corresponding alkanes **5aa–5ca** bearing both amino and chloride groups in moderate yields, analogous to those previously reported (Method **A**, Fig. 2b). As in the C(sp^2^)–H chlorination of aldehydes, these aminochlorination reactions did not proceed without photo-irradiation. Moreover, green light irradiation enabled the aminochlorination of cyclohexene **4d** and styrene derivatives **4e** and **4** **f** to afford **5dc,** **5ec**, and **5** **fc**, respectively, in good yields, where interestingly, the aminochlorination of **4d** with **1c** afforded *trans*-**5dc** product stereoselectively (Method **B**, Fig. 2b). This selectivity can be explained as follows. In the reaction of succinimide/chlorine radicals with alkenes, the addition of the nitrogen-centered radical is favorable for unactivated alkenes (**4b** and **4c**)^47,48^. Vinyl ether derivate **4a** may simultaneously react ionically via halogenium intermediates (in addition to the free radical mechanism), giving both products as reported for the reaction with NCP^55^. In contrast, changing the nitrogen-centered radical to saccharin favors the addition of the chlorine radical, yielding **5ec** and **5** **fc**, as previously reported^49^.

Next, we examined whether unexpectedly long wavelengths of light would similarly activate electronegative interelement bonds involving the F atom in the second row instead of the Cl atom in the third row. We were pleased to find that blue LEDs irradiation enables the fluorination of aldehydes using **1b** through the N–F bond photolysis (Fig. 2c). Thus, we have established that the heavy atom effect is not the key to the present photolysis. Aldehydes **3a,** **3d**, and **3** **s** were successfully converted and isolated in good to high yields as the corresponding fluorinated derivates, more stable than their chlorinated counterparts. The fluorination product from the electron-rich aldehyde **1** **l** was unstable and thus was isolated after amidation, yielding the target product in high yield. As in the other cases, according to the theoretically predicted S_0_–S_1_ (246 nm) and S_0_–T_1_ (307 nm) absorption peaks for **1b** (Supplementary Table 2), absorption of blue LEDs is not expected. It is worth mentioning that the present reaction constitutes the first example of a catalyst-free visible-light-induced C(sp^2^)–H fluorination of aldehydes by reagents with N–F bonds^56^.

In addition, the O–Cl bond of **1d**, for which S_0_–S_1_ (333 nm) and S_0_–T_1_ (399 nm) absorption peaks are theoretically predicted (Supplementary Table 2), underwent photoinduced homolysis with cyclohexene (**4d**) under green LEDs irradiation (525 nm) to give the allylic C(sp^3^)–H chlorination^57^ product **7d**, as shown in Fig. 2d. Furthermore, red LEDs irradiation (>600 nm) also enabled this chlorination to give **7d** in good yield.

### Mechanistic studies

**Fig. 3:**
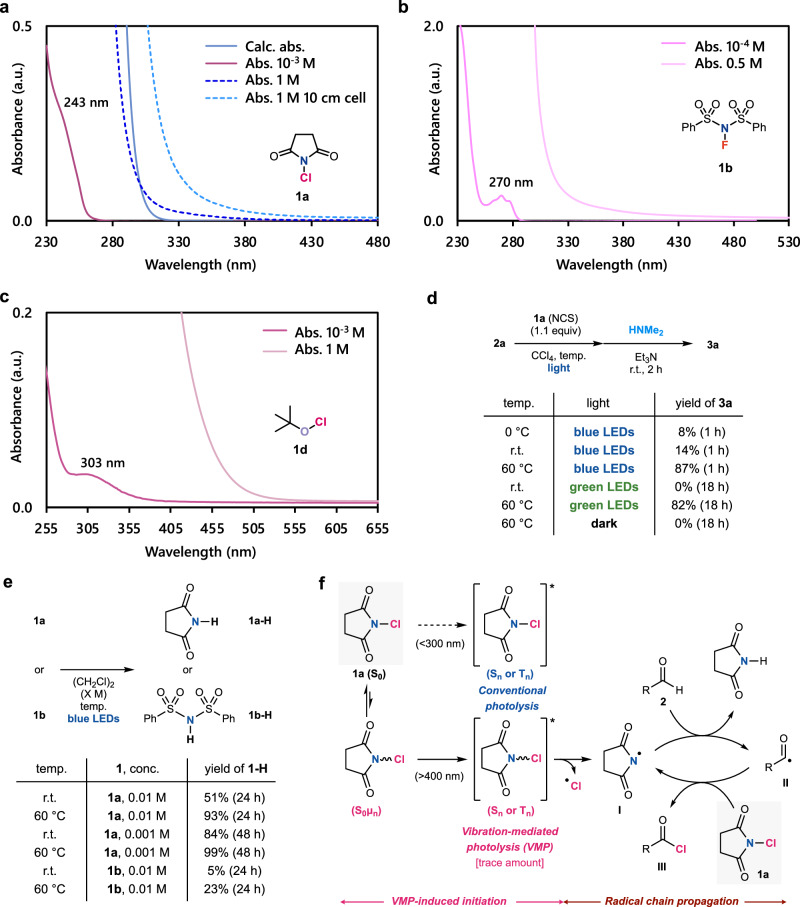
Experimental mechanistic studies and proposed mechanism. **a–c** Absorption spectra of **1a,** **1b**, and **1d** in MeCN, respectively. DFT calculated absorption was obtained at the M06/6-31 + G* level of theory. **d–e** Temperature dependence of the photoinduced C(sp^2^)–H chlorination of **2a** with **1a**, the photolysis of **1a**, and the photolysis of **1b**. Yields were determined by ^1^H NMR using mesitylene as the internal standard. Calc, calculated. Abs, absorption. **f** Proposed mechanism.

We conducted spectroscopic studies to elucidate the mechanism of this activation (Fig. 3). When we measured the absorption spectrum of **1a** in MeCN (1 × 10^–3^ M), the major absorption peaks, corresponding to the spin-allowed S_0_–S_n_ transitions, were observed below 250 nm, as predicted in Table 1 (Fig. 3a). Although we observed a considerable broadening of the original absorption peak at the high concentration of 1 M, the absorption of visible light is still almost undetectable. On the other hand, when we used a 10 cm cell (10 times longer than a conventional cell), the peak broadening can finally reach the visible light range, but the molar absorption coefficient (ε) is only 2 × 10^–3^ at 400 nm. In addition, spectroscopic measurements of **1b** and **1d** in MeCN gave similar results. We observed very weak absorption of longer wavelengths (visible light) only in highly concentrated solutions, but no discernible peaks could be observed (Figs. 3b and 3c). Therefore, these results support the possibility that weak transitions to S_n_ or T_n_ states can occur at much larger wavelengths than theoretically predicted and experimentally observed at standard measuring conditions.

Next, we conducted experimental mechanistic studies with external stimuli. In the present photoinduced C(sp^2^)–H chlorination of aldehydes with **1a**, the temperature has a critical impact on the product yields (Fig. 3d). The high temperature of 60 °C drastically accelerated the blue-light-induced reaction to afford **3a** in a high 87% yield, but the low temperature of 0 °C reduced the yield to 8%. More importantly, green LEDs irradiation promoted the reaction at 60 °C, in contrast to no reaction at room temperature (Fig. 3d). Additionally, the simple photolysis of **1a** and **1b** in highly diluted solutions (0.01 or 0.001 M), where radical chain propagation processes are minimized, was also accelerated at 60 °C, while heating alone could not promote these reactions (Fig. 3e). These results indicate that heating cannot be used to overcome the energy barrier and promote homolysis directly from the ground state. In contrast, based on VMP theories^34–36^, heating could enhance photoexcitation.

Therefore, we propose the reaction proceeds as shown in Fig. 3f. The unexpectedly red-shifted absorption could be derived from the excitation from a naturally occurring vibrationally excited state (S_0_μ_n_) that is energetically closer to the excited state. This state could theoretically absorb light since electronic transitions (S_0_μ_n_–S_n_ or T_n_) are a faster process (10^-14^–10^-15^ s) than internal conversion (S_0_μ_n_– S_0_μ_0_) (10^-12 ^s)^58^. In the excited state, the homolysis of the N–Cl bond of **1a** can afford the nitrogen-centered radical **I**, which reacts with the aldehyde **2** to provide the C(sp^2^)-centered radical **II**. **II** affords the desired chlorinated product **III** by abstracting the chlorine from **1a**, which regenerates the nitrogen-centered radical **I**. Therefore, a propagation process enables trace amounts of photo-generated radicals to drive the reactions in bulk.

**Fig. 4:**
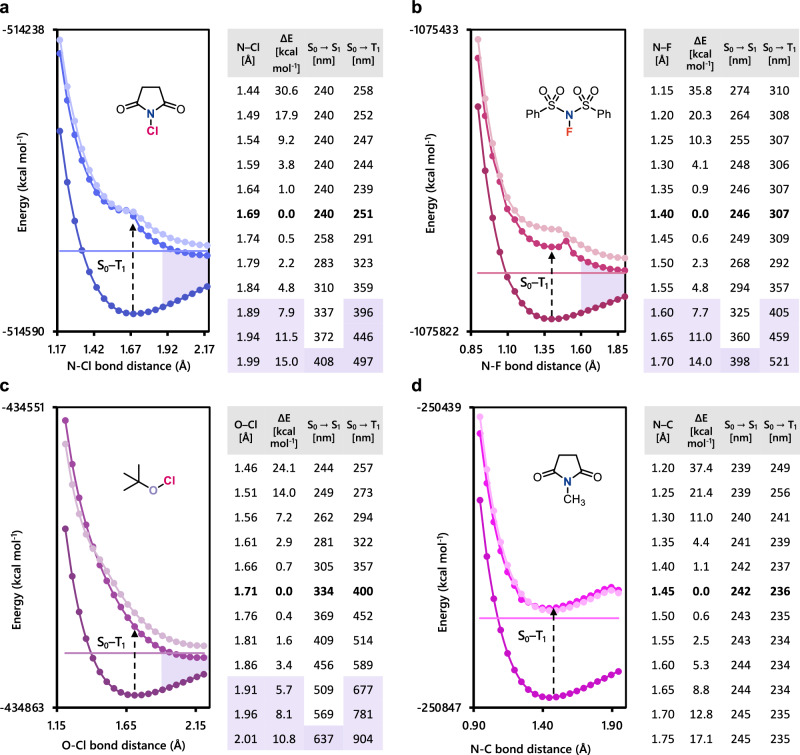
Calculated transitions from unstable conformations in the N–Cl, N–F, O–Cl, and N–C axis. **a**–**d** Potential energy surface and transition values for various N–Cl, N–F, O–Cl, and N–C bond lengths of **1a,** **1b,** **1d**, and **1j**, respectively, by DFT and TD-DFT calculations at the (U)M06/6-311 + G** levels of theory. The vertical line in each graph illustrates the calculated BDE. The changes in the electronic energies are shown as ⊿*E* in kcal mol^-1^.

To examine this hypothesis, we predicted both S_0_–S_1_ and S_0_–T_1_ transition wavelengths from the ground state surfaces by scanning the potential energy along the electronegative-bond lengths using DFT and TD-DFT methods (Fig. 4). When the distances of the electronegative N–Cl, N–F, and O–Cl bonds increase, not only the destabilization of the ground state but also the stabilization of the excited states reduces the energy required for S_0_–S_n_ or S_0_–T_n_ transitions to allow an increase of absorption wavelengths (Fig. 4a–c and Supplementary Table 14). For instance, in the S_0_–T_1_ transitions of **1a** (Fig. 4a), the destabilization of the ground state of 7.9 kcal mol^-1^ (N–Cl = 1.89 Å) leads to an increase of over 100 nm in the absorption wavelength (from 251 nm to 396 nm), reaching the visible range. Vibrational analysis showed that this energy state is accessible for the 4th vibrational state of the N–Cl vibrational mode, for example, which has a Boltzmann factor of 4.0× 10^–6^ at 27 °C and 1.9× 10^–5^ at 60 °C (vibration number = μ10-4 in Supplementary Tables 16 and 17). Similarly, in the S_0_–T_1_ transitions of **1b** (Fig. 4b), the destabilization of the ground state of 7.7 kcal mol^-1^ (N–F = 1.60 Å) leads to a larger absorption wavelength from 307 nm to 405 nm. Furthermore, in the S_0_–T_1_ transitions of **1d** (Fig. 4c), the absorption wavelength of the 1.6 kcal mol^-1^ unstable structure (O–Cl = 1.81 Å) reaches the green light region, and that of the even more unstable one (5.7 kcal mol^-1^, O–Cl = 1.91 Å) extends into the red light range. In contrast, in **1j** where the N–C bond is not an electronegative interelement bond, absorption wavelengths do not increase due to the destabilization of both the ground and excited state (Fig. 4d).

Thus, the observed red-shifts common to electronegative interelement bonds are thought to be dependent on the repulsive character of the excited state. These unstable molecular configurations would be accessible via naturally occurring vibrationally excited states, which are expected to follow a Boltzmann distribution in equilibrium. Not only the S_0_–T_1_ but the S_0_–S_1_ transition, which does not require a spin inversion, can also contribute to the red-shift of the absorption. For example, in the S_0_–S_1_ transitions of **1a** (Fig. 4a), a destabilization of the ground state of 15.0 kcal mol^–1^ (N–Cl = 1.99 Å) can reach the visible region from 240 nm to 408 nm. Thus, we propose that these visible-light-induced infinitesimal transitions facilitated by the vibration in electronegative interelement bonds could generate trace amounts of radical initiators that allow a variety of radical reactions to proceed. Importantly, these bonds were cleaved with irradiation energies lower than the respective BDEs. For example, **1i** (calculated BDE of N–Cl bond = 75.3 kcal mol^–1^, Table 1) was successfully cleaved with green LEDs, whose irradiation energy is up to only 58.4 kcal mol^–1^ ( > 490 nm).

## Conclusion

We have established that electronegative interelement bonds involving N–Cl, N–F, and O–Cl bonds can be cleaved by long-wavelength light irradiation at room temperature, contradicting conventional prediction methods used to determine appropriated irradiation wavelengths, such as absorption spectroscopy and S_0_–S_n_ or S_0_–T_n_ transition energy calculations. Experimental, spectroscopic, and computational studies support that this “hidden” absorption is accessible via electronic excitations from vibrationally excited ground states to unbonded excited states without external stimulus. Consistent with vibration-mediated photodissociation theories, this absorption would lead to bond homolysis, giving the corresponding free radicals. Hence, the observed red-shift in absorption common to electronegative interelement bonds would be dependent on the repulsive character of the excited state. We propose that this vibration-mediated photolysis, although infinitesimal when compared with the typical excitation from a vibrationally stable ground state, could initiate diverse visible-light-induced radical reactions, such as the C(sp^2^)–H chlorination and fluorination of aldehydes and the aminochlorination or allylic C(sp^3^)–H chlorination of alkenes under mild conditions. Visible light photolysis of specific bonds without external stimuli is valuable for materials recycling, such as polymer degradation, especially when using light in the low-energy deep red or near-infrared region (*λ* > 600 nm), which is essential for in vivo reactions. Although, we are still unable to exclude alternative mechanisms, such as two photon absorption, complexation, or an impurity-initiated chain reaction, and further studies, such as UV-vis/IR transient absorption spectroscopy, temperature dependence studies, and theoretical calculations are required to further examine this interpretation, we believe that this electronegative bond-selective photolysis, using a wide range of light wavelengths from blue to red can become a fundamental technology for these applications.

## Methods

### General procedure for the C(sp^2^)–H chlorination of aldehydes (3, Fig. 2a)

Aldehyde **2** freshly passed through a basic silica pad (0.01 mmol), **1a** or **1c** (0.11 mmol), and CCl_4_ (0.20 mL) or MeCN (0.10 mL) were placed in a 3 mL screw cap vial. The vial was capped and wrapped with a Teflon seal. The mixture was stirred at room temperature under argon with blue LEDs irradiation for 18 h. To the resulting mixture, Et_3_N (0.30 mL) and a THF solution of HNMe_2_ (2.0 mol/L, 0.5 mL) were added, and the mixture was stirred at room temperature under argon for an additional period of 2 h. The final mixture was concentrated under reduced pressure and purified by silica gel preparative thin-layer chromatography (PTLC) to give the desired product **3**.

### General procedure for the aminochlorination of olefins (5, Fig. 2b)

Alkene **4** (0.50 mmol), **1a** or **1c** (0.55 mmol), and CH_2_Cl_2_ (1.0 mL) were placed in a 3 mL screw cap vial. The vial was capped and wrapped with a Teflon seal. The mixture was stirred at room temperature under argon with blue LEDs irradiation for 18 h. The final mixture was concentrated under reduced pressure and purified by silica gel PTLC to give the desired product **5**.

### General procedure for the C(sp^2^)–H fluorination of aldehydes (6, Fig. 2c)

Aldehyde **3**, freshly passed through a basic silica pad, (0.20 mmol), **1b** (0.20 mmol), and MeCN (0.40 mL) were placed in a 3 mL screw cap vial. The vial was capped and wrapped with a Teflon seal. The mixture was stirred at room temperature under argon with blue LEDs irradiation for 18 h. The final mixture was concentrated under reduced pressure and purified by silica gel PTLC to give the desired product **6**.

### Procedure for the allylic chlorination of cyclohexene (7d, Fig. 2d)

Cyclohexene (**4d**, 0.50 mmol), **1d** (1.0 mmol), and CH_2_Cl_2_ (1.0 mL) were placed in a 3 mL screw cap vial. The vial was capped and wrapped with a Teflon seal. The mixture was stirred at room temperature with red LEDs irradiation for 8 h to give the desired crude product **7d**, the yield of which was determined by ^1^H NMR using mesitylene as an internal standard.

### Supplementary information

Supplementary information

Description of Additional Supplementary Files

Supplementary Data 1

Supplementary Data 2

## Data Availability

The data that supports the findings of the present study are available in this article and its Supplementary Information, which includes materials and methods, synthetic experiments, computational studies, Supplementary Fig. 1, Supplementary Tables 1–17, Supplementary Data 1 for computational studies, and Supplementary Data 2 (Supplementary Figs. 2–80) for NMR spectra.
