# Supplementary material for: Vibration-mediated long-wavelength photolysis of electronegative bonds beyond S0–S1 and S0–T1 transitions
Source: Commun Chem. 2024 Jun 4;7:126. doi: 10.1038/s42004-024-01208-0 (PMC11150518; doi:10.1038/s42004-024-01208-0)
Supplement: Supplementary file 1 — Supplementary information [file 42004_2024_1208_MOESM1_ESM.pdf]

Supplementary Information

Vibration-mediated long-wavelength photolysis of electronegative bonds beyond S_0–S_1 and S_0–T_1 transitions

Antônio Junio Araujo Dias,[1] Atsuya Muranaka,[2] Masanobu Uchiyama,[3] Ken Tanaka,[1,]* and Yuki Nagashima[1,]*

[1] Department of Chemical Science and Engineering, Tokyo Institute of Technology, O-okayama, Meguro-ku, Tokyo 152-8550, Japan.
[2] Molecular Structure Characterization Unit, RIKEN Center for Sustainable Resource Science, 2-1 Hirosawa, Wako, Saitama 351-0198, Japan
[3] Graduate School of Pharmaceutical Sciences, The University of Tokyo, 7-3-1 Hongo, Bunkyo-ku, Tokyo 113-0033, Japan.
*e-mail: nagashima.y.ae@m.titech.ac.jp; tanaka.k.cg@m.titech.ac.jp

Table of Contents

1. Supplementary Methods S2

2. Experimental section S3
   2-1. C(sp^2)–H chlorination of aldehydes (Fig. 2a) S3
   2-2. Aminochlorination of alkenes (Fig. 2b) S8
   2-3. C(sp^2)–H fluorination of aldehydes (Fig. 2c) S10
   2-4. Allylic C(sp^3)–H chlorination of cyclohexene (Fig. 2d) S11

3. Computational details S12
   3-1. Theoretical prediction of S_0–S_1 and S_0–T_1 transition (Table 1) S12
   3-2. Potential energy surface (Fig. 4) S17
   3-3. Vibrational analysis S26

4. Spectroscopical details S29

5. Supplementary References S30

1. Supplementary Methods

Instrumentation.

^{1}H, ^{13}C, and ^{19}F NMR spectra were collected on a Bruker AVANCE III HD 400 spectrometer at ambient temperature. Chemical shifts are expressed in δ (ppm) values, and coupling constants are expressed in hertz (Hz). All ^{1}H NMR experiments are reported in δ units, parts per million (ppm), and were measured relative to the signals for residual chloroform (7.26 ppm). All ^{13}C NMR spectra are reported in ppm relative to deuterochloroform (77.16 ppm) and were obtained with ^{1}H decoupling. The following abbreviations are used: s = singlet, d = doublet, t = triplet, q = quartet, m = multiplet, and brs = broad singlet. ESI mass spectra (HRMS analyses) were measured on a Bruker micrOTOF Focus II spectrometer. UV-vis absorption spectra were recorded on a JASCO V-630 spectrophotometer or a JASCO V-770 spectrophotometer. Fluorescence spectra were recorded on a JASCO FP-6600. Melting points were determined with a Mettler Toledo MP50 One Click Melting Point System and are uncorrected. All reactions were carried out in oven-dried glassware with magnetic stirring. Photoreactions were performed with a Kessil A160WE TUNA Blue for reactions with blue light, a Kessil PR160-525 for reactions with green light, and an iNextStation SMD5050 red LED Tape Light for reactions with red light. The irradiance of the LEDs used was measured with a TENMARS TM-206 solar power meter.

Materials.

Unless otherwise noted, materials were purchased from Aldrich Inc., Wako Pure Chemical Industries, Ltd., Tokyo Kasei Co., and other commercial suppliers and were used after appropriate purification. Anhydrous CCl_4 (No. 030-15731), CH_2Cl_2 (No. 041-32345), and cyclohexane (No. 032-22445) were obtained from Wako and used as received. Anhydrous MeCN (No. 27,100-4) was obtained from Aldrich and used as received. Solvents for the synthesis of substrates were dried over Molecular Sieves 4Å (Wako) prior to use. All other reagents were obtained from commercial sources and used as received. Preparative thin-layer chromatography (PTLC) was performed with silica gel 60 from Merck.

2. Experimental Section

2.1. C(sp^2)–H chlorination of aldehydes (Fig. 2a)

Representative procedure for the C(sp^2)–H chlorination of aldehydes (3a, Fig. 2)

Method A: Benzaldehyde **2a** (10.6 mg, 0.10 mmol), freshly passed through a basic alumina pad, *N*-chlorosuccinimide **1a** (14.7 mg, 0.11 mmol), and CCl_4 (0.20 mL) were placed in a 3 mL screw cap vial. The vial was capped and wrapped with a Teflon seal. The mixture was stirred at room temperature under Ar with blue LEDs irradiation for 18 h. To the resulting mixture were added triethylamine (0.30 mL) and a THF solution of dimethylamine (2 mol/L, 0.50 mL) and the mixture was stirred at room temperature under Ar for an additional period of 2 h. The final mixture was concentrated under reduced pressure, and to the residue were added 1,3,5-trimethylbenzene (as an internal standard) and $CDCl_3$. NMR crude yield was analyzed by ^{1}H NMR. Finally, the crude product was purified by PTLC to afford the desired product **3a**.

Method A': Benzaldehyde **2a** (10.6 mg, 0.1 mmol), freshly passed through a basic silica pad, *N*-chlorosuccinimide **1a** (14.7 mg, 0.11 mmol), and MeCN (0.10 mL) were placed in 3 mL screw cap vial. Then, the remaining procedure was executed as in method A.

Method B: Benzaldehyde **2a** (10.6 mg, 0.1 mmol), freshly passed through a basic silica pad, *N*-chlorosaccharin **1c** (23.9 mg, 0.11 mmol), and CCl_4 (0.2 mL) were placed in 3 mL screw cap vial. Then, the remaining procedure was executed as in method A except by using green LEDs irradiation.

Supplementary Table 1. Solvent screening for the C(sp^2)–H chlorination of aldehydes. **2a** (10.6 mg, 0.10 mmol), **1a** (14.7 mg, 0.11 mmol), 0.5 M concentration.

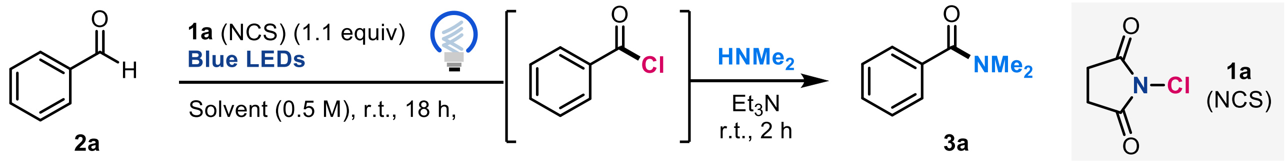

Entry	Solvent	Yield (%)
1	CH_2Cl_2	44
2	neat	47
3	MeCN	46
4	Cyclohexane	13
5	EtOAc	20
6	H_2O	0
7	MeOH	0
8	THF	0
9	CCl_4	83

***N,N*-dimethylbenzamide (3a, Fig. 2)**[1]

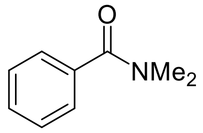

Colorless oil; **Method A:** 83% NMR crude yield. 11.4 mg, 0.076 mmol, 76% yield. **Method A':** 55% NMR crude yield. 6.3 mg, 0.042 mmol, 42% yield. **Method B:** 86% NMR crude yield. 13.0 mg, 0.087 mmol, 87% yield. Purified by PTLC (*n*-hexane/EtOAc = 3:1), followed by a wash with water, extraction with CH_2Cl_2, and drying with Na_2SO_4. **^{1}H NMR (400 MHz, $CDCl_3$)** 7.45–7.35 (m, 5H), 3.12 (brs, 3H), 2.98 (brs, 3H); **^{13}C NMR ($CDCl_3$, 100 MHz)** δ 171.8, 136.5, 129.6, 128.5, 127.2, 39.7, 35.5.

***N,N*-dimethyl-[1,1'-biphenyl]-4-carboxamide (3b, Fig. 2)**[1]

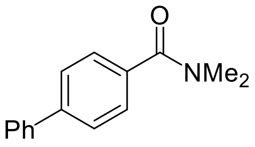

Colorless solid; 12.4 mg, 0.055 mmol, 55% yield, purified by PTLC (*n*-hexane/EtOAc = 1:3). **^{1}H NMR (400 MHz, $CDCl_3$)** δ 7.65-7.57 (m, 4H), 7.52–7.42 (m, 4H), 7.39–7.33 (m, 1H), 3.13 (brs, 3H), 3.04 (brs, 3H); **^{13}C NMR ($CDCl_3$, 100 MHz)** δ 171.6, 142.6, 140.5, 135.2, 129.0, 127.9, 127.8, 127.3, 127.2, 39.8, 35.5.

***N*,*N*-dimethyl-(*p*-methoxycarbonyl)-benzamide (3c, Fig. 2)**[2]

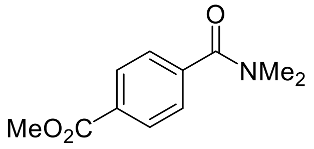

Colorless solid; 15.7 mg, 0.076 mmol, 76% yield, purified by PTLC (*n*-hexane/EtOAc = 2:3), followed by a wash with water, extraction with CH_2Cl_2, and drying with Na_2SO_4. **^{1}H NMR (400 MHz, $CDCl_3$)** δ 8.10–8.05 (m, 2H), 7.51–7.44 (m, 2H), 3.94 (s, 3H), 3.13 (s, 3H), 2.95 (s, 3H); **^{13}C NMR ($CDCl_3$, 100 MHz)** δ 170.7, 166.5, 140.8, 131.1, 129.9, 127.1, 52.4, 39.5, 35.4.

4-chloro-*N*,*N*-dimethylbenzamide (3d, Fig. 2)[2]

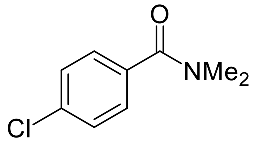

Colorless solid; **Method A:** Reaction performed on 0.25mmol scale; 36.0 mg, 0.196 mmol, 78% yield. **Method A':** 85% NMR crude yield. 15.3 mg, 0.084 mmol, 84% yield. **Method B:** 15.6 mg, 0.085 mmol, 85% yield. Purified by PTLC ($CHCl_3$/MeOH = 30:1). **^{1}H NMR (400 MHz, $CDCl_3$)** δ 7.41–7.33 (m, 4H), 3.10 (brs, 3H), 2.98 (brs, 3H); **^{13}C NMR ($CDCl_3$, 100 MHz)** δ 170.6, 135.7, 134.8, 128.7, 39.7, 35.5.

3-chloro-*N*,*N*-dimethylbenzamide (3e, Fig. 2)[3]

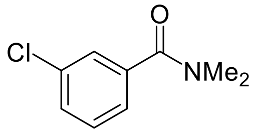

Colorless oil; 15.6 mg, 0.085 mmol, 85% yield, purified by PTLC (*n*-hexane/EtOAc = 1:3). **^{1}H NMR (400 MHz, $CDCl_3$)** δ 7.43–7.37 (m, 2H), 7.34 (t, J = 7.5 Hz, 1H), 7.29 (dt, J = 7.4, 1.5 Hz, 1H), 3.11 (brs, 3H), 2.98 (brs, 3H); **^{13}C NMR ($CDCl_3$, 100 MHz)** δ 170.2, 138.2, 134.6, 129.9, 129.8, 127.4, 125.3, 39.6, 35.5.

2-chloro-*N*,*N*-dimethylbenzamide (3f, Fig. 2)[3]

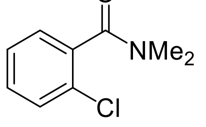

Colorless oil; 16.2 mg, 0.088 mmol, 88% yield, purified by PTLC (*n*-hexane/EtOAc = 1:3). **^{1}H NMR (400 MHz, $CDCl_3$)** δ 7.43–7.36 (m, 1H), 7.35–7.27 (m, 3H), 3.14 (s, 3H), 2.86 (s, 3H); **^{13}C NMR ($CDCl_3$, 100 MHz)** δ 168.6, 136.5, 130.5, 130.2, 129.7, 127.9, 127.3, 38.2, 34.8.

***N*-(*tert*-butyl)-4-fluorobenzamide (3g, Fig. 2)**[4]

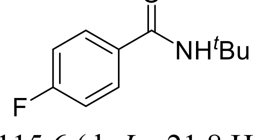

Colorless solid; 14.2 mg, 0.073 mmol, 73% yield, purified by PTLC (*n*-hexane/EtOAc = 10:1). **^{1}H NMR (400 MHz, $CDCl_3$)** δ 7.76–7.69 (m, 2H), δ 7.12–7.04 (m, 2H), 5.87 (brs, 1H), 1.47 (s, 9H); **^{13}C NMR ($CDCl_3$, 100 MHz)** δ 166.0, 164.6 (d, J = 251.1 Hz), 132.3 (d, J = 3.2 Hz), 129.1 (d, J = 8.8 Hz), 115.6 (d, J = 21.8 Hz), 51.9, 29.0; **^{19}F NMR ($CDCl_3$, 376 MHz)** δ -109.1.

***N*,*N*-dimethyl-4-(trifluoromethyl)benzamide (3h, Fig. 2)**[1]

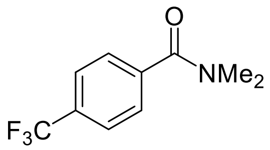

Colorless solid; 19.3 mg, 0.089 mmol, 89% yield, purified by PTLC (*n*-hexane/EtOAc = 1:3). **^{1}H NMR (400 MHz, $CDCl_3$)** δ 7.71–7.64 (m, 2H), 7.58–7.49 (m, 2H), 3.13 (s, 3H), 2.97 (s, 3H); **^{13}C NMR ($CDCl_3$, 100 MHz)** δ 170.3, 140.1 (d, J = 1.2 Hz), 131.7 (q, J = 32.7 Hz), 127.5, 125.6 (q, J = 3.8 Hz), 123.9 (q, J = 272.3 Hz), 39.5, 35.5.

4-bromo-*N*,*N*-dimethylbenzamide (3i, Fig. 2)[2]

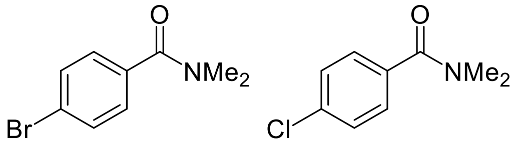

mixture 89/11

Colorless solid; 18.3 mg, 0.074 mmol, 74% yield, purified by PTLC (*n*-hexane/EtOAc = 1:3) and isolated as a mixture of the major 4-Br and of the corresponding 4-Cl derivate (12%).[2] **^{1}H NMR (400 MHz, $CDCl_3$)** δ 7.57–7.50 (m, 2H), 7.33–7.27 (m, 2H), 3.10 (brs, 3H), 2.97 (brs, 3H); **^{13}C NMR ($CDCl_3$, 100 MHz)** δ 170.7, 135.3, 131.7, 129.0, 124.0, 39.7, 35.5.

2-bromo-4-chloro-*N*,*N*-dimethylbenzamide (3j, Fig. 2)

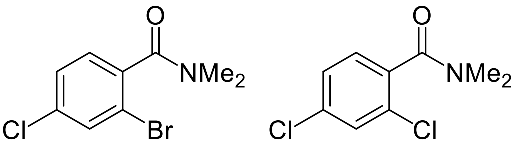

mixture 84/16

Yellow oil; 18.2 mg, 0.069 mmol, 69% yield, purified by PTLC (*n*-hexane/EtOAc = 1:3), and isolated as a mixture of the major 2-Br and of the corresponding 2-Cl derivate (16%).[5] **^{1}H NMR (400 MHz, $CDCl_3$)** δ 7.60 (d, J = 2.0 Hz, 1H), 7.35 (dd, J = 8.2, 3.8 Hz, 1H), 7.21 (d, J = 8.2 Hz, 1H), 3.13 (s, 3H), 2.86 (s, 3H); **^{13}C NMR ($CDCl_3$, 100 MHz)** δ 168.4, 137.2, 135.5, 132.6, 128.7, 128.2, 119.8, 38.3, 34.8. **HRMS** (ESI) *m*/*z*: $[M+Na]^+$ Calcd for $C_9H_9BrClNNaO$ 283.9448; Found 283.9448.

2-cyano-*N*,*N*-dimethylbenzamide (3k, Fig. 2)[6]

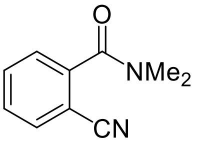

Colorless oil; 7.1 mg, 0.041 mmol, 41% yield, purified by PTLC (*n*-hexane/EtOAc = 2:3), followed by a wash with water, extraction with CH_2Cl_2, and drying with Na_2SO_4. **^{1}H NMR (400 MHz, $CDCl_3$)** δ 7.74–7.69 (m, 1H), 7.65 (td, J = 11.5, 1.3 Hz, 1H), 7.54–7.45 (m, 1H), 3.18 (s, 3H), 2.95 (s, 3H); **^{13}C NMR ($CDCl_3$, 100 MHz)** δ 167.8, 140.7, 133.2, 133.0, 129.6, 127.8, 117.0, 110.2, 38.7, 35.3.

***N*-(*tert*-butyl)-4-methylbenzamide (3l, Fig. 2)**[4]

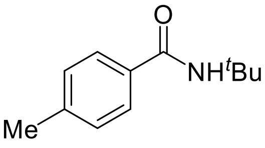

Colorless solid; 14.3 mg, 0.075 mmol, 75% yield, purified by PTLC (*n*-hexane/EtOAc = 10:1). **^{1}H NMR (400 MHz, $CDCl_3$)** δ 7.64–7.59 (m, 2H), 7.20 (d, J = 7.9 Hz, 2H), 5.91 (brs, 1H), 2.38 (s, 3H), 1.47 (s, 9H); **^{13}C NMR ($CDCl_3$, 100 MHz)** δ 167.0, 141.5, 133.2, 129.2, 126.8, 51.6, 29.1, 21.5.

4-methoxy-*N*,*N*-dimethylbenzamide (3m, Fig. 2)[1]

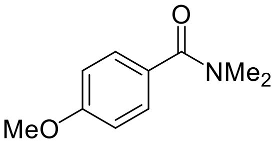

Colorless oil; 9.5 mg, 0.053 mmol, 53% yield, purified by PTLC (*n*-hexane/EtOAc = 1:3). **^{1}H NMR (400 MHz, $CDCl_3$)** δ 7.43–7.37 (m, 2H), 6.94–6.87 (m, 2H), 3.83 (s, 3H), 3.05 (brs, 6H); **^{13}C NMR ($CDCl_3$, 100 MHz)** δ 171.7, 160.7, 129.3, 128.6, 113.7, 55.5, 39.9, 35.7.

3-methoxy-*N*,*N*-dimethylbenzamide (3n, Fig. 2)[1]

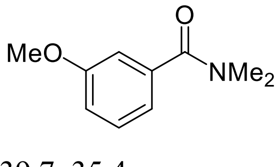

Colorless oil; 8.5 mg, 0.047 mmol, 47% yield, purified by PTLC (*n*-hexane/EtOAc = 1:4). **^{1}H NMR (400 MHz, $CDCl_3$)** δ 7.33–7.27 (m, 1H), 7.00–6.91 (m, 3H), 3.82 (s, 3H), 3.11 (brs, 3H), 2.98 (brs, 3H); **^{13}C NMR ($CDCl_3$, 100 MHz)** δ 171.5, 159.7, 137.8, 129.6, 119.3, 115.6, 112.5, 55.5, 39.7, 35.4.

2-methoxy-*N*,*N*-dimethylbenzamide (3o, Fig. 2)[1]

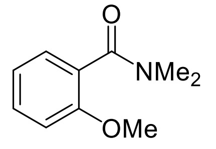

Colorless oil; 6.7 mg, 0.037 mmol, 37% yield, purified by PTLC (*n*-hexane/EtOAc =1:4). **^{1}H NMR (400 MHz, $CDCl_3$)** δ 7.34 (ddd, J = 8.3, 7.5, 1.7 Hz, 1H), 7.23 (dd, J = 7.4, 1.7 Hz, 1H), 6.97 (td, J = 11.2, 0.9 Hz, 1H), 6.91 (d, J = 8.4 Hz, 1H), 3.84 (s, 3H), 3.11 (s, 3H), 2.85 (s, 3H); **^{13}C NMR ($CDCl_3$, 100 MHz)** δ 169.6, 155.5, 130.4, 128.0, 126.6, 121.0, 111.1, 55.7, 38.4, 34.9.

***N*,*N*-dimethyl-2-naphthamide (3p, Fig. 2)**[1]

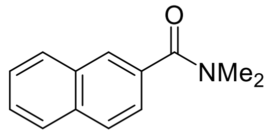

Colorless solid; 10.0 mg, 0.050 mmol, 50% yield, purified by PTLC ($CHCl_3$/MeOH = 30:1). **^{1}H NMR (400 MHz, $CDCl_3$)** δ 7.94–7.90 (m, 1H), 7.90–7.82 (m, 3H), 7.57–7.48 (m, 3H), 3.16 (brs, 3H), 3.04 (brs, 3H); **^{13}C NMR ($CDCl_3$, 100 MHz)** δ 171.8, 133.80, 133.76, 132.8, 128.5, 128.3, 127.9, 127.1, 127.0, 126.7, 124.6, 39.8, 35.6.

***N*-(*tert*-butyl)thiophene-2-carboxamide (3q, Fig. 2)**[7]

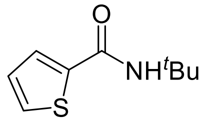

Colorless solid; 11.3 mg, 0.062 mmol, 62% yield, purified by PTLC (*n*-hexane/EtOAc = 10:1). **^{1}H NMR (400 MHz, $CDCl_3$)** δ 7.44–7.39 (m, 2H), 7.04 (dd, J = 4.9, 3.8 Hz, 1H), 5.80 (brs, 1 H), 1.46 (s, 9 H); **^{13}C NMR ($CDCl_3$, 100 MHz)** δ 161.4, 140.7, 129.5, 127.58, 127.55, 52.1, 29.1.

***N*-(*tert*-butyl)thiophene-3-carboxamide (3r, Fig. 2)**[4]

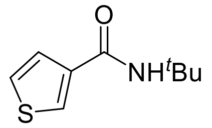

Colorless solid; 6.2 mg, 0.034 mmol, 34% yield, purified by PTLC (*n*-hexane/EtOAc = 10:1). **^{1}H NMR (400 MHz, $CDCl_3$)** δ 7.77 (dd, J = 2.8, 1.6 Hz, 1H), 7.34–7.29 (m, 2H), 5.76 (brs, 1 H), 1.46 (s, 9 H); **^{13}C NMR ($CDCl_3$, 100 MHz)** δ 162.7, 139.1, 127.6, 126.5, 126.1, 51.8, 29.1.

***N*,*N*-dimethyl-3-phenylpropanamide (3s, Fig. 2)**[2]

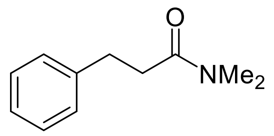

Colorless oil; 4.8 mg, 0.027 mmol, 27% yield, purified by PTLC (*n*-hexane/EtOAc = 1:1). **^{1}H NMR (400 MHz, $CDCl_3$)** δ 7.32–7.27 (m, 2H), 7.25–7.17 (m, 3H), 3.00–2.94 (m, 5H), 2.93 (s, 3H), 2.62 (t, J = 8.0 Hz, 2H); **^{13}C NMR ($CDCl_3$, 100 MHz)** δ 172.3, 141.7, 128.61, 128.58, 126.2, 37.3, 35.6, 35.5, 31.5.

***N*-(*tert*-butyl)cyclopropanecarboxamide (3t, Fig. 2)**[8]

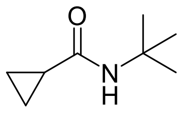

Colorless solid; **Method A:** 99% NMR crude yield. 10.0 mg, 0.071 mmol, 71% yield. **Method A'** 63% NMR crude yield. 4.8 mg, 0.034 mmol, 34% yield. purified by PTLC (*n*-hexane/EtOAc = 3:1). **^{1}H NMR (400 MHz, $CDCl_3$)** δ 5.44 (brs, 1H), 1.35 (s, 9H), 1.28–1.20 (m, 1H), 0.93–0.87 (m, 2H), 0.70–0.62 (m, 2H); **^{13}C NMR ($CDCl_3$, 100 MHz)** δ 172.7, 51.3, 29.1, 15.6, 6.8.

***N*-(*tert*-butyl)cyclohexanecarboxamide (3u, Fig. 2)**[8]

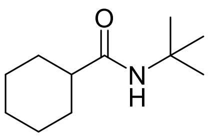

Colorless solid; 10.6 mg, 0.058 mmol, 58% yield, purified by PTLC (*n*-hexane/EtOAc = 3:1). **^{1}H NMR (400 MHz, $CDCl_3$)** δ 5.23 (brs, 1H), 1.99–1.89 (m, 1H), 1.86–1.71 (m, 4H), 1.69–1.61 (m, 1H), 1.42–1.20 (m, 5H), 1.33 (s, 9H); **^{13}C NMR ($CDCl_3$, 100 MHz)** δ 175.7, 50.9, 46.5, 29.9, 29.0, 25.9.

***N*-(*tert*-butyl)pivalamide (3v, Fig. 2)**[9]

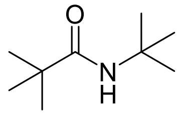

Colorless solid; 3.3 mg, 0.021 mmol, 21% yield, purified by PTLC (*n*-hexane/EtOAc = 3:1). **^{1}H NMR (400 MHz, $CDCl_3$)** δ 5.39 (brs, 1H), 1.34 (s, 9H), 1.16 (s, 9H); **^{13}C NMR ($CDCl_3$, 100 MHz)** δ 178.0, 50.8, 39.1, 28.9, 27.8.

***N*-*tert*-butylbenzamide (3ab, Fig. 2)**[4]

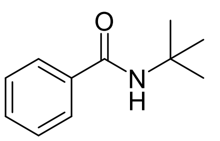

Colorless solid; 14.9 mg, 0.084 mmol, 84% yield, purified by PTLC (*n*-hexane/EtOAc = 7:3). **^{1}H NMR (400 MHz, $CDCl_3$)** δ 7.74–7.69 (m, 2H), 7.50–7.44 (m, 1H), 7.44–7.37 (m, 2H), 5.93 (brs, 1H), 1.48 (s, 9H); **^{13}C NMR ($CDCl_3$, 100 MHz)** δ 167.0, 136.1, 131.2, 128.6, 126.8, 51.7, 29.0.

***N*-propylbenzamide (3ac, Fig. 2)**[10]

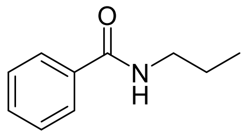

Colorless solid; 15.2 mg, 0.093 mmol, 93% yield, purified by PTLC (*n*-hexane/EtOAc = 1:1). **^{1}H NMR (400 MHz, $CDCl_3$)** δ 7.78–7.73 (m, 2H), 7.53–7.46 (m, 1H), 7.46–7.39 (m, 2H), 6.16 (brs, 1H), 3.47–3.38 (m, 2H), 1.71–1.59 (m, 2H), 0.99 (t, J = 7.4 Hz, 3H); **^{13}C NMR ($CDCl_3$, 100 MHz)** δ 167.7, 135.1, 131.4, 128.7, 127.0, 41.9, 23.1, 11.6.

***N*-benzylbenzamide (3ad, Fig. 2)**[11]

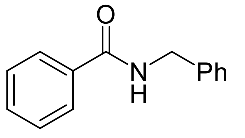

Pale yellow solid; Reaction performed on 0.25 mmol scale; 36.4 mg, 0.172 mmol, 69% yield, purified by PTLC (CH_2Cl_2/EtOAc = 10:1). **^{1}H NMR (400 MHz, $CDCl_3$)** δ 7.82–7.80 (m, 2H), 7.52–7.45 (m, 1H), 7.45–7.37 (m, 2H), 7.37–7.27 (m, 5H), 6.55 (brs, 1H), 4.62 (d, J = 5.7 Hz, 2H); **^{13}C NMR ($CDCl_3$, 100 MHz)** δ 167.5, 138.3, 134.5, 131.7, 128.9, 128.7, 128.1, 127.8, 127.1, 44.3.

***N*-benzoylmorpholine (3ae, Fig. 2)**[12]

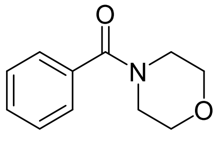

Colorless solid; Reaction performed on 0.25 mmol scale; 35.5 mg, 0.186 mmol, 74% yield, purified by PTLC (*n*-hexane/EtOAc = 2:3). **^{1}H NMR (400 MHz, $CDCl_3$)** δ 7.44–7.38 (m, 5H), 3.87–3.34 (m, 8H); **^{13}C NMR ($CDCl_3$, 100 MHz)** δ 170.5, 135.4, 129.9, 128.6, 127.2, 67.0, 48.3, 42.7.

2.2. Aminochlorination of alkenes (Fig. 2b)

Representative procedure for the aminochlorination of olefins (5aa, 5dc Fig. 2)

Method A: 1,2-dihydrofuran **4a** (35.1 mg, 0.50 mmol), *N*-chlorosuccinimide **1a** (73.4 mg, 0.55 mmol), and CH_2Cl_2 (1.0 mL) were placed in a 3 mL screw cap vial. The vial was capped and wrapped with a Teflon seal. The mixture was stirred at room temperature under Ar with blue LEDs irradiation for 18 h. The final mixture was concentrated under reduced pressure and to the residue were added 1,3,5-trimethylbenzene (as an internal standard) and $CDCl_3$. NMR crude yield was analyzed by ^1H NMR. Finally, the crude product was purified by PTLC to afford the desired product **5aa**.

Method B: Cyclohexene **4d** (8.2 mg, 0.10 mmol), *N*-chlorosaccharin **1c** (23.9 mg, 0.11 mmol), and CH_2Cl_2 (0.2 mL) were placed in a 3 mL screw cap vial. Then, the remaining procedure was executed as in method A except by using green LEDs irradiation to afford the desired product **5dc**.

1-(3-chlorotetrahydrofuran-2-yl)pyrrolidine-2,5-dione (5aa, Fig. 2)

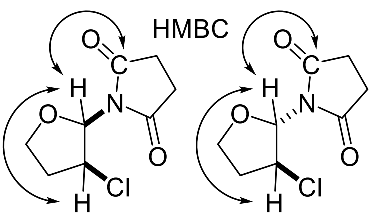

dr = 1.6:1 mixture

Colorless oil; 69.7 mg, 0.342 mmol, 68% yield, purified by PTLC (*n*-hexane/EtOAc = 2:1) and isolated as a mixture of diastereomers. Regioselectivity was confirmed through HMBC and the different diastereomers were assigned based on the coupling constant of the hydrogens as shown.[13] **Major diastereomer *cis*: ^{1}H NMR (400 MHz, $CDCl_3$)** δ 5.79 (d, J = 3.8 Hz, 1H), 4.95–4.87 (m, 1H), 4.28–4.20 (m, 1H), 4.15–4.07 (m, 1H), 2.90–2.79 (m, 1H), 2.72 (s, 4H), 2.31-2.20 (m, 1H); **^{13}C NMR ($CDCl_3$, 100 MHz)** δ 176.4, 88.5, 69.0, 57.4, 36.9, 28.21. **Minor diastereomer *trans*: ^{1}H NMR (400 MHz, $CDCl_3$)** δ 5.97 (d, J = 6.9 Hz, 1H), 4.59–4.50 (m, 1H), 4.45–4.37 (m, 1H), 3.97–3.90 (m, 1H), 2.72 (s, 4H), 2.70–2.58 (m, 1H), 2.57–2.47 (m, 1H); **^{13}C NMR ($CDCl_3$, 100 MHz)** δ 176.5, 83.1, 69.3, 56.6, 35.0, 28.17. **HRMS** (ESI) *m/z*: $[M+Na]^+$ Calcd for $C_8H_{10}ClNNaO_3$ 226.0241; Found 226.0241.

1-((1-chlorocyclohexyl)methyl)pyrrolidine-2,5-dione (5ba, Fig. 2)

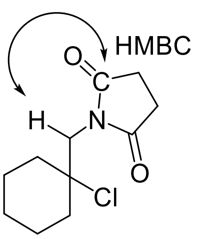

Colorless solid; **mp** 108.4–109.6 ºC; 20.1 mg, 0.088 mmol, 18% yield, purified by PTLC (*n*-hexane/EtOAc = 1:1). Regioselectivity was confirmed through HMBC as shown. **^{1}H NMR (400 MHz, $CDCl_3$)** δ 3.82 (s, 2H), 2.76 (s, 4H), 1.92–1.82 (m, 2H), 1.78–1.50 (m, 7H), 1.23–1.08 (m, 1H); **^{13}C NMR ($CDCl_3$, 100 MHz)** δ 177.2, 74.0, 50.2, 38.1, 28.2, 25.1, 21.8. **HRMS** (ESI) *m/z*: $[M+Na]^+$ Calcd for $C_{11}H_{16}ClNNaO_2$ 252.0762; Found 252.0762.

Methyl 9-chloro-10-(2,5-dioxopyrrolidin-1-yl)decanoate (5ca, Fig. 2)

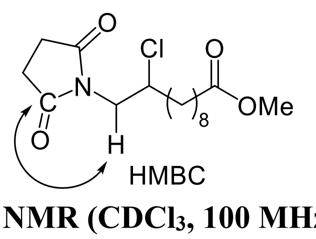

Colorless oil; 44.3 mg, 0.133 mmol, 27% yield, purified by PTLC (*n*-hexane/EtOAc = 1:1). Regioselectivity was confirmed through HMBC as shown. **^{1}H NMR (400 MHz, $CDCl_3$)** δ 4.24 (sep, J = 4.5 Hz, 1H), 3.87 (dd, J = 13.7, 9.5 Hz, 1H), 3.67 (s, 3H), 3.62 (dd, J = 13.8, 4.6 Hz, 1H), 2.75 (s, 4H), 2.30 (t, J = 7.5 Hz, 2H), 1.81–1.54 (m, 5H), 1.51–1.20 (m, 9H); **^{13}C NMR ($CDCl_3$, 100 MHz)** δ 177.0, 174.4, 58.7, 51.6, 45.1, 35.9, 34.2, 29.3, 29.19, 29.17, 29.0, 28.2, 26.2, 25.0. **HRMS** (ESI) *m/z*: $[M+Na]^+$ Calcd for $C_{16}H_{26}ClNNaO_4$ 354.1443; Found 354.1442.

2-(2-chlorocyclohexyl)benzo[*d*]isothiazol-3(2*H*)-one 1,1-dioxide (5dc, Fig. 2)[14]

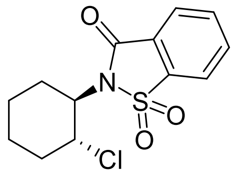

Colorless solid; 13.6 mg, 0.045 mmol, 45% yield, purified by PTLC (*n*-hexane/EtOAc = 3:1). **^{1}H NMR (400 MHz, CDCl$_3$)** δ 8.10–8.01 (m, 1H), 7.93–7.78 (m, 3H), 4.73 (brs, 1H), 4.18 (brs, 1H), 2.50–2.36 (m, 1H), 2.31–2.15 (m, 2H), 1.95–1.81 (m, 2H), 1.81–1.68 (m, 1H), 1.53–1.38 (m, 2H); **^{13}C NMR (CDCl$_3$, 100 MHz)** δ 158.9, 137.5, 134.9, 134.4, 127.2, 125.4, 120.9, 60.1, 58.8, 37.3, 31.0, 25.7, 25.4.

2-(2-chloro-1-phenylethyl)benzo[*d*]isothiazol-3(2*H*)-one 1,1-dioxide (5ec, Fig. 2)[14]

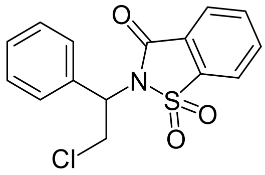

Colorless solid; 14.4 mg, 0.045 mmol, 45% yield, purified by PTLC (*n*-hexane/EtOAc = 3:1). **^{1}H NMR (400 MHz, CDCl$_3$)** δ 8.07–8.01 (m, 1H), 7.91–7.78 (m, 3H), 7.63–7.57 (m, 2H), 7.44–7.33 (m, 3H), 5.41 (dd, J = 9.1, 6.7 Hz, 1H), 4.68 (dd, J = 11.5, 9.2 Hz, 1H), 4.25 (dd, J = 11.5, 6.7 Hz, 1 H); **^{13}C NMR (CDCl$_3$, 100 MHz)** δ 158.9, 137.5, 135.1, 134.8, 134.5, 129.3, 129.0, 128.6, 127.1, 125.4, 121.1, 58.6, 42.3.

2-(2-chloro-1-(*p*-tolyl)ethyl)benzo[*d*]isothiazol-3(2*H*)-one 1,1-dioxide (5fc, Fig. 2)[14]

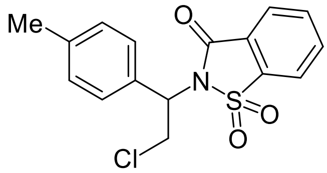

Colorless solid; 19.6 mg, 0.058 mmol, 58% yield, purified by PTLC (*n*-hexane/EtOAc = 3:1). **^{1}H NMR (400 MHz, CDCl$_3$)** δ 8.06–7.99 (m, 1H), 7.90–7.78 (m, 3H), 7.48 (d, J = 8.2 Hz, 2H), 7.20 (d, J = 7.9 Hz, 2H), 5.38 (dd, J = 9.1, 6.8 Hz, 1H), 4.66 (dd, J = 11.4, 9.1 Hz, 1H), 4.23 (dd, J = 11.4, 6.8 Hz, 1H), 2.34 (s, 3 H); **^{13}C NMR (CDCl$_3$, 100 MHz)** δ 158.9, 139.3, 137.5, 135.0, 134.5, 131.8, 129.7, 128.5, 127.2, 125.4, 121.0, 58.4, 42.4, 21.3.

2.3. C(sp^2)–H fluorination of aldehydes (Fig. 2c)

Representative procedure for the fluorination of aldehydes (6a Fig. 2):

Benzaldehyde **2a** (21.2 mg, 0.20 mmol), freshly passed through a basic alumina pad, *N*-fluorobenzenesulfonimide **1b** (63.1 mg, 0.20 mmol), and MeCN (0.4 mL) were placed in a 3 mL screw cap vial. The vial was capped and wrapped with a Teflon seal. The mixture was stirred at room temperature under Ar with blue LEDs irradiation for 18 h. The final mixture was concentrated under reduced pressure and to the residue were added 1,3,5-trimethylbenzene (as an internal standard) and $CDCl_3$. Then NMR yield was analyzed by ^{1}H NMR. Finally, the crude product was purified by column chromatography to afford the desired product **6a**.

Benzoyl fluoride (6a, Fig. 2)[15]

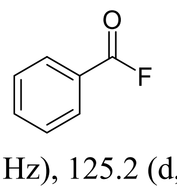

Colorless oil; 88% NMR crude yield. 12.4 mg, 0.100 mmol, 50% yield. Purified by column chromatography (*n*-hexane/Et_2O = 20:1). **^{1}H NMR (400 MHz, $CDCl_3$)** 8.09–8.01 (m, 2H), 7.74–7.67 (m, 1H), 7.57–7.50 (m, 2H); **^{13}C NMR ($CDCl_3$, 100 MHz)** δ 157.5 (d, J = 344.4 Hz), 135.4 (d, J = 0.9 Hz), 131.6 (d, J = 4.0 Hz), 129.2 (d, J = 1.2 Hz), 125.2 (d, J = 60.8 Hz); **^{19}F NMR ($CDCl_3$, 376 MHz)** δ 18.03.

4-chlorobenzoyl fluoride (6d, Fig. 2)[16]

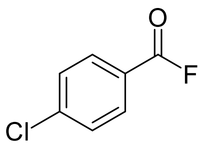

Colorless oil; 74% NMR crude yield. 15.6 mg, 0.098 mmol, 49% yield. Purified by column chromatography (*n*-hexane/Et_2O = 20:1). **^{1}H NMR (400 MHz, $CDCl_3$)** 8.02–7.96 (m, 2H), 7.55–7.48 (m, 2H); **^{13}C NMR ($CDCl_3$, 100 MHz)** δ 156.7 (d, J = 343.5 Hz), 142.4, 132.9 (d, J = 3.9 Hz), 129.7 (d, J = 1.1 Hz), 123.5 (d, J = 62.6 Hz); **^{19}F NMR ($CDCl_3$, 376 MHz)** δ 18.4.

4-methylbenzoyl fluoride/*N*-benzyl-4-methylbenzamide (6l/6l', Fig. 2)[16]

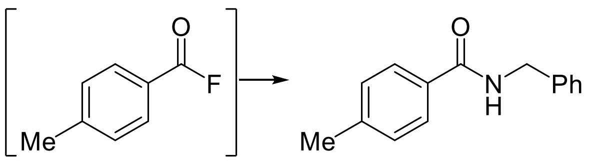

81% NMR crude yield. **6l: ^{1}H NMR (400 MHz, $CDCl_3$)** 7.93 (d, J = 8.2 Hz, 2H), 7.32 (d, J = 8.0 Hz, 2H), 2.46 (s, 3H); **^{19}F NMR ($CDCl_3$, 376 MHz)** δ 17.4. Isolated after amination due to the instability of the fluoride derivate, as reported. To the crude were added benzylamine (64.3, 0.60 mmol) and triethylamine (0.30 mL), and the mixture was stirred for 2 h.; **6l':** Colorless solid; 28.7 mg, 0.127 mmol, 64% yield in two steps. Purified by PTLC (*n*-hexane/EtOAc = 10:1). **^{1}H NMR (400 MHz, $CDCl_3$)** δ 7.68 (d, J = 8.2 Hz, 2H), 7.34 (d, J = 4.4 Hz, 4H), 7.33–7.26 (m, 1H), 7.21 (d, J = 7.8 Hz, 2H), 6.48 (s, 1H), 4.62 (d, J = 5.2 Hz, 2H), 2.38 (s, 3H); **^{13}C NMR ($CDCl_3$, 100 MHz)** δ 167.4, 142.1, 138.5, 131.7, 129.3, 128.9, 128.0, 127.7, 127.1, 44.2, 21.6.

3-phenylpropanoyl fluoride (6s, Fig. 2)[15]

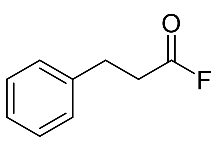

Colorless oil; 36% NMR crude yield. 7.5 mg, 0.049 mmol, 25% yield. Purified by column chromatography (*n*-hexane/Et_2O = 20:1). **^{1}H NMR (400 MHz, $CDCl_3$)** 7.35–7.28 (m, 2H), 7.26–7.18 (m, 3H), 3.00 (t, J = 7.6 Hz, 2H), 2.83 (t, J = 7.5 Hz, 2H); **^{13}C NMR ($CDCl_3$, 100 MHz)** δ 162.9 (d, J = 360.6 Hz), 139.0, 128.9, 128.4, 127.0, 34.0 (d, J = 50.6 Hz), 30.1 (d, J = 2.4 Hz). **^{19}F NMR ($CDCl_3$, 376 MHz)** δ 45.2.

2.4. Allylic C(sp^3)–H chlorination of cyclohexene (Fig. 2d)

Procedure for the allylic chlorination of cyclohexene 4d (7d Fig. 2)

Cyclohexene **4d** (41.1 mg, 0.50 mmol), *tert*-butyl hypochlorite **1d** (108.6 mg, 1.0 mmol), and CH_2Cl_2 (1.0 mL) were placed in a 3 mL screw cap vial. The vial was capped and wrapped with a Teflon seal. The mixture was stirred at room temperature inside a flask with red LED tape wrapped on the inside of it for 8 h to afford the crude mixture of **7d**. The final mixture was transferred to a Schlenk flask under air and 3.0 mL of triethylamine and aniline (93.1 mg, 1.0 mmol) were added. The resulting mixture was stirred at 80 °C for 2 h and rested at room temperature overnight. The crude product was purified by PTLC to afford *N*-(cyclohex-2-en-1-yl)aniline **7d'** as a colorless oil.

3-chlorocyclohex-1-ene/*N*-(cyclohex-2-en-1-yl)aniline (7d/7d', Fig. 2)[17,18]

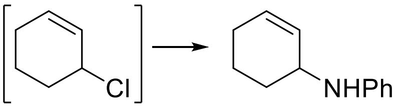

42% NMR crude yield. **7d: ^{1}H NMR (400 MHz, CDCl$_3$)** 5.91–5.84 (m, 1H), 5.84–5.77 (m, 1H), 4.65–4.58 (m, 1H), 2.18–1.97 (m, 4H), 1.94–1.82 (m, 1H), 1.70–1.61 (m, 1H). Isolated after amination as reported in the literature.[19] **7d':** Colorless oil; 13.8 mg, 0.080 mmol, 16% yield. Purified by PTLC (*n*-hexane/Et_2O = 30:1). **^{1}H NMR (400 MHz, CDCl$_3$)** 7.20–7.13 (m, 2H), 6.68 (t, J = 7.3, 1H), 6.65–6.59 (m, 2H), 5.89–5.81 (m, 1H), 5.79–5.71 (m, 1H), 3.99 (brs, 1H), 3.62 (brs, 1H), 2.11–1.96 (m, 2H), 1.95–1.84 (m, 1H), 1.77–1.68 (m, 1H), 1.68–1.60 (m, 2H); **^{13}C NMR (CDCl$_3$, 100 MHz)** δ 147.4, 130.3, 129.5, 128.8, 117.3, 113.4, 48.0, 29.1, 25.3, 19.8.

3. Computational details

All calculations were carried out with the Gaussian 16 program package.[20] The hybrid density functional method based on (U)M06[21–24] with a standard 6-31+G* basis set was used for preliminary geometry optimizations and in the BDE calculations. The 6-311+G** basis set was used to calculate the single-point energies and transitions for NCS, NCP, NFSI, *t*-BuOCl, and *N*-methylsuccinimide because it was envisaged that this strategy would provide greater accuracy with regard to the energetic information. Geometry optimization and vibrational analysis were performed at the same level. All stationary points were optimized without any symmetry assumptions and characterized by normal coordinate analysis at the same level of theory (number of imaginary frequencies, NIMAG, 0 for minima). Excitation wavelengths via S_0–S_1 transitions and oscillator strengths were obtained at the density functional level using the time-dependent perturbation theory (TD-DFT) approach.

3.1. Theoretical prediction of S_0–S_1 and S_0–T_1 transition (Table 1)

Supplementary Table 2. (TD)-DFT calculated transitions from the ground state (S_0) to singlet (S_1) and triplet (T_1) excited states and respective X–Y bond dissociation energies. (TD-)DFT calculations were performed at (U)M06/6-31+G* levels of theory unless otherwise noted. BDE = bond dissociation energy.

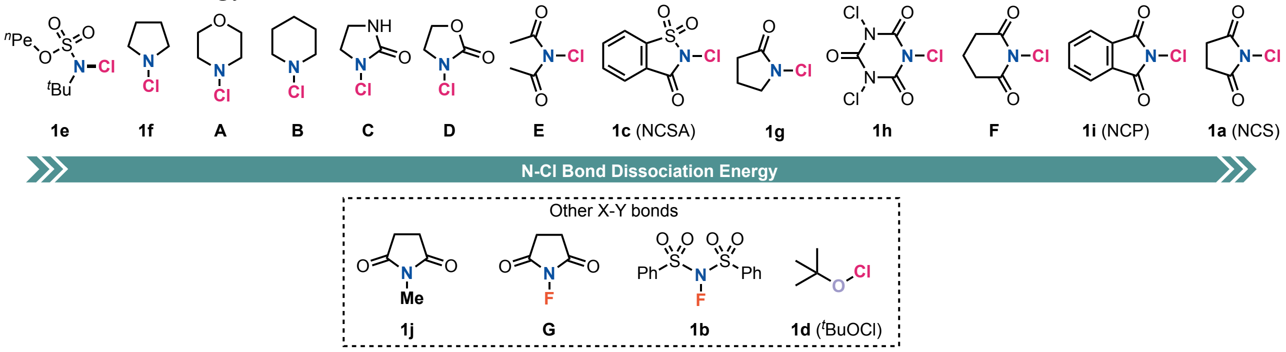

N–Cl	$S_0 \rightarrow S_1$ [nm]	ΔE (S_0–S_1) [kcal mol^{-1}]	$S_0 \rightarrow T_1$ [nm]	ΔE (S_0–T_1) [kcal mol^{-1}]	BDE [kcal mol^{-1} /(nm)]
**1e**	294	97.4	333	85.8	46.7 (612)
**1f**	294	97.2	363	78.8	50.2 (570)
**A**	319	89.6	389	73.5	52.2 (547)
**B**	311	91.9	382	74.9	53.5 (534)
**C**	273	104.7	324	88.2	54.5 (525)
**D**	270	105.9	320	89.5	54.9 (521)
**E**	271	105.5	313	91.4	55.3 (517)
**1c** (NCSA)	277	103.2	408	70.0	58.2 (491)
**1g**	270	105.9	316	90.6	60.1 (476)
**1h**	253	113.0	333	85.8	62.5 (457)
**F**	250	114.5	252	113.4	66.2 (432)
**1i** (NCP)	313	91.3	434	65.8	69.5 (411)
**1i** (6-311+G**)	311	92.0	380	75.3	70.6 (405)
**1a** (NCS)	242	118.1	275	103.8	73.2 (391)
**1a**	240	118.9	251	113.8	73.2 (391)

(6-311+G**)					
**1j**	244	117.4	234	119.8	108.7 (263)
**G**	245	116.9	234	120.5	77.6 (369)
**1b** (NFSI)	250	114.3	309	92.7	61.3 (467)
**1d** (^{t}BuOCl)	333	85.7	399	71.7	44.3 (645)

Supplementary Table 3. Sum of electronic and zero-point energies and sum of electronic and thermal free energies for **Table 1** and **Supplementary Table 2**. The dotted names indicated the corresponding N or O-centered radical, after the dissociation of Cl, F (fluorine) or Me radical. The names followed by T1 indicate the corresponding triplet.

Compound	*E* (hartree) @(U)M06/6-31+G*	*G* (hartree) @(U)M06/6-31+G*
**Cl•**	-460.101463	-460.117140
**F• (fluorine)**	-99.697883	-99.712696
**Me•**	-39.769814	-39.788671
**1e**	-1492.961769	-1493.010220
**1f**	-671.839794	-671.869681
**A**	-747.020051	-747.050608
**B**	-711.100404	-711.131121
**C**	-761.935085	-761.965766
**D**	-781.811830	-781.842476
**E**	-821.070637	-821.105788
**1c**	-1407.385974	-1407.423554
**1g**	-745.877449	-745.908700
**1h**	-1884.369074	-1884.408061
**F**	-859.161877	-859.195459
**1i**	-972.201110	-972.236383
**1a**	-819.910772	-819.943628
**1j**	-399.635741	-399.668256
**G**	-459.514184	-459.546375
**1b**	-1714.167432	-1714.214741
**1d**	-692.894964	-692.926344
**1e•**	-1032.785834	-1032.832950
**1f•**	-211.658348	-211.686571
**A•**	-286.835335	-286.864401
**B•**	-250.913670	-250.942974
**C•**	-301.746849	-301.776915
**D•**	-321.622923	-321.652583
**E•**	-360.880988	-360.916867
**1c•**	-947.191717	-947.227718
**1g•**	-285.680178	-285.709802
**1h•**	-1424.168019	-1424.205338
**F•**	-398.954946	-398.987273

Compound	*E* (hartree) @(U)M06/6-31+G*	*G* (hartree) @(U)M06/6-31+G*
**1i•**	-511.988883	-512.022926
**1a•**	-359.692689	-359.721795
**1j•**	-359.692689	-359.721795
**G•**	-359.692689	-359.721795
**1b•**	-1614.37194	-1614.423234
**1d•**	-232.722861	-232.752400
**1e T1**	-1492.824945	-1492.870152
**1f T1**	-671.706453	-671.737440
**A T1**	-746.893952	-746.925333
**B T1**	-710.972566	-711.004147
**C T1**	-761.788680	-761.819569
**D T1**	-781.663518	-781.694319
**E T1**	-820.920221	-820.955509
**1c T1**	-1407.253336	-1407.291339
**1g T1**	-745.727570	-745.759060
**1h T1**	-1884.215338	-1884.252810
**F T1**	-858.981172	-859.014512
**1i T1**	-972.082171	-972.118569
**1a T1**	-819.740058	-819.771719
**1j T1**	-399.444768	-399.475964
**G T1**	-459.322187	-459.353286
**1b T1**	-1714.019772	-1714.065317
**1d T1**	-692.780750	-692.813273
**Cl•** (6-311+G**)	-460.126597	-460.142274
**1i** (6-311+G**)	-972.333588	-972.368880
**1a** (6-311+G**)	-820.017612	-820.050573
**1i•** (6-311+G**)	-512.094446	-512.128250
**1a•** (6-311+G**)	-359.774394	-359.806270

For such small molecules with only N–Cl bonds, carbonyl groups, and phenyl groups, the S_0–S_1 transition is limited to wavelengths below 320 nm, as expected, and the spin-restricted S_0–T_1 transition was assigned to UV-A (320–400 nm) for **1e**, **1f**, **1h** and **1i** (*N*–chlorophthalimide, NCP), or visible light (> 400 nm) for **1c**. In addition, compounds **A**, **B**, and **C** can absorb UV-A if the spin-restricted S_0–T_1 transitions occur. The transitions of compounds **D**, **E**, **F**, and **G** are limited to wavelengths below 300 nm. NFSI **1b** with an N–F bond cannot absorb visible light or UV-A.

Supplementary Table 4. TD-DFT vertical one-electron excitations for **Table 1**. Calculated at M06/6-31+G* levels of theory.

Compound	Excited state	Energy (eV)	Wavelength (nm)	Oscillator strength (*f*)	Transition
**1e**	1	4.2229	293.60	0.0037	HOMO → LUMO (0.69393)
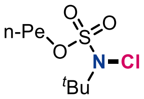	2	5.7502	215.62	0.0070	HOMO-1 → LUMO (0.64522)
	3	6.2009	199.95	0.0020	HOMO-2 → LUMO (0.60684)
	4	6.2537	198.26	0.0057	HOMO → LUMO+1 (0.64982)
	5	6.5089	190.49	0.0037	HOMO-3 → LUMO (0.50437)
**1f**	1	4.2204	293.77	0.0162	HOMO → LUMO (0.69178)
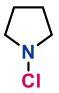	2	5.5570	223.11	0.0033	HOMO → LUMO+1 (0.68518)
	3	5.8267	212.79	0.0009	HOMO-1 → LUMO (0.68996)
	4	5.8900	210.50	0.0007	HOMO → LUMO+2 (0.70185)
	5	6.1105	202.90	0.0090	HOMO → LUMO+3 (0.62654)
**A**	1	3.8914	318.61	0.0150	HOMO → LUMO (0.68525)
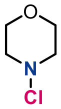	2	5.2105	237.95	0.0012	HOMO → LUMO+1 (0.69270)
	3	5.4540	227.33	0.0172	HOMO → LUMO+2 (0.70448)
	4	5.4791	226.29	0.0029	HOMO → LUMO+3 (0.69307)
	5	5.8181	213.10	0.0103	HOMO → LUMO+4 (0.69162)
**B**	1	3.9879	310.90	0.0148	HOMO → LUMO (0.66274)
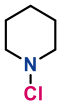	2	5.1849	239.13	0.0014	HOMO → LUMO+1 (0.65470)
	3	5.4701	226.66	0.0118	HOMO → LUMO+2 (0.70031)
	4	5.5579	223.08	0.0008	HOMO → LUMO+3 (0.66583)
	5	5.6915	217.84	0.0025	HOMO → LUMO+4 (0.66881)
**C**	1	4.5374	273.25	0.0072	HOMO → LUMO (0.61861)
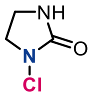	2	5.5883	221.86	0.0102	HOMO-1 → LUMO (0.60794)
	3	5.7181	216.83	0.0119	HOMO → LUMO+1 (0.54644)
	4	5.8488	211.98	0.0018	HOMO-2 → LUMO (0.54290)
	5	6.0465	205.05	0.0058	HOMO → LUMO+2 (0.68614)
**D**	1	4.6034	269.33	0.0058	HOMO → LUMO (0.69342)
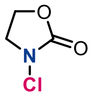	2	5.936	208.87	0.0044	HOMO-1 → LUMO (0.66225)
	3	6.0978	203.33	0.0061	HOMO → LUMO+1 (0.68513)
	4	6.2167	199.44	0.0032	HOMO-3 → LUMO (0.57758)
	5	6.4687	191.67	0.0038	HOMO → LUMO+3 (0.64608)
**E**	1	4.5811	270.64	0.0009	HOMO-1 → LUMO (0.51153)
					HOMO → LUMO (0.42286)
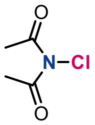	2	4.7454	261.27	0.0205	HOMO → LUMO (0.50214)
	3	5.3676	230.98	0.0030	HOMO-2 → LUMO (0.57067)
	4	5.5423	223.7	0.0000	HOMO-1 → LUMO+2 (0.45909)

					HOMO-1 → LUMO+1 (-0.35975)
	5	5.8284	212.73	0.1083	HOMO → LUMO+1 (0.41464)
**1c**	1	4.4691	277.42	0.0168	HOMO → LUMO (0.55438)
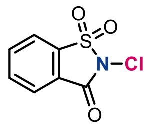	2	4.6372	267.37	0.0061	HOMO-3 → LUMO (0.48356)
	3	4.727	262.29	0.0066	HOMO → LUMO+1 (0.42936)
					HOMO → LUMO (0.34234)
	4	4.8973	253.17	0.0144	HOMO-1 → LUMO (0.56245)
	5	5.3448	231.97	0.0521	HOMO-2 → LUMO (0.58822)
**1g**	1	4.5968	269.72	0.0016	HOMO → LUMO (0.66581)
	2	5.3601	231.31	0.0101	HOMO-1 → LUMO (0.57932)
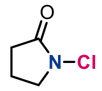	3	5.8507	211.91	0.0183	HOMO → LUMO+1 (0.57425)
	4	5.9553	208.19	0.0108	HOMO-1 → LUMO+4 (0.42713)
					HOMO → LUMO+1 (-0.33895)
	5	6.2455	198.52	0.0036	HOMO → LUMO+2 (0.62224)
**1h**	1	4.3879	282.56	0.0000	HOMO → LUMO (0.64010)
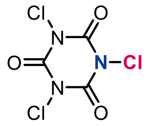	2	4.3882	282.54	0.0000	HOMO-1 → LUMO (0.64010)
	3	4.8499	255.64	0.0000	HOMO-2 → LUMO (0.69698)
	4	4.9111	252.46	0.0001	HOMO-3 → LUMO (0.57502)
	5	5.7221	216.68	0.0002	HOMO-4 → LUMO (0.56674)
**F**	1	4.9664	249.65	0.0008	HOMO-1 → LUMO+1 (0.67744)
	2	5.0240	246.78	0.0000	HOMO → LUMO (0.62396)
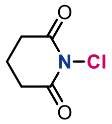	3	5.4558	227.25	0.0002	HOMO-1 → LUMO (0.45193)
					HOMO-2 → LUMO+1 (-0.43796)
	4	5.5154	224.80	0.0000	HOMO-2 → LUMO+1 (0.43608)
					HOMO-1 → LUMO (0.42862)
	5	5.9416	208.67	0.1621	HOMO → LUMO+1 (0.66354)
**1i**	1	3.9569	313.34	0.0001	HOMO → LUMO (0.69924)
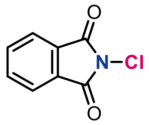	2	3.9801	311.51	0.0001	HOMO-2 → LUMO (0.69233)
	3	4.5458	272.75	0.0426	HOMO-1 → LUMO (0.62691)
	4	4.6479	266.76	0.0000	HOMO-4 → LUMO (0.67244)
	5	5.0067	247.64	0.0000	HOMO → LUMO+1 (0.65723)
**1a**	1	5.1252	241.91	0.0011	HOMO → LUMO+1 (0.68194)
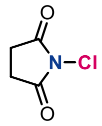	2	5.1685	239.89	0.0002	HOMO-1 → LUMO (0.67879)
	3	5.5125	224.91	0.0022	HOMO → LUMO (0.68562)
	4	5.7248	216.57	0.0000	HOMO-2 → LUMO+1 (0.58317)
	5	6.2391	198.72	0.1800	HOMO-1 → LUMO+1 (0.68103)
**1j**	1	5.0919	243.49	0.0011	HOMO → LUMO+1 (0.68202)
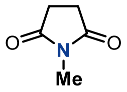	2	5.6343	220.05	0.0000	HOMO-2 → LUMO+1 (0.59459)
	3	6.1370	202.03	0.0020	HOMO → LUMO (0.68369)
	4	6.5878	188.20	0.1048	HOMO → LUMO+2 (0.58959)
	5	6.6378	186.79	0.0000	HOMO → LUMO+3 (0.69930)
**G**	1	5.0714	244.48	0.0012	HOMO → LUMO (0.68667)
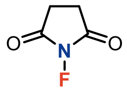	2	5.7163	216.90	0.0000	HOMO-2 → LUMO (0.61502)
	3	6.3916	193.98	0.0040	HOMO → LUMO+2 (0.55826)
	4	6.5339	189.76	0.0403	HOMO → LUMO+1 (0.57579)
	5	6.6995	185.06	0.0000	HOMO-1 → LUMO+2 (0.63919)
**1b**	1	4.9585	250.04	0.0461	HOMO → LUMO (0.60898)

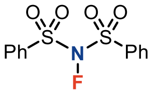	2	4.9749	249.22	0.0003	HOMO-1 → LUMO (0.59641)
	3	5.0654	244.77	0.0462	HOMO-2 → LUMO (0.60635)
	4	5.2792	234.85	0.3014	HOMO-3 → LUMO (0.65744)
	5	5.5874	221.90	0.0394	HOMO-4 → LUMO (0.58254)
**1d**	1	3.7184	333.43	0.0000	HOMO → LUMO (0.69967)
	2	4.9383	251.07	0.0032	HOMO-1 → LUMO (0.69798)
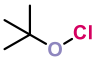	3	6.2939	196.99	0.0001	HOMO → LUMO+1 (0.68925)
	4	6.3363	195.67	0.0001	HOMO-2 → LUMO (0.65982)
	5	6.6808	185.58	0.0192	HOMO → LUMO+2 (0.68380)

3.2. Potential energy surface (Fig. 4)

Supplementary Table 5. Sum of electronic and zero-point energies and imaginary frequencies for **Fig. 4a**. The point numbers indicate the distance from the original N–Cl bond times 0.05 Å. The names preceded by T1 indicate the corresponding triplet.

Point	*ΔE* (kcal mol^{-1})	N–Cl bond length (Å)	*E* (hartree) @M06/6-31+G*
**-10**	213.10	0.90	-819.678017
**-9**	153.38	0.95	-819.773186
**-8**	108.13	1.00	-819.845300
**-7**	74.14	1.05	-819.899458
**-6**	48.96	1.10	-819.939591
**-5**	30.64	1.15	-819.968787
**-4**	17.87	1.20	-819.989132
**-3**	9.17	1.25	-820.003004
**-2**	3.77	1.30	-820.011609
**-1**	0.95	1.35	-820.016098
**0**	0.00	1.40	-820.017612
**+1**	0.54	1.45	-820.016759
**+2**	2.23	1.50	-820.014058
**+3**	4.76	1.55	-820.010027
**+4**	7.90	1.60	-820.005019
**+5**	11.51	1.65	-819.999265
**+6**	15.41	1.70	-819.993061
**+7**	19.45	1.75	-819.986619
**+8**	23.54	1.80	-819.980093
**+9**	27.72	1.85	-819.973443
**+10**	31.89	1.90	-819.966786

Point	*ΔE* (kcal mol^{-1})	N–Cl bond length (Å)	*E* (hartree) @UM06/6-31+G*
**T1 -10**	189.69	1.19	-819.533937
**T1 -9**	135.26	1.24	-819.620673
**T1 -8**	94.77	1.29	-819.685204
**T1 -7**	64.94	1.34	-819.732737
**T1 -6**	43.13	1.39	-819.767493
**T1 -5**	27.66	1.44	-819.792152
**T1 -4**	17.55	1.49	-819.808251
**T1 -3**	11.17	1.54	-819.818426
**T1 -2**	7.05	1.59	-819.824986
**T1 -1**	6.87	1.64	-819.825278
**T1 0**	0.00	1.69	-819.836225
**T1 +1**	-14.96	1.74	-819.860064
**T1 +2**	-23.14	1.79	-819.873093
**T1 +3**	-29.42	1.84	-819.883101
**T1 +4**	-33.69	1.89	-819.889914
**T1 +5**	-38.15	1.94	-819.897023
**T1 +6**	-40.93	1.99	-819.901454
**T1 +7**	-42.59	2.04	-819.904104
**T1 +8**	-44.42	2.09	-819.907018
**T1 +9**	-45.25	2.14	-819.908336
**T1 +10**	-45.72	2.19	-819.909079

Supplementary Table 6. TD-DFT vertical one-electron excitations for **Fig. 4a**. Calculated at M06/6-311+G** levels of theory. Respective HOMO/LUMO are shown below. 0.08 was used for the isovalue.

N–Cl compound	*ΔE* (kcal mol^{-1})	Energy (eV)	Wavelength (nm)	Oscillator strength (*f*)	Transition
**-10**	200.52	4.6107	268.91	0.0062	HOMO → LUMO (0.68398)
**-9**	146.11	4.8410	256.11	0.0060	HOMO → LUMO (0.68441)
**-8**	105.91	5.0605	245.01	0.0057	HOMO → LUMO (0.68372)
**-7**	73.31	5.1204	242.14	0.0012	HOMO-1 → LUMO+1 (0.67643)
**-6**	48.79	5.1491	240.79	0.0013	HOMO-1 → LUMO+1 (0.67574)
**-5**	30.86	5.1658	240.01	0.0013	HOMO-1 → LUMO+1 (0.67596)
**-4**	18.26	5.1732	239.67	0.0013	HOMO-1 → LUMO+1 (0.67672)
**-3**	9.57	5.1739	239.63	0.0013	HOMO-1 → LUMO+1 (0.67739)
**-2**	4.09	5.1706	239.79	0.0013	HOMO-1 → LUMO+1 (0.67911)
**-1**	1.15	5.1652	240.04	0.0013	HOMO-1 → LUMO+1 (0.68037)
**0**	0.00	5.1563	240.45	0.0013	HOMO → LUMO+1 (0.68130)

**+1**	-7.76	4.7967	258.48	0.0001	HOMO-1 → LUMO (0.69154)
**+2**	-15.79	4.3750	283.39	0.0001	HOMO-1 → LUMO (0.70017)
**+3**	-21.95	3.9982	310.10	0.0001	HOMO-1 → LUMO (0.70273)
**+4**	-26.16	3.6793	336.98	0.0001	HOMO-1 → LUMO (0.70383)
**+5**	-30.63	3.3289	372.45	0.0001	HOMO-1 → LUMO (0.70443)
**+6**	-33.45	3.0378	408.14	0.0000	HOMO-1 → LUMO (0.70485)
**+7**	-35.23	2.7851	445.17	0.0000	HOMO-1 → LUMO (0.70486)
**+8**	-37.32	2.5171	492.56	0.0000	HOMO-1 → LUMO (0.70595)
**+9**	-38.50	2.2846	542.69	0.0087	HOMO → LUMO (0.70513)
**+10**	-39.15	2.0753	597.43	0.0088	HOMO → LUMO (0.70466)

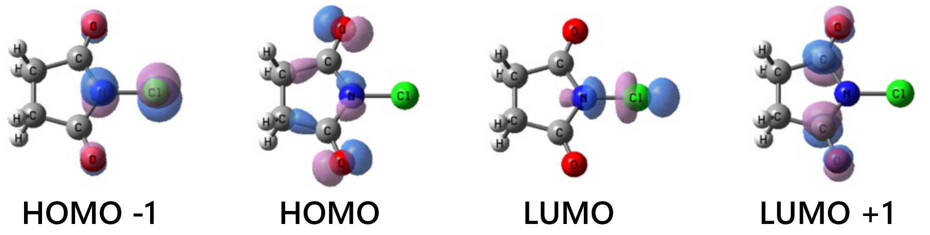

Supplementary Table 7. Sum of electronic and zero-point energies and imaginary frequencies for **Fig. 4b**. The point numbers indicate the distance from the original N–F bond times 0.05 Å. The names preceded by T1 indicate the corresponding triplet.

Point	*ΔE* (kcal mol^{-1})	N–F bond length (Å)	*E* (hartree) @M06/6-31+G*
**-10**	273.73	0.90	-1713.968170
**-9**	191.32	0.95	-1714.099502
**-8**	130.64	1.00	-1714.196194
**-7**	86.24	1.05	-1714.266957
**-6**	54.23	1.10	-1714.317976
**-5**	35.76	1.15	-1714.347405
**-4**	20.29	1.20	-1714.372053
**-3**	10.26	1.25	-1714.388038
**-2**	4.11	1.30	-1714.397838
**-1**	0.95	1.35	-1714.402876
**0**	0.00	1.40	-1714.404389
**+1**	0.57	1.45	-1714.403477
**+2**	2.28	1.50	-1714.400752
**+3**	4.76	1.55	-1714.396801
**+4**	7.74	1.60	-1714.392047
**+5**	11.03	1.65	-1714.386805
**+6**	14.49	1.70	-1714.381293
**+7**	18.01	1.75	-1714.375694
**+8**	21.46	1.80	-1714.370184
**+9**	25.30	1.85	-1714.364078
**+10**	28.92	1.90	-1714.358303

Point	*ΔE* (kcal mol^{-1})	N–F bond length (Å)	*E* (hartree) @UM06/6-31+G*
**T1 -10**	243.48	0.90	-1713.868130
**T1 -9**	169.06	0.95	-1713.986720
**T1 -8**	117.24	1.00	-1714.069296
**T1 -7**	84.17	1.05	-1714.122003
**T1 -6**	48.81	1.10	-1714.178354
**T1 -5**	35.08	1.15	-1714.200228
**T1 -4**	20.19	1.20	-1714.223952
**T1 -3**	10.45	1.25	-1714.239476
**T1 -2**	4.39	1.30	-1714.249142
**T1 -1**	1.15	1.35	-1714.254300
**T1 0**	0.00	1.40	-1714.256132
**T1 +1**	0.20	1.45	-1714.255817
**T1 +2**	7.32	1.50	-1714.244462
**T1 +3**	-8.26	1.55	-1714.269294
**T1 +4**	-14.64	1.60	-1714.279459
**T1 +5**	-19.76	1.65	-1714.287616
**T1 +6**	-23.61	1.70	-1714.293764
**T1 +7**	-26.40	1.75	-1714.298210
**T1 +8**	-28.46	1.80	-1714.301490
**T1 +9**	-29.56	1.85	-1714.303231
**T1 +10**	-30.30	1.90	-1714.304422

Supplementary Table 8. TD-DFT vertical one-electron excitations for **Fig. 4b.** Calculated at M06/6-311+G** levels of theory. Respective HOMO/LUMO are shown below. 0.05 was used for the isovalue.

N–F compound	*ΔE* (kcal mol^{-1})	Energy (eV)	Wavelength (nm)	Oscillator strength (*f*)	Transition
**-10**	244.53	3.7641	329.39	0.0213	HOMO → LUMO (0.44795)
**-9**	168.30	4.0322	307.49	0.0374	HOMO → LUMO (0.64287)
**-8**	110.28	4.1471	298.97	0.0349	HOMO → LUMO (0.66839)
**-7**	68.91	4.2786	289.78	0.0506	HOMO → LUMO (0.68204)
**-6**	40.74	4.4453	278.91	0.0654	HOMO → LUMO (0.68555)
**-5**	24.12	4.5255	273.97	0.0183	HOMO → LUMO (0.68669)
**-4**	12.61	4.6969	263.97	0.0189	HOMO → LUMO (0.68490)
**-3**	6.37	4.8616	255.03	0.0243	HOMO → LUMO (0.67612)
**-2**	3.40	4.9996	247.99	0.0301	HOMO-1 → LUMO (0.59673)
**-1**	1.25	5.0431	245.85	0.0130	HOMO → LUMO+1 (0.55402)
**0**	0.00	5.0302	246.48	0.0143	HOMO → LUMO+1 (0.57343)
**+1**	-0.58	4.9804	248.95	0.0019	HOMO → LUMO+1 (0.32349)<br>HOMO-4 → LUMO (0.27011)<br>HOMO-4 → LUMO+1 (0.25907)
**+2**	-6.94	4.6303	267.77	0.0015	HOMO-4 → LUMO (0.51524)
**+3**	-14.00	4.2166	294.04	0.0011	HOMO-4 → LUMO (0.56515)
**+4**	-20.32	3.8131	325.16	0.0010	HOMO-4 → LUMO (0.59030)
**+5**	-25.60	3.4415	360.26	0.0009	HOMO-4 → LUMO (0.59853)
**+6**	-29.73	3.1126	398.33	0.0009	HOMO-4 → LUMO (0.59937)
**+7**	-32.87	2.8238	439.06	0.0011	HOMO-4 → LUMO (0.59622)
**+8**	-35.33	2.5673	482.94	0.0013	HOMO-4 → LUMO (0.58927)
**+9**	-36.54	2.3487	527.89	0.0017	HOMO-4 → LUMO (0.58018)
**+10**	-37.28	2.1593	574.19	0.0023	HOMO-4 → LUMO (0.56933)

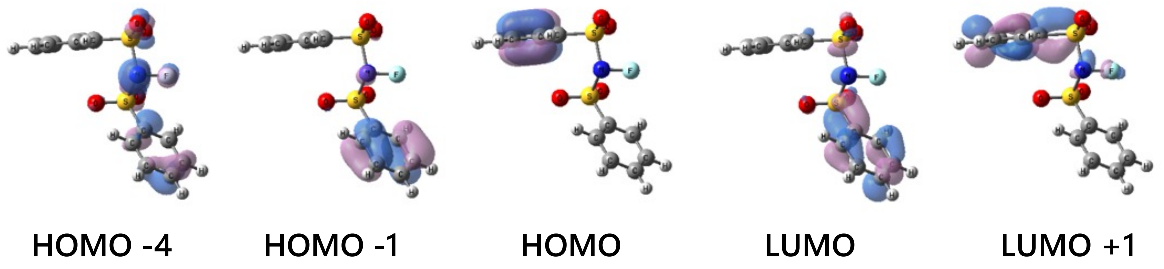

In **Fig. 4b**, at approximately 1.5 Å of the N–F bond the T_1 energy increases. To better understand this specific energy point within the analyzed surface we recalculated the surrounding points with a finer scan (0.025 Å intervals instead of the initial 0.050 Å).

Supplementary Table 9. Sum of electronic and zero-point energies and imaginary frequencies for the finer rescan (0.025 Å) of a section of **Fig. 4b** where the T_1 energy arises. The point numbers indicate the distance from the original N–F bond times 0.025 Å. The names preceded by T1 indicate the corresponding triplet.

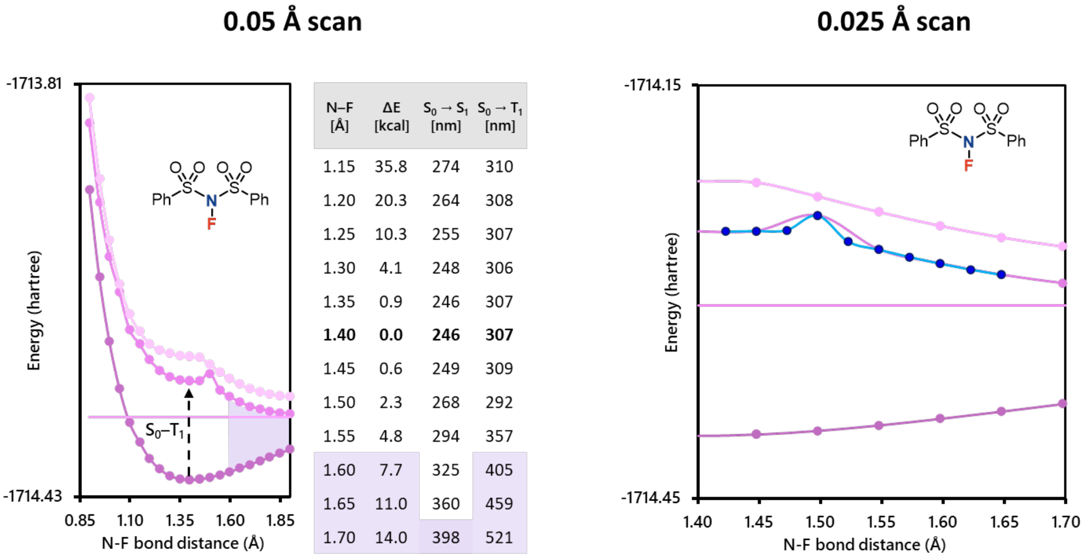

Point	ΔE (kcal mol^{-1})	N–F bond length (Å)	*E* (hartree) @UM06/6-31+G*
**T1 0**	0.00	1.40	-1714.256132
**T1 +1**	-0.03	1.42	-1714.256177
**T1 +2**	0.20	1.45	-1714.255806
**T1 +3**	0.61	1.47	-1714.255164
**T1 +4**	7.32	1.50	-1714.24447
**T1 +5**	-4.58	1.52	-1714.263434
**T1 +6**	-8.26	1.55	-1714.269302
**T1 +7**	-11.62	1.57	-1714.274655
**T1 +8**	-14.65	1.60	-1714.279477
**T1 +9**	-17.35	1.62	-1714.283782
**T1 +10**	-19.73	1.65	-1714.287579

From the results, we can infer that this energy point probably represents an outlier on the analyzed energy surface. There are several factors that could influence this outcome, such as changes in the initial structure, level of theory, or basis set. In addition to the possibility of an outlier, there might be a very small activation barrier for the N–F bond cleavage in the T_1 state. In any case, this potential energy surface clearly supports our conclusion that the present reactions proceed via a VMP pathway.

Supplementary Table 10. Sum of electronic and zero-point energies and imaginary frequencies for **Fig. 4c**. The point numbers indicate the distance from the original O–Cl bond times 0.05Å. The names preceded by T1 indicate the corresponding triplet.

Point	ΔE (kcal mol^{-1})	O–Cl bond length (Å)	E (hartree) @M06/6-31+G*
**-10**	172.89	1.21	-692.701623
**-9**	123.58	1.26	-692.780197
**-8**	86.52	1.31	-692.839253
**-7**	58.93	1.36	-692.883222
**-6**	38.65	1.41	-692.915540
**-5**	24.13	1.46	-692.938684
**-4**	13.99	1.51	-692.954834
**-3**	7.16	1.56	-692.965725
**-2**	2.89	1.61	-692.972526
**-1**	0.70	1.66	-692.976018
**0**	0.00	1.71	-692.977136
**+1**	0.40	1.76	-692.976505
**+2**	1.61	1.81	-692.974566
**+3**	3.42	1.86	-692.971683
**+4**	5.65	1.91	-692.968128
**+5**	8.14	1.96	-692.964157
**+6**	10.81	2.01	-692.959916
**+7**	13.52	2.06	-692.955594
**+8**	16.23	2.11	-692.951274
**+9**	19.02	2.16	-692.946825
**+10**	21.75	2.21	-692.942469

Point	ΔE (kcal mol^{-1})	O–Cl bond length (Å)	E (hartree) @UM06/6-31+G*
**T1 -10**	213.95	1.21	-692.522347
**T1 -9**	168.66	1.26	-692.594526
**T1 -8**	133.58	1.31	-692.650424
**T1 -7**	105.74	1.36	-692.694794
**T1 -6**	83.01	1.41	-692.731022
**T1 -5**	63.83	1.46	-692.761586
**T1 -4**	47.29	1.51	-692.787940
**T1 -3**	32.85	1.56	-692.810944
**T1 -2**	20.17	1.61	-692.831155
**T1 -1**	9.25	1.66	-692.848555
**T1 0**	0.00	1.71	-692.863299
**T1 +1**	-7.77	1.76	-692.875686
**T1 +2**	-14.21	1.81	-692.885941
**T1 +3**	-19.44	1.86	-692.894280
**T1 +4**	-23.54	1.91	-692.900820
**T1 +5**	-26.69	1.96	-692.905825
**T1 +6**	-29.00	2.01	-692.909510
**T1 +7**	-30.61	2.06	-692.912072
**T1 +8**	-31.63	2.11	-692.913698
**T1 +9**	-32.16	2.16	-692.914542
**T1 +10**	-32.29	2.21	-692.914750

Supplementary Table 11. TD-DFT vertical one-electron excitations for **Fig. 4c.** Calculated at M06/6-311+G** levels of theory. Respective HOMO/LUMO are shown below. 0.04 was used for the isovalue.

O–Cl compound	ΔE (kcal mol^{-1})	Energy (eV)	Wavelength (nm)	Oscillator strength (f)	Transition
**-10**	175.46	3.8238	324.24	0.0023	HOMO → LUMO (0.69156)
**-9**	133.28	4.1328	300.00	0.0019	HOMO → LUMO (0.69102)
**-8**	102.92	4.4233	280.30	0.0014	HOMO → LUMO (0.69043)
**-7**	81.47	4.6898	264.37	0.0010	HOMO → LUMO (0.68831)
**-6**	66.58	4.9233	251.83	0.0005	HOMO → LUMO (0.67667)
**-5**	55.48	5.0718	244.46	0.0000	HOMO → LUMO (0.56733)
**-4**	43.37	4.9860	248.66	0.0000	HOMO → LUMO+1 (0.45696)
**-3**	30.81	4.7378	261.69	0.0000	HOMO → LUMO+1 (0.52036)
**-2**	19.13	4.4166	280.73	0.0000	HOMO → LUMO (0.63830)
**-1**	8.89	4.0674	304.82	0.0000	HOMO → LUMO (0.69044)
**0**	0.00	3.7124	333.98	0.0000	HOMO → LUMO (0.69908)
**+1**	-7.67	3.3626	368.72	0.0000	HOMO → LUMO (0.70225)
**+2**	-14.10	3.0308	409.08	0.0000	HOMO → LUMO (0.70340)
**+3**	-19.48	2.7190	455.99	0.0000	HOMO → LUMO (0.70388)

**+4**	-23.80	2.4352	509.13	0.0000	HOMO → LUMO (0.70378)
**+5**	-27.25	2.1774	569.40	0.0000	HOMO → LUMO (0.70348)
**+6**	-29.91	1.9467	636.88	0.0000	HOMO → LUMO (0.70280)
**+7**	-31.89	1.7433	711.21	0.0000	HOMO → LUMO (0.70156)
**+8**	-33.28	1.5655	791.98	0.0000	HOMO → LUMO (0.69946)
**+9**	-34.04	1.4112	878.55	0.0000	HOMO → LUMO (0.69607)
**+10**	-34.35	1.2795	969.04	0.0001	HOMO → LUMO (0.69076)

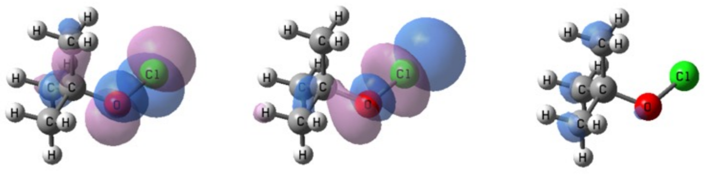

HOMO LUMO LUMO +1

Supplementary Table 12. Sum of electronic and zero-point energies and imaginary frequencies for **Fig. 4d**. The point numbers indicate the distance from the original N–C bond times 0.05Å. The names preceded by T1 indicate the corresponding triplet.

Point	*ΔE* (kcal mol^{-1})	N–C bond length (Å)	*E* (hartree) @M06/6-31+G*
**-10**	268.25	0.95	-399.300169
**-9**	191.77	1.00	-399.422045
**-8**	134.34	1.05	-399.513562
**-7**	91.54	1.10	-399.581761
**-6**	60.08	1.15	-399.631909
**-5**	37.37	1.20	-399.668096
**-4**	21.44	1.25	-399.693486
**-3**	11.01	1.30	-399.710098
**-2**	4.43	1.35	-399.720588
**-1**	1.11	1.40	-399.725882
**0**	0.00	1.45	-399.727645
**+1**	0.60	1.50	-399.726691
**+2**	2.49	1.55	-399.723676
**+3**	5.32	1.60	-399.719169
**+4**	8.83	1.65	-399.713574
**+5**	12.82	1.70	-399.707214
**+6**	17.14	1.75	-399.700334
**+7**	21.65	1.80	-399.693140
**+8**	26.23	1.85	-399.685842
**+9**	30.85	1.90	-399.678484
**+10**	35.54	1.95	-399.671007

Point	*ΔE* (kcal mol^{-1})	N–C bond length (Å)	*E* (hartree) @UM06/6-31+G*
**T1 -10**	237.49	0.95	-399.155937
**T1 -9**	170.04	1.00	-399.263421
**T1 -8**	117.35	1.05	-399.347398
**T1 -7**	77.79	1.10	-399.410435
**T1 -6**	49.18	1.15	-399.456033
**T1 -5**	30.77	1.20	-399.485358
**T1 -4**	11.66	1.25	-399.515812
**T1 -3**	8.20	1.30	-399.521340
**T1 -2**	2.95	1.35	-399.529698
**T1 -1**	0.52	1.40	-399.533580
**T1 0**	0.00	1.45	-399.534401
**T1 +1**	1.01	1.50	-399.532790
**T1 +2**	3.15	1.55	-399.529379
**T1 +3**	6.04	1.60	-399.524776
**T1 +4**	9.49	1.65	-399.519274
**T1 +5**	13.33	1.70	-399.513165
**T1 +6**	17.39	1.75	-399.506683
**T1 +7**	21.52	1.80	-399.500106
**T1 +8**	26.03	1.85	-399.492925
**T1 +9**	28.66	1.90	-399.488726
**T1 +10**	24.66	1.95	-399.495109

Supplementary Table 13. TD-DFT vertical one-electron excitations for **Fig. 4d.** Calculated at M06/6-311+G** levels of theory. Respective HOMO/LUMO are shown below. 0.04 was used for the isovalue.

N–C compound	*ΔE* (kcal mol^{-1})	Energy (eV)	Wavelength (nm)	Oscillator strength (*f*)	Transition
**-10**	263.33	4.9066	252.69	0.0091	HOMO → LUMO (0.69172)
**-9**	192.08	5.1333	241.53	0.0019	HOMO-1 → LUMO+1 (0.64564)
**-8**	135.55	5.1720	239.72	0.0017	HOMO-1 → LUMO+1 (0.65091)
**-7**	93.14	5.1890	238.94	0.0016	HOMO-1 → LUMO+1 (0.65987)
**-6**	61.72	5.1908	238.85	0.0015	HOMO-1 → LUMO+1 (0.66545)
**-5**	39.01	5.1847	239.14	0.0015	HOMO-1 → LUMO+1 (0.66975)
**-4**	22.93	5.1741	239.63	0.0014	HOMO → LUMO+1 (0.67300)
**-3**	12.26	5.1611	240.23	0.0014	HOMO → LUMO+1 (0.67553)
**-2**	5.05	5.1468	240.90	0.0013	HOMO → LUMO+1 (0.67754)
**-1**	1.39	5.1321	241.59	0.0013	HOMO → LUMO+1 (0.67919)
**0**	0.00	5.1197	242.17	0.0013	HOMO → LUMO+1 (0.68046)
**+1**	0.36	5.1092	242.67	0.0013	HOMO → LUMO+1 (0.68155)
**+2**	1.96	5.0969	243.26	0.0013	HOMO → LUMO+1 (0.68256)
**+3**	4.52	5.0853	243.81	0.0013	HOMO → LUMO+2 (0.68338)
**+4**	7.80	5.0750	244.30	0.0012	HOMO → LUMO+2 (0.68410)
**+5**	11.58	5.0658	244.75	0.0012	HOMO → LUMO+2 (0.68472)
**+6**	15.70	5.0573	245.16	0.0012	HOMO → LUMO+2 (0.68526)
**+7**	20.03	5.0495	245.54	0.0012	HOMO → LUMO+2 (0.68574)
**+8**	24.45	5.0424	245.88	0.0012	HOMO → LUMO+2 (0.68615)
**+9**	27.33	4.9670	249.61	0.0026	HOMO → LUMO (0.68962)
**+10**	23.97	4.6180	268.48	0.0028	HOMO → LUMO (0.69660)

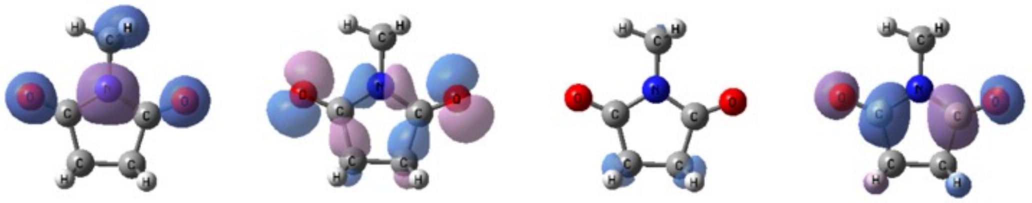

HOMO -1 **HOMO** **LUMO** **LUMO +1**

Supplementary Table 14. Potential energy surface, transition values, sum of electronic and zero-point energies and imaginary frequencies for the calculated transitions from unstable conformations in the N–Cl, axis of NCP (**1i**) at the (U)M06/6-311+G** level; complementary to **Fig. 4**. Δ*E* in kcal mol^{-1}. The point numbers indicate the distance from the original N–Cl bond times 0.05 Å. The names preceded by T_1 indicate the corresponding triplet.

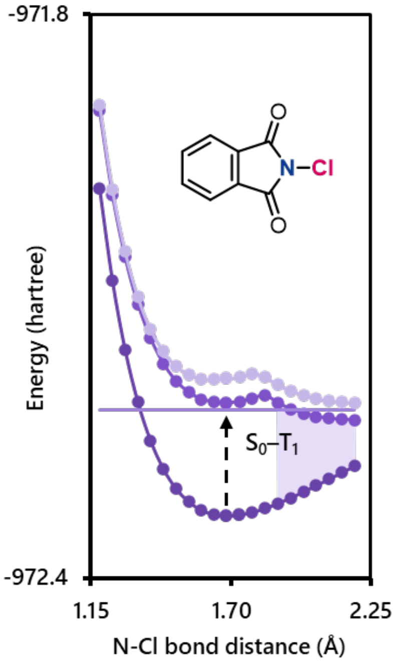

N–Cl [Å]	ΔE [kcal]	$S_0 \rightarrow S_1$ [nm]	$S_0 \rightarrow T_1$ [nm]
1.43	31.6	365	407
1.48	18.3	349	399
1.53	9.5	337	392
1.58	3.9	326	386
1.63	0.9	317	381
**1.68**	**0.0**	**311**	**380**
1.73	0.6	310	377
1.78	2.4	310	376
1.83	5.0	326	379
1.88	8.3	360	426
1.93	12.0	398	481
1.98	16.1	442	545

Point	*ΔE* (kcal mol^{-1})	N–Cl bond length (Å)	*E* (hartree) @M06/6-31+G*
**-10**	218.68	1.18	-971.985093
**-9**	157.50	1.23	-972.082596
**-8**	111.11	1.28	-972.156523
**-7**	76.25	1.33	-972.212077
**-6**	50.41	1.38	-972.253250
**-5**	31.61	1.43	-972.283221
**-4**	18.30	1.48	-972.304425
**-3**	9.48	1.53	-972.318488
**-2**	3.87	1.58	-972.327415
**-1**	0.87	1.63	-972.332204
**0**	0.00	1.68	-972.333588
**+1**	0.62	1.73	-972.332605
**+2**	2.35	1.78	-972.329839
**+3**	5.00	1.83	-972.325623
**+4**	8.28	1.88	-972.320396
**+5**	12.01	1.93	-972.314448
**+6**	16.06	1.98	-972.308000
**+7**	20.27	2.03	-972.301292
**+8**	24.51	2.08	-972.294522
**+9**	28.83	2.13	-972.287639
**+10**	33.14	2.18	-972.280780

Point	*ΔE* (kcal mol^{-1})	N–Cl bond length (Å)	*E* (hartree) @UM06/6-31+G*
**T1 -10**	195.20	1.18	-971.902488
**T1 -9**	139.39	1.23	-971.991422
**T1 -8**	97.54	1.28	-972.058111
**T1 -7**	66.27	1.33	-972.107951
**T1 -6**	43.24	1.38	-972.144642
**T1 -5**	26.47	1.43	-972.171376
**T1 -4**	14.69	1.48	-972.190146
**T1 -3**	7.16	1.53	-972.202148
**T1 -2**	2.58	1.58	-972.209446
**T1 -1**	0.53	1.63	-972.212707
**T1 0**	0.00	1.68	-972.213555
**T1 +1**	1.07	1.73	-972.211857
**T1 +2**	2.98	1.78	-972.208807
**T1 +3**	5.08	1.83	-972.205458
**T1 +4**	0.02	1.88	-972.213521
**T1 +5**	-3.93	1.93	-972.219811
**T1 +6**	-6.85	1.98	-972.224467
**T1 +7**	-8.92	2.03	-972.227768
**T1 +8**	-10.31	2.08	-972.229989
**T1 +9**	-10.87	2.13	-972.230871
**T1 +10**	-11.39	2.18	-972.231713

Supplementary Table 15. TD-DFT vertical one-electron excitations for **Supplementary Table 14**. Calculated at M06/6-311+G** levels of theory. Respective HOMO/LUMO are shown below. 0.04 was used for the isovalue.

N–Cl compound	*ΔE* (kcal mol^{-1})	Energy (eV)	Wavelength (nm)	Oscillator strength (*f*)	Transition
**-10**	182.27	2.4097	514.51	0.0102	HOMO → LUMO (0.70284)
**-9**	126.21	2.6321	471.06	0.0097	HOMO → LUMO (0.70213)
**-8**	84.69	2.8433	436.06	0.0089	HOMO → LUMO (0.70144)
**-7**	54.35	3.0394	407.92	0.0080	HOMO → LUMO (0.70080)
**-6**	32.78	3.2243	384.53	0.0068	HOMO → LUMO (0.70021)
**-5**	17.93	3.3957	365.12	0.0054	HOMO → LUMO (0.69971)
**-4**	8.15	3.5487	349.38	0.0040	HOMO → LUMO (0.69935)
**-3**	2.45	3.6844	336.52	0.0027	HOMO → LUMO (0.69915)
**-2**	-0.43	3.8024	326.07	0.0016	HOMO → LUMO (0.69914)
**-1**	-0.88	3.9889	310.82	0.0003	HOMO → LUMO (0.69916)
**0**	0.00	3.9131	316.84	0.0008	HOMO → LUMO (0.69914)
**+1**	0.74	3.9942	310.41	0.0001	HOMO-1 → LUMO (0.69255)
**+2**	2.66	4.002	309.78	0.0001	HOMO-1→ LUMO (0.69228)
**+3**	0.73	3.8037	325.95	0.0000	HOMO → LUMO+1 (0.63939)
**+4**	-4.29	3.4439	360.01	0.0000	HOMO → LUMO (0.62841)
**+5**	-8.22	3.1118	398.43	0.0000	HOMO → LUMO (0.61810)
**+6**	-11.20	2.8071	441.68	0.0000	HOMO → LUMO (0.60942)
**+7**	-13.42	2.5280	490.44	0.0000	HOMO → LUMO (0.60228)
**+8**	-15.05	2.2733	545.40	0.0000	HOMO → LUMO (0.59698)
**+9**	-15.85	2.0514	604.40	0.0000	HOMO → LUMO (0.59171)
**+10**	-16.57	1.8334	676.26	0.0000	HOMO → LUMO (0.58941)

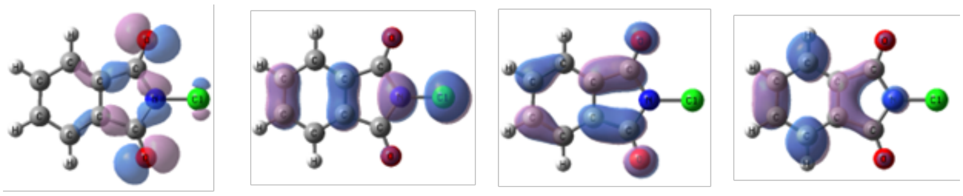

HOMO -1 HOMO LUMO LUMO +1

3.3. Vibrational analysis

Since the photoexcitation of NCS is known to lead to N–Cl bond homolysis, in this work we focused on analyzing the energy surfaces (ground and excited) on the electronegative interelement bond axis, as shown. In the case of NCS, when considering the N–Cl vibrational mode, for example, excitation energies followed by their respective Boltzmann factors (at room temperature and 60ºC) for each vibrational state were calculated as follows.

The N–Cl vibrational mode frequency was estimated to be 648.1086 cm^{-1} (@M06/6-311+G**), which is equivalent to 1.853 kcal mol^{-1}. By assuming that the other states of this mode are harmonic, the corresponding energies will be equivalent to multiples of 1.853. Moreover, by approximating each of the obtained energy surfaces (S_0, S_1, and T_1) to a polynomial trendline (order = 3), it is possible to calculate the S_0–S_1 or S_0–T_1 transitions for each state of the N–Cl vibrational mode as shown below.

Supplementary Table 16. Approximated excitation energy (values in blue represent transitions in the visible range.) and Boltzmann factors (at 27 ºC and 60ºC, values in yellow and red respectively) corresponding to each vibrational state of the N–Cl vibrational mode for NCS (at M06/6-311+G**).

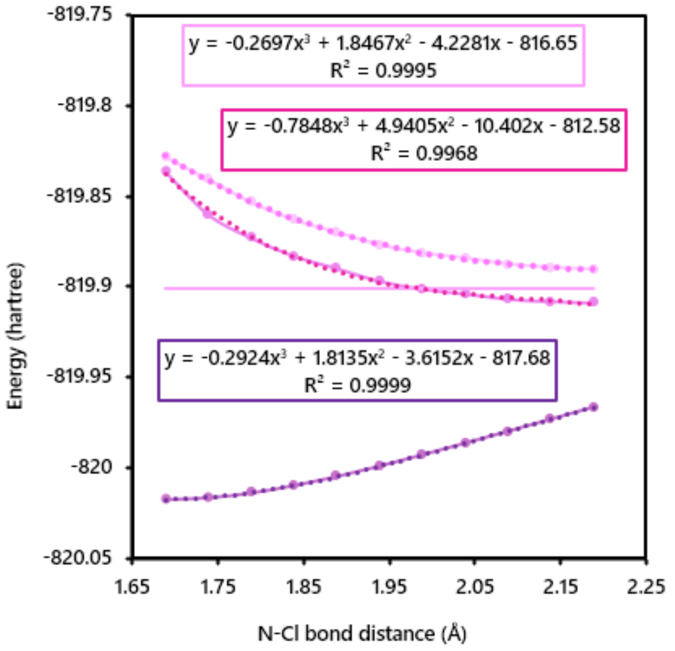

Vib.	Δ*E* (kcal mol^{-1})	y (S_0)	x	y1 (S_1)	y2 (T_1)	S_0–S_1 (nm)	S_0–T_1 (nm)	27 ºC	60 ºC
**µ0**	0.000	-820.02	1.69	-819.83	-819.84	240.5	251.2	1.000	1.000
**µ10-1**	1.853	-820.01	1.83	-819.86	-819.88	286.5	337.9	0.045	0.066
**µ10-2**	3.706	-820.01	1.86	-819.86	-819.89	302.6	362.1	0.002	0.004
**µ10-3**	5.559	-820.01	1.89	-819.87	-819.89	317.8	384.5	9.0E-05	2.9E-04
**µ10-4**	7.412	-820.01	1.92	-819.87	-819.89	332.7	406.0	4.0E-06	1.9E-05
**µ10-5**	9.265	-820.00	1.94	-819.87	-819.90	347.4	427.0	1.8E-07	1.3E-06
**µ10-6**	11.118	-820.00	1.97	-819.87	-819.90	362.2	447.9	8.0E-09	8.3E-08
**µ10-7**	12.971	-820.00	1.99	-819.88	-819.90	377.3	468.9	3.6E-10	5.5E-09
**µ10-8**	14.824	-819.99	2.01	-819.88	-819.90	392.6	490.4	1.6E-11	3.6E-10

**µ10-9**	16.677	-819.99	2.03	-819.88	-819.90	408.2	512.6	7.2E-13	2.4E-11
**µ10-10**	18.530	-819.99	2.06	-819.88	-819.90	424.4	535.9	3.2E-14	1.6E-12

Furthermore, when considering the excitations achievable from each of the other vibrational modes, the transition energies and Boltzmann factors can be also calculated as shown below.

Supplementary Table 17. Approximated excitation energy (values in blue represent transitions in the visible range.) and Boltzmann factors (at 27 ºC and 60 ºC, values in yellow and red respectively) corresponding to each vibrational mode of NCS (at M06/6-311+G**).

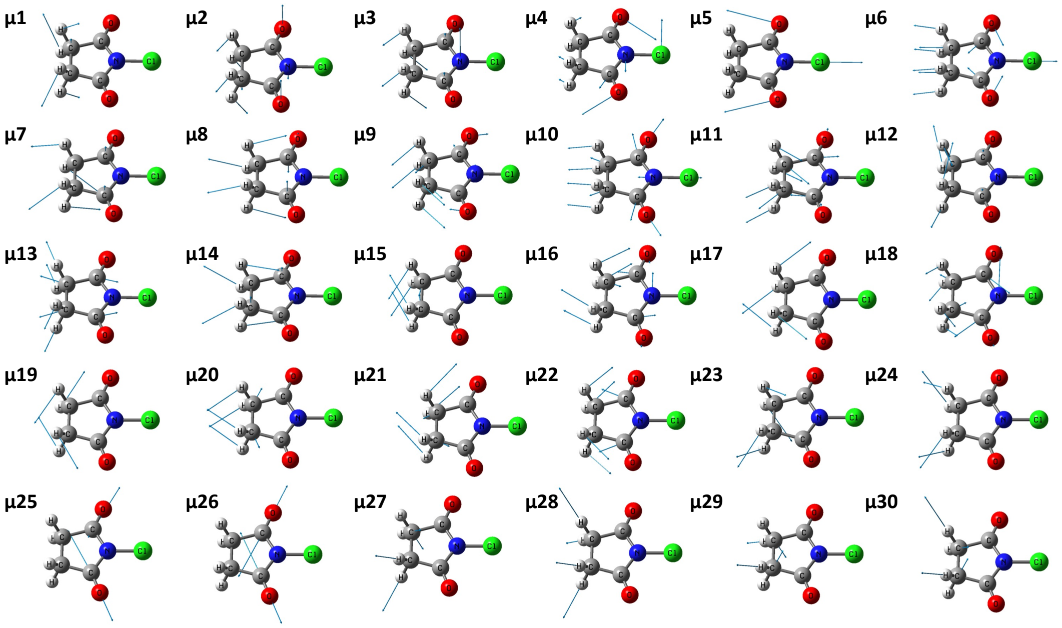

Vib.	Freq. (cm^{-1})	Δ*E* (kcal mol^{-1})	y (S_0)	x	y1 (S_1)	y2 (T_1)	S_0–S_1 (nm)	S_0–T_1 (nm)	27 ºC	60 ºC
**µ0**	0.0000	0.000000	-820.02	1.69	-819.83	-819.84	240.5	251.2	1.000	1.000
**µ1**	53.0118	0.151568	-820.02	1.79	-819.85	-819.87	270.2	312.6	0.776	0.801
**µ2**	99.1276	0.283418	-820.02	1.80	-819.85	-819.87	271.6	314.8	0.622	0.660
**µ3**	184.1427	0.526488	-820.02	1.80	-819.85	-819.87	274.0	318.6	0.414	0.462
**µ4**	212.0462	0.606267	-820.02	1.80	-819.85	-819.87	274.8	319.8	0.362	0.411
**µ5**	334.7798	0.957178	-820.02	1.81	-819.85	-819.88	278.2	325.1	0.201	0.246
**µ6**	528.8199	1.511964	-820.02	1.82	-819.85	-819.88	283.4	333.1	0.079	0.109
**µ7**	556.9409	1.592365	-820.02	1.82	-819.85	-819.88	284.2	334.2	0.069	0.097
**µ8**	561.856	1.606418	-820.02	1.82	-819.85	-819.88	284.3	334.4	0.068	0.095
**µ9**	567.757	1.623290	-820.02	1.82	-819.85	-819.88	284.4	334.7	0.066	0.093
**µ10**	648.1086	1.853025	-820.01	1.83	-819.86	-819.88	286.5	337.9	0.045	0.066
**µ11**	650.1681	1.858914	-820.01	1.83	-819.86	-819.88	286.6	337.9	0.044	0.065

**µ12**	811.1031	2.319048	-820.01	1.84	-819.86	-819.88	290.7	344.2	0.020	0.033
**µ13**	978.0482	2.796365	-820.01	1.85	-819.86	-819.88	294.8	350.5	0.009	0.017
**µ14**	1002.593	2.866542	-820.01	1.85	-819.86	-819.88	295.4	351.4	8.18E-03	1.49E-02
**µ15**	1025.843	2.933016	-820.01	1.85	-819.86	-819.88	296.0	352.3	7.32E-03	1.36E-02
**µ16**	1039.077	2.970853	-820.01	1.85	-819.86	-819.88	296.3	352.7	6.87E-03	1.28E-02
**µ17**	1155.975	3.305079	-820.01	1.85	-819.86	-819.88	299.2	357.0	3.92E-03	7.85E-03
**µ18**	1176.205	3.362921	-820.01	1.86	-819.86	-819.88	299.7	357.8	3.56E-03	7.21E-03
**µ19**	1231.062	3.519763	-820.01	1.86	-819.86	-819.89	301.0	359.7	2.74E-03	5.73E-03
**µ20**	1264.366	3.614985	-820.01	1.86	-819.86	-819.89	301.8	360.9	2.33E-03	4.99E-03
**µ21**	1303.739	3.727558	-820.01	1.86	-819.86	-819.89	302.7	362.3	1.93E-03	4.23E-03
**µ22**	1322.266	3.780528	-820.01	1.86	-819.86	-819.89	303.2	363.0	1.77E-03	3.91E-03
**µ23**	1421.498	4.064245	-820.01	1.87	-819.86	-819.89	305.6	366.5	1.10E-03	2.58E-03
**µ24**	1442.201	4.123436	-820.01	1.87	-819.86	-819.89	306.0	367.3	9.95E-04	2.37E-03
**µ25**	1861.778	5.323062	-820.01	1.89	-819.86	-819.89	315.9	381.7	1.33E-04	4.07E-04
**µ26**	1914.105	5.472672	-820.01	1.89	-819.87	-819.89	317.1	383.5	1.04E-04	3.27E-04
**µ27**	3057.875	8.742855	-820.00	1.94	-819.87	-819.90	343.3	421.1	4.31E-07	2.70E-06
**µ28**	3062.473	8.756002	-820.00	1.94	-819.87	-819.90	343.4	421.3	4.21E-07	2.65E-06
**µ29**	3100.642	8.865133	-820.00	1.94	-819.87	-819.90	344.2	422.5	3.51E-07	2.26E-06
**µ30**	3113.558	8.902059	-820.00	1.94	-819.87	-819.90	344.5	422.9	3.30E-07	2.14E-06

4. Spectroscopical details

Sample solutions were prepared with spectroscopy grade solvents (LC/MS) inside a glove box under an Ar atmosphere or under air and subsequently bubbled with inert gas (Ar or N_2) for more than 10 minutes. Samples were sealed with Teflon tape after being transferred to the measuring cells. All measurements, except absorption with a 100 mm cell, were conducted on a 10 mm screw cap quartz cuvette. Solutions with a concentration greater than 10^{-2} M were prepared by directly weighting the substrate (±0.1 mg precision) and adding the appropriate amount of solvent to obtain a 3 mL solution. Samples with a concentration smaller than 10^{-2} M were prepared by diluting a previously prepared 3 mL 10^{-2} M solution (as described above) with a 10 mL volumetric flask (linearly, from 10-fold for 10^{-3} M, by using 1 mL concentrated solution, to 100-fold for 10^{-4} M, by using 100 µL concentrated solution). All spectra were corrected against a blank solution measurement obtained in the same settings. UV-vis absorption spectra were measured with 1 nm steps at a 1000 nm/min rate.

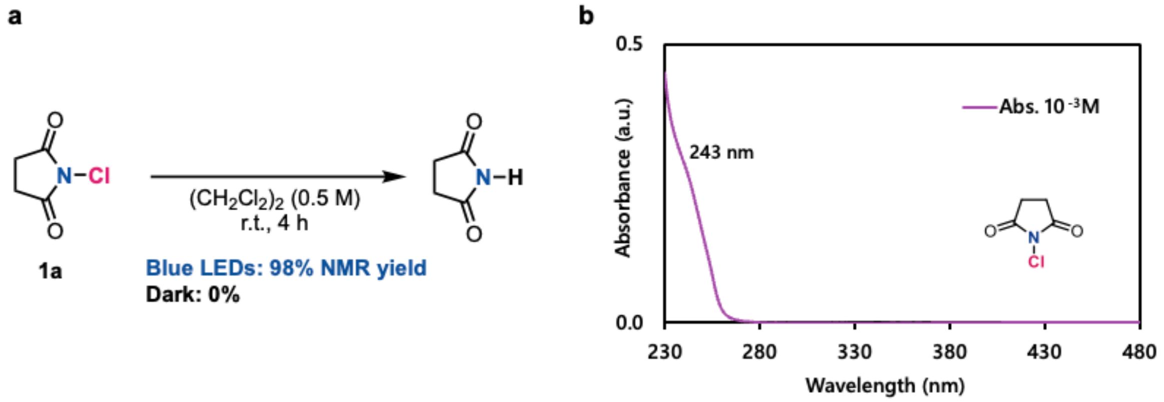

Supplementary Figure 1. **a,** Photolysis of **1a** under blue LED irradiation. **b,** Absorption spectra of **1a** in MeCN.

Supplementary Figure 1 shows the unexpected blue-light-induced photolysis of **1a** against its absorption spectrum, which only features absorption peaks below 320 nm under standard measurement conditions.

5. Supplementary References

(1) Ju, J.; Jeong, M.; Moon, J.; Jung, H. M.; Lee, S. Aminocarbonylation of Aryl Halides Using a Nickel Phosphite Catalytic System. *Org. Lett.* **2007**, *9*, 4615–4618.

(2) Hanada, S.; Tsutsumi, E.; Motoyama, Y.; Nagashima, H. Practical Access to Amines by Platinum-Catalyzed Reduction of Carboxamides with Hydrosilanes: Synergy of Dual Si−H Groups Leads to High Efficiency and Selectivity. *J. Am. Chem. Soc.* **2009**, *131*, 15032–15040.

(3) Tan, B.; Toda, N.; Barbas, C. F. Organocatalytic Amidation and Esterification of Aldehydes with Activating Reagents by a Cross-Coupling Strategy. *Angew. Chem., Int. Ed.* **2012**, *51*, 12538–12541.

(4) Chen, W.; Li, K.; Hu, Z.; Wang, L.; Lai, G.; Li, Z. Utility of Dysprosium as a Reductant in Coupling Reactions of Acyl Chlorides: The Synthesis of Amides and Diaryl-Substituted Acetylenes. *Organometallics* **2011**, *30*, 2026–2030.

(5) Jiang, H.; Liu, B.; Li, Y.; Wang, A.; Huang, H. Synthesis of Amides via Palladium-Catalyzed Amidation of Aryl Halides. *Org. Lett.* **2011**, *13*, 1028–1031.

(6) Akiyama, R.; Yokota, N.; Nishino, E.; Asazawa, K.; Miyatake, K. Anion Conductive Aromatic Copolymers from Dimethylaminomethylated Monomers: Synthesis, Properties, and Applications in Alkaline Fuel Cells. *Macromolecules* **2016**, *49*, 4480–4489.

(7) Liu, W.; Ackermann, L. Versatile Ruthenium(II)-Catalyzed C–H Cyanations of Benzamides. *Chem. Comm.* **2014**, *50*, 1878.

(8) Baum, J. C.; Milne, J. E.; Murry, J. A.; Thiel, O. R. An Efficient and Scalable Ritter Reaction for the Synthesis of *tert*-Butyl Amides. *J. Org. Chem.* **2009**, *74*, 2207–2209.

(9) Khalafi-Nezhad, A.; Foroughi, H. ollah; Doroodmand, M. M.; Panahi, F. Silica Boron–Sulfuric Acid Nanoparticles (SBSANs): Preparation, Characterization and Their Catalytic Application in the Ritter Reaction for the Synthesis of Amide Derivatives. *J. Mater. Chem.* **2011**, *21*, 12842.

(10) Kalkhambkar, R. G.; Waters, S. N.; Laali, K. K. Highly Efficient Synthesis of Amides via Ritter Chemistry with Ionic Liquids. *Tetrahedron Lett.* **2011**, *52*, 867–871.

(11) Chen, C.-T.; Kuo, J.-H.; Pawar, V. D.; Munot, Y. S.; Weng, S.-S.; Ku, C.-H.; Liu, C.-Y. Nucleophilic Acyl Substitutions of Anhydrides with Protic Nucleophiles Catalyzed by Amphoteric, Oxomolybdenum Species. *J. Org. Chem.* **2005**, *70*, 1188–1197.

(12) Syrgiannis, Z.; Gebhardt, B.; Dotzer, C.; Hauke, F.; Graupner, R.; Hirsch, A. Reductive Retrofunctionalization of Single-Walled Carbon Nanotubes. *Angew. Chem., Int. Ed.* **2010**, *49*, 3322–3325.

(13) Chen, C.-T.; Kuo, J.-H.; Pawar, V. D.; Munot, Y. S.; Weng, S.-S.; Ku, C.-H.; Liu, C.-Y. Nucleophilic Acyl Substitutions of Anhydrides with Protic Nucleophiles Catalyzed by Amphoteric, Oxomolybdenum Species. *J. Org. Chem.* **2005**, *70*, 1188–1197.

(14) Li, J.; Cai, S.; Chen, J.; Zhao, Y.; Wang, D. Visible Light Induced Photocatalytic Conversion of Enamines into Amides. *Synlett.* **2014**, *25*, 1626–1628.

(15) Karplus, M. Contact Electron-Spin Coupling of Nuclear Magnetic Moments. *J. Chem. Phys.* **1959**, *30*, 11–15.

(16) Song, L.; Luo, S.; Cheng, J.-P. Visible-Light Promoted Intermolecular Halofunctionalization of Alkenes with N-Halogen Saccharins. *Org. Chem. Front.* **2016**, *3*, 447–452.

(17) Beaulieu, F.; Beauregard, L.-P.; Courchesne, G.; Couturier, M.; LaFlamme, F.; L'Heureux, A. Aminodifluorosulfinium Tetrafluoroborate Salts as Stable and Crystalline Deoxofluorinating Reagents. *Org. Lett.* **2009**, *11*, 5050–5053.

(18) Liu, Y.; Zhou, C.; Jiang, M.; Arndtsen, B. A. Versatile Palladium-Catalyzed Approach to Acyl Fluorides and Carbonylations by Combining Visible Light- and Ligand-Driven Operations. *J.*

Am. Chem. Soc. **2022**, *144*, 9413–9420.

(19) Rauser, M.; Ascheberg, C.; Niggemann, M. Electrophilic Amination with Nitroarenes. *Angew. Chem., Int. Ed.* **2017**, *56*, 11570–11574.

(20) Denton, R. M.; An, J.; Adeniran, B.; Blake, A. J.; Lewis, W.; Poulton, A. M. Catalytic Phosphorus(V)-Mediated Nucleophilic Substitution Reactions: Development of a Catalytic Appel Reaction. *J. Org. Chem.* **2011**, *76*, 6749–6767.

(21) Mustafin, A. G.; Khalilov, I. N.; Tal'vinskii, E. v.; Abdrakhmanov, I. B.; Spirikhin, L. v.; Tolstikov, G. A. Intramolecular Cyclization of Ortho-(Cyclohex-2-Enyl) Anilines Synthesis of Ellipticine. *Chem. Nat. Compd.* **1992**, *28*, 479–483.

(22) C.01 Gaussian 16 Revision; CT Gaussian Inc. Wallingford; Frisch, M. J.; Trucks, G. W.; Schlegel, H. B.; Scuseria, G. E.; Robb, M. A.; Cheeseman, J. R.; Scalmani, G.; Barone, V.; Petersson, G. A.; Nakatsuji, H.; Li, X.; Caricato, M.; Marenich, A. v.; Bloino, J.; Janesko, B. G.; Gomperts, R.; Mennucci, B.; Hratchian, H. P.; Ortiz, J. v.; Izmaylov, A. F.; Sonnenberg, J. L.; Williams-Young, D.; Ding, F.; Lipparini, F.; Egidi, F.; Goings, J.; Peng, B.; Petrone, A.; Henderson, T.; Ranasinghe, D.; Zakrzewski, V. G.; Gao, J.; Rega, N.; Zheng, G.; Liang, W.; Hada, M.; Ehara, M.; Toyota, K.; Fukuda, R.; Hasegawa, J.; Ishida, M.; Nakajima, T.; Honda, Y.; Kitao, O.; Nakai, H.; Vreven, T.; Throssell, K.; Montgomery, J. A., Jr.; Peralta, J. E.; Ogliaro, F.; Bearpark, M. J.; Heyd, J. J.; Brothers, E. N.; Kudin, K. N.; Staroverov, V. N.; Keith, T. A.; Kobayashi, R.; Normand, J.; Raghavachari, K.; Rendell, A. P.; Burant, J. C.; Iyengar, S. S.; Tomasi, J.; Cossi, M.; Millam, J. M.; Klene, M.; Adamo, C.; Cammi, R.; Ochterski, J. W.; Martin, R. L.; Morokuma, K.; Farkas, O.; Foresman, J. B.; Fox, D. J. Gaussian 16, Revision C.01, Gaussian, Inc., Wallingford CT. CT Gaussian Inc. Wallingford 2016.

(23) Becke, A. D. Density-Functional Exchange-Energy Approximation with Correct Asymptotic Behavior. *Phys. Rev. A. (Coll Park)* **1988**, *38*, 3098–3100.

(24) Lee, C.; Yang, W.; Parr, R. G. Development of the Colle-Salvetti Correlation-Energy Formula into a Functional of the Electron Density. *Phys. Rev. B.* **1988**, *37*, 785–789.

(25) Becke, A. D. A New Mixing of Hartree–Fock and Local Density-functional Theories. *J. Chem. Phys.* **1993**, *98*, 1372–1377.

(26) Becke, A. D. Density-functional Thermochemistry. III. The Role of Exact Exchange. *J. Chem. Phys.* **1993**, *98*, 5648–5652.
