# Supplementary material for: Vibration-mediated long-wavelength photolysis of electronegative bonds beyond S0–S1 and S0–T1 transitions
Source: Commun Chem. 2024 Jun 4;7:126. doi: 10.1038/s42004-024-01208-0 (PMC11150518; doi:10.1038/s42004-024-01208-0)
Supplement: Supplementary file 2 — Description of Additional Supplementary Files [file 42004_2024_1208_MOESM2_ESM.docx]

**Description of Additional Supplementary Files**

**File name:** Supplementary Data 1

**Description:** Cartesian coordinates of the computed structures

**File name:** Supplementary Data 2

**Description:** NMR spectra
