# Supplementary material for: Vibration-mediated long-wavelength photolysis of electronegative bonds beyond S0–S1 and S0–T1 transitions
Source: Commun Chem. 2024 Jun 4;7:126. doi: 10.1038/s42004-024-01208-0 (PMC11150518; doi:10.1038/s42004-024-01208-0)
Supplement: Supplementary file 3 — Supplementary Data 1 [file 42004_2024_1208_MOESM3_ESM.docx]

***Supplementary data 1***

**Vibration-mediated long-wavelength photolysis of electronegative interelement bonds beyond S_0_–S_1_ and S_0_–T_1_ transitions**

**Antônio Junio Araujo Dias,^1^ Atsuya Muranaka,^2^ Masanobu Uchiyama,^3^ Ken Tanaka,^1,^* and Yuki Nagashima^1,^***

^1^ Department of Chemical Science and Engineering, Tokyo Institute of Technology, O-okayama, Meguro-ku, Tokyo 152-8550, Japan.

^2^ Molecular Structure Characterization Unit, RIKEN Center for Sustainable Resource Science, 2-1 Hirosawa, Wako, Saitama 351-0198, Japan

^3^ Graduate School of Pharmaceutical Sciences, The University of Tokyo, 7-3-1 Hongo, Bunkyo-ku, Tokyo 113-0033, Japan.

*e-mail: nagashima.y.ae@m.titech.ac.jp; tanaka.k.cg@m.titech.ac.jp

**Table of Contents**

1. Cartesian coordinates of the computed structures S2

**Cl•**

Cl 0.000000 0.000000 0.000000

**F• (fluorine)**

F 0.000000 0.000000 0.000000

**Me•**

C 0.000000 0.000000 -0.000016

H 0.000000 1.084200 0.000032

H 0.938945 -0.542100 0.000032

H -0.938945 -0.542100 0.000032

**1e**

N 1.622475 0.370217 -0.368359

Cl 0.710587 1.656127 0.413550

C 3.129004 0.531867 -0.188612

C 3.465757 1.960211 -0.609991

H 3.127435 2.706743 0.116335

H 4.556411 2.047601 -0.692319

H 3.026861 2.193584 -1.588529

C 3.581774 0.268872 1.240378

H 3.445628 -0.781592 1.526626

H 4.650401 0.506613 1.330580

H 3.032259 0.892865 1.956975

C 3.817888 -0.414337 -1.172612

H 3.407668 -0.290011 -2.182331

H 4.885107 -0.157443 -1.195719

H 3.724939 -1.465342 -0.890587

S 0.901293 -1.121295 0.120354

O 1.588153 -2.150031 -0.622572

O 0.740400 -1.228634 1.557681

O -0.527491 -0.904397 -0.553463

C -1.709419 -0.812415 0.283254

H -1.972116 -1.825530 0.616921

H -1.483009 -0.203905 1.167604

C -2.793181 -0.193047 -0.561743

H -2.938522 -0.798686 -1.469355

H -2.457513 0.801306 -0.896146

C -4.100488 -0.078435 0.209530

H -4.419213 -1.077557 0.553617

H -3.942560 0.519846 1.123512

C -5.216283 0.548128 -0.614635

H -4.895920 1.544943 -0.958376

H -5.372085 -0.050442 -1.526678

C -6.519049 0.661017 0.159927

H -7.315543 1.113873 -0.443484

H -6.871158 -0.327338 0.487618

H -6.391591 1.278835 1.060090

**1f**

C 1.389957 -0.771560 -0.585175

C 0.493099 -1.146291 0.602879

C 0.493059 1.146154 0.603154

C 1.389658 0.771803 -0.585177

H 2.395945 -1.194135 -0.475112

H 0.974775 -1.163245 -1.521816

H 1.084810 -1.260327 1.524378

H -0.096564 -2.058381 0.470917

H -0.096598 2.058307 0.471591

H 1.084926 1.259850 1.524598

H 0.973857 1.163283 -1.521638

H 2.395506 1.194829 -0.475608

N -0.391916 -0.000111 0.871675

Cl -1.680464 -0.000002 -0.371360

**A**

C -1.142377 1.168114 -0.417789

C -0.161630 1.202963 0.738844

C -0.161605 -1.202956 0.738866

C -1.142328 -1.168131 -0.417788

H 0.492465 2.082059 0.697029

H -0.606206 1.213927 -1.383034

H -1.830418 2.020246 -0.368702

H 0.492514 -2.082032 0.697074

H -0.718234 -1.237298 1.688464

H -0.606125 -1.213918 -1.383020

H -1.830335 -2.020290 -0.368735

H -0.718254 1.237327 1.688444

O -1.937917 -0.000026 -0.362023

N 0.676643 0.000014 0.845110

Cl 1.867004 0.000009 -0.478818

**B**

C -1.978553 -0.000006 -0.398905

C -1.108182 1.252898 -0.396017

C -0.118341 1.212888 0.760663

C -0.118343 -1.212900 0.760671

C -1.108165 -1.252896 -0.396025

H 0.555999 2.077780 0.759421

H -0.557081 1.329169 -1.346207

H -1.723667 2.160297 -0.314309

H -2.619021 -0.000010 0.500815

H -2.656560 -0.000015 -1.263596

H 0.555999 -2.077788 0.759433

H -0.662864 -1.227504 1.720489

H -0.557017 -1.329109 -1.346192

H -1.723634 -2.160311 -0.314395

H -0.662853 1.227512 1.720488

N 0.706572 0.000001 0.837184

Cl 1.864364 0.000004 -0.512856

**C**

C 1.891274 -0.872928 0.097421

C 0.479793 -1.414706 -0.115415

H 2.214369 -1.027525 1.138771

H 2.620926 -1.335265 -0.576030

H 0.255680 -2.300453 0.487335

H 0.288460 -1.635961 -1.178665

N -0.304499 -0.267839 0.325870

Cl -1.975129 -0.292593 -0.048045

C 0.391738 0.926558 0.030047

O -0.064467 2.043605 0.009765

N 1.693024 0.534387 -0.204836

H 2.416993 1.225078 -0.052316

**D**

C 1.884158 -0.816828 0.084755

C 0.501854 -1.408507 -0.149838

H 2.226939 -0.984374 1.113691

H 2.643235 -1.166091 -0.619488

H 0.303991 -2.299940 0.454000

H 0.308684 -1.633491 -1.211377

N -0.290424 -0.276282 0.300365

Cl -1.965571 -0.296680 -0.026919

C 0.397863 0.916439 0.032619

O -0.047737 2.023368 -0.038777

O 1.705435 0.590982 -0.109595

**E**

N -0.000122 -0.210515 -0.000331

Cl -0.001029 -1.927286 -0.000255

C 1.446812 1.822172 -0.334407

H 0.831816 2.113293 -1.191447

H 1.205845 2.476944 0.509542

H 2.501368 1.969218 -0.581903

C -1.444675 1.823839 0.334024

H -0.829278 2.114580 1.190901

H -1.203000 2.477946 -0.510236

H -2.499030 1.972262 0.581567

C 1.302569 0.365617 0.021010

C -1.302218 0.366974 -0.020787

O 2.264820 -0.323543 0.252522

O -2.265359 -0.321254 -0.251375

**1c**

C 2.192000 1.543538 -0.010667

C 0.995852 0.840498 -0.019616

C 1.008726 -0.549494 -0.002563

C 2.177246 -1.289071 0.013045

C 3.376427 -0.576284 0.023097

C 3.382736 0.820105 0.013047

H 2.177466 2.631649 -0.024372

H 2.160045 -2.377177 0.019544

H 4.319601 -1.118615 0.039788

H 4.333064 1.349987 0.021587

C -0.358068 1.449814 -0.049148

S -0.637802 -1.205716 0.007172

O -0.912920 -1.990822 -1.177246

O -1.022748 -1.691098 1.317172

O -0.635707 2.620718 0.026727

N -1.277298 0.406802 -0.210251

Cl -2.936633 0.647822 0.009651

**1g**

C -1.892981 -0.929529 0.159004

C -0.478385 -1.431575 -0.142604

C -1.828004 0.571815 -0.125857

H -2.647026 -1.452256 -0.438755

H -2.123449 -1.105043 1.217279

H -0.345717 -1.723943 -1.197422

H -0.165409 -2.268208 0.492471

H -2.449677 1.186034 0.533674

H -2.114769 0.811903 -1.159802

N 0.310980 -0.245954 0.159728

Cl 2.003883 -0.304088 -0.010214

C -0.366861 0.953288 0.024090

O 0.125070 2.057336 0.015038

**1h**

C -1.360006 0.523205 -0.000272

C 1.133512 0.916174 0.000161

C 0.227030 -1.439843 0.000073

N -1.044782 -0.844816 -0.000055

N 1.254230 -0.482688 0.000039

N -0.208979 1.326915 -0.000165

O 0.412721 -2.621605 0.000223

O 2.063774 1.668685 0.000685

O -2.476383 0.953002 -0.000450

Cl 2.840228 -1.092683 0.000088

Cl -0.473762 3.005469 -0.000241

Cl -2.366902 -1.912418 0.000026

**F**

C 1.650306 1.236452 0.179036

C 2.271570 0.000054 -0.441469

C 1.650337 -1.236505 0.178738

H 2.008787 -2.169733 -0.269601

H 2.107069 0.000193 -1.529072

H 3.357380 0.000040 -0.286726

H 1.894507 1.288657 1.252686

H 2.008932 2.169815 -0.268878

H 1.894788 -1.289153 1.252310

C 0.145388 1.271099 0.073625

C 0.145385 -1.271081 0.073742

N -0.477893 0.000002 0.019056

O -0.497281 2.290254 0.054520

O -0.497282 -2.290227 0.054643

Cl -2.185154 -0.000010 -0.090556

**1i**

C 1.996187 1.421582 0.000156

C 0.819168 0.696520 0.000030

C 0.819278 -0.696563 -0.000056

C 1.996351 -1.421545 -0.000052

C 3.192511 -0.698108 0.000055

C 3.192431 0.698251 0.000156

H 1.981567 2.509911 0.000226

H 1.981755 -2.509876 -0.000112

H 4.141675 -1.230844 0.000064

H 4.141532 1.231100 0.000224

C -0.583670 1.185080 0.000026

O -1.011706 2.309458 -0.000531

N -1.354528 -0.000069 0.000103

Cl -3.039442 -0.000001 0.000242

C -0.583522 -1.185172 -0.000090

O -1.011635 -2.309465 -0.000292

**1a**

C 1.876042 0.764172 0.000179

C 1.876065 -0.764143 0.000002

H 2.361951 1.203031 -0.879059

H 2.361131 1.202406 0.880243

H 2.361365 -1.202627 0.879820

H 2.361776 -1.202728 -0.879486

N -0.324702 -0.000000 0.000569

C 0.424401 -1.188211 -0.000019

C 0.424383 1.188220 -0.000073

O -0.033255 -2.299435 -0.000452

O -0.033293 2.299424 -0.000505

Cl -2.014487 -0.000013 0.000095

**1j**

C 0.713260 -1.621211 0.000033

C -0.811793 -1.574290 -0.000042

H 1.140134 -2.118241 0.879124

H 1.140235 -2.118318 -0.878968

H -1.268746 -2.044324 0.878835

H -1.268630 -2.044202 -0.879044

C 1.155478 -0.173910 0.000006

C -1.169978 -0.103713 0.000001

O 2.286757 0.255514 0.000000

O -2.277599 0.382356 0.000032

N 0.017096 0.622769 -0.000010

C 0.078048 2.068414 -0.000016

H 0.611176 2.422135 -0.888916

H -0.947775 2.446650 -0.000329

H 0.610574 2.422228 0.889220

**G**

C -0.767252 1.550319 0.000344

C 0.767236 1.550323 -0.000288

H -1.201218 2.038697 -0.879673

H -1.200619 2.038230 0.880881

H 1.201475 2.039078 0.879336

H 1.200326 2.037857 -0.881220

N 0.000004 -0.613966 0.000238

F 0.000013 -1.959399 -0.000045

C 1.198544 0.099957 0.000019

C -1.198547 0.099950 0.000007

O 2.301076 -0.376539 0.000069

O -2.301076 -0.376560 -0.000203

**1b**

N -0.145263 0.785096 -0.586172

F -0.989586 1.905686 -0.502492

S 1.493698 1.560364 -0.369748

S -0.722240 -0.301600 0.719059

O 0.002476 -1.530973 0.448217

O -0.662601 0.356815 2.010079

O 1.705514 2.165724 -1.668332

O 1.499077 2.338113 0.851223

C -2.407678 -0.456551 0.204512

C -3.384641 0.282734 0.864155

C -2.704301 -1.320670 -0.846234

C -4.706730 0.139762 0.458177

H -3.104837 0.949713 1.677182

C -4.030386 -1.449286 -1.241321

H -1.908549 -1.880940 -1.333526

C -5.025250 -0.721424 -0.589991

H -5.489739 0.704358 0.960167

H -4.288753 -2.120376 -2.057920

H -6.062331 -0.827194 -0.903879

C 2.469772 0.106601 -0.188148

C 2.747048 -0.652692 -1.322438

C 2.889461 -0.262402 1.086735

C 3.465485 -1.830456 -1.164834

H 2.401076 -0.321455 -2.299931

C 3.615377 -1.439348 1.223208

H 2.638274 0.358166 1.945030

C 3.894253 -2.220032 0.103609

H 3.693221 -2.446150 -2.032567

H 3.955909 -1.752269 2.207952

H 4.455329 -3.145773 0.220072

**1d**

C 0.735869 -0.000007 0.036415

C 0.761390 -1.259992 0.882323

H 1.701368 -1.311557 1.447886

H -0.064788 -1.279993 1.603948

H 0.689018 -2.151050 0.245575

C 0.761339 1.259657 0.882800

H 1.701268 1.310991 1.448466

H 0.689036 2.150958 0.246385

H -0.064904 1.279395 1.604359

C 1.874040 0.000205 -0.973801

H 1.827461 -0.890595 -1.612025

H 1.827384 0.891210 -1.611733

H 2.834863 0.000161 -0.442640

O -0.409359 0.000153 -0.856423

Cl -1.921274 0.000004 -0.061486

**1e•**

N 1.543563 0.587622 -0.089054

C 2.959272 0.951729 -0.043567

C 2.985166 2.417073 0.385295

H 2.606046 2.528595 1.409196

H 4.014462 2.798812 0.353992

H 2.363693 3.027135 -0.281908

C 3.820594 0.111352 0.898726

H 3.876453 -0.938590 0.588147

H 4.840714 0.517566 0.896213

H 3.437184 0.150758 1.925596

C 3.472597 0.829482 -1.491573

H 2.853410 1.423233 -2.175307

H 4.500252 1.216898 -1.525476

H 3.471707 -0.213605 -1.826161

S 1.118835 -1.019001 0.086436

O 1.846972 -1.888354 -0.812501

O 1.072332 -1.279303 1.516430

O -0.366649 -0.972273 -0.492610

C -1.364391 -0.262020 0.279021

H -1.429165 -0.721655 1.274594

H -1.039533 0.783154 0.389816

C -2.665956 -0.366389 -0.475096

H -2.913734 -1.429322 -0.618390

H -2.537401 0.065605 -1.479342

C -3.793619 0.341986 0.261991

H -3.907199 -0.089163 1.271595

H -3.530633 1.403171 0.414134

C -5.123222 0.254936 -0.473727

H -5.007777 0.684709 -1.481908

H -5.383967 -0.804984 -0.625134

C -6.245486 0.963905 0.266396

H -7.198674 0.894310 -0.272328

H -6.395691 0.530576 1.265325

H -6.017430 2.030574 0.402403

**1f•**

C -0.727895 -0.973529 0.224410

C -1.154320 0.454975 -0.124091

C 1.153551 0.456651 0.124105

C 0.729349 -0.972631 -0.224505

H -0.791998 -1.132588 1.311404

H -1.342223 -1.741243 -0.261124

H -1.985480 0.849908 0.478529

H -1.497251 0.514904 -1.176177

H 1.984342 0.852813 -0.478278

H 1.496375 0.516647 1.176218

H 0.794049 -1.131830 -1.311370

H 1.344599 -1.739350 0.261519

N -0.000931 1.316849 -0.000033

**A•**

C 1.162120 -0.712809 0.193301

C 1.172670 0.774334 -0.188613

C -1.172670 0.774334 -0.188613

C -1.162120 -0.712809 0.193301

H 2.076109 1.259249 0.204262

H 1.213716 -0.811787 1.292711

H 2.019435 -1.234484 -0.246907

H -2.076109 1.259249 0.204262

H -1.192265 0.835310 -1.293770

H -1.213716 -0.811787 1.292711

H -2.019435 -1.234483 -0.246907

H 1.192265 0.835310 -1.293770

O -0.000000 -1.337698 -0.295676

N 0.000000 1.409530 0.342364

**B•**

C -0.000000 1.407365 0.239876

C 1.248834 0.669761 -0.224569

C 1.183088 -0.806900 0.197285

C -1.183087 -0.806900 0.197285

C -1.248834 0.669761 -0.224569

H 2.070019 -1.349690 -0.154423

H 1.324535 0.718094 -1.322179

H 2.162652 1.125539 0.181765

H -0.000000 1.469265 1.341511

H -0.000000 2.441403 -0.133653

H -2.070019 -1.349690 -0.154423

H -1.175608 -0.852186 1.306286

H -1.324535 0.718094 -1.322179

H -2.162652 1.125539 0.181765

H 1.175608 -0.852185 1.306287

N 0.000000 -1.427529 -0.334659

**C•**

C -1.351105 0.749546 -0.052755

C -1.297435 -0.786552 0.046697

H -1.773892 1.082359 -1.012801

H -1.938350 1.207762 0.754014

H -1.898065 -1.299484 -0.718531

H -1.688403 -1.149762 1.013990

N 0.067073 -1.207563 -0.052349

C 0.873168 -0.044018 -0.001613

O 2.086263 -0.038686 -0.002558

N 0.049961 1.059508 0.054811

H 0.441598 1.991142 0.012591

**D•**

C -1.287981 0.763138 -0.055366

C -1.299787 -0.764443 0.067358

H -1.665584 1.111693 -1.024928

H -1.828481 1.281571 0.742932

H -1.943339 -1.277232 -0.661530

H -1.649322 -1.105826 1.059498

N 0.052141 -1.193876 -0.084724

C 0.850988 -0.036797 -0.010000

O 2.051987 -0.021059 0.015959

O 0.090815 1.093000 0.042185

**E•**

N -0.000018 0.207437 -0.635804

C 2.126462 1.051239 0.164684

H 2.409643 1.493405 -0.798648

H 1.603290 1.827740 0.738758

H 3.025347 0.732289 0.700401

C -2.126620 1.051114 0.164370

H -3.025519 0.732222 0.700096

H -1.603577 1.827825 0.738283

H -2.409740 1.493016 -0.799100

C 1.236851 -0.130626 -0.068517

C -1.236844 -0.130706 -0.068422

O 1.531855 -1.287765 0.125892

O -1.531656 -1.287820 0.126376

**1c•**

C 2.153041 0.941873 0.000350

C 0.783055 0.701360 0.000044

C 0.299981 -0.600750 -0.000375

C 1.131314 -1.706981 -0.000466

C 2.503491 -1.461999 -0.000218

C 3.007347 -0.155589 0.000223

H 2.526628 1.964060 0.000599

H 0.732027 -2.719109 -0.000889

H 3.195545 -2.301924 -0.000357

H 4.084333 -0.000586 0.000484

C -0.294921 1.719548 0.000070

S -1.476639 -0.544324 -0.000031

O -2.007164 -1.018603 -1.265669

O -2.006212 -1.018901 1.266024

O -0.141224 2.923008 -0.000672

N -1.596480 1.151134 0.000774

**1g•**

C 1.392125 0.671719 -0.198969

C 1.262673 -0.828800 0.109664

C 0.004409 1.189171 0.153753

H 2.204098 1.150849 0.358557

H 1.597825 0.813329 -1.268383

H 1.526020 -1.044462 1.165168

H 1.909127 -1.486645 -0.487409

H -0.363149 2.015132 -0.463812

H -0.058629 1.518092 1.202184

N -0.110529 -1.212881 -0.052153

C -0.876314 -0.038915 -0.003704

O -2.092368 -0.054398 -0.063212

**1h•**

C 1.205809 1.253273 0.045651

C -1.205814 1.252575 0.048174

C 0.000087 -0.942002 0.088788

N 1.169392 -0.153468 0.087278

N -1.169494 -0.153671 0.086417

N 0.000184 1.820173 0.459947

O 0.000104 -2.137086 0.070491

O -2.177282 1.901369 -0.228575

O 2.176955 1.901001 -0.233190

Cl -2.644526 -0.979370 -0.071622

Cl 2.644568 -0.979253 -0.069612

**F•**

C 0.000062 1.644955 -0.400200

C -1.254655 1.027651 0.208526

C 1.254679 1.027589 0.208675

H -1.322463 1.280534 1.277863

H -2.178333 1.363408 -0.276537

H 0.000122 1.489013 -1.489136

H 0.000088 2.730334 -0.239246

H 1.322318 1.280387 1.278042

H 2.178437 1.363370 -0.276221

N -0.000020 -1.004247 0.532158

C 1.219099 -0.489969 0.079117

C -1.219120 -0.489917 0.079131

O -2.130018 -1.174927 -0.315668

O 2.129966 -1.174969 -0.315753

**1i•**

C -1.320744 -1.426063 0.010700

C -0.140188 -0.697406 0.016151

C -0.140142 0.697344 0.016482

C -1.320672 1.426032 0.011083

C -2.510348 0.702132 -0.000640

C -2.510403 -0.702128 -0.000782

H -1.307008 -2.514328 0.008960

H -1.306985 2.514302 0.009804

H -3.461910 1.230582 -0.013959

H -3.462013 -1.230487 -0.014014

C 1.275074 -1.147552 0.014108

O 1.708631 -2.263315 -0.148863

N 2.079118 -0.000040 0.272479

C 1.275380 1.147549 0.013977

O 1.708412 2.263410 -0.149214

**1a•**

C -0.765954 1.197066 0.000495

C 0.765950 1.197067 0.000320

H -1.212807 1.667042 -0.883224

H -1.212599 1.667208 0.884210

H 1.212831 1.667256 0.883890

H 1.212568 1.666991 -0.883544

N 0.000001 -0.966507 0.006590

C 1.195714 -0.278033 -0.000127

C -1.195714 -0.278032 0.000276

O 2.326261 -0.683207 -0.003219

O -2.326257 -0.683212 -0.003437

**1j•**

C -0.765954 1.197066 0.000495

C 0.765950 1.197067 0.000320

H -1.212807 1.667042 -0.883224

H -1.212599 1.667208 0.884210

H 1.212831 1.667256 0.883890

H 1.212568 1.666991 -0.883544

N 0.000001 -0.966507 0.006590

C 1.195714 -0.278033 -0.000127

C -1.195714 -0.278032 0.000276

O 2.326261 -0.683207 -0.003219

O -2.326257 -0.683212 -0.003437

**G•**

C -0.765954 1.197066 0.000495

C 0.765950 1.197067 0.000320

H -1.212807 1.667042 -0.883224

H -1.212599 1.667208 0.884210

H 1.212831 1.667256 0.883890

H 1.212568 1.666991 -0.883544

N 0.000001 -0.966507 0.006590

C 1.195714 -0.278033 -0.000127

C -1.195714 -0.278032 0.000276

O 2.326261 -0.683207 -0.003219

O -2.326257 -0.683212 -0.003437

**1b•**

N -0.307913 1.093281 -0.269839

S 1.313157 1.645770 -0.193768

S -0.725605 -0.491431 0.181373

O -0.213187 -1.387386 -0.846566

O -0.353800 -0.656347 1.580576

O 1.510599 2.269113 -1.490242

O 1.324760 2.410605 1.043723

C -2.486240 -0.391355 0.050980

C -3.214396 0.049773 1.152102

C -3.091438 -0.737439 -1.153206

C -4.596867 0.140245 1.038220

H -2.702789 0.307686 2.077384

C -4.475270 -0.641467 -1.250394

H -2.485883 -1.082052 -1.989078

C -5.222527 -0.203263 -0.158949

H -5.187446 0.478753 1.887095

H -4.971109 -0.911397 -2.180615

H -6.305603 -0.130503 -0.241347

C 2.416061 0.268599 -0.050578

C 2.912011 -0.306360 -1.218347

C 2.738390 -0.215233 1.214772

C 3.759395 -1.401571 -1.109190

H 2.633507 0.102358 -2.187414

C 3.589070 -1.310614 1.305317

H 2.324938 0.255970 2.103504

C 4.093581 -1.900877 0.148344

H 4.158898 -1.867134 -2.007789

H 3.855927 -1.705713 2.283320

H 4.756530 -2.760879 0.227772

**1d•**

C 0.000057 -0.028172 0.084414

C 1.267713 -0.789318 -0.301853

H 1.294817 -0.981891 -1.383269

H 2.158980 -0.214686 -0.021028

H 1.303351 -1.755399 0.218019

C -0.002279 1.364708 -0.595193

H -0.001588 1.208471 -1.682398

H -0.896830 1.931011 -0.312762

H 0.889992 1.934291 -0.312141

C -1.265228 -0.793369 -0.301650

H -1.297141 -1.760127 0.217205

H -2.158205 -0.222053 -0.019495

H -1.292552 -0.984872 -1.383249

O -0.000300 0.290271 1.420601

**1a (NCS) (6-311+G**)**

C -1.872042 -0.762715 0.000305

C -1.872042 0.762716 0.000127

H -2.354852 -1.200817 -0.877162

H -2.354200 -1.200500 0.878303

H -2.354492 1.200736 0.877842

H -2.354556 1.200584 -0.877625

N 0.323310 0.000000 0.000249

C -0.423061 1.187958 0.000100

C -0.423063 -1.187958 -0.000137

O 0.030570 2.291699 -0.000298

O 0.030569 -2.291699 -0.000276

Cl 2.012180 -0.000001 -0.000051

**1b (NFSI) (6-311+G**)**

N -0.302142 1.092870 -0.254102

S 1.314068 1.655589 -0.229111

S -0.708969 -0.481470 0.231165

O -0.180665 -1.405630 -0.751547

O -0.359690 -0.597352 1.633473

O 1.486536 2.235990 -1.539746

O 1.356156 2.451778 0.977500

C -2.468845 -0.395215 0.068802

C -3.211208 0.111809 1.125587

C -3.051944 -0.807270 -1.120060

C -4.586569 0.200791 0.982280

H -2.715619 0.421788 2.039785

C -4.429377 -0.712375 -1.246817

H -2.434057 -1.201220 -1.920140

C -5.190794 -0.209691 -0.199776

H -5.189079 0.590452 1.795977

H -4.909302 -1.033458 -2.165273

H -6.268708 -0.137239 -0.305484

C 2.406717 0.271000 -0.068144

C 2.878458 -0.335772 -1.224287

C 2.727790 -0.192097 1.200333

C 3.695611 -1.446653 -1.099912

H 2.601634 0.059667 -2.195720

C 3.546967 -1.304632 1.306178

H 2.332932 0.305666 2.079101

C 4.024586 -1.928568 0.160873

H 4.075520 -1.938739 -1.988900

H 3.810861 -1.686654 2.286575

H 4.662182 -2.802509 0.252128

**1d (*t*-BuOCl) (6-311+G**)**

C 0.736388 0.000011 0.035445

C 0.763369 -1.256927 0.881597

H 1.698516 -1.303351 1.449485

H -0.064049 -1.278059 1.596904

H 0.697956 -2.146166 0.246979

C 0.763522 1.257431 0.880878

H 1.698575 1.303980 1.448912

H 0.698404 2.146324 0.245746

H -0.064019 1.279140 1.596027

C 1.871327 -0.000357 -0.974226

H 1.823029 -0.889430 -1.609167

H 1.823017 0.888256 -1.609811

H 2.829738 -0.000151 -0.445111

O -0.407621 -0.000163 -0.853321

Cl -1.922813 -0.000011 -0.060915

**1j (*N*-methylsuccinimide) (6-311+G**)**

C 0.714183 -1.616482 0.000033

C -0.808077 -1.571736 -0.000037

H 1.141278 -2.109777 0.877207

H 1.141373 -2.109854 -0.877050

H -1.263780 -2.039276 0.876899

H -1.263677 -2.039202 -0.877069

C 1.156199 -0.171404 0.000012

C -1.169880 -0.104220 -0.000020

O 2.279347 0.255765 0.000006

O -2.270630 0.376635 0.000012

N 0.016380 0.620917 -0.000008

C 0.074404 2.065263 -0.000001

H 0.604718 2.421653 -0.886132

H -0.949439 2.440612 -0.000321

H 0.604151 2.421699 0.886453

**1i (NCP) (6-311+G**)**

C 1.993033 1.416728 0.000090

C 0.818817 0.694294 -0.000049

C 0.818818 -0.694294 -0.000085

C 1.993034 -1.416729 -0.000034

C 3.185732 -0.696024 0.000077

C 3.185731 0.696023 0.000141

H 1.977469 2.501829 0.000114

H 1.977471 -2.501830 -0.000058

H 4.132423 -1.226729 0.000129

H 4.132421 1.226729 0.000226

C -0.582941 1.184976 -0.000224

O -1.007899 2.301196 0.000047

N -1.351354 0.000004 -0.000042

Cl -3.035859 -0.000002 0.000098

C -0.582936 -1.184978 -0.000116

O -1.007903 -2.301192 -0.000121

**Cl•(6-311+G**)**

Cl 0.000000 0.000000 0.000000

**1a• (NCS) (6-311+G**)**

C -0.764739 1.215801 0.013104

C 0.764841 1.215744 0.014779

H -1.213815 1.673917 -0.871960

H -1.203515 1.700894 0.889018

H 1.201439 1.698930 0.892842

H 1.216190 1.675544 -0.868228

N -0.000035 -0.940891 0.409618

C 1.159478 -0.258464 0.024745

C -1.159472 -0.258248 0.024720

O 2.231298 -0.728246 -0.211186

O -2.231386 -0.728259 -0.210450

**1i• (NCP) (6-311+G**)**

C -1.317786 -1.421458 0.013070

C -0.139989 -0.695285 0.017143

C -0.139988 0.695285 0.017138

C -1.317784 1.421458 0.013063

C -2.503527 0.700021 -0.001244

C -2.503528 -0.700019 -0.001241

H -1.303364 -2.506510 0.012636

H -1.303363 2.506511 0.012620

H -3.452745 1.226298 -0.016818

H -3.452747 -1.226295 -0.016807

C 1.275613 -1.148837 0.017047

O 1.711638 -2.247579 -0.178662

N 2.050606 -0.000001 0.330691

C 1.275615 1.148832 0.017018

O 1.711639 2.247582 -0.178643
