# Supplementary material for: Vibration-mediated long-wavelength photolysis of electronegative bonds beyond S0–S1 and S0–T1 transitions
Source: Commun Chem. 2024 Jun 4;7:126. doi: 10.1038/s42004-024-01208-0 (PMC11150518; doi:10.1038/s42004-024-01208-0)

Supplementary data 2

Vibration-mediated long-wavelength photolysis of electronegative bonds beyond S_0–S_1 and S_0–T_1 transitions

Antônio Junio Araujo Dias,[1] Atsuya Muranaka,[2] Masanobu Uchiyama,[3] Ken Tanaka,[1,*] and Yuki Nagashima[1,*]

[1] Department of Chemical Science and Engineering, Tokyo Institute of Technology, O-okayama, Meguro-ku, Tokyo 152-8550, Japan.
[2] Molecular Structure Characterization Unit, RIKEN Center for Sustainable Resource Science, 2-1 Hirosawa, Wako, Saitama 351-0198, Japan
[3] Graduate School of Pharmaceutical Sciences, The University of Tokyo, 7-3-1 Hongo, Bunkyo-ku, Tokyo 113-0033, Japan.
*e-mail: nagashima.y.ae@m.titech.ac.jp; tanaka.k.cg@m.titech.ac.jp

Table of Contents

1. NMR spectra S2

Supplementary Figure 2. ^{1}H NMR (400 MHz, $CDCl_3$) *N*,*N*-dimethylbenzamide (3a)

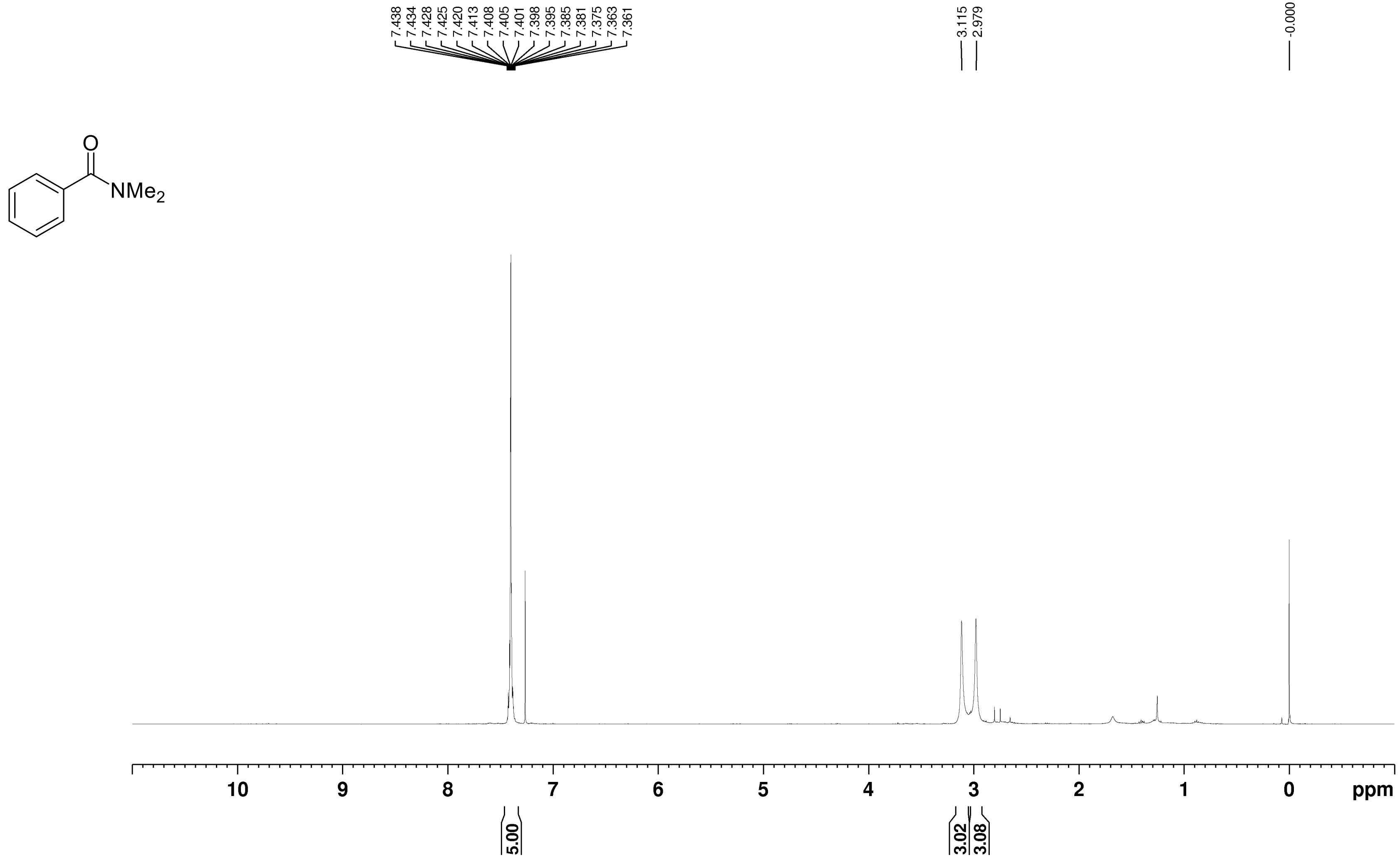

Supplementary Figure 3. **^{13}C NMR ($CDCl_3$, 100 MHz) *N,N*-dimethylbenzamide (3a)**

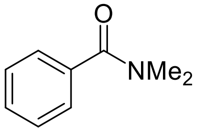

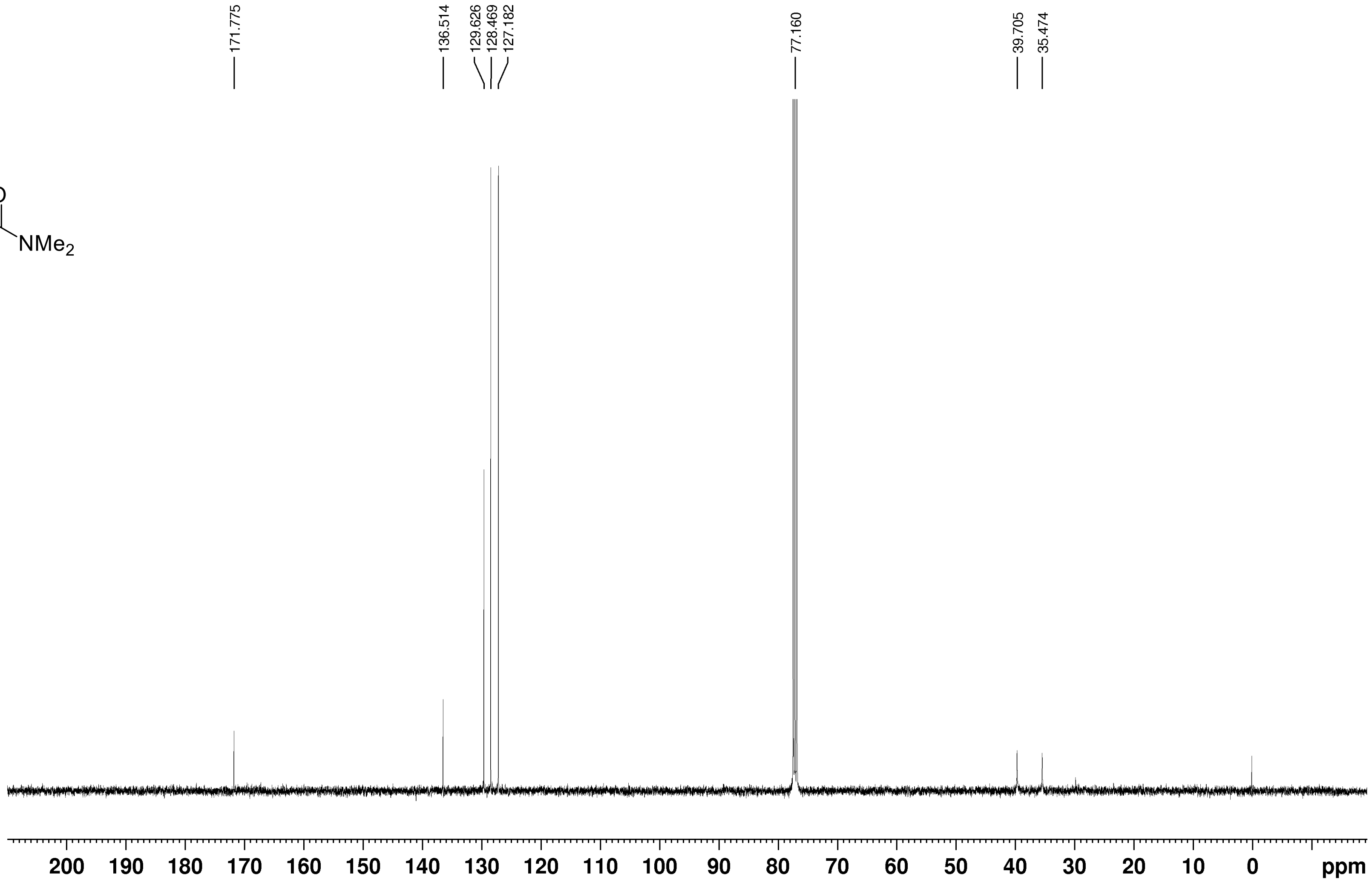

Supplementary Figure 4. ^{1}H NMR (400 MHz, $CDCl_3$) *N,N*-dimethyl-[1,1'-biphenyl]-4-carboxamide (3b)

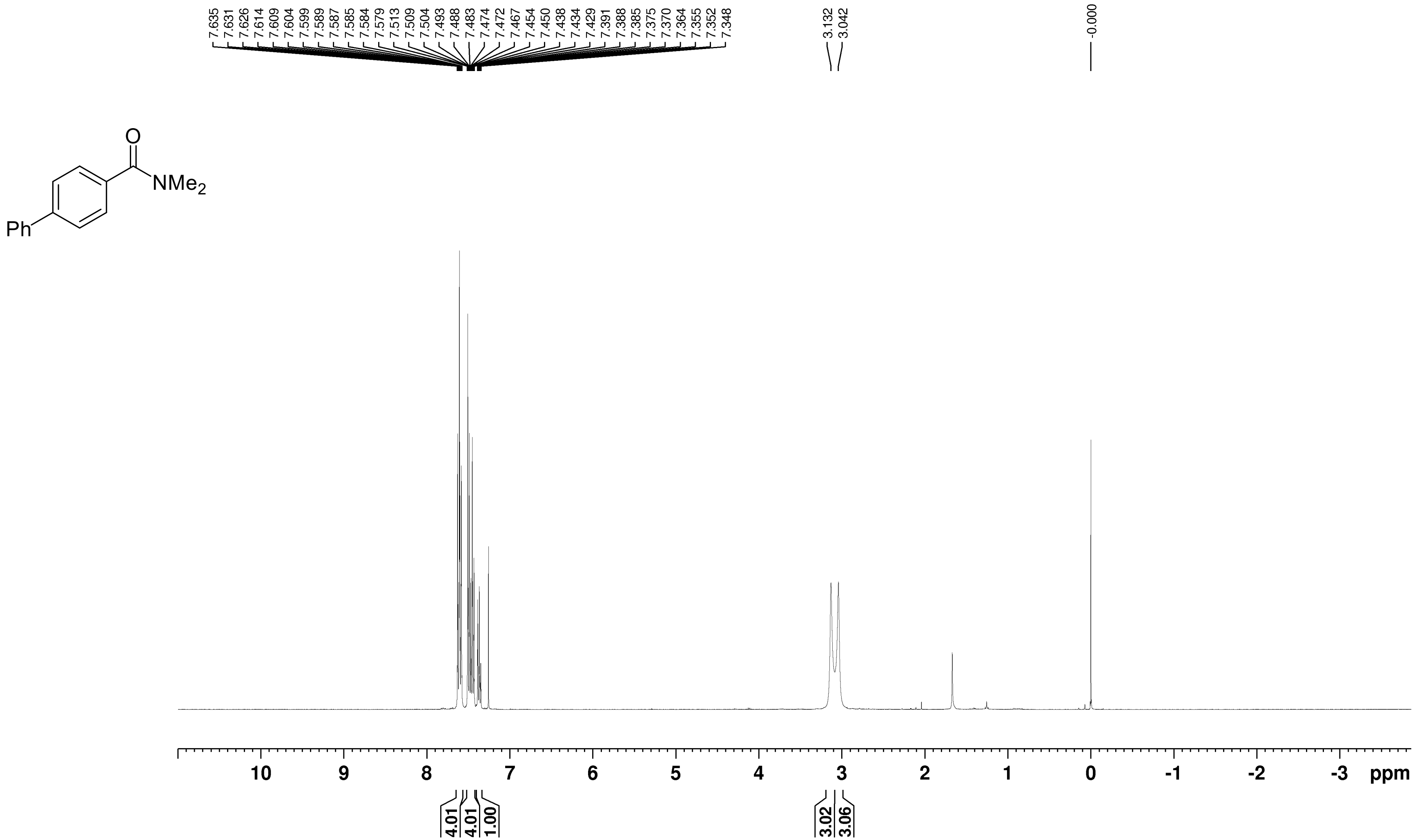

Supplementary Figure 5. ^{13}C NMR ($CDCl_3$, 100 MHz) *N*,*N*-dimethyl-[1,1'-biphenyl]-4-carboxamide (3b)

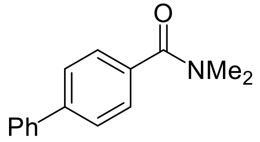

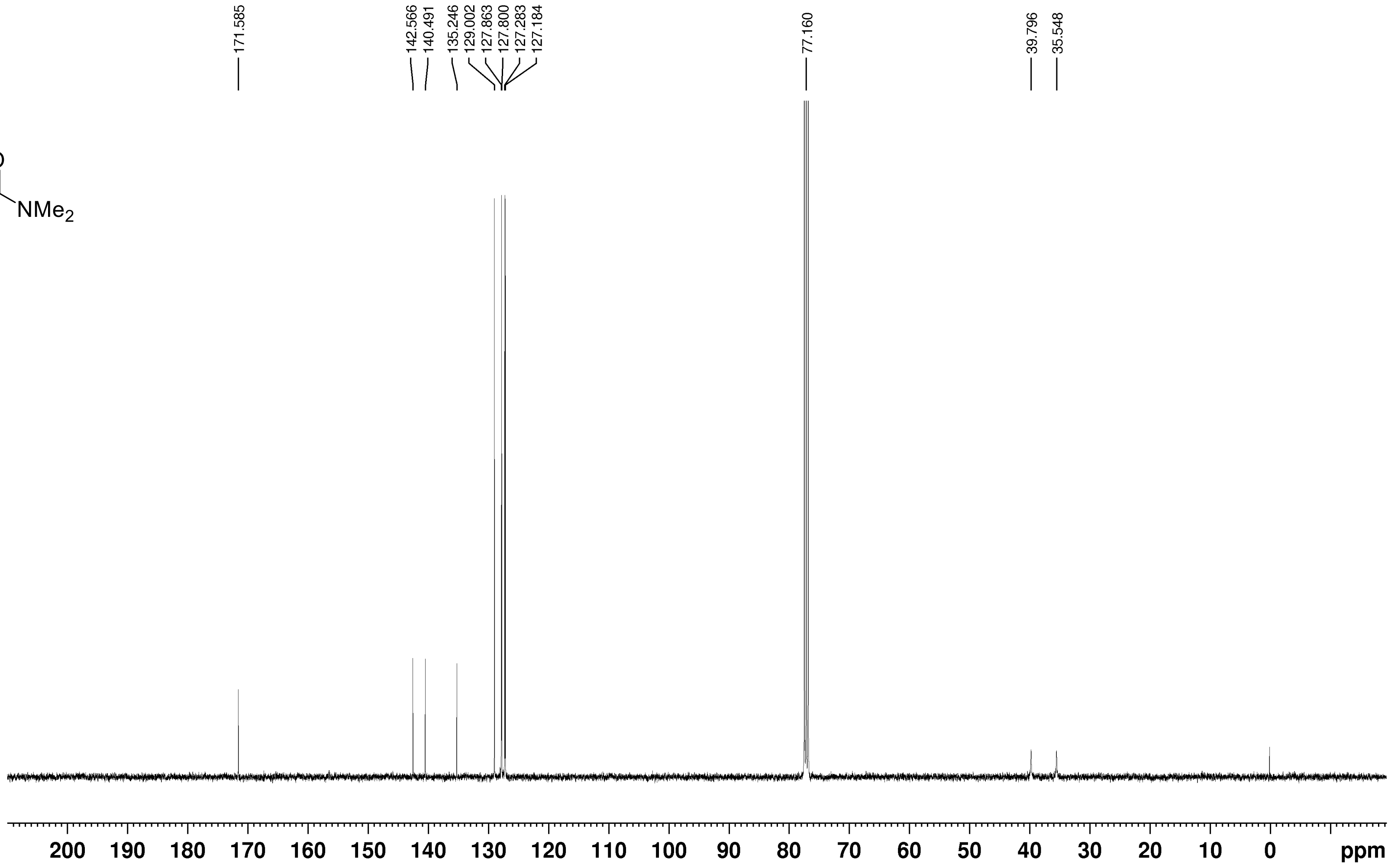

Supplementary Figure 6. ^{1}H NMR (400 MHz, $CDCl_3$) *N*,*N*-dimethyl-(*p*-methoxycarbonyl)-benzamide (3c)

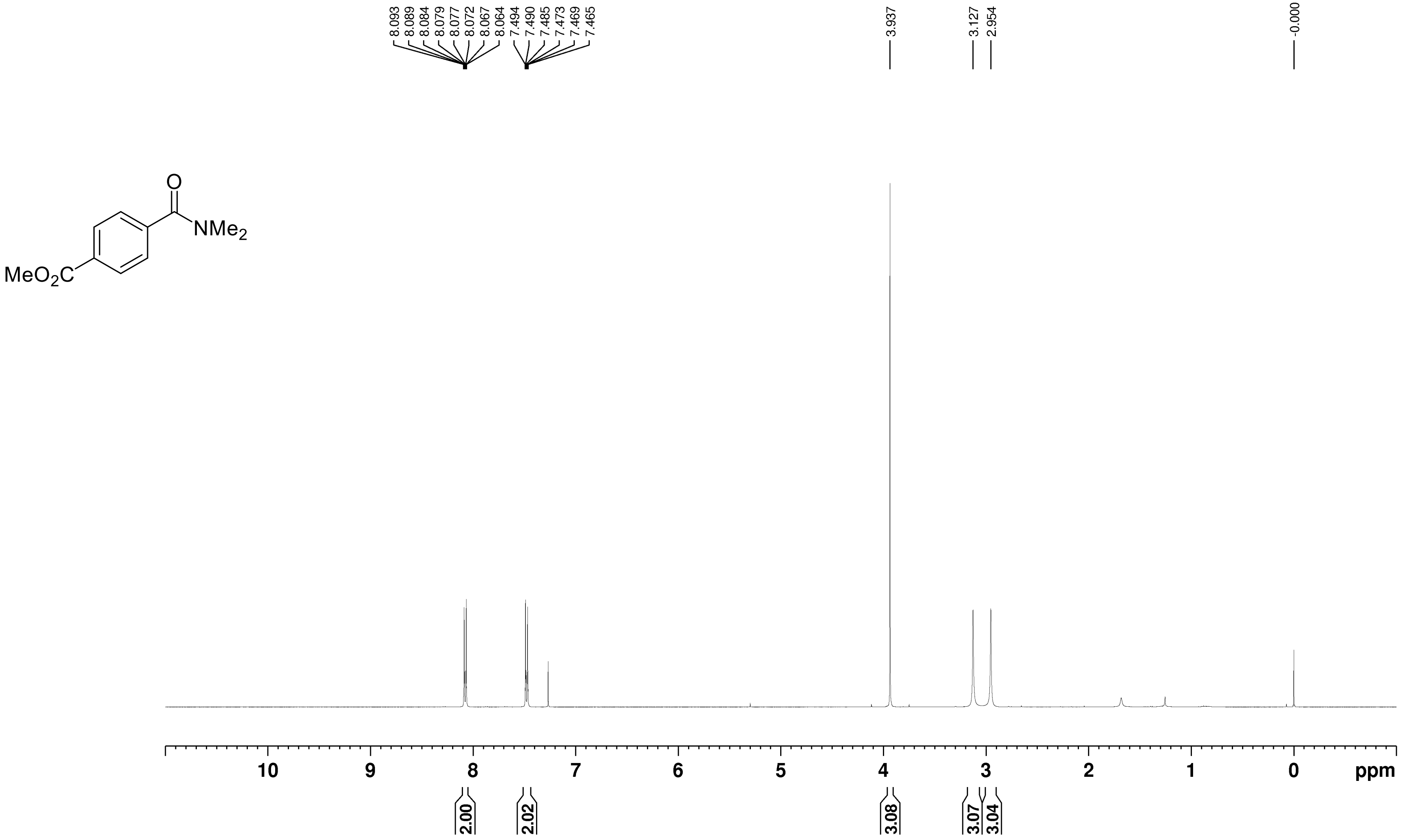

Supplementary Figure 7. ^{13}C NMR ($CDCl_3$, 100 MHz) *N*,*N*-dimethyl-(*p*-methoxycarbonyl)-benzamide (3c)

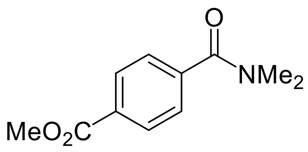

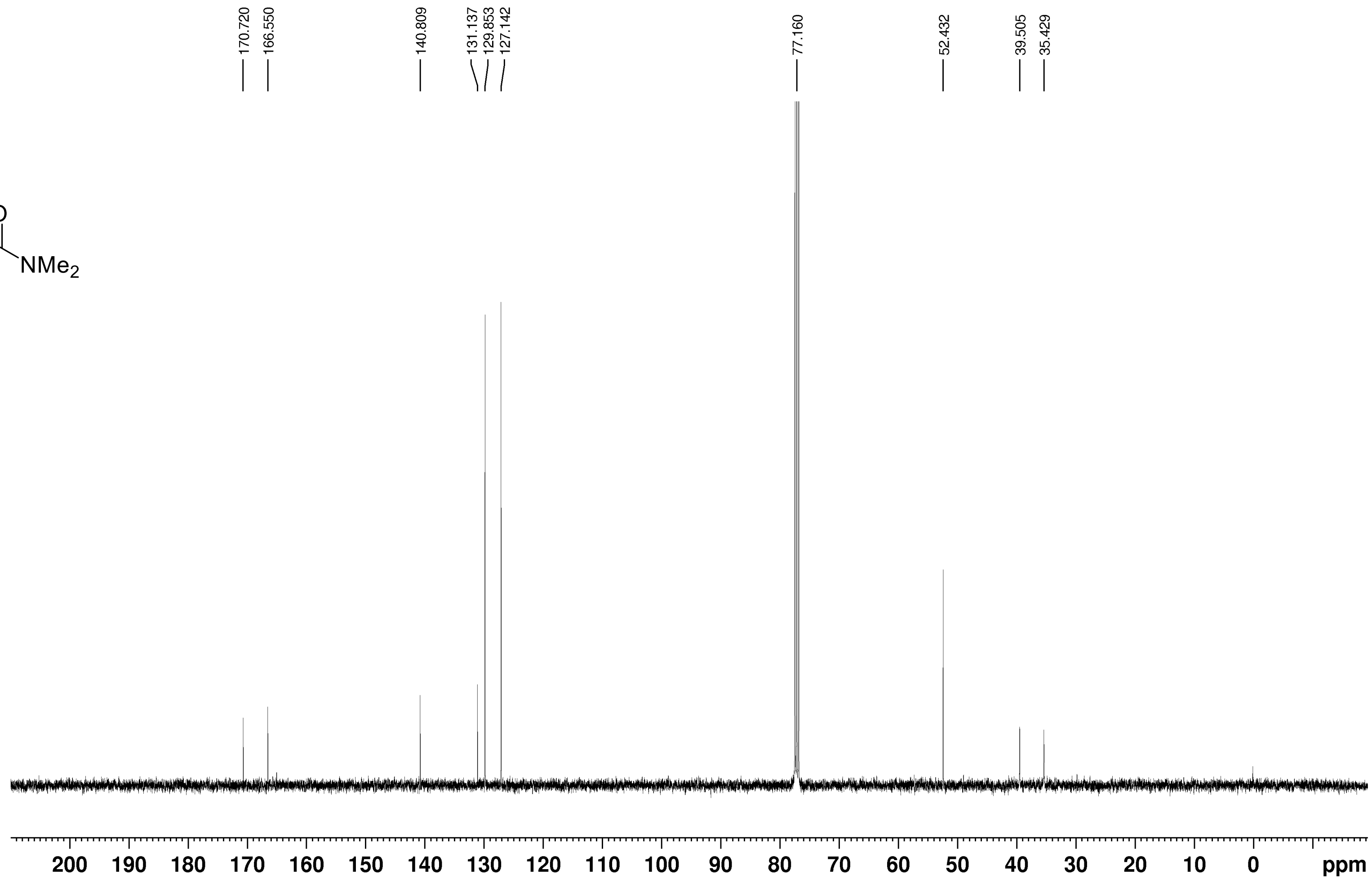

Supplementary Figure 8. ^{1}H NMR (400 MHz, $CDCl_3$) 4-chloro-*N*,*N*-dimethylbenzamide (3d)

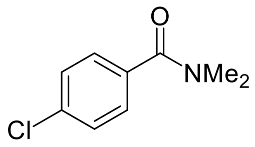

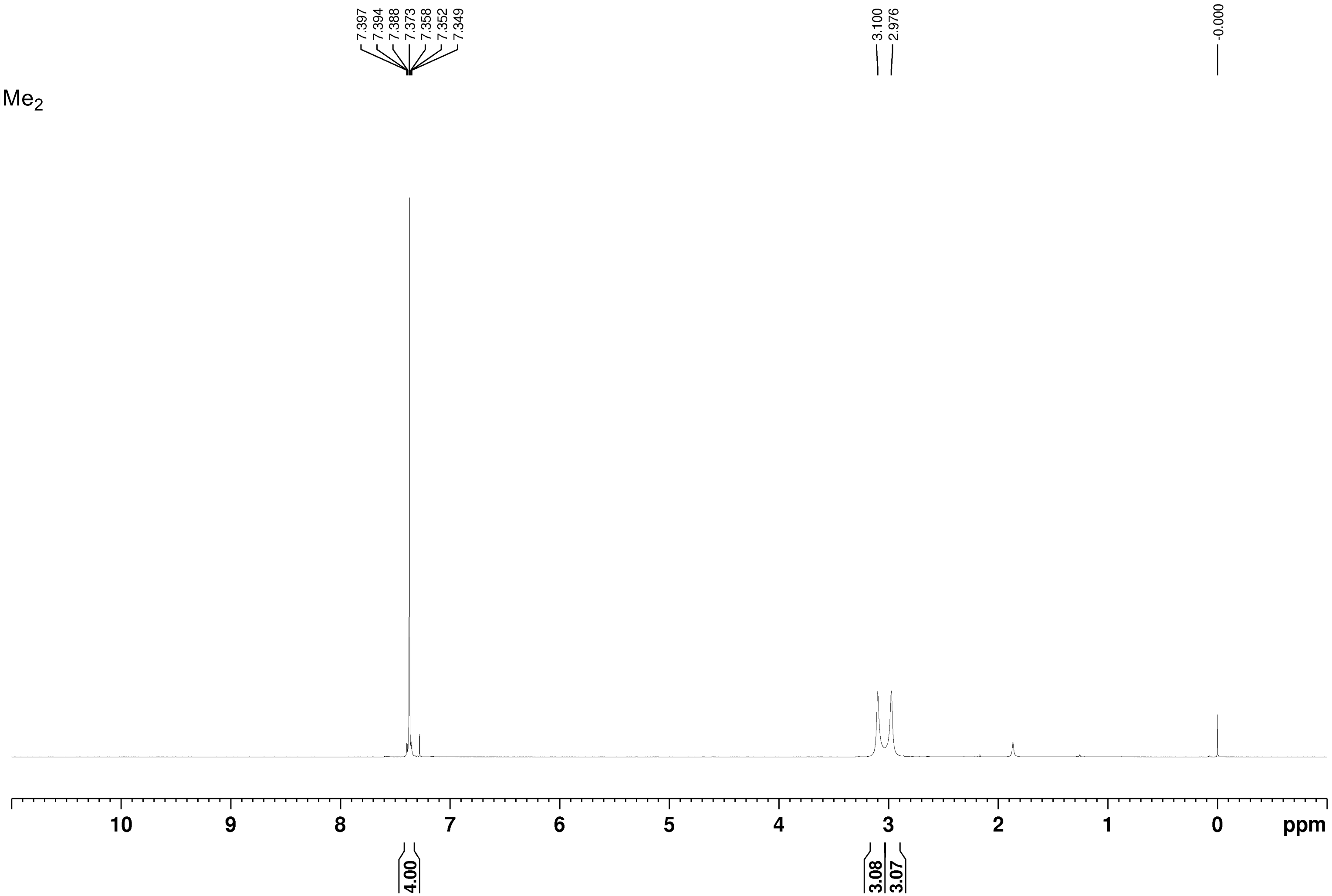

Supplementary Figure 9. ^{13}C NMR ($CDCl_3$, 100 MHz) 4-chloro-*N*,*N*-dimethylbenzamide (3d)

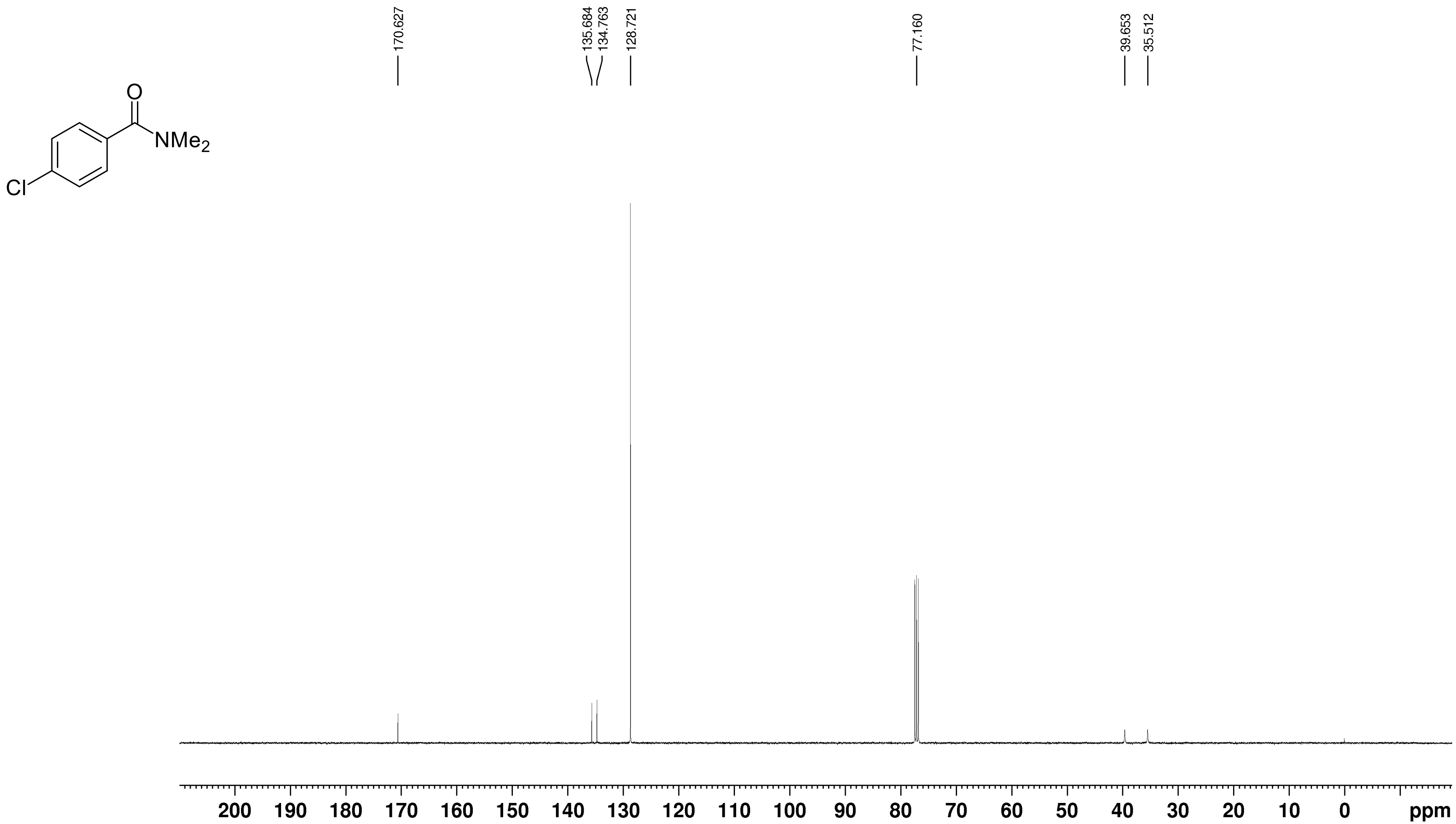

Supplementary Figure 10. ^{1}H NMR (400 MHz, $CDCl_3$) 3-chloro-*N*,*N*-dimethylbenzamide (3e)

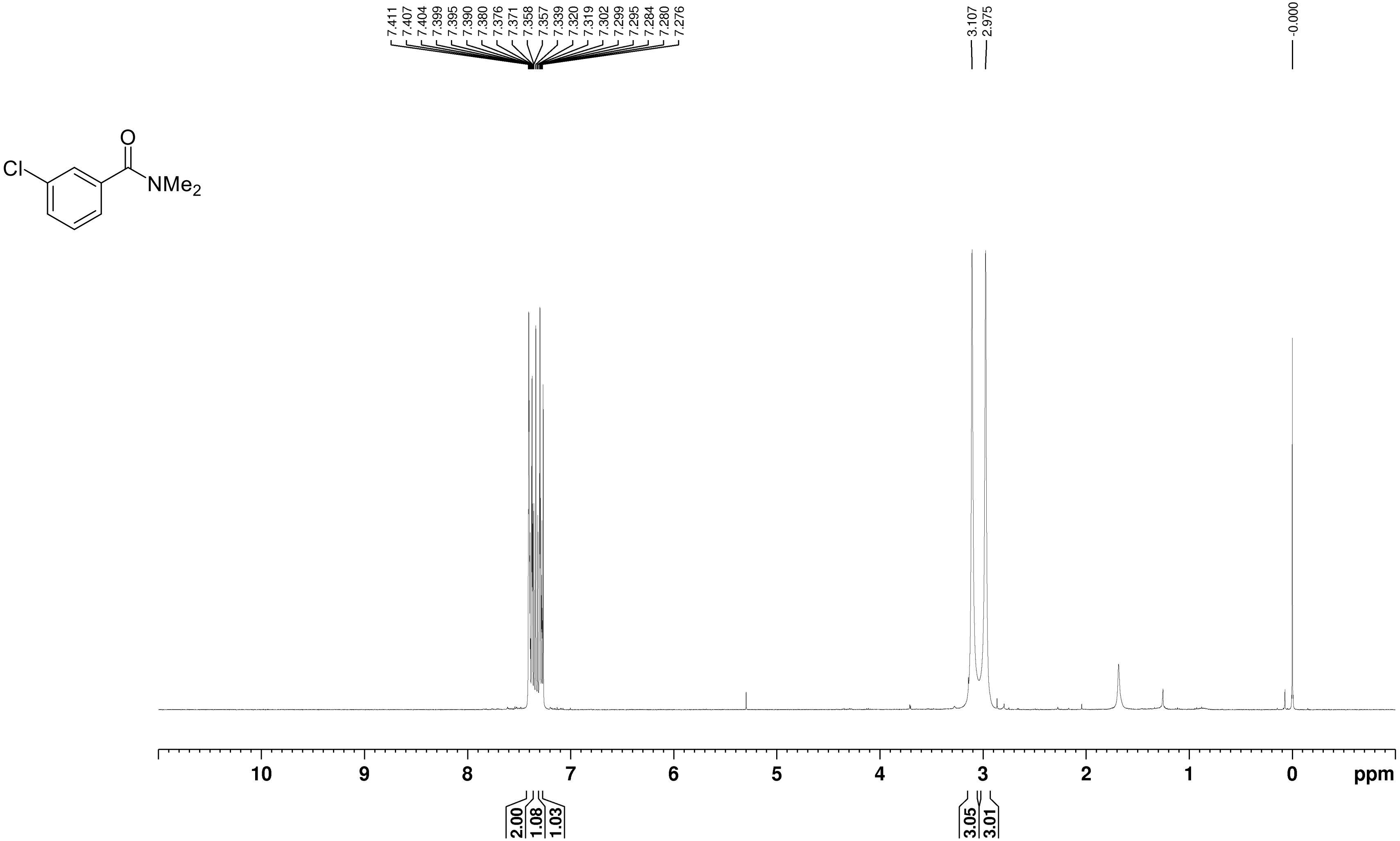

Supplementary Figure 11. ^{13}C **NMR (** $CDCl_3$ **, 100 MHz) 3-chloro-*N*,*N*-dimethylbenzamide (3e)**

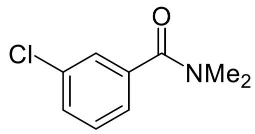

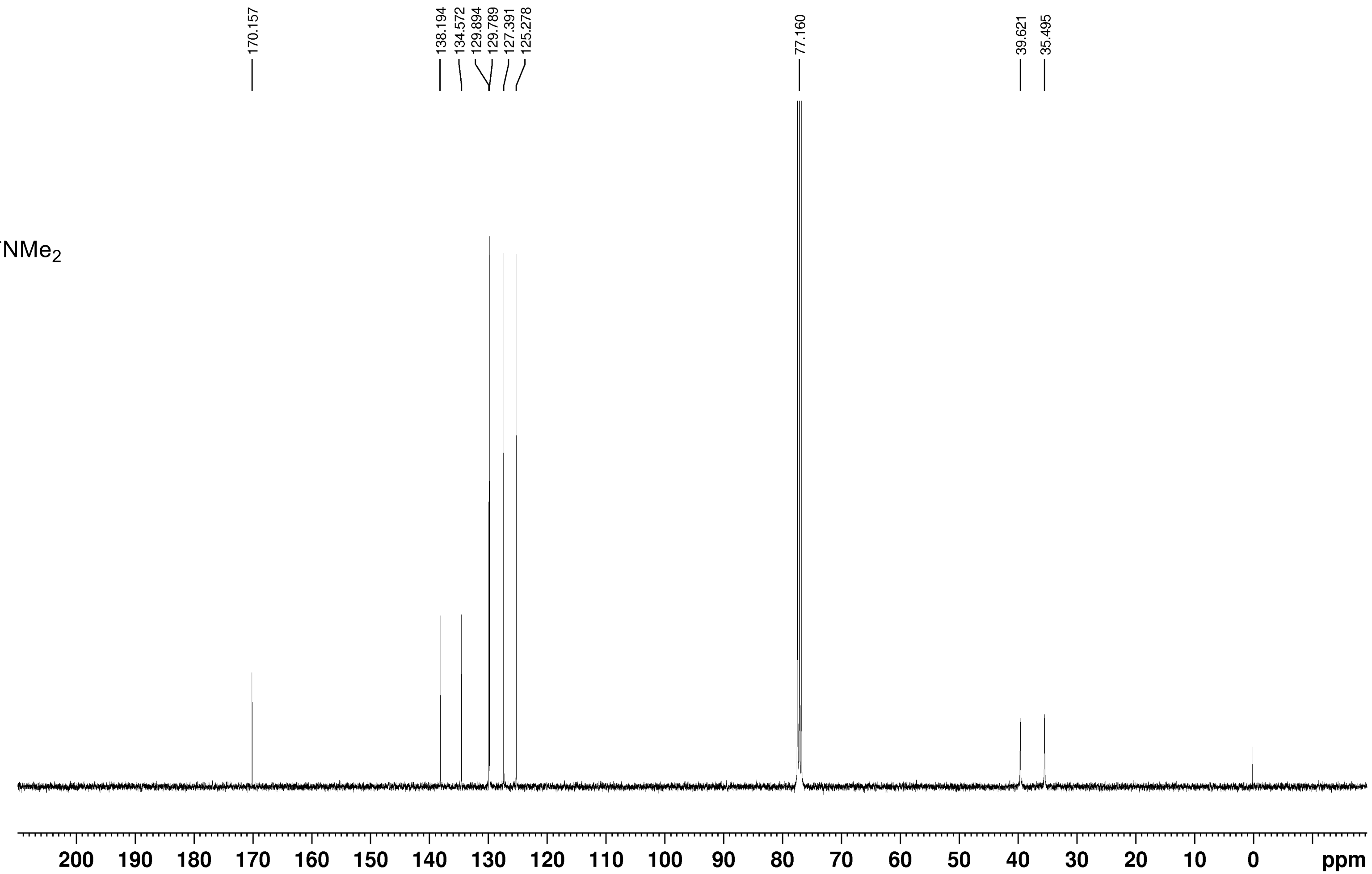

Supplementary Figure 12. ¹H NMR (400 MHz, $CDCl_3$) 2-chloro-*N*,*N*-dimethylbenzamide (3f)

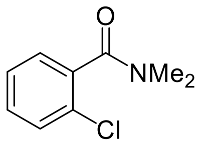

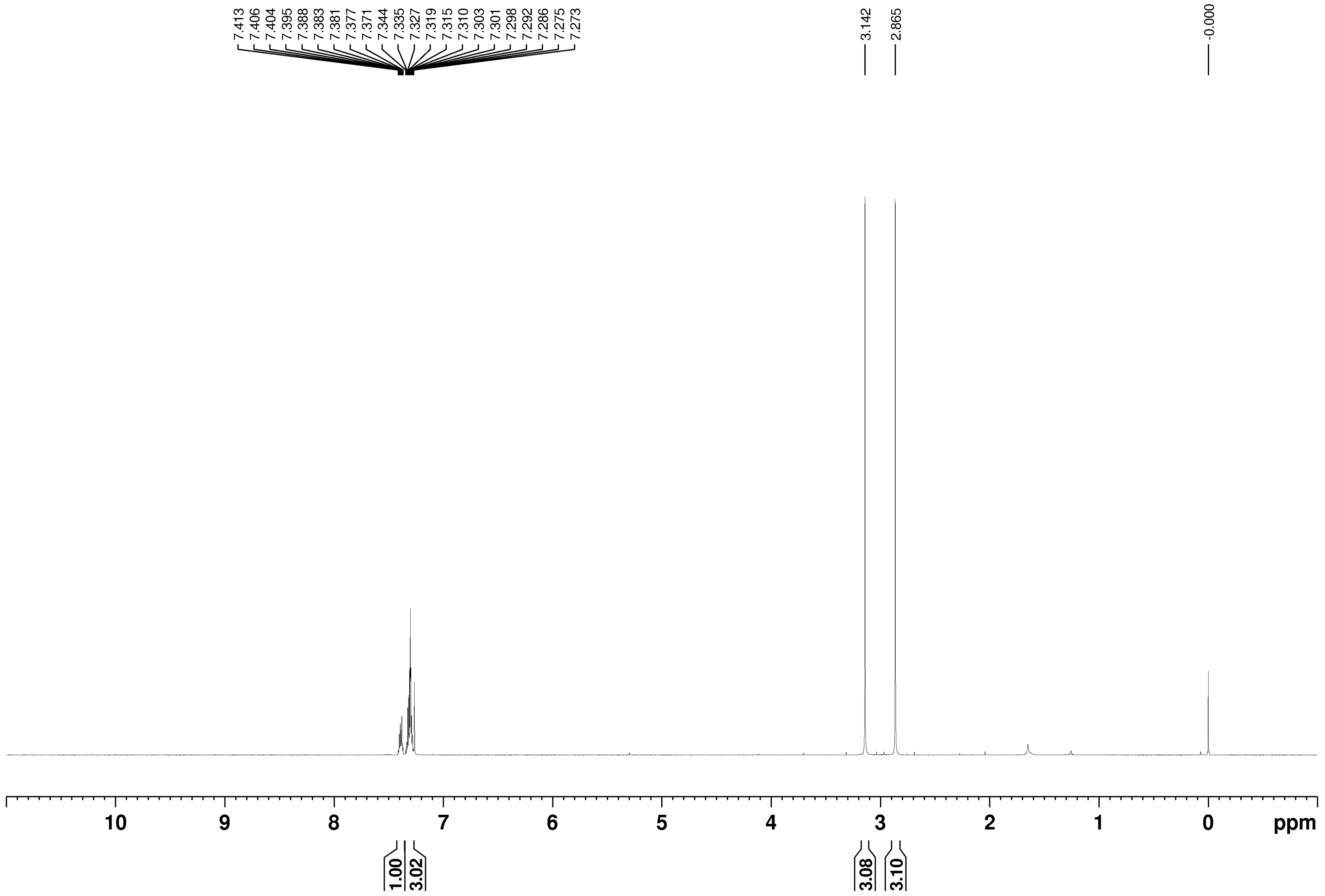

Supplementary Figure 13. ^{13}C NMR ($CDCl_3$, 100 MHz) 2-chloro-*N*,*N*-dimethylbenzamide (3f)

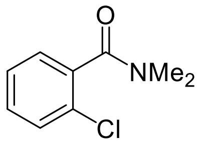

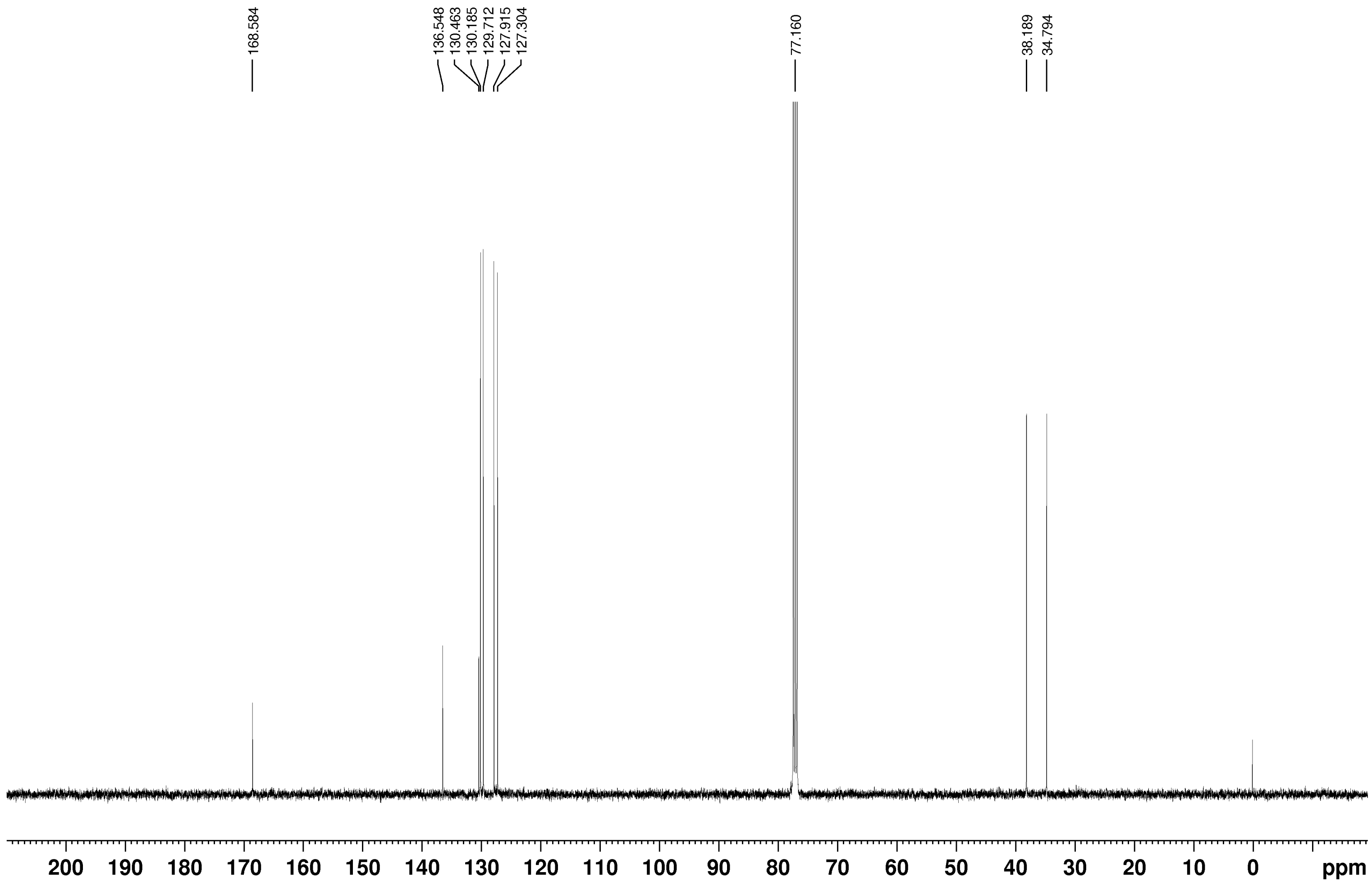

Supplementary Figure 14. ^{1}H NMR (400 MHz, $CDCl_3$) *N*-(*tert*-butyl)-4-fluorobenzamide (3g)

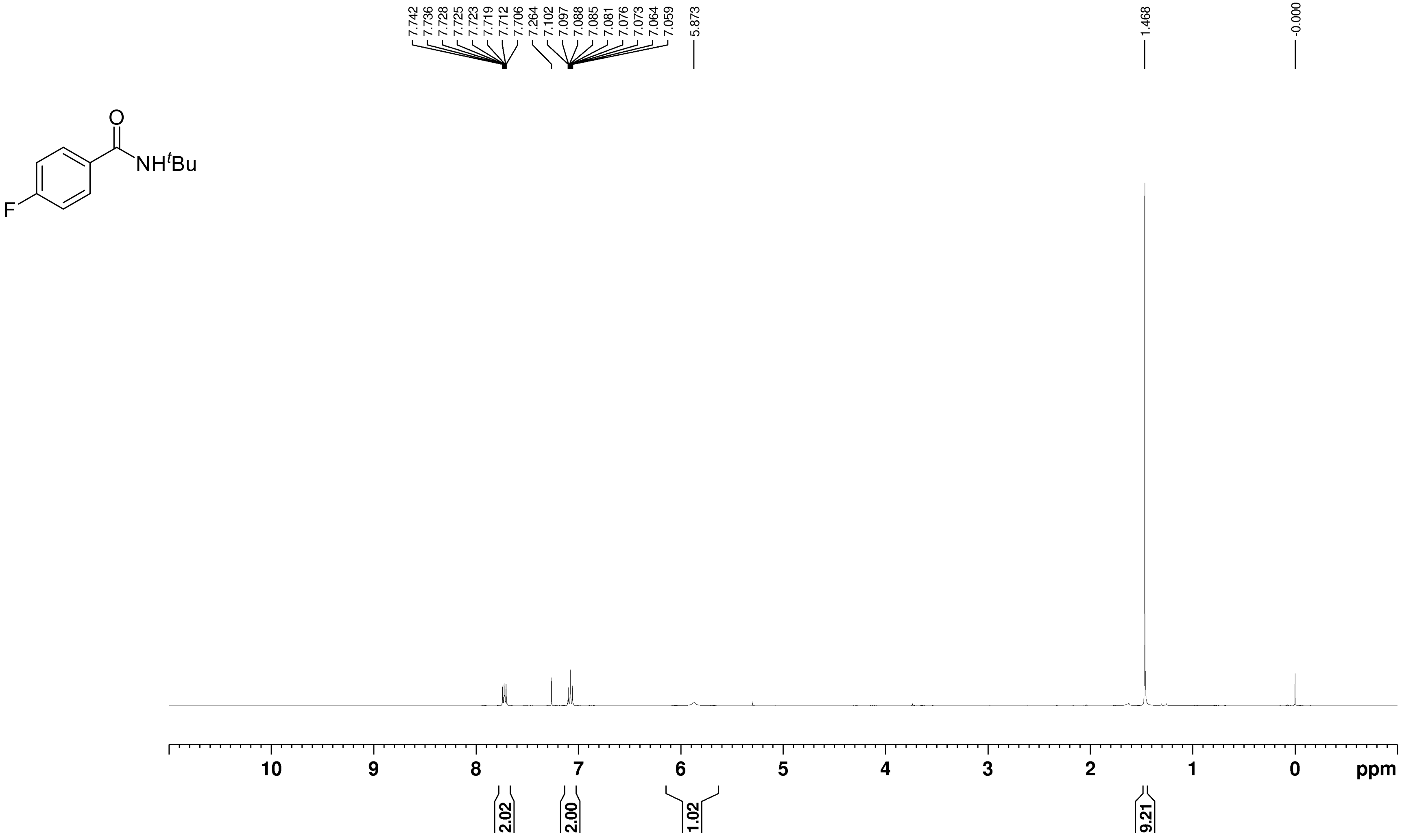

Supplementary Figure 15. ^{13}C NMR ($CDCl_3$, 100 MHz) *N*-(*tert*-butyl)-4-fluorobenzamide (3g)

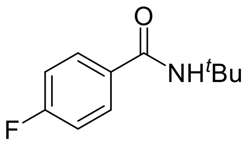

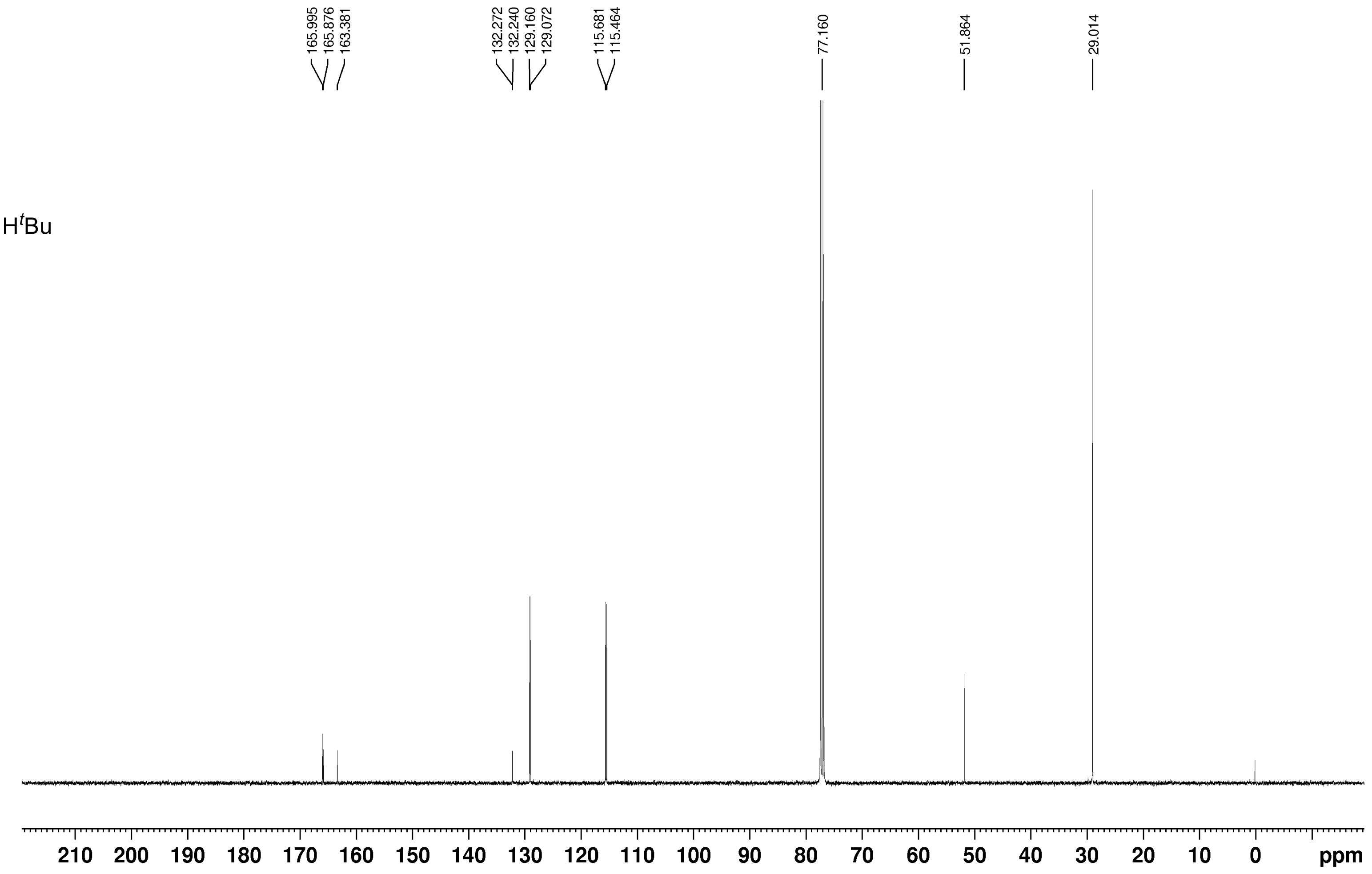

Supplementary Figure 16. ^{19}F NMR ($CDCl_3$, 376 MHz) *N*-(*tert*-butyl)-4-fluorobenzamide (3g)

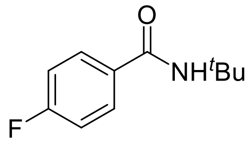

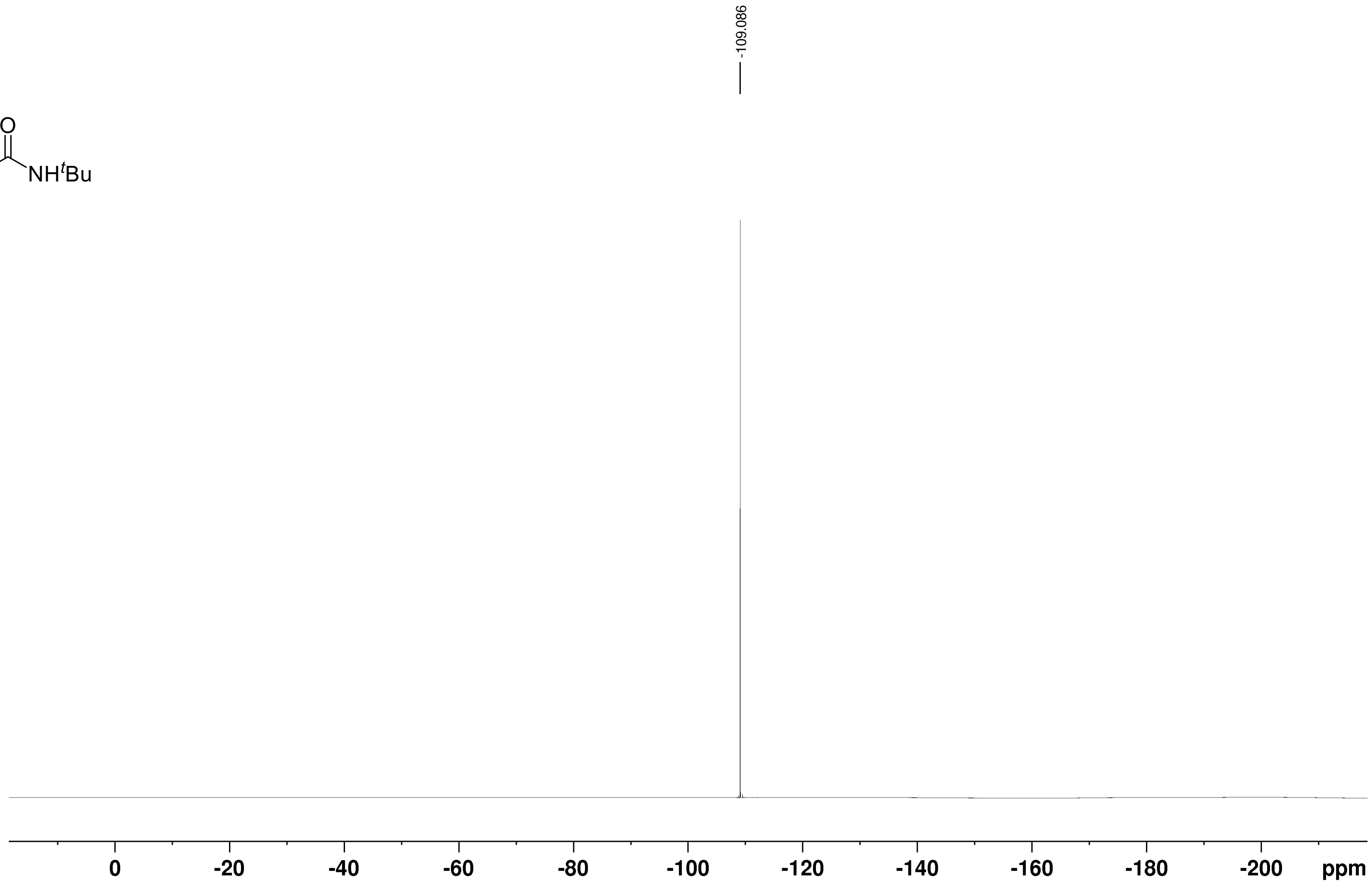

Supplementary Figure 17. ^{1}H NMR (400 MHz, $CDCl_3$) *N,N*-dimethyl-4-(trifluoromethyl)benzamide (3h)

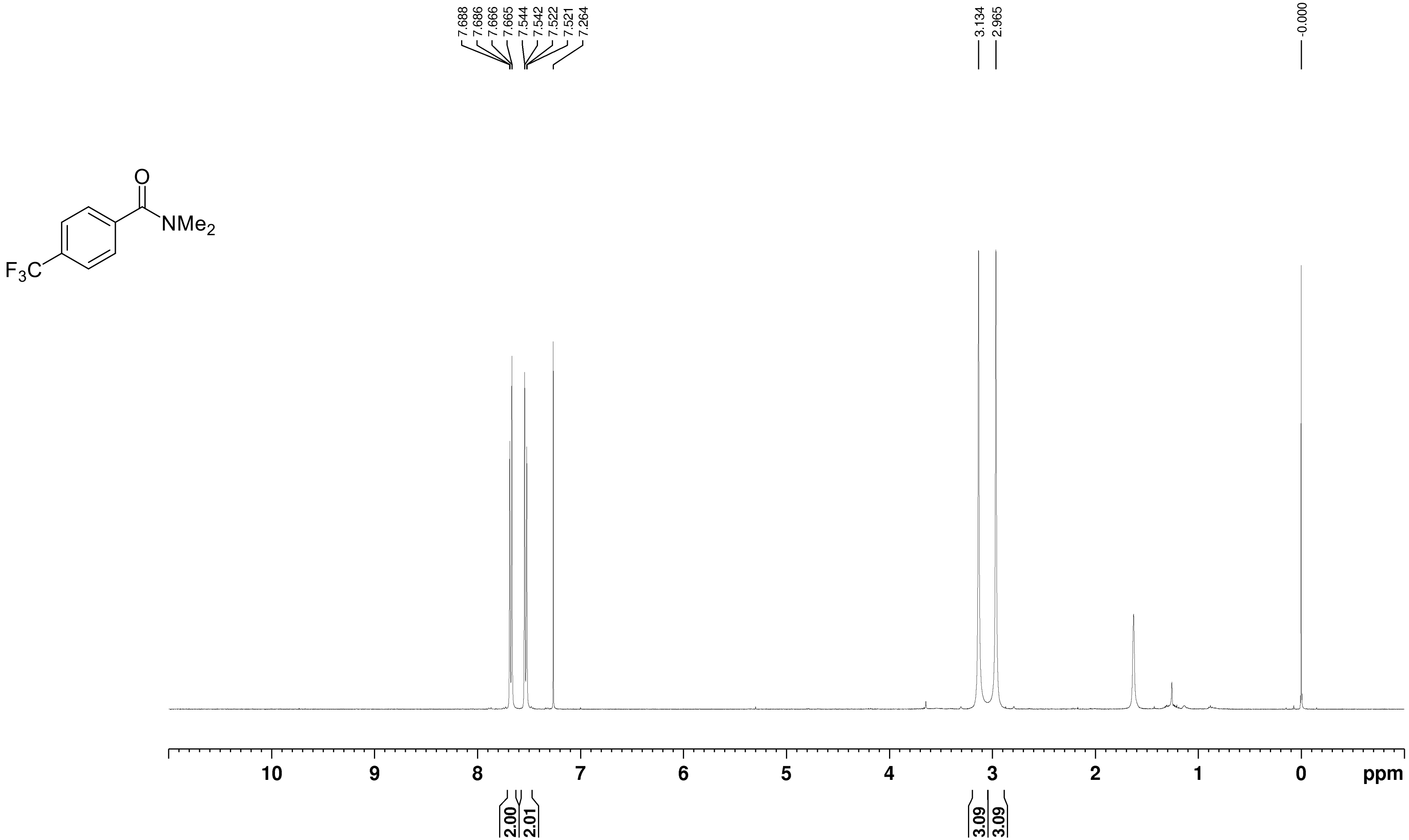

Supplementary Figure 18. ^{13}C NMR ($CDCl_3$, 100 MHz) *N,N*-dimethyl-4-(trifluoromethyl)benzamide (3h)

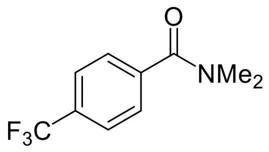

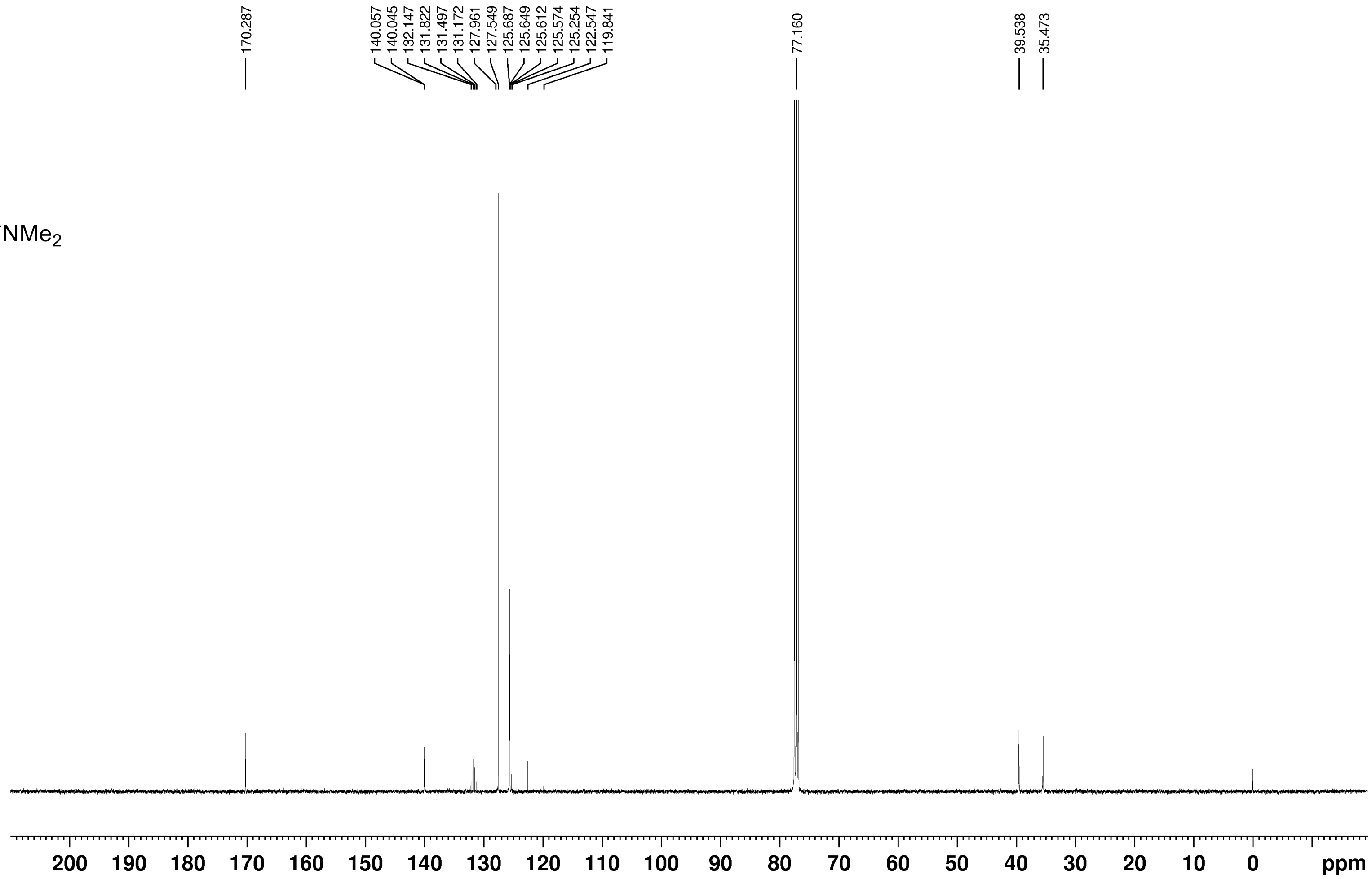

Supplementary Figure 19. ^{19}F NMR ($CDCl_3$, 376 MHz) *N*,*N*-dimethyl-4-(trifluoromethyl)benzamide (3h)

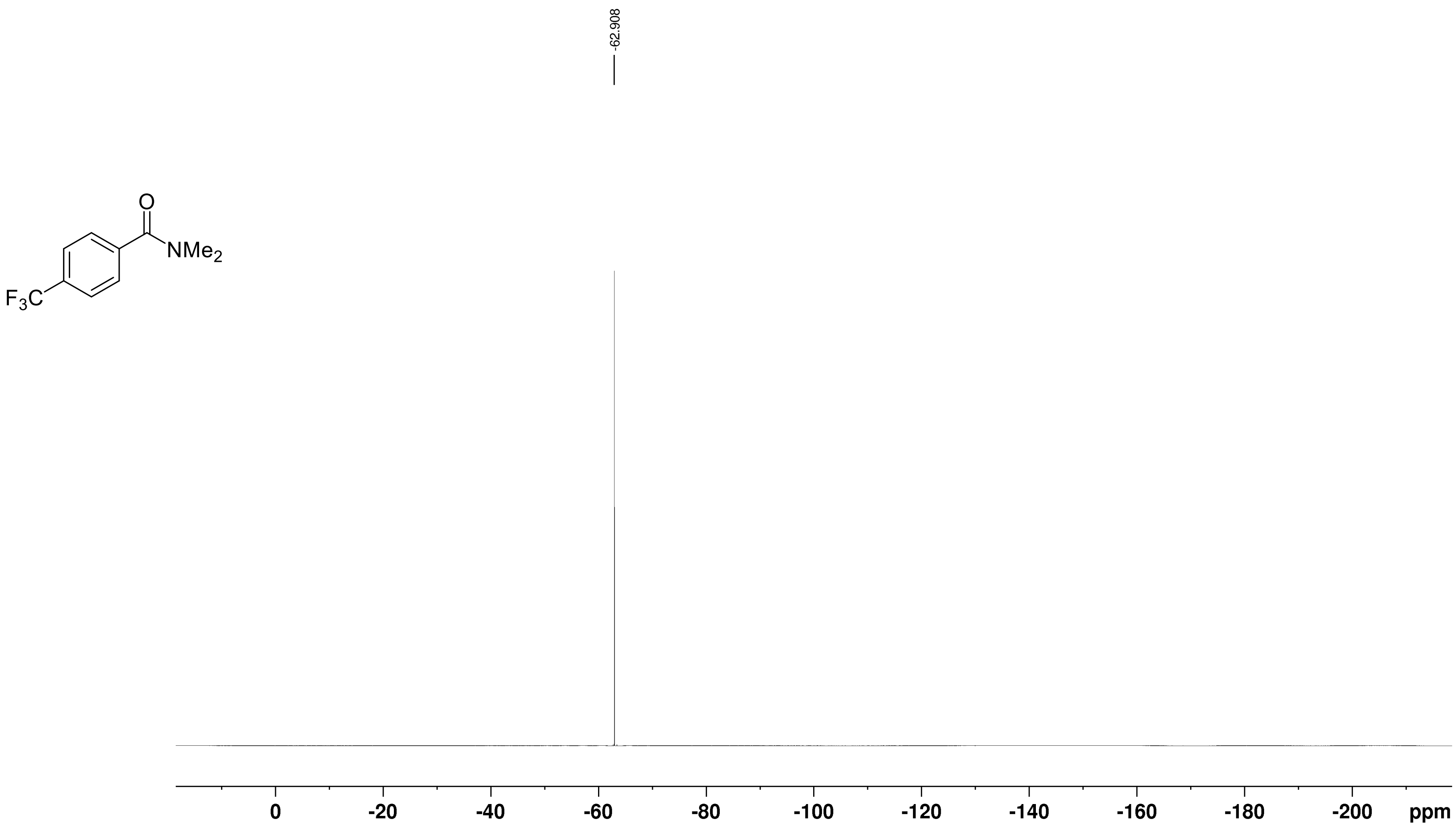

Supplementary Figure 20. ^{1}H NMR (400 MHz, $CDCl_3$) 4-bromo-*N*,*N*-dimethylbenzamide (3i)

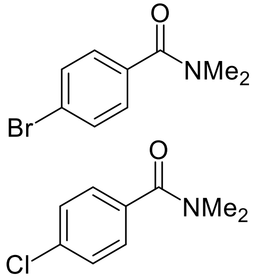

mixture 89/11

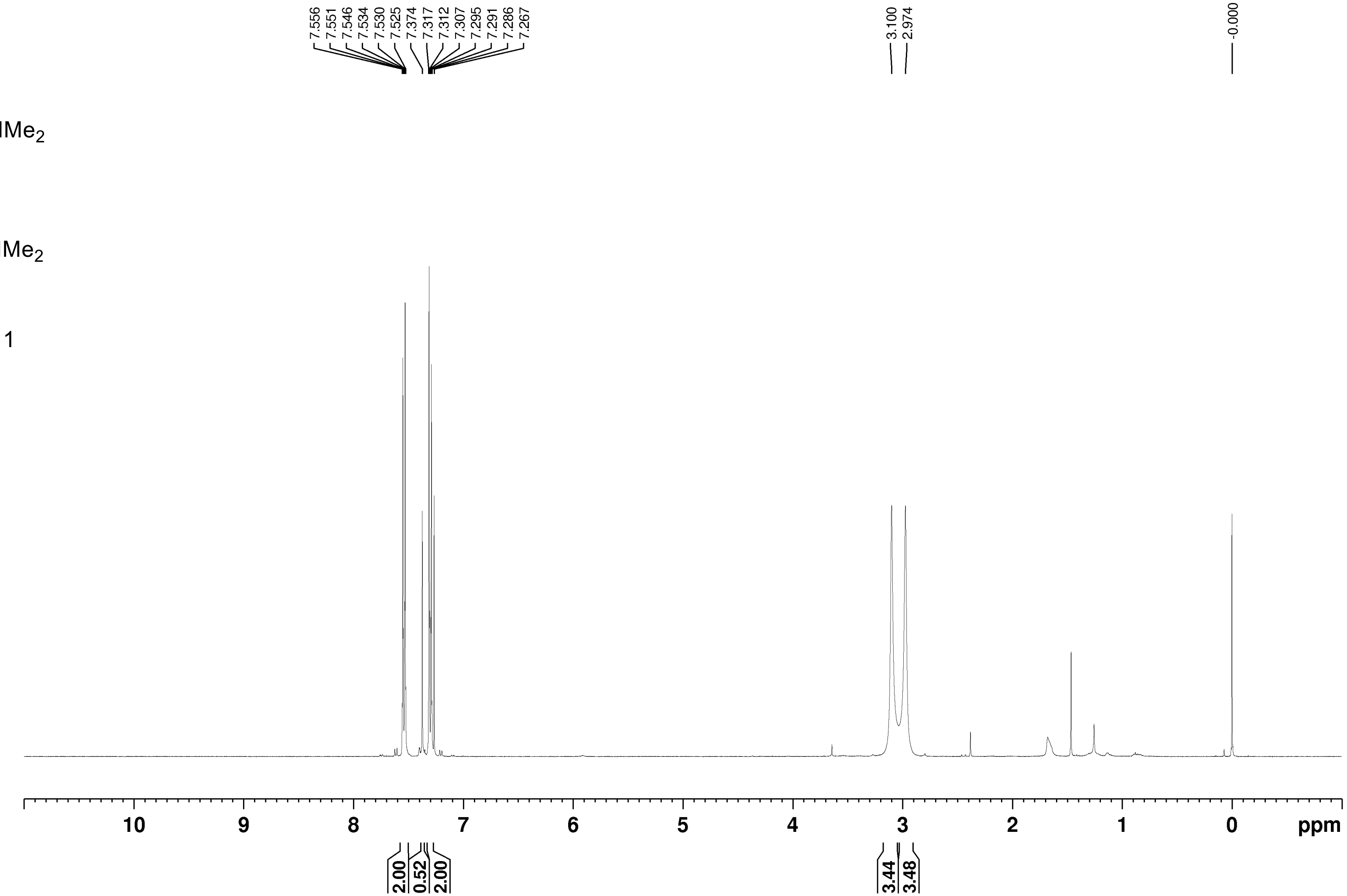

Supplementary Figure 21. ^{13}C NMR (CDCl$_3$, 100 MHz) 4-bromo-*N*,*N*-dimethylbenzamide (3i)

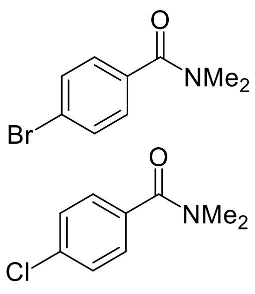

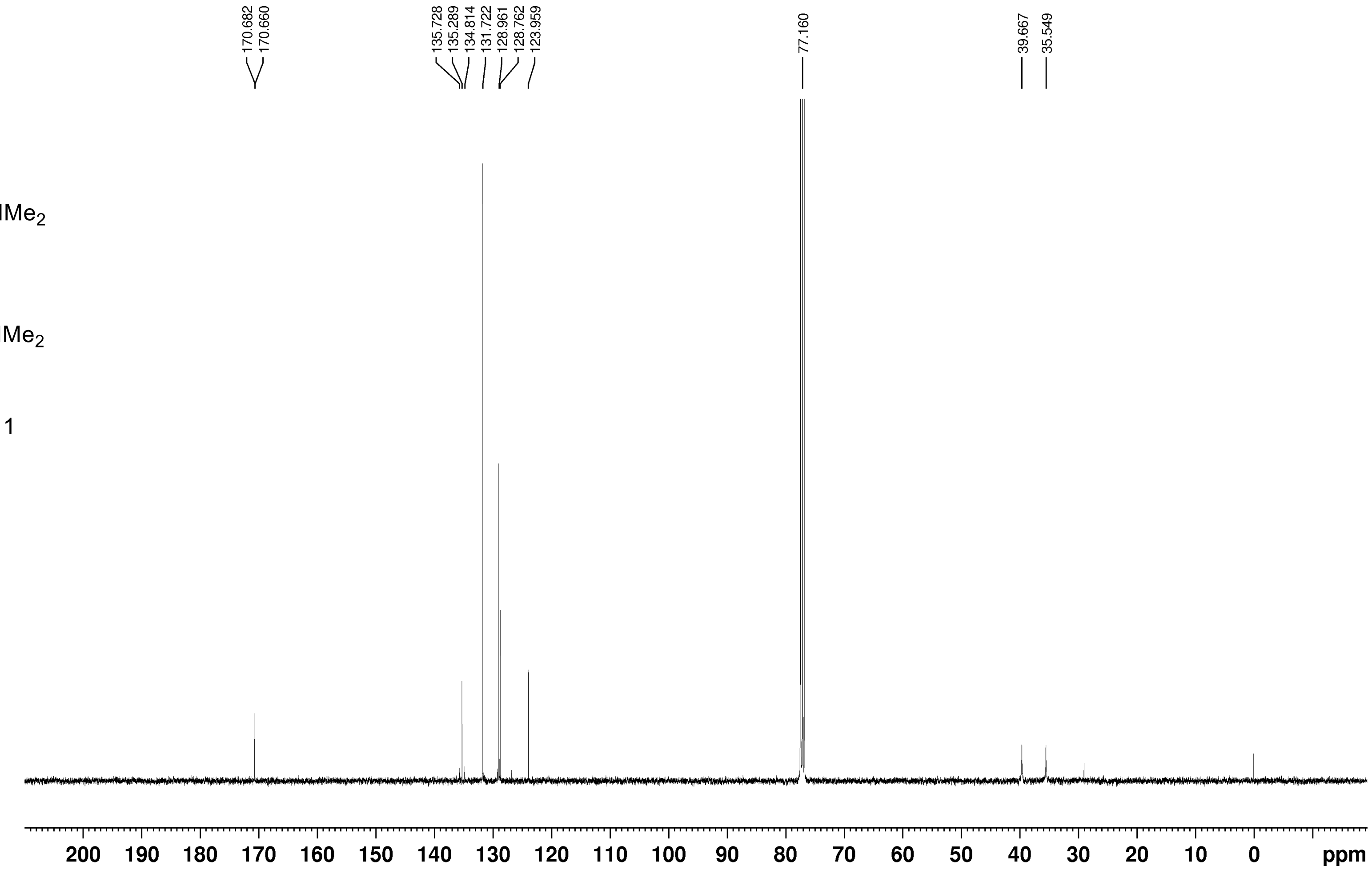

Supplementary Figure 22. ^1H NMR (400 MHz, $CDCl_3$) 2-bromo-4-chloro-*N*,*N*-dimethylbenzamide (3j)

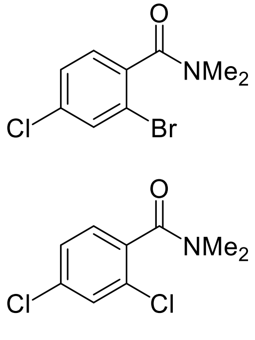

mixture 84/16

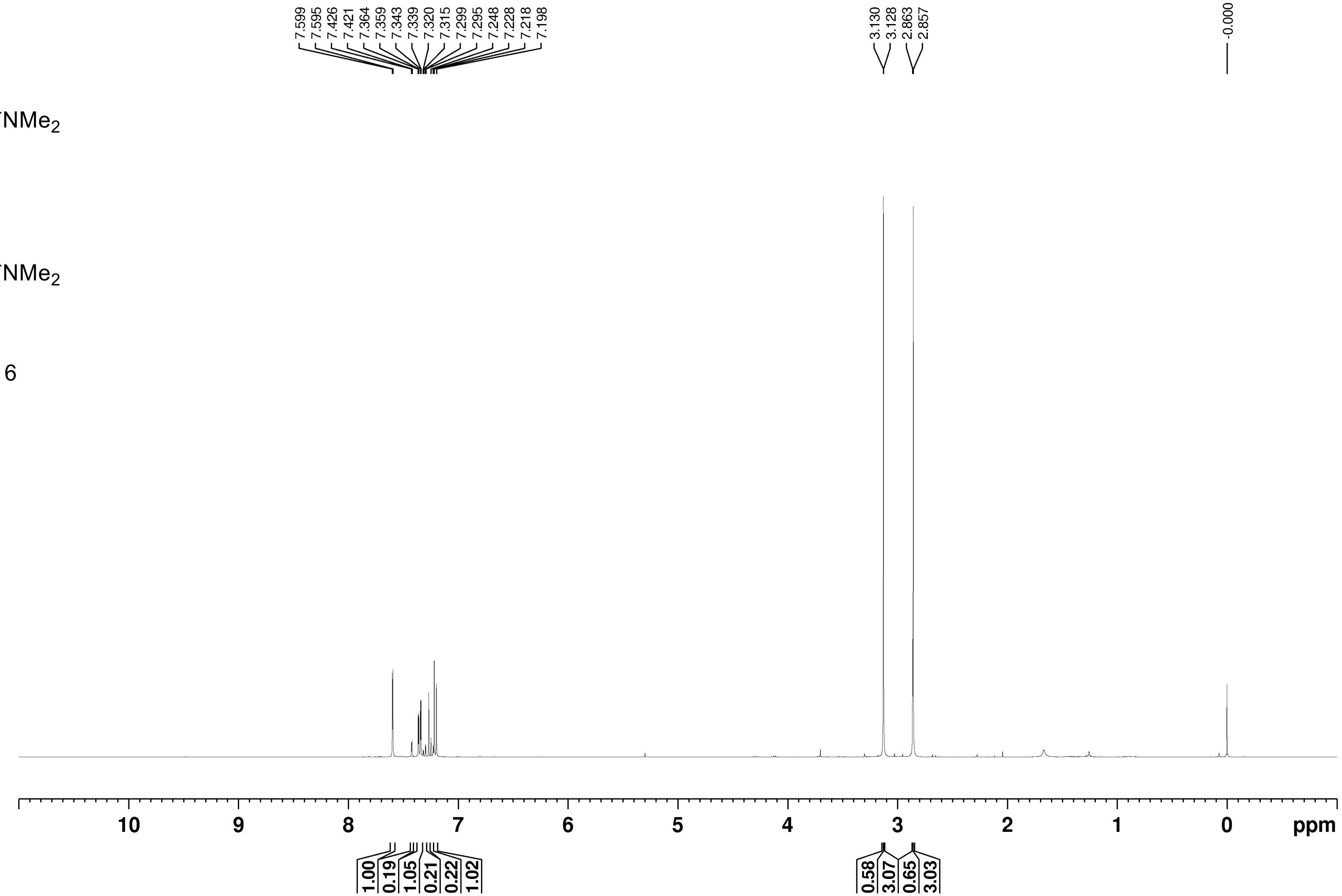

Supplementary Figure 23. ^{13}**C NMR (CDCl$_3$, 100 MHz) 2-bromo-4-chloro-*N*,*N*-dimethylbenzamide (3j)**

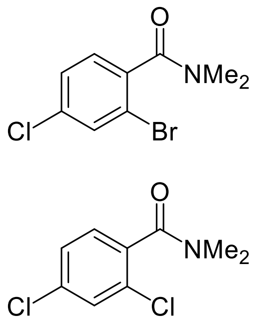

mixture 84/16

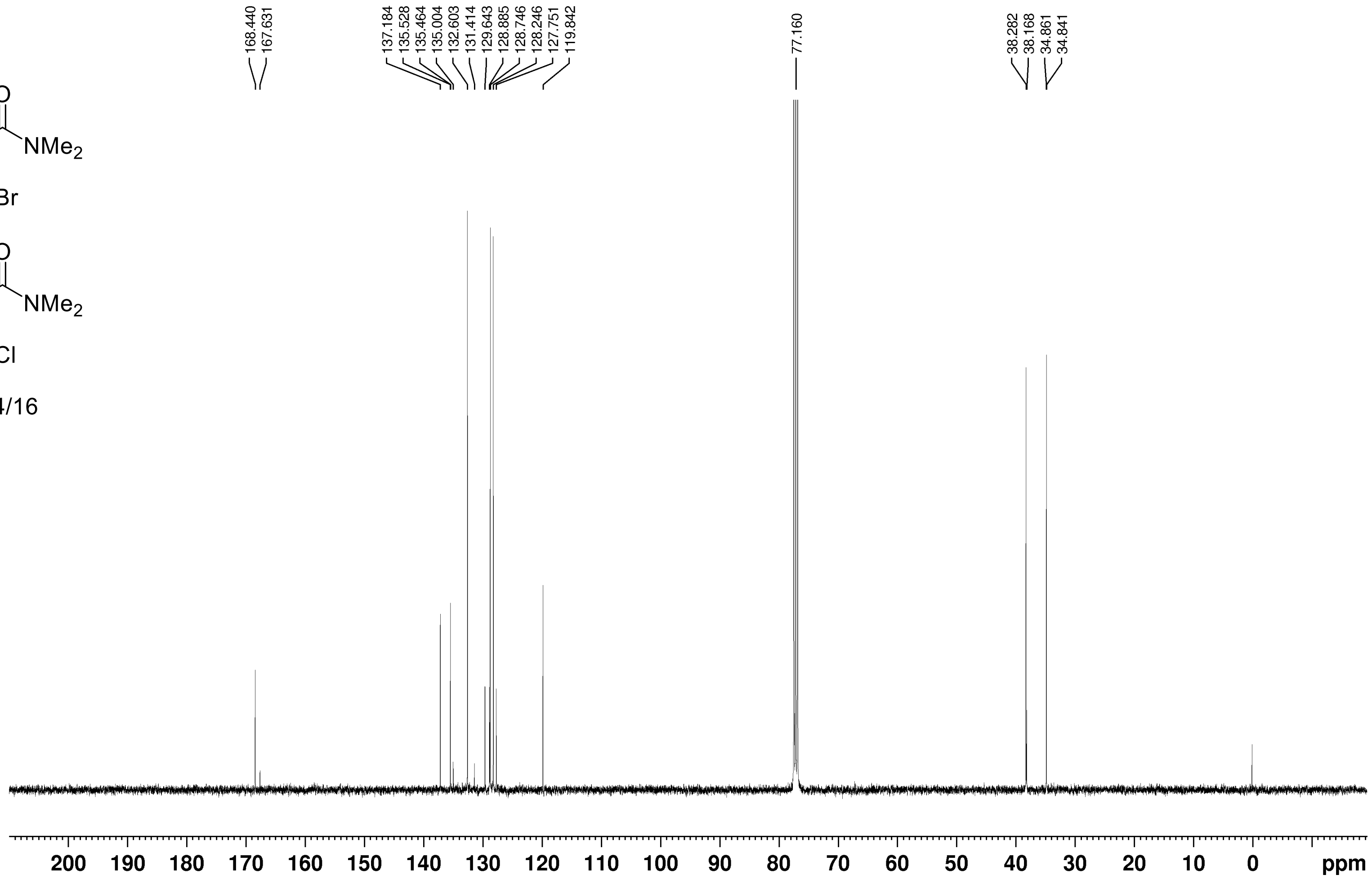

Supplementary Figure 24. ^{1}H NMR (400 MHz, $CDCl_3$) 2-cyano-*N*,*N*-dimethylbenzamide (3k)

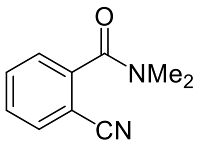

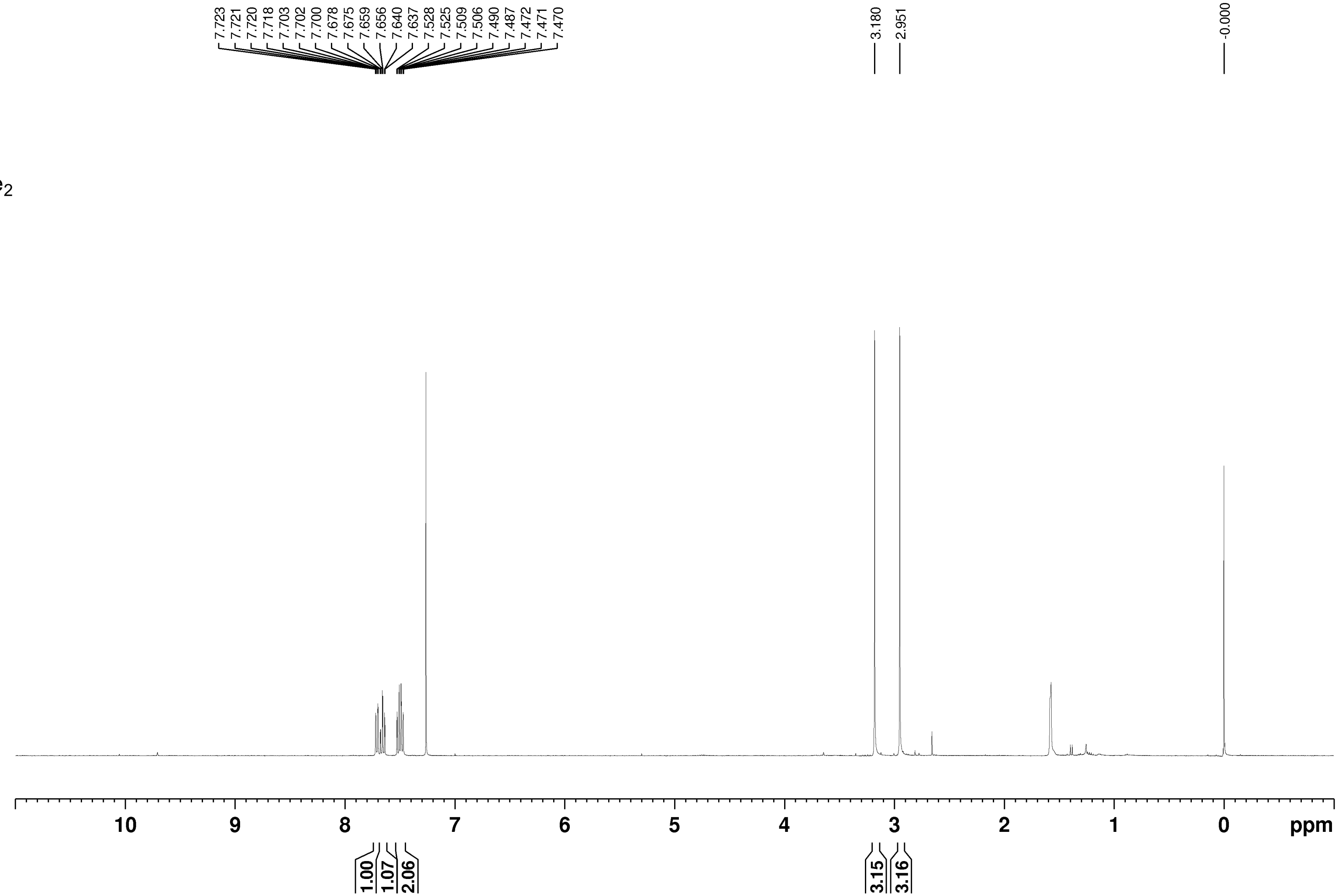

Supplementary Figure 25. ^{13}C NMR ($CDCl_3$, 100 MHz) 2-cyano-*N*,*N*-dimethylbenzamide (3k)

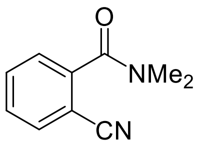

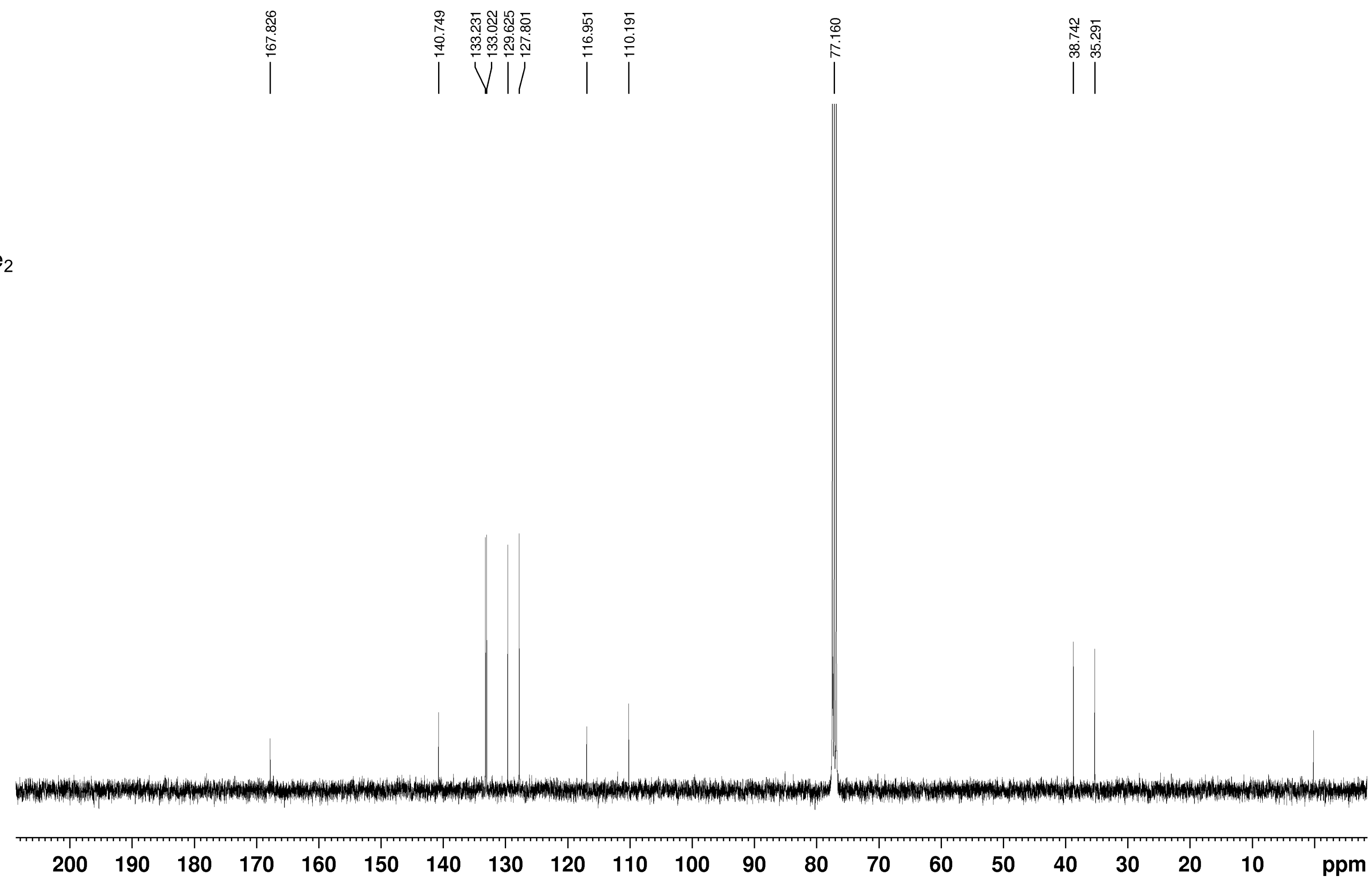

Supplementary Figure 26. ^{1}H NMR (400 MHz, $CDCl_3$) *N*-(*tert*-butyl)-4-methylbenzamide (3l)

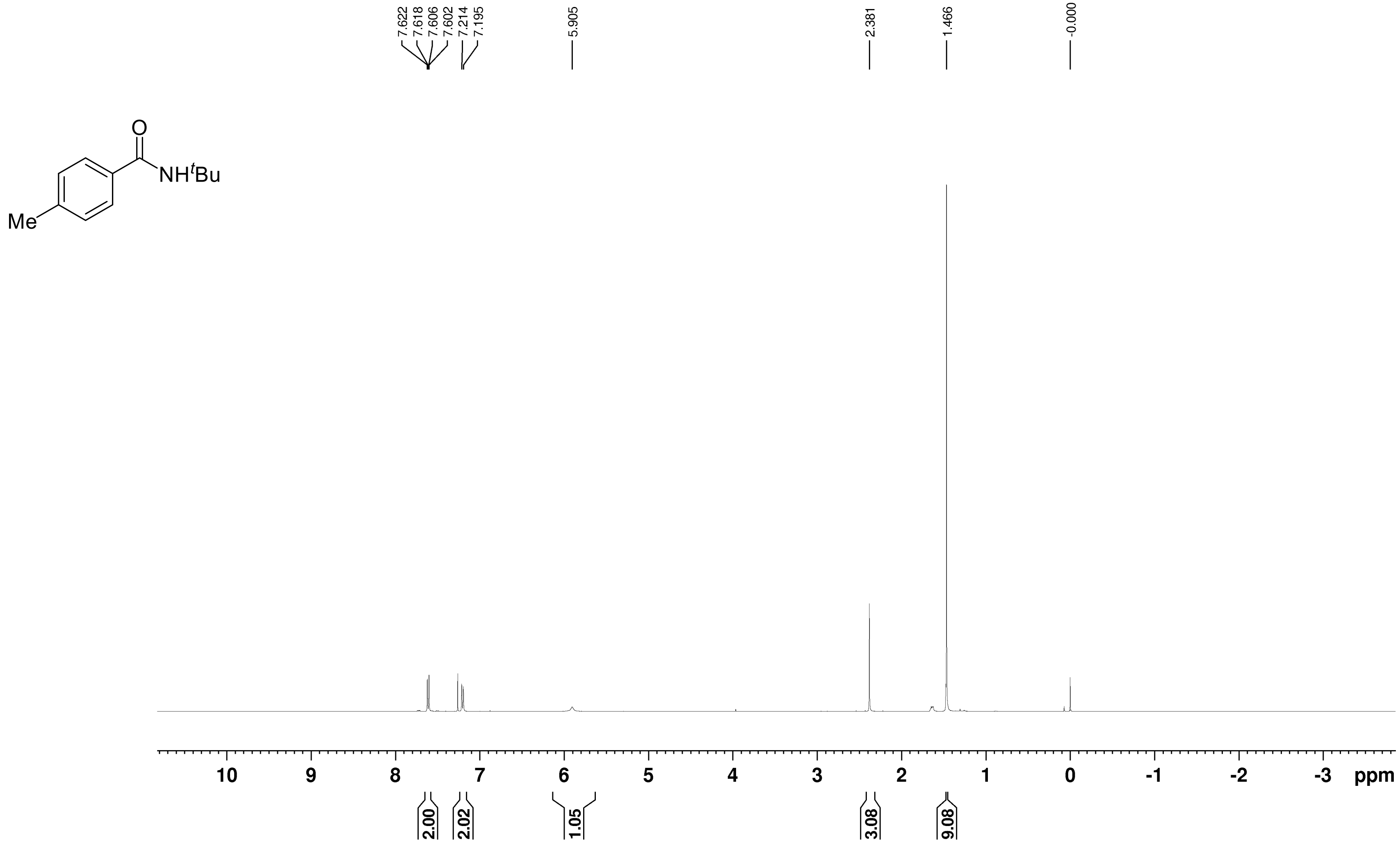

Supplementary Figure 27. ^{13}C NMR ($CDCl_3$, 100 MHz) *N*-(*tert*-butyl)-4-methylbenzamide (3l)

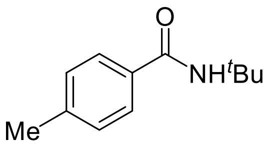

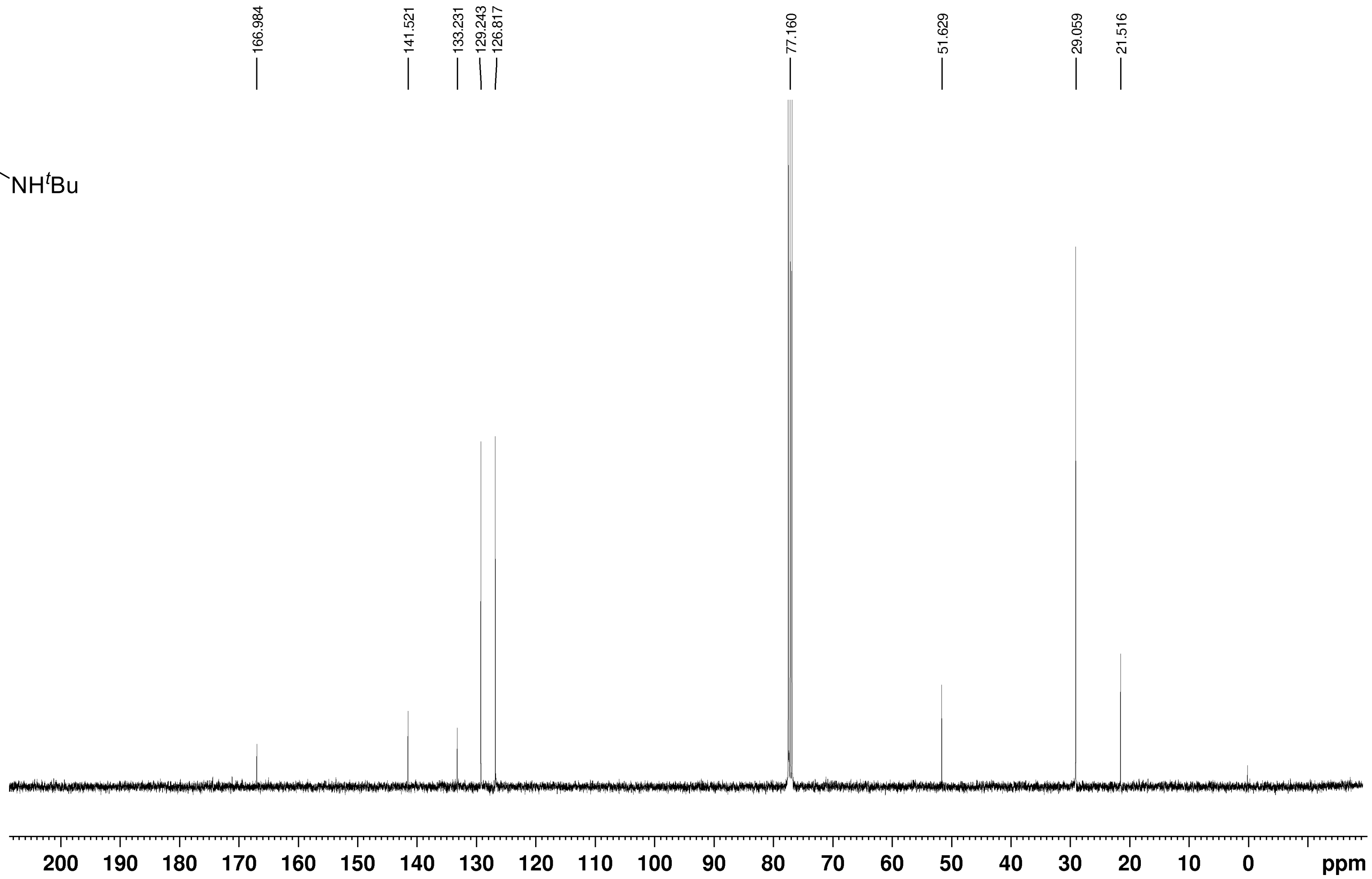

Supplementary Figure 28. ¹H NMR (400 MHz, $CDCl_3$) 4-methoxy-*N*,*N*-dimethylbenzamide (3m)

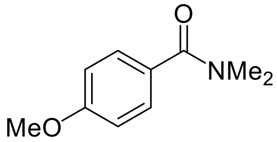

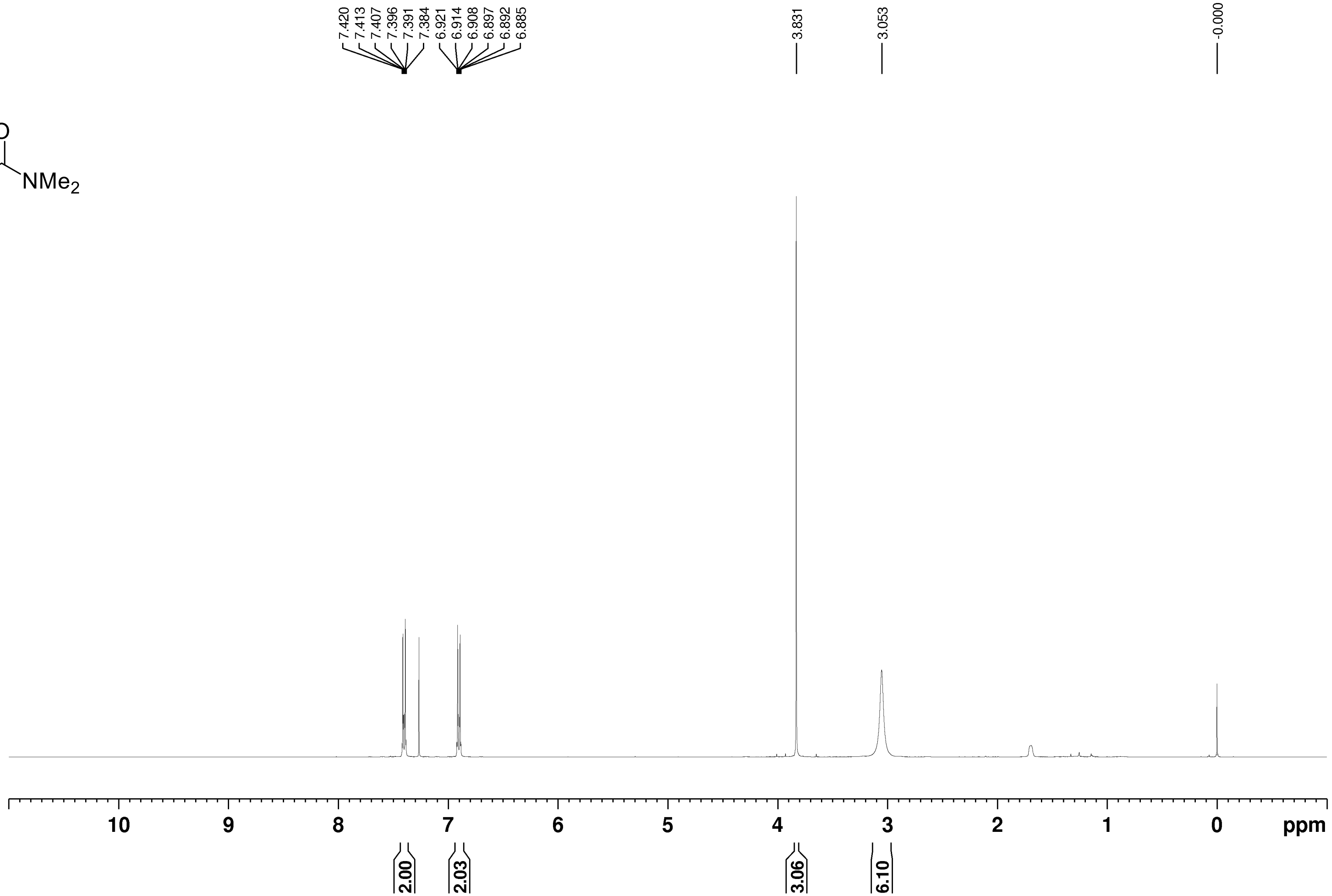

Supplementary Figure 29. ^{13}C **NMR (**$CDCl_3$**, 100 MHz) 4-methoxy-*N*,*N*-dimethylbenzamide (3m)**

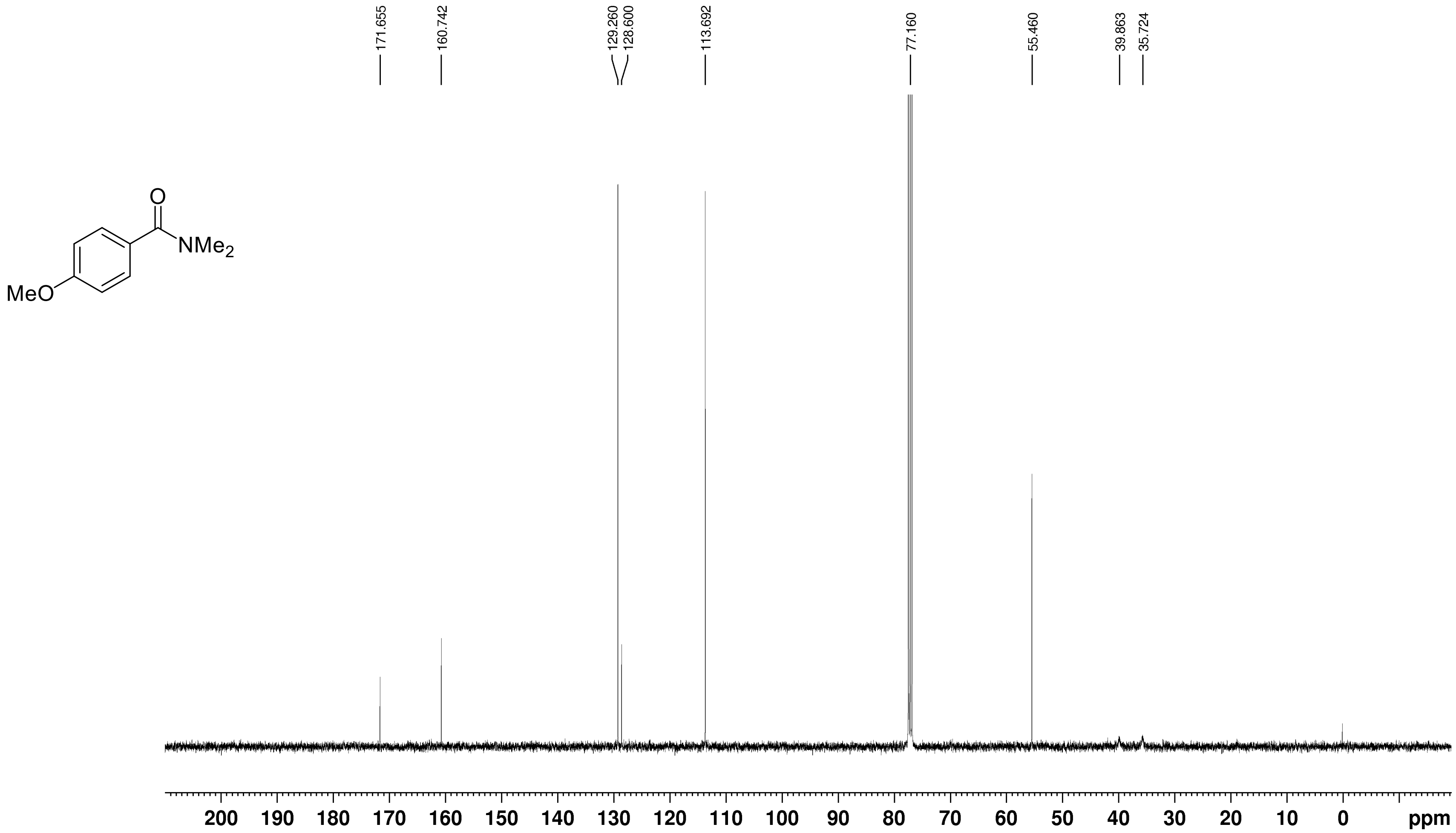

Supplementary Figure 30. ¹H NMR (400 MHz, $CDCl_3$) 3-methoxy-*N*,*N*-dimethylbenzamide (3n)

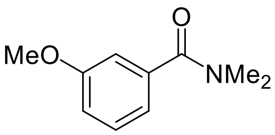

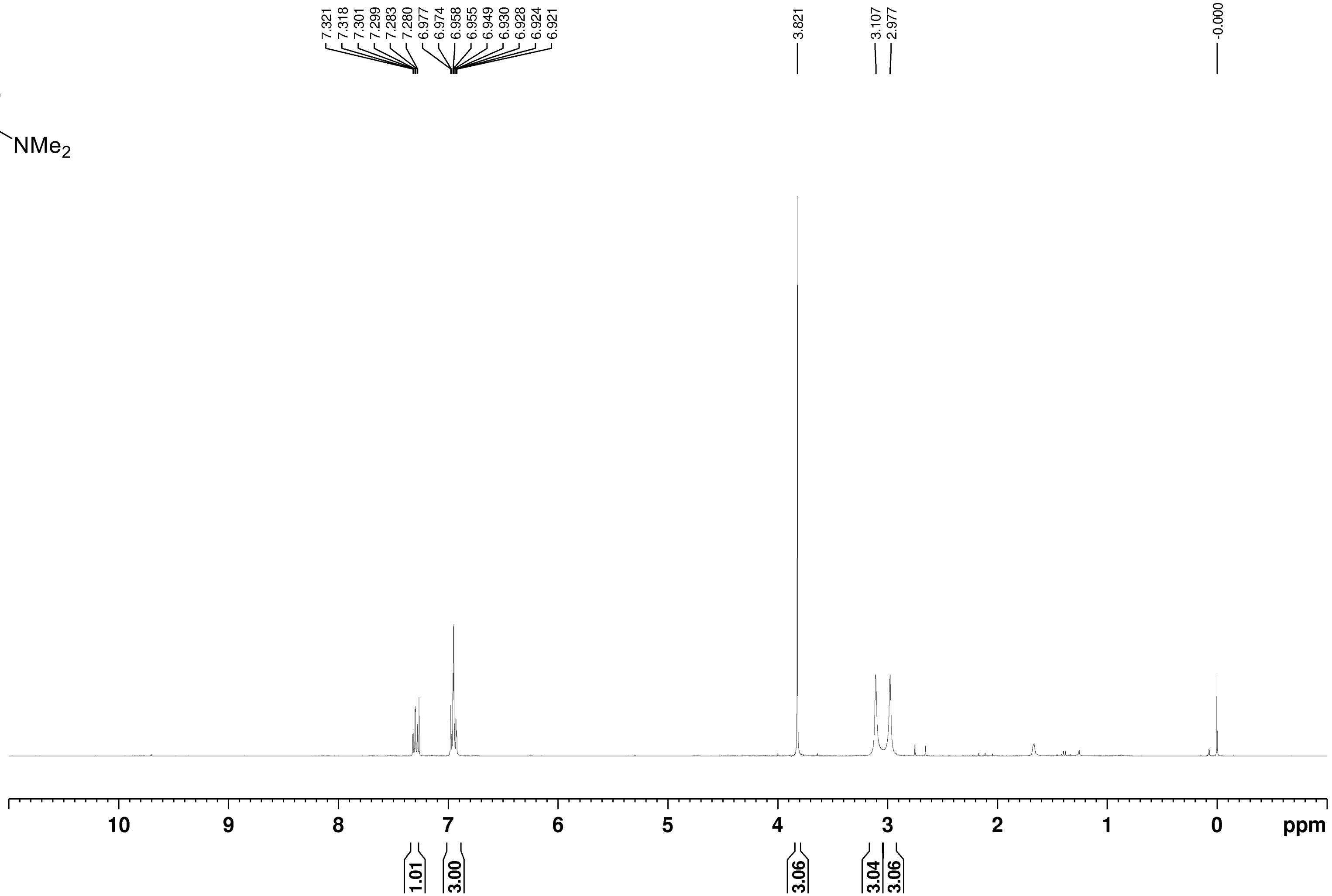

Supplementary Figure 31. ^{13}C NMR ($CDCl_3$, 100 MHz) 3-methoxy-*N*,*N*-dimethylbenzamide (3n)

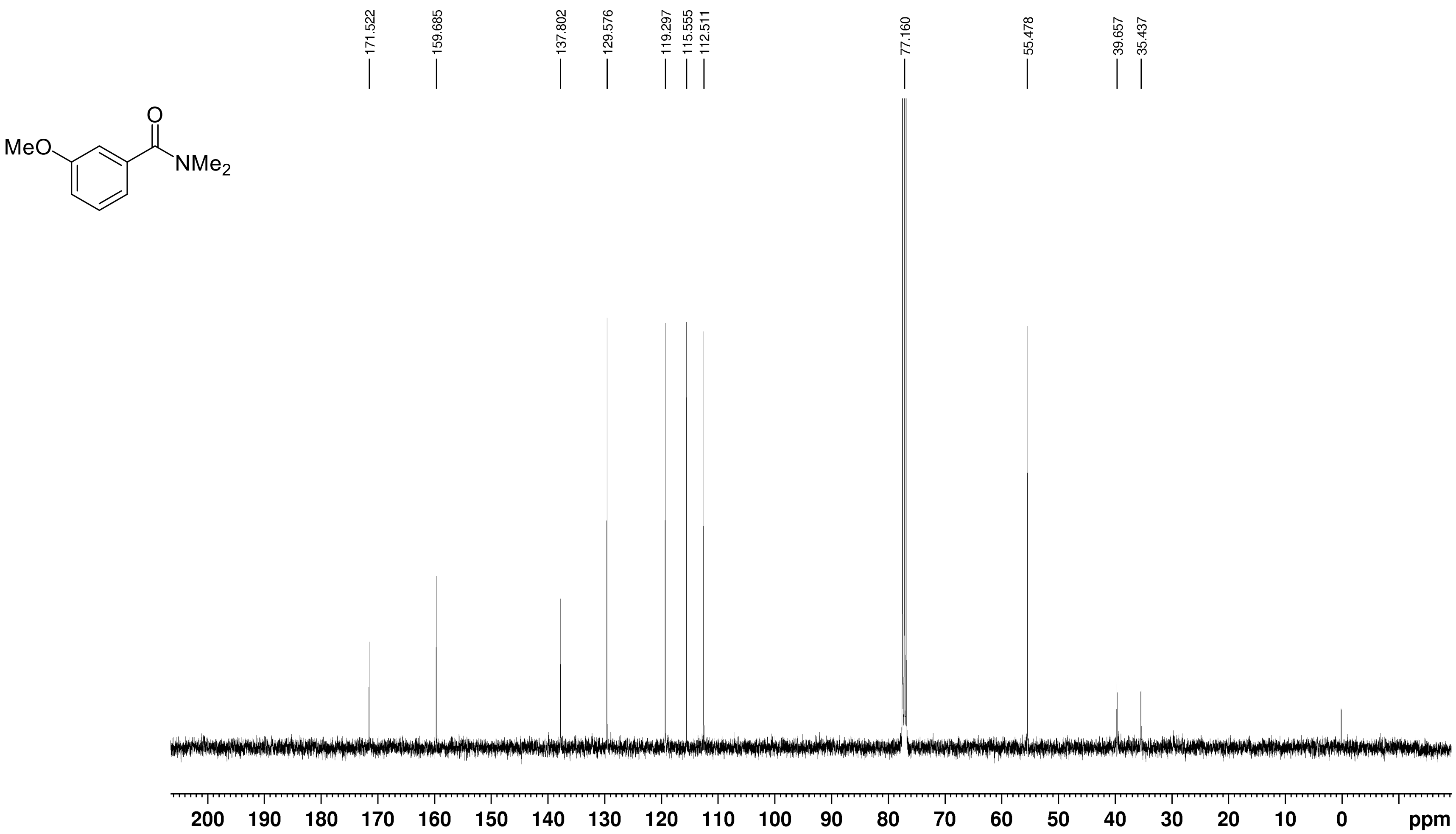

Supplementary Figure 32. ^{1}H NMR (400 MHz, $CDCl_3$) 2-methoxy-*N*,*N*-dimethylbenzamide (3o)

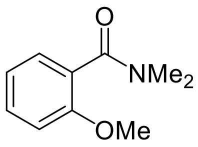

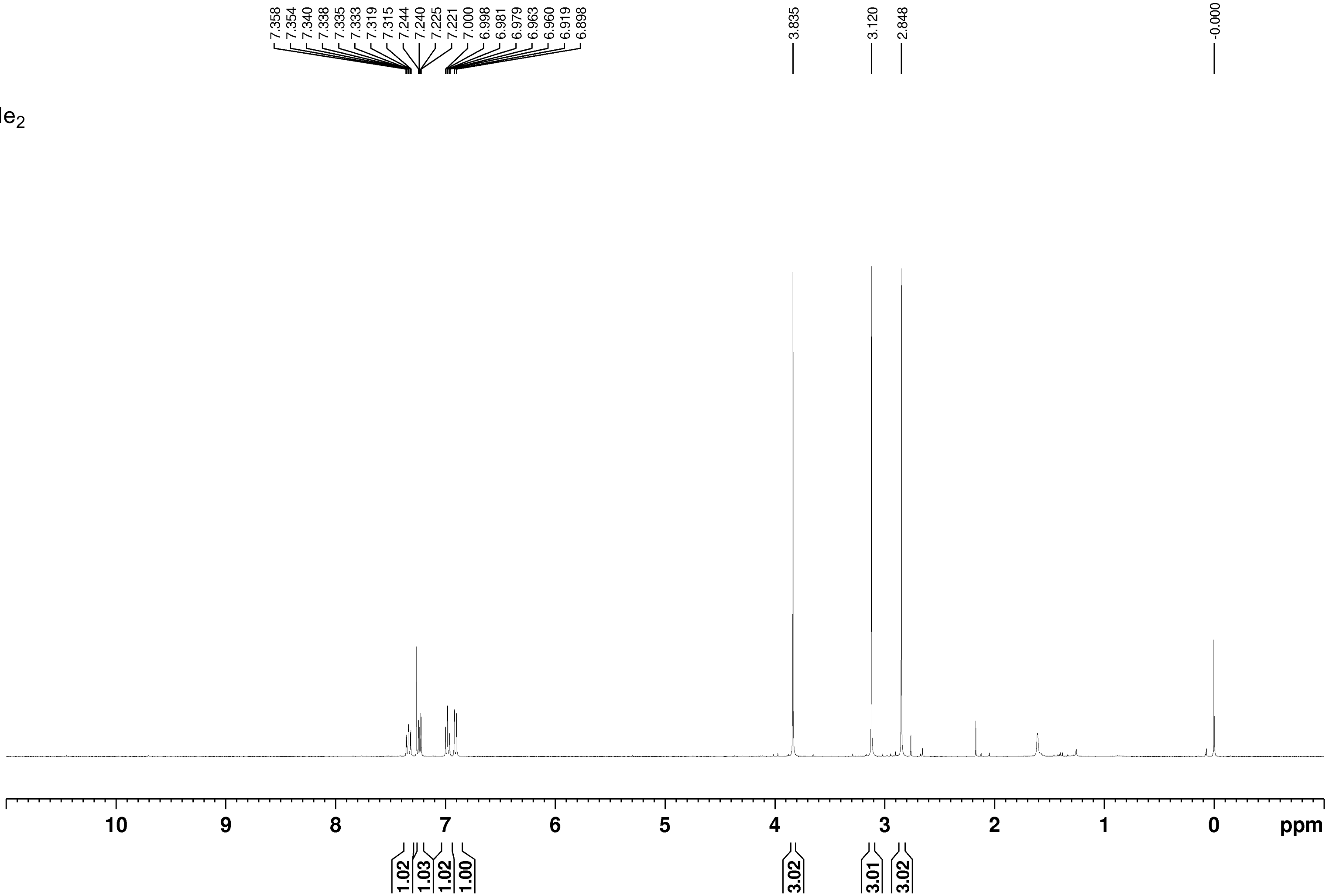

Supplementary Figure 33. ^{13}C NMR ($CDCl_3$, 100 MHz) 2-methoxy-*N*,*N*-dimethylbenzamide (3o)

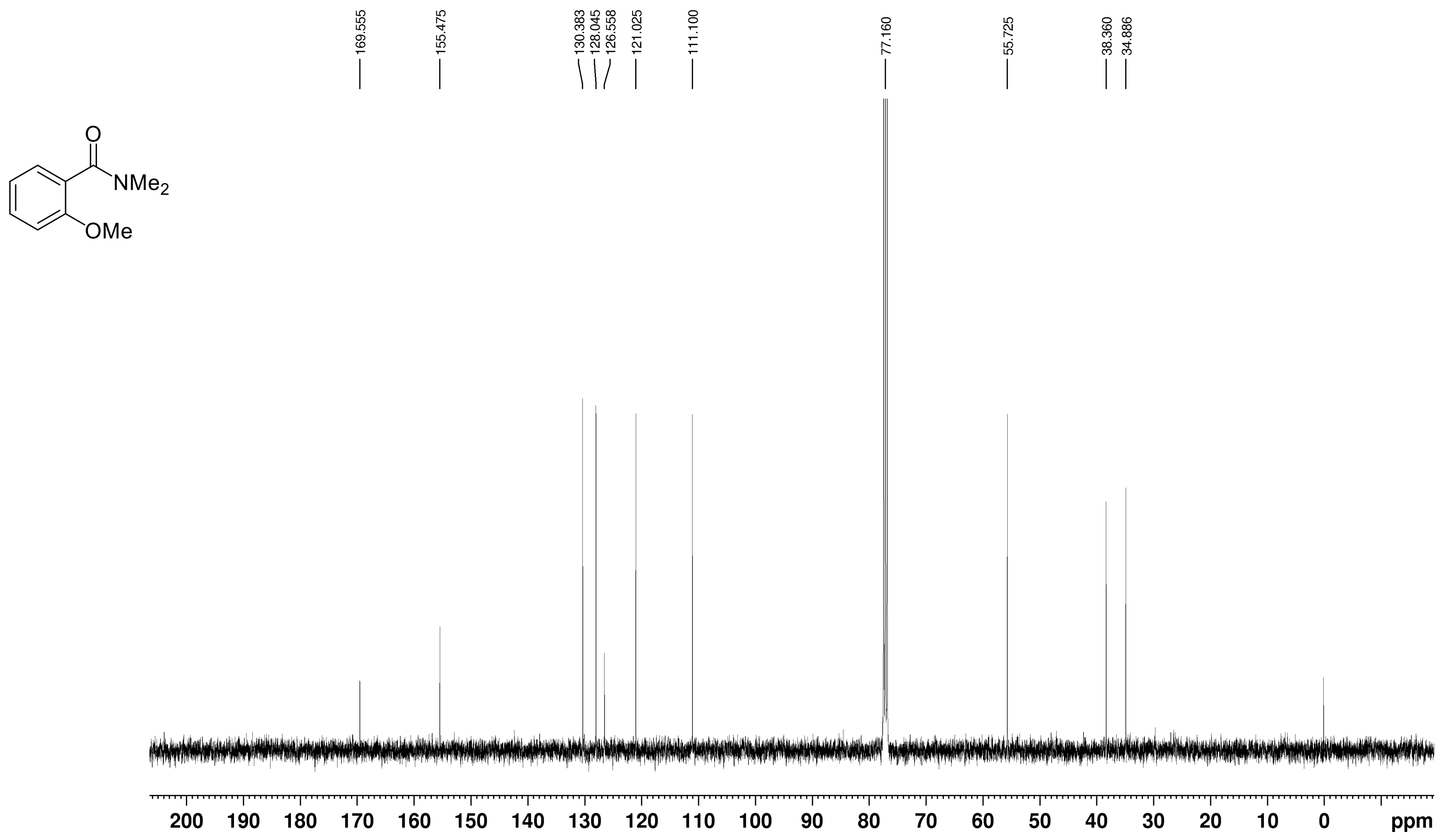

Supplementary Figure 34. ¹H NMR (400 MHz, CDCl$_3$) *N*,*N*-dimethyl-2-naphthamide (3p)

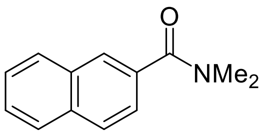

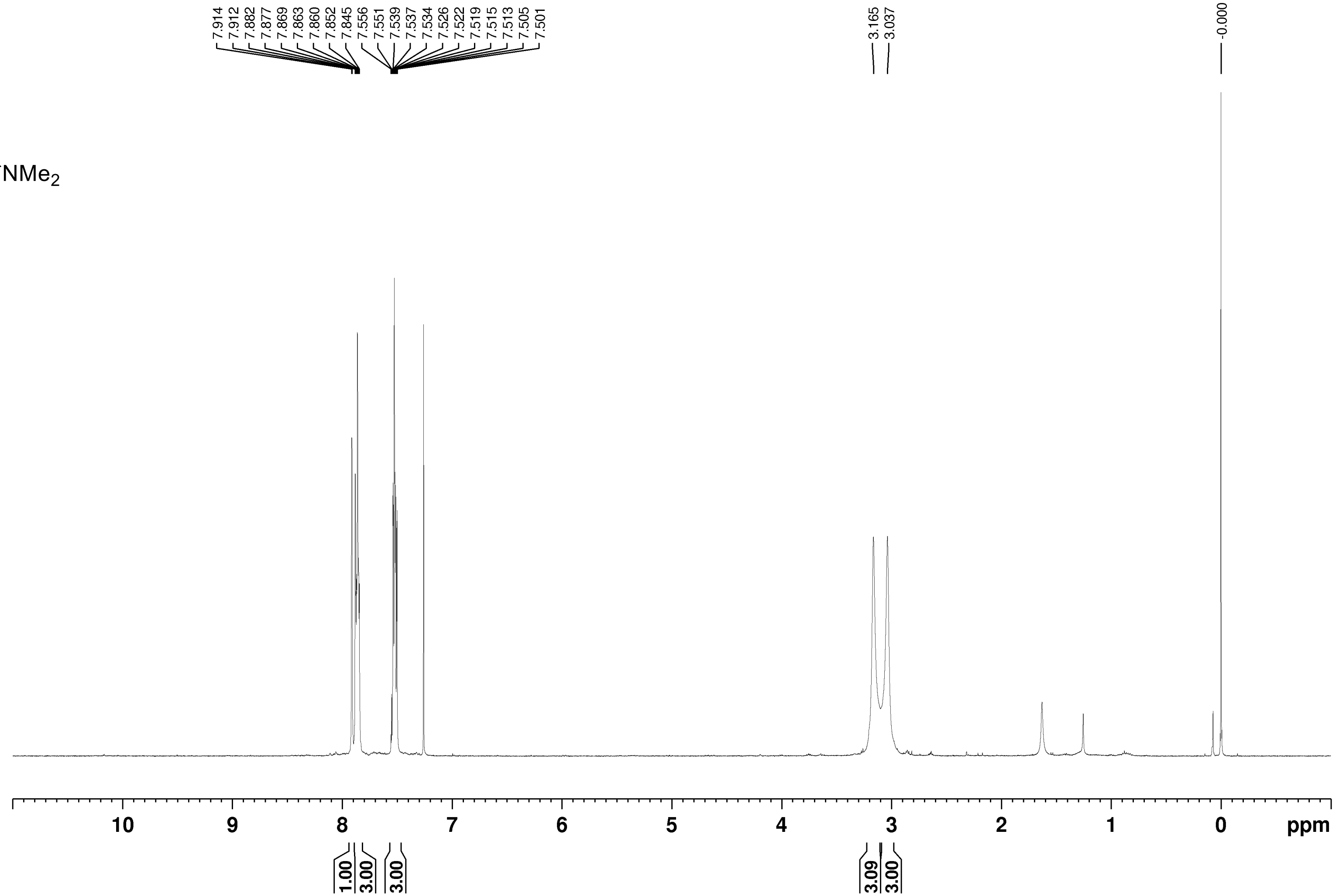

Supplementary Figure 35. ^{13}C NMR ($CDCl_3$, 100 MHz) *N,N*-dimethyl-2-naphthamide (3p)

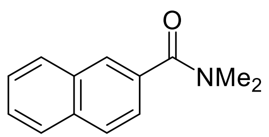

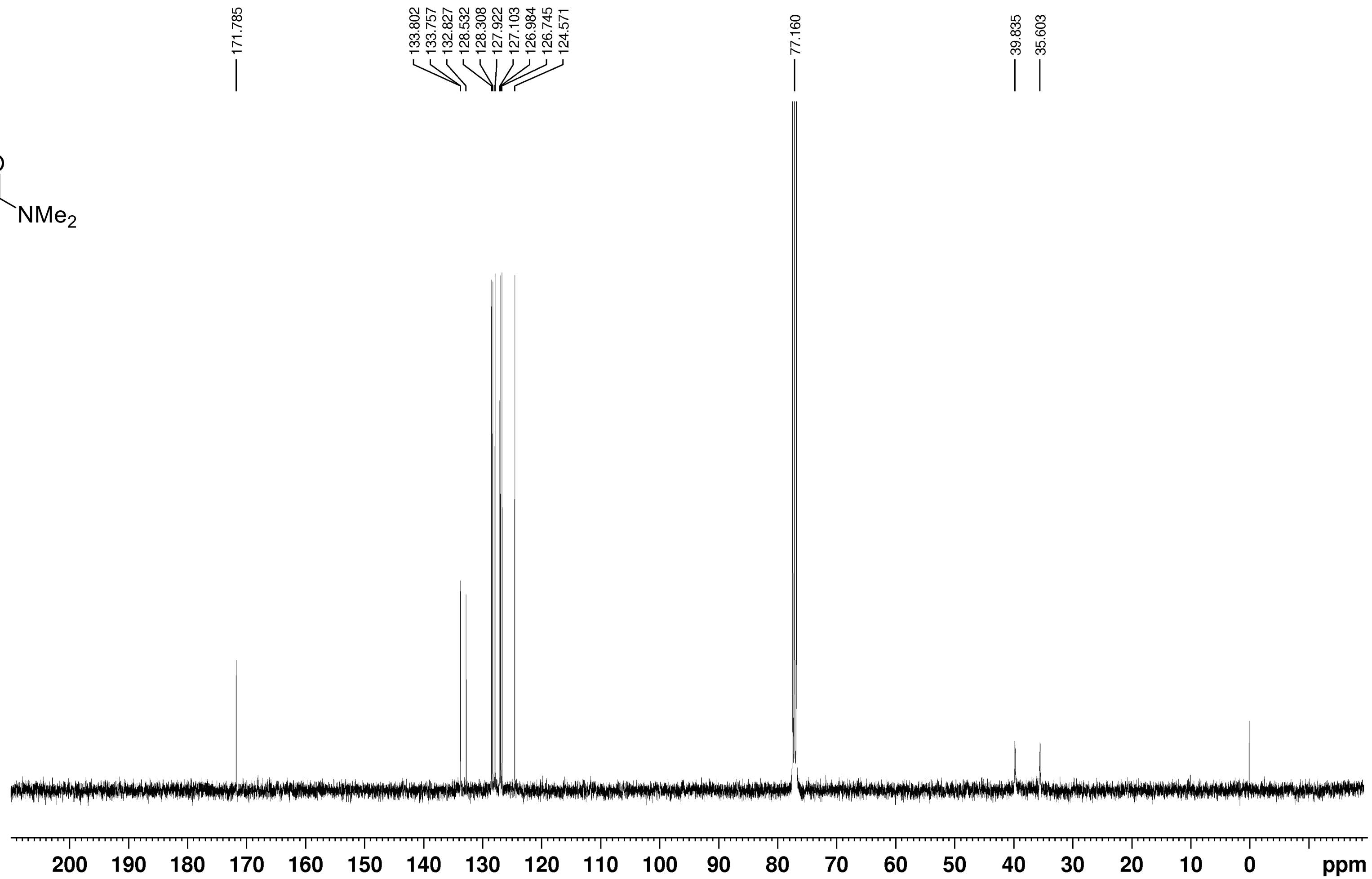

Supplementary Figure 36. ^{1}H NMR (400 MHz, $CDCl_3$) *N*-(*tert*-butyl)thiophene-2-carboxamide (3q)

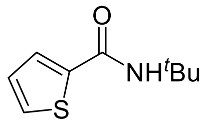

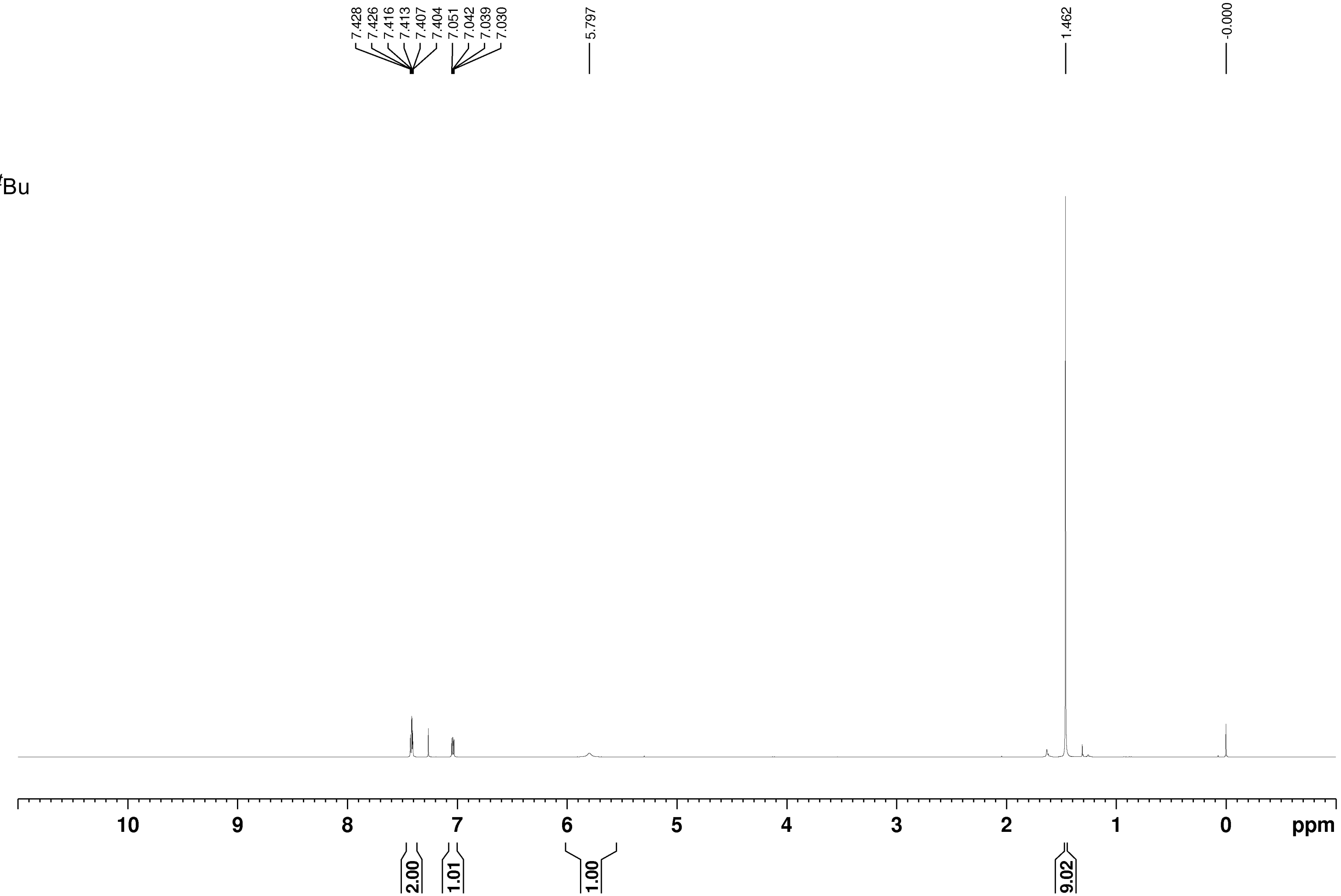

Supplementary Figure 37. ^{13}C NMR ($CDCl_3$, 100 MHz) *N*-(*tert*-butyl)thiophene-2-carboxamide (3q)

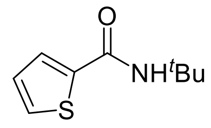

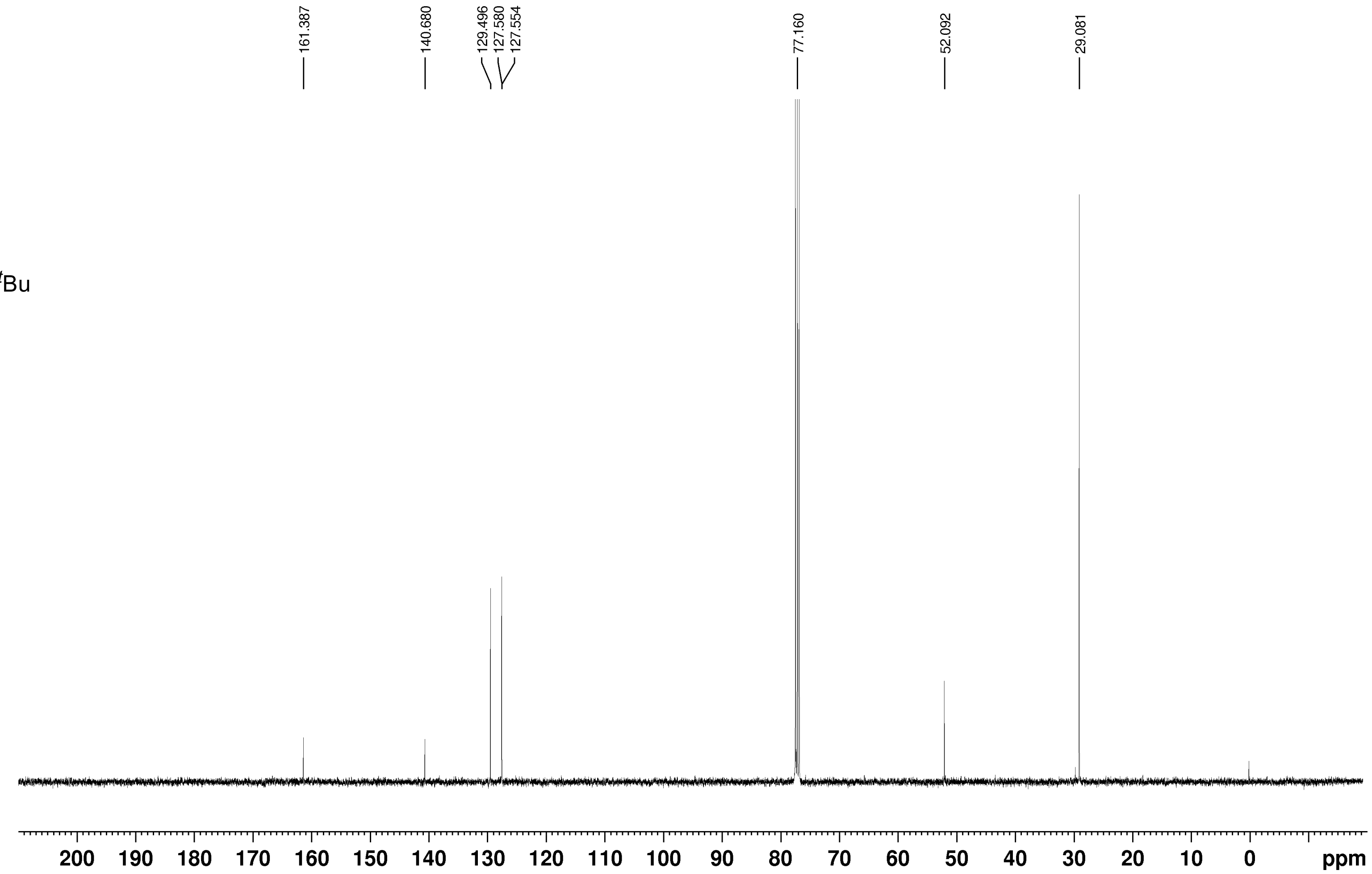

Supplementary Figure 38. ^{1}H NMR (400 MHz, $CDCl_3$) *N*-(*tert*-butyl)thiophene-3-carboxamide (3r)

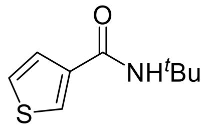

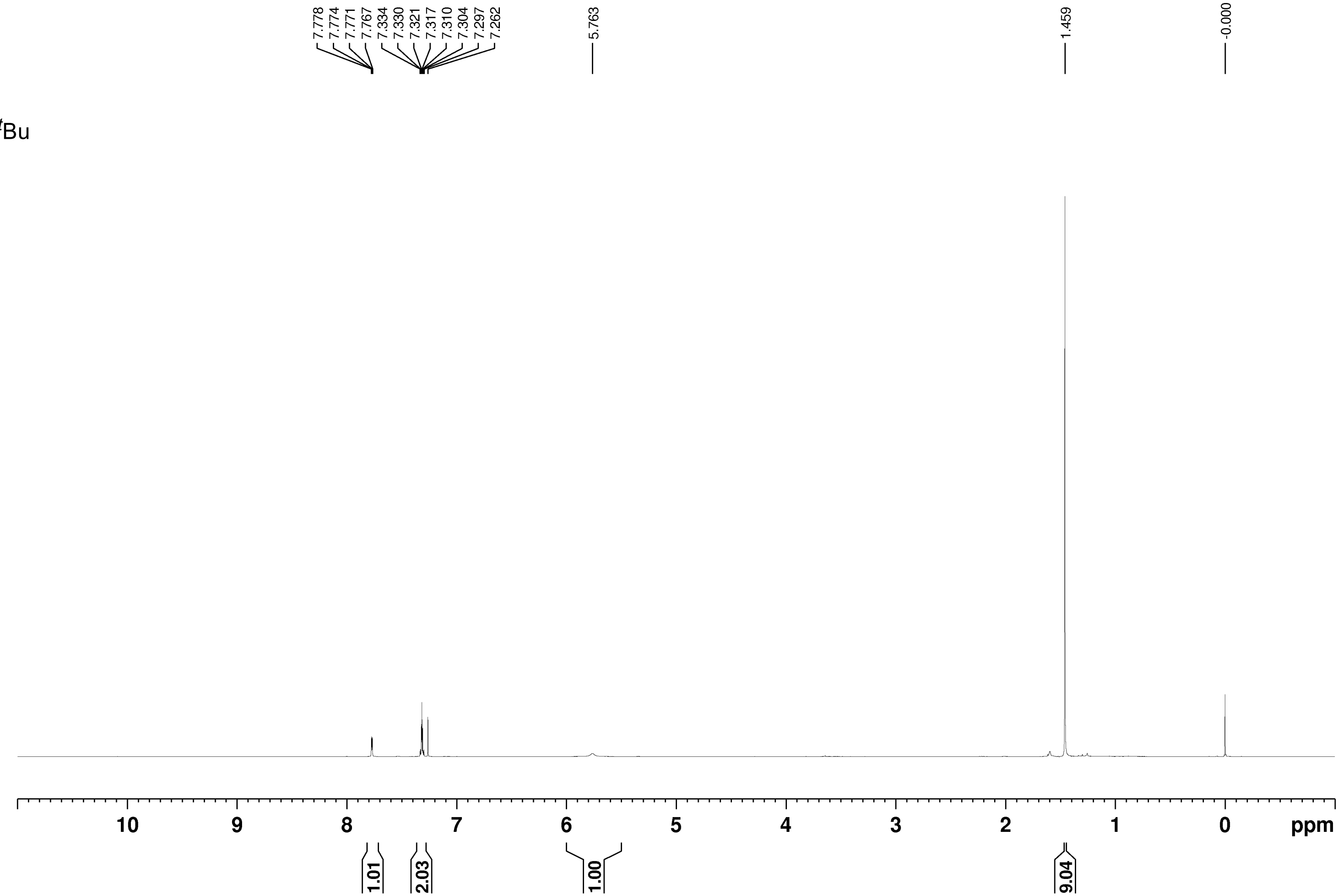

Supplementary Figure 39. ^{13}C **NMR (CDCl$_3$, 100 MHz)** ***N*-(*tert*-butyl)thiophene-3-carboxamide (3r)**

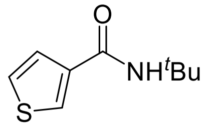

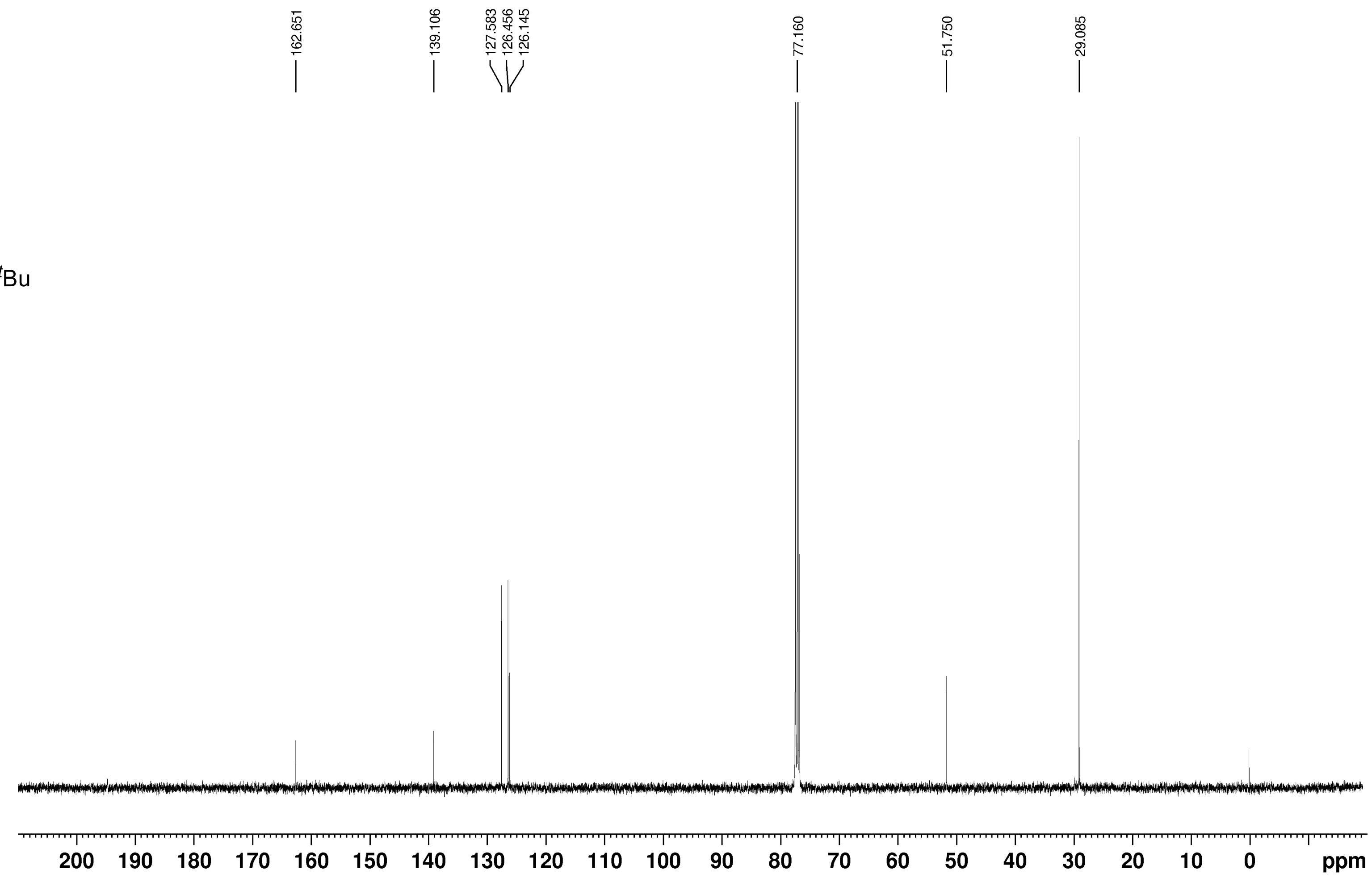

Supplementary Figure 40. ¹H NMR (400 MHz, $CDCl_3$) *N*,*N*-dimethyl-3-phenylpropanamide (3s)

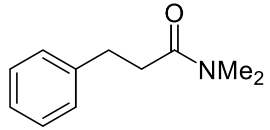

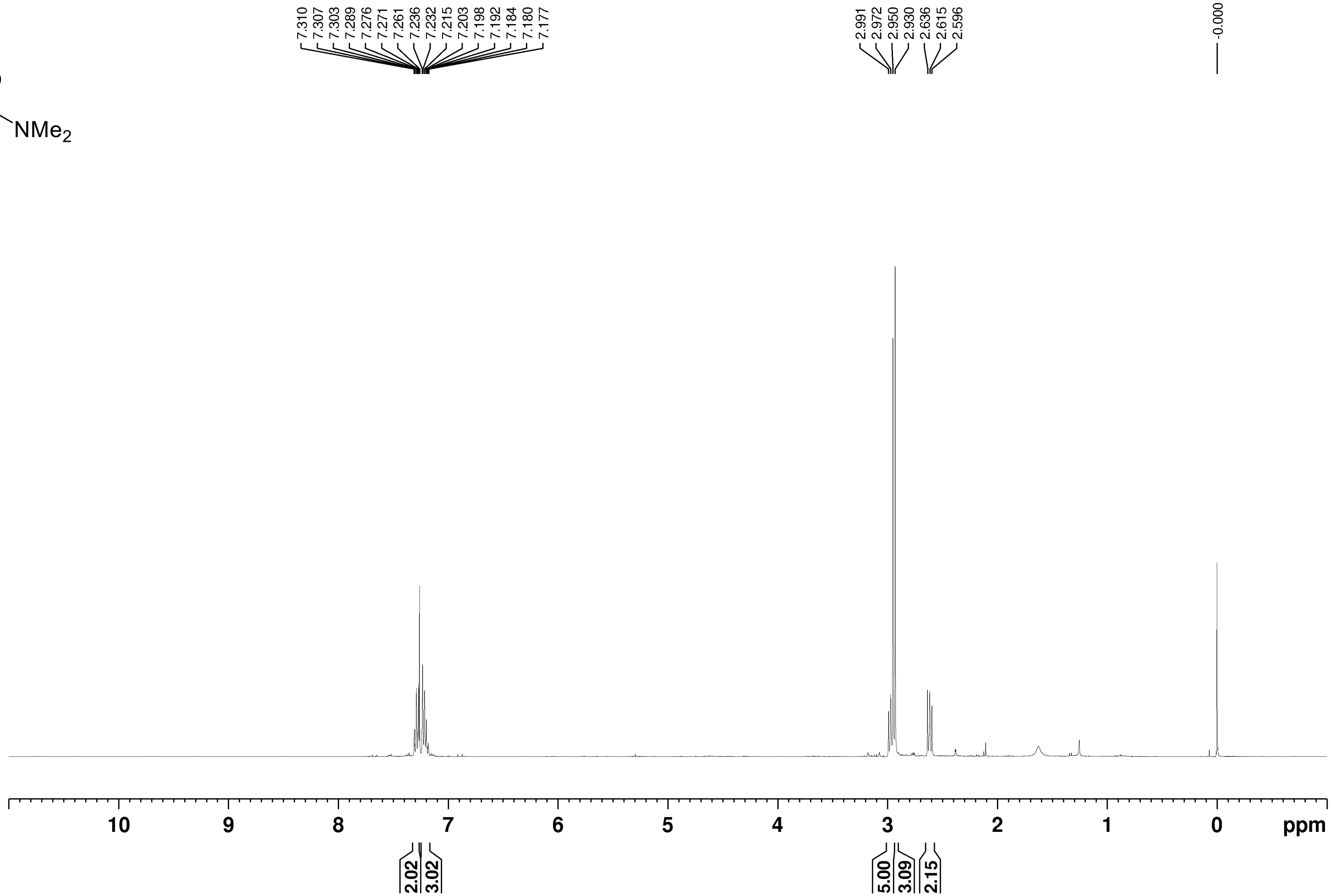

Supplementary Figure 41. ^{13}C NMR ($CDCl_3$, 100 MHz) *N*,*N*-dimethyl-3-phenylpropanamide (3s)

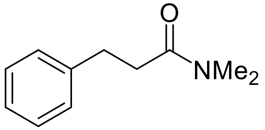

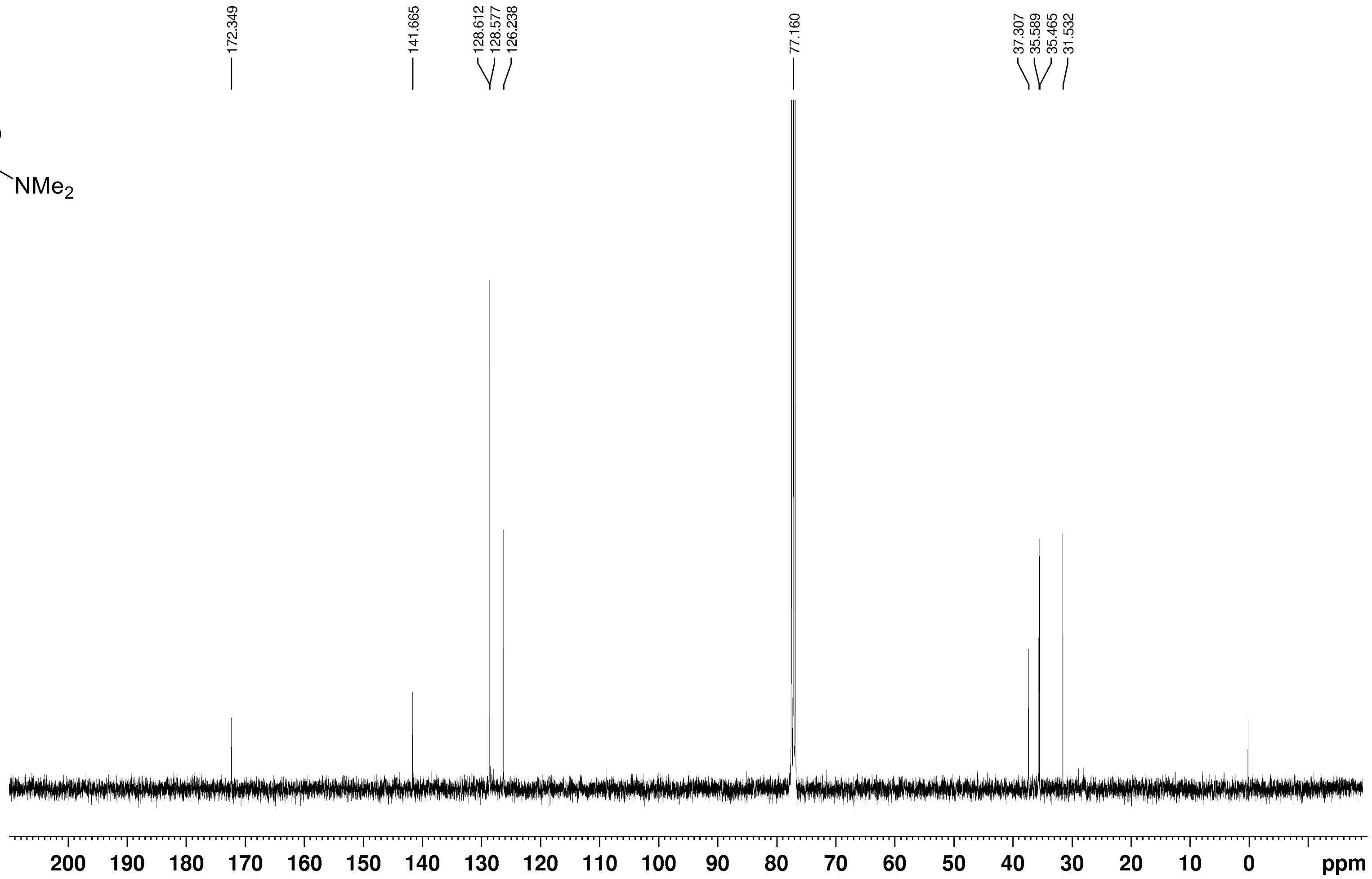

Supplementary Figure 42. **^{1}H NMR (400 MHz, $CDCl_3$) *N*-(*tert*-butyl)cyclopropanecarboxamide (3t)**

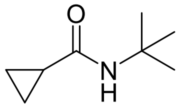

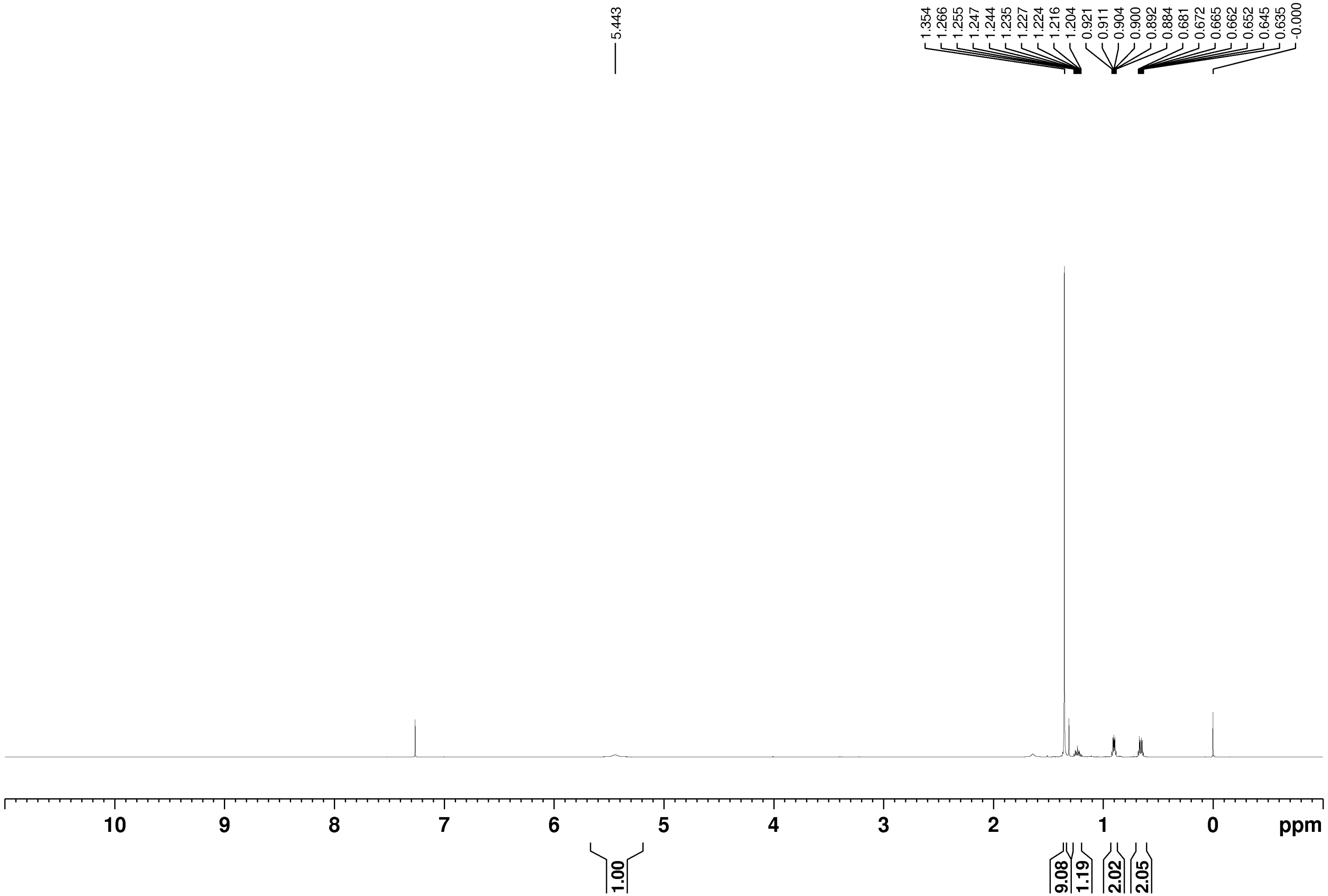

Supplementary Figure 43. ^{13}C NMR ($CDCl_3$, 100 MHz) *N*-(*tert*-butyl)cyclopropanecarboxamide (3t)

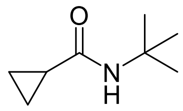

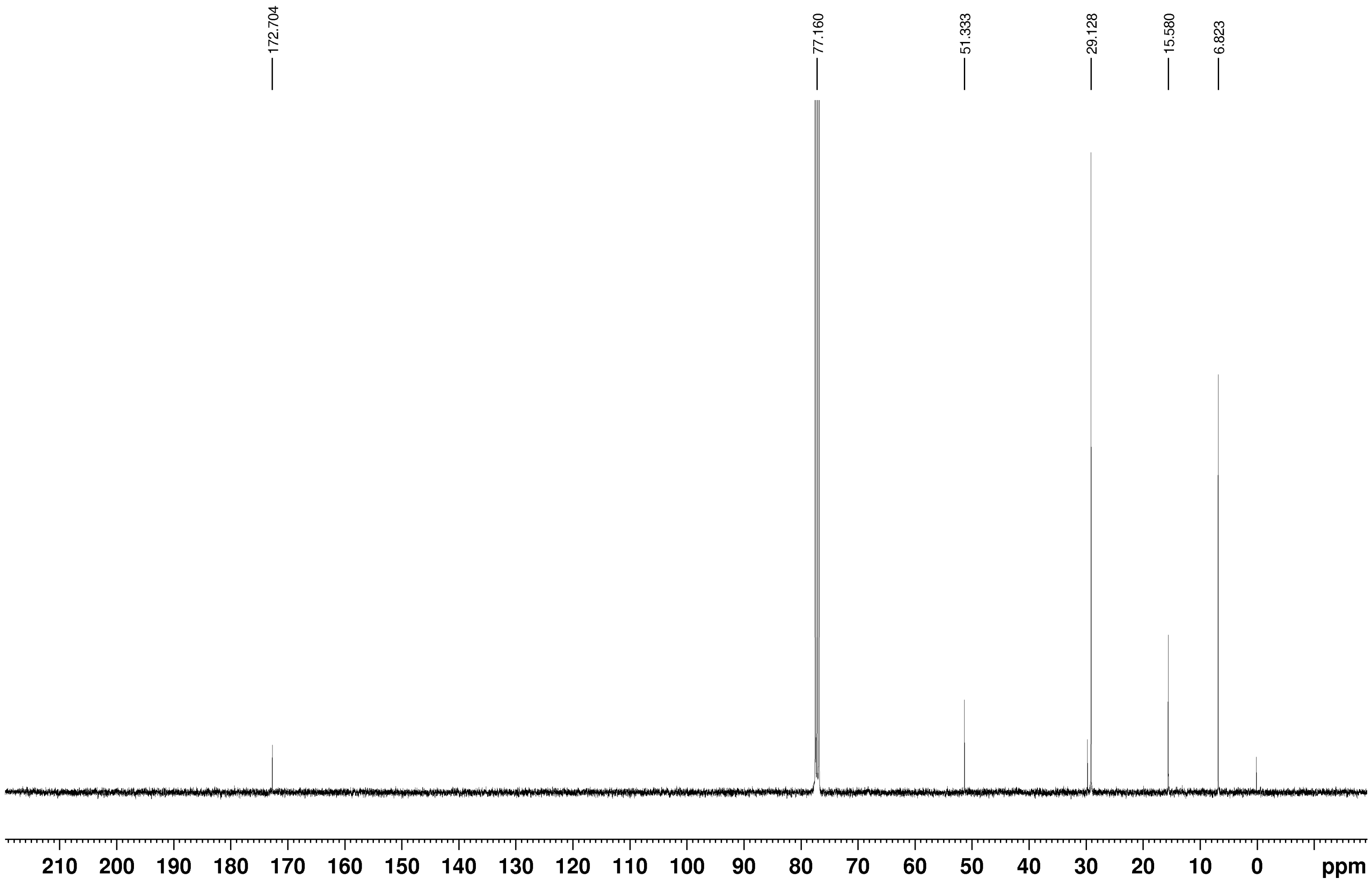

Supplementary Figure 44. ^{1}H NMR (400 MHz, $CDCl_3$) *N*-(*tert*-butyl)cyclohexanecarboxamide (3u)

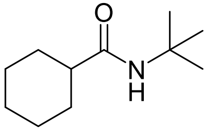

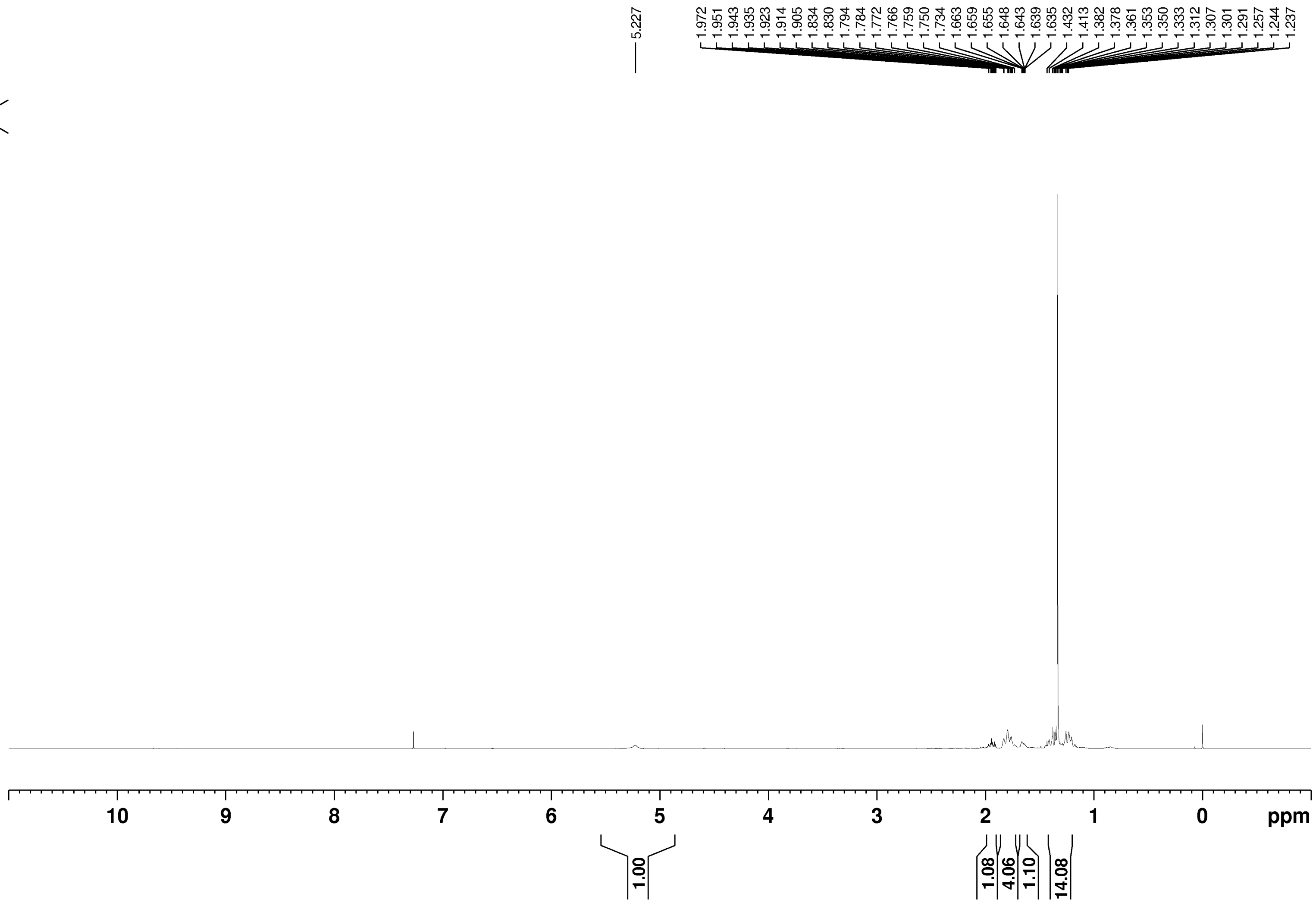

Supplementary Figure 45. ^{13}C NMR ($CDCl_3$, 100 MHz) *N*-(*tert*-butyl)cyclohexanecarboxamide (3u)

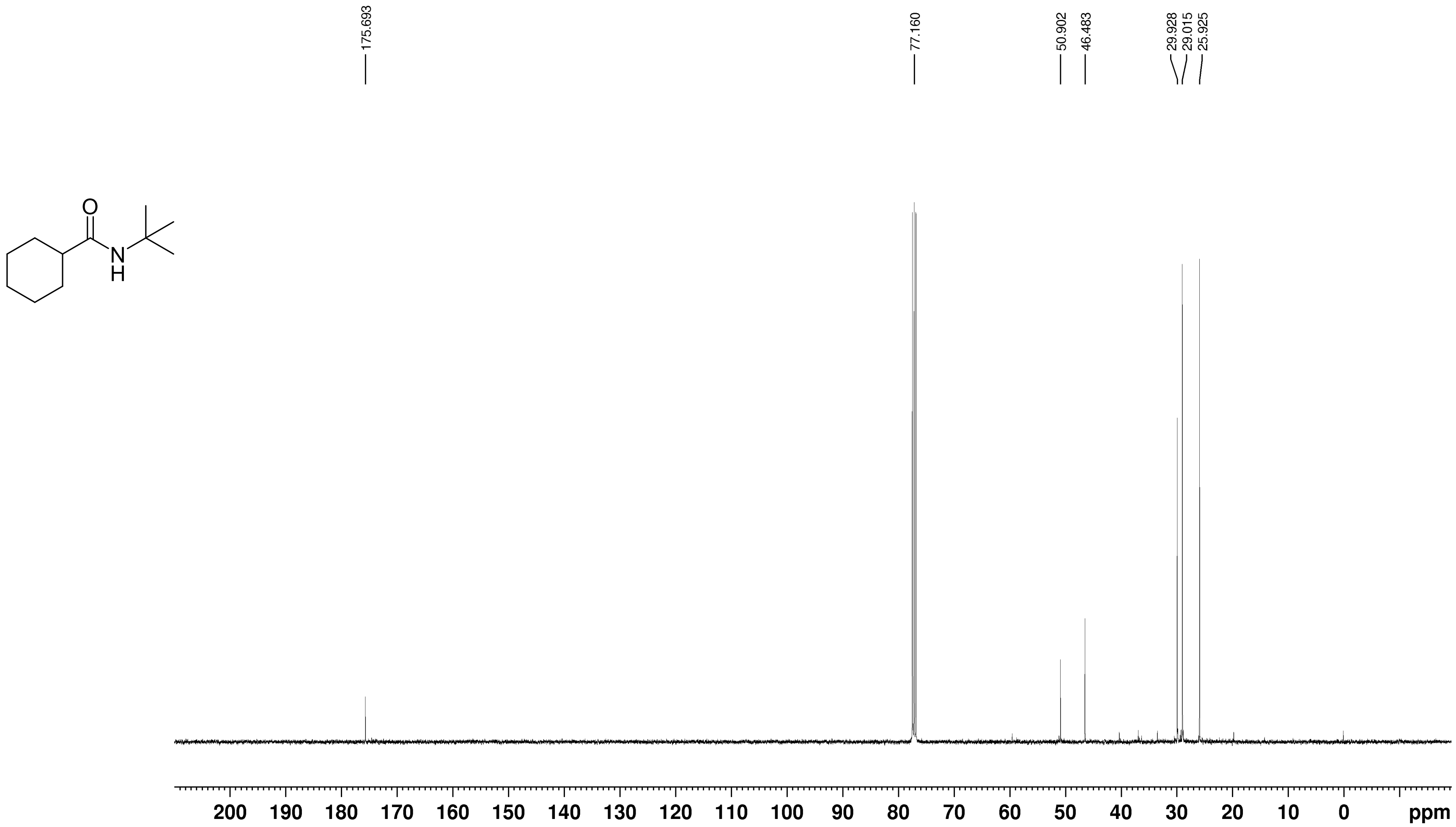

Supplementary Figure 46. ¹H NMR (400 MHz, CDCl$_3$) *N*-(*tert*-butyl)pivalamide (3v)

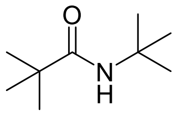

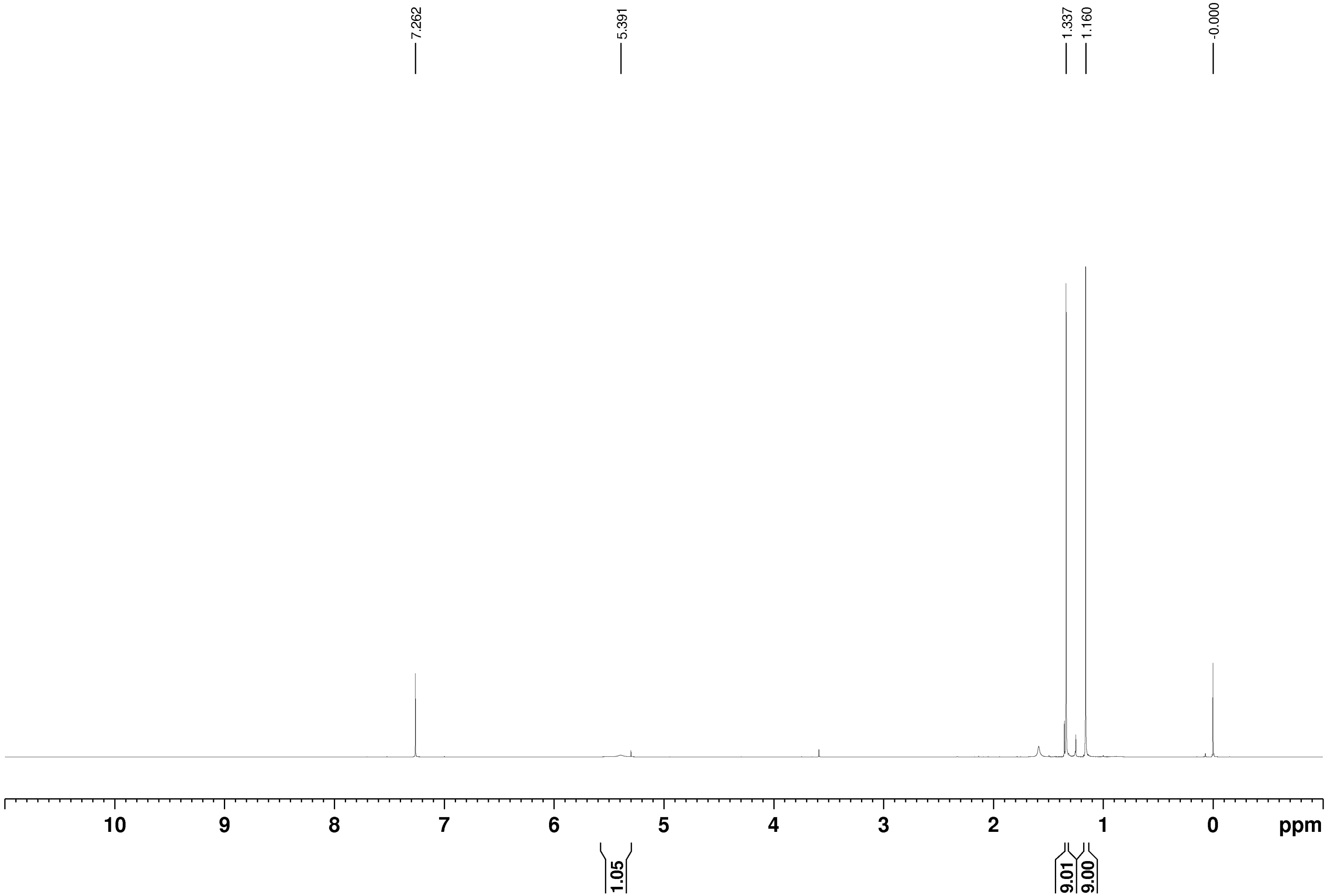

Supplementary Figure 47. ^{13}**C NMR (CDCl$_3$, 100 MHz)** ***N*-(*tert*-butyl)pivalamide (3v)**

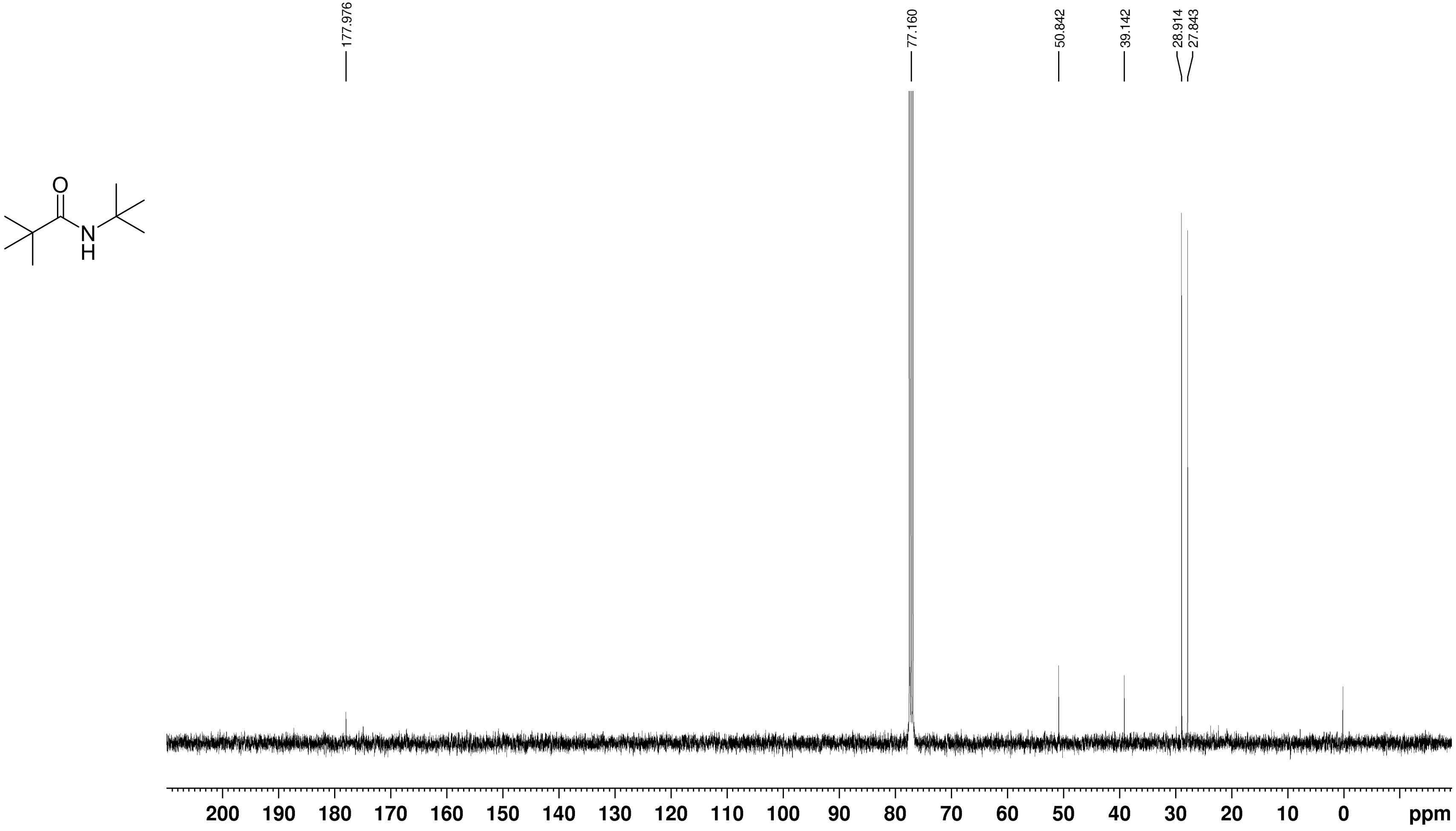

Supplementary Figure 48. ^{1}H NMR (400 MHz, $CDCl_3$) *N-tert*-Butylbenzamide (3ab)

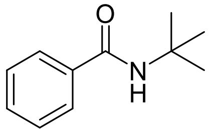

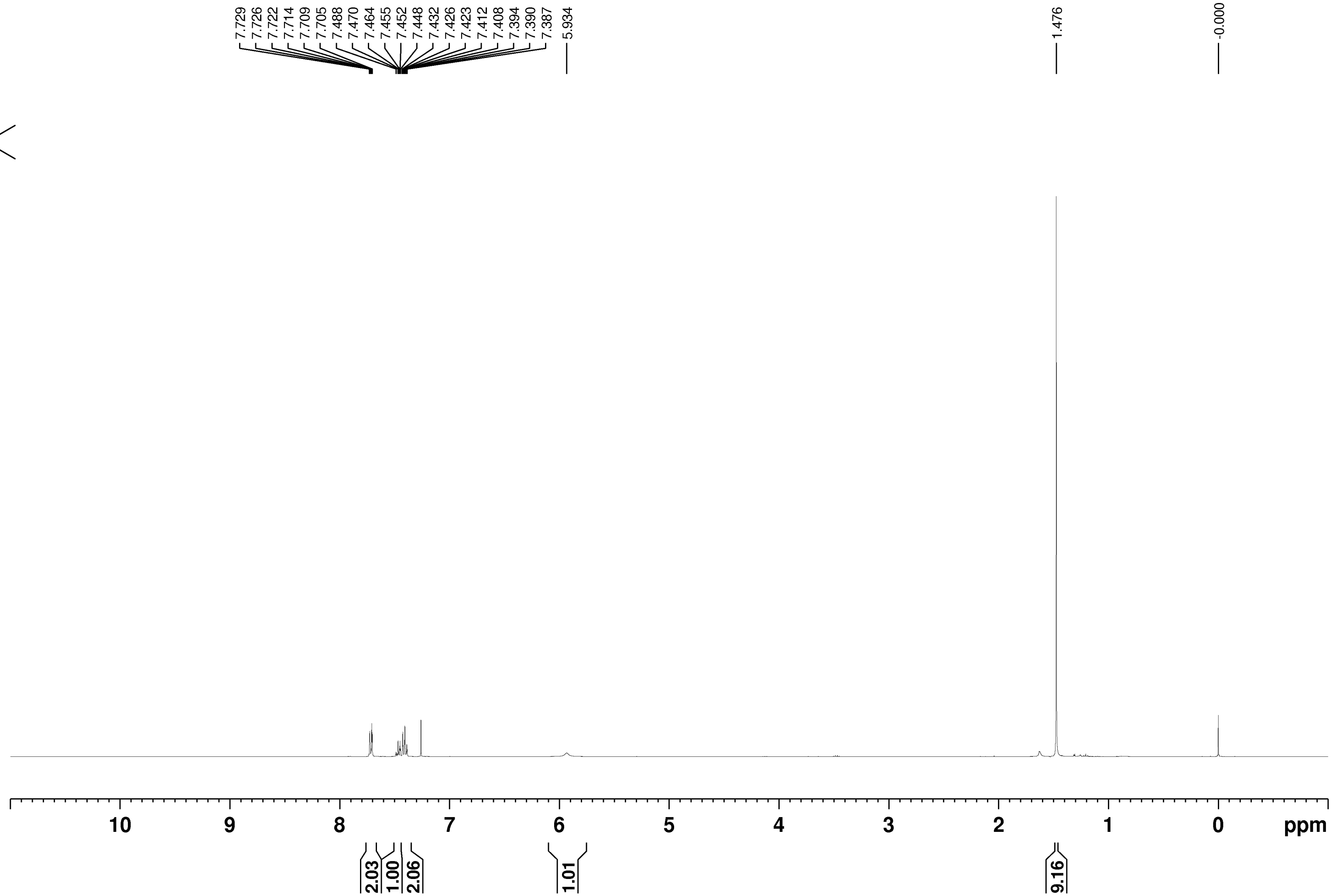

Supplementary Figure 49. ^{13}C NMR ($CDCl_3$, 100 MHz) *N-tert*-Butylbenzamide (3ab)

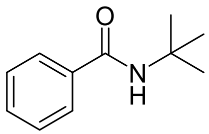

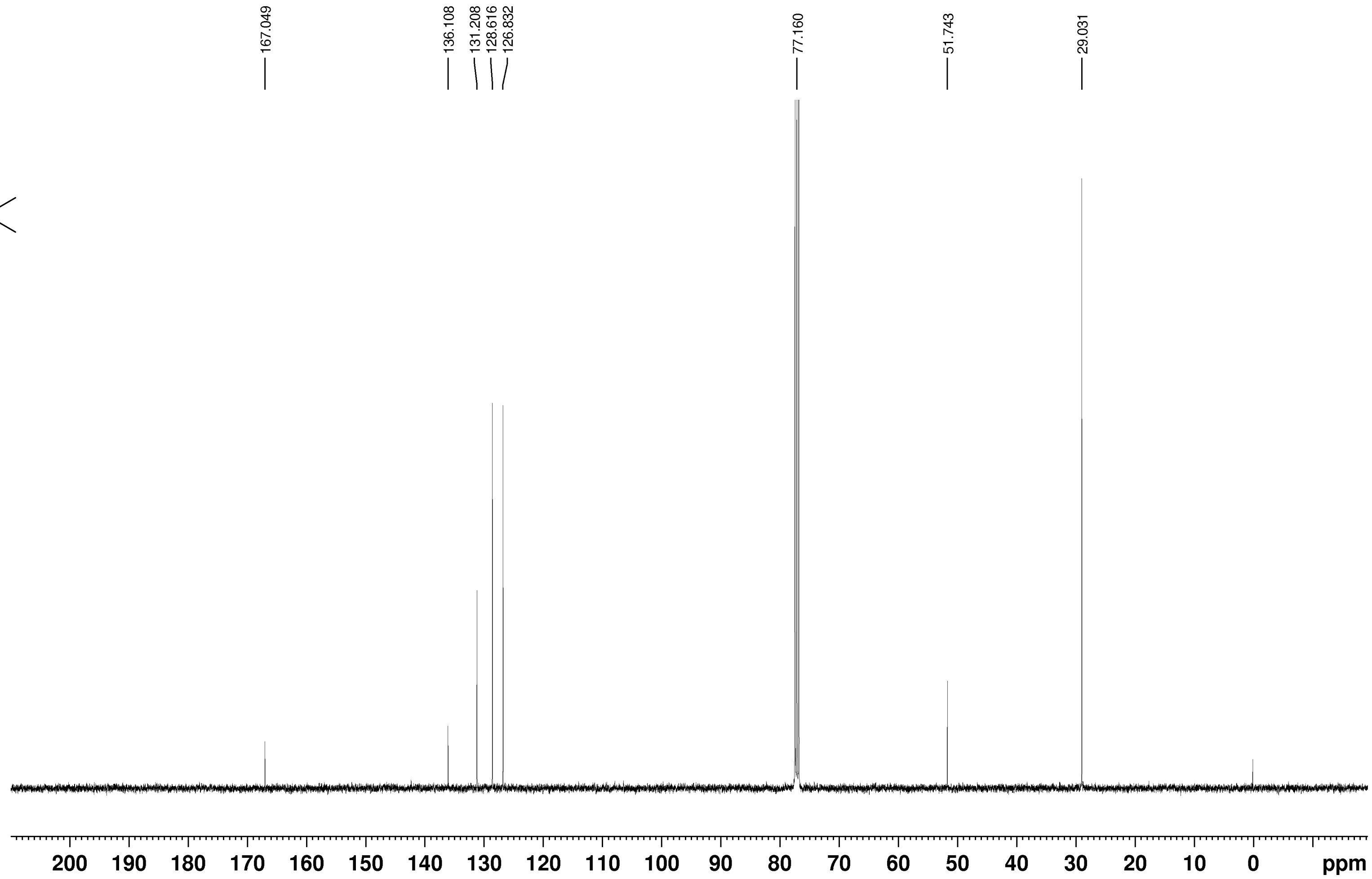

Supplementary Figure 50. ¹H NMR (400 MHz, $CDCl_3$) *N*-propylbenzamide (3ac)

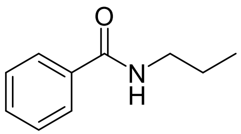

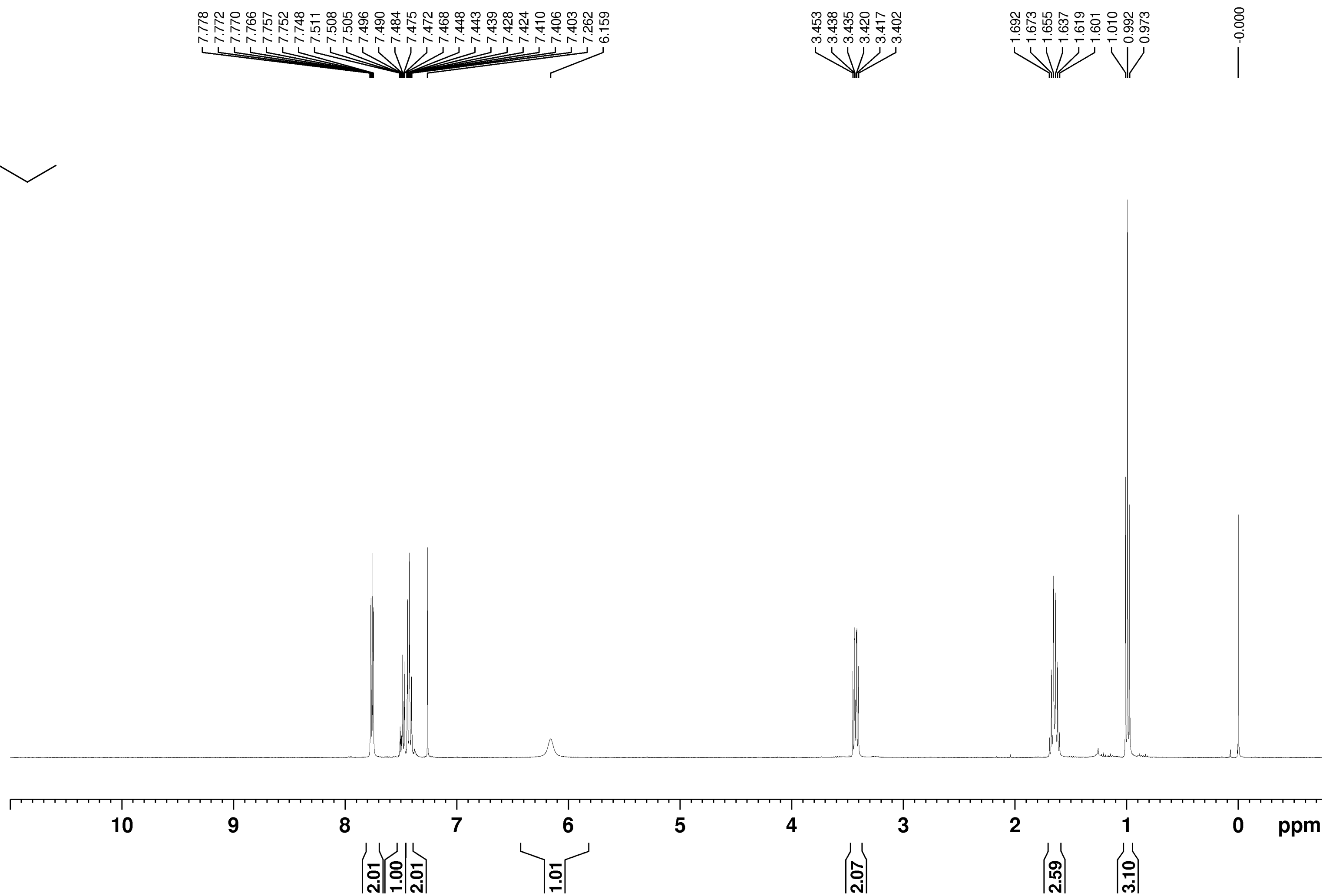

Supplementary Figure 51. ^{13}C NMR ($CDCl_3$, 100 MHz) *N*-propylbenzamide (3ac)

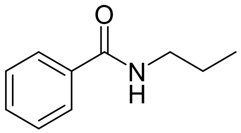

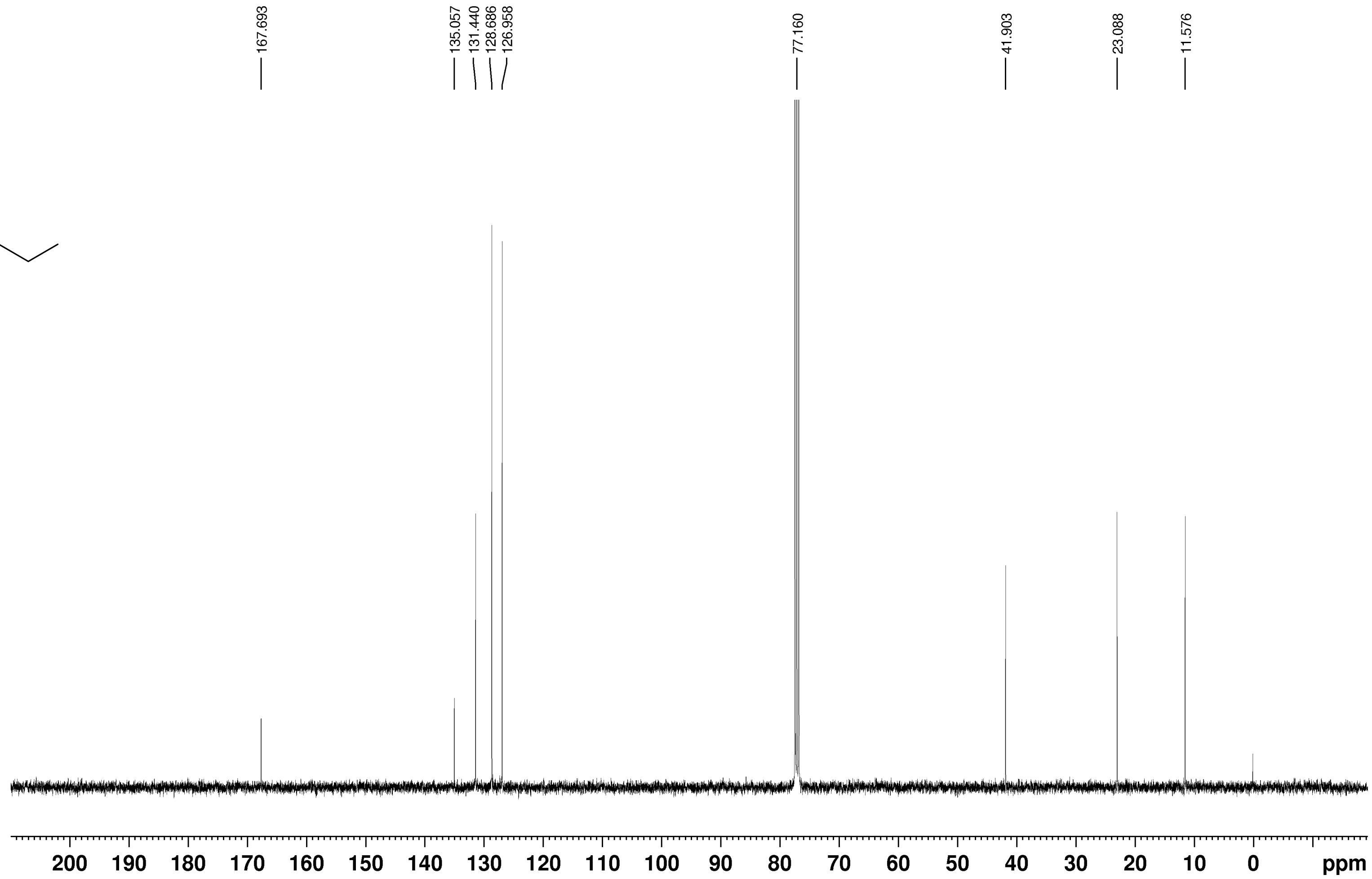

Supplementary Figure 52. ^{1}H NMR (400 MHz, $CDCl_3$) *N*-benzylbenzamide (3ad)

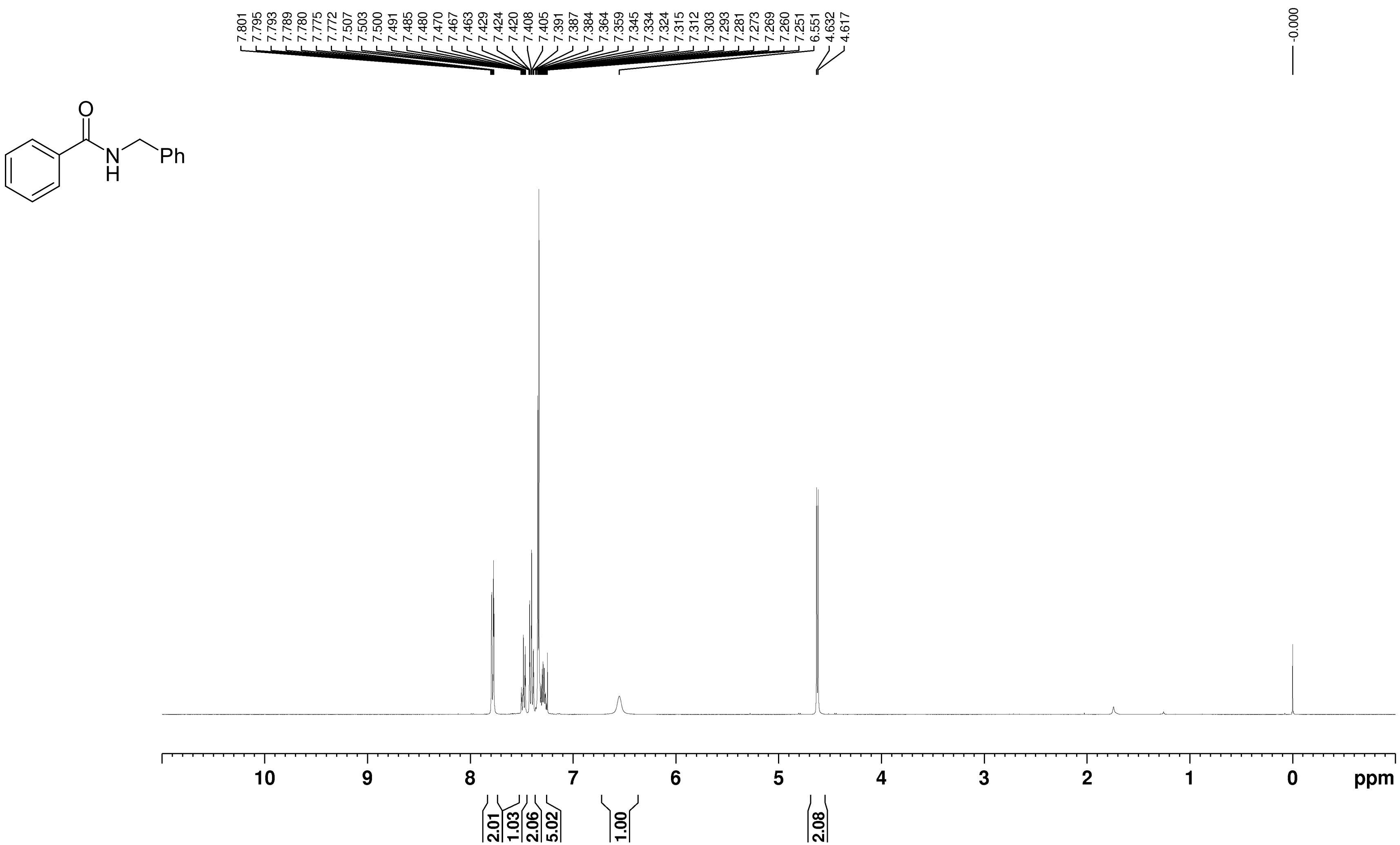

Supplementary Figure 53. ^{13}C NMR ($CDCl_3$, 100 MHz) *N*-benzylbenzamide (3ad)

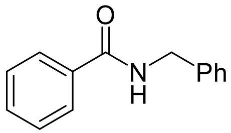

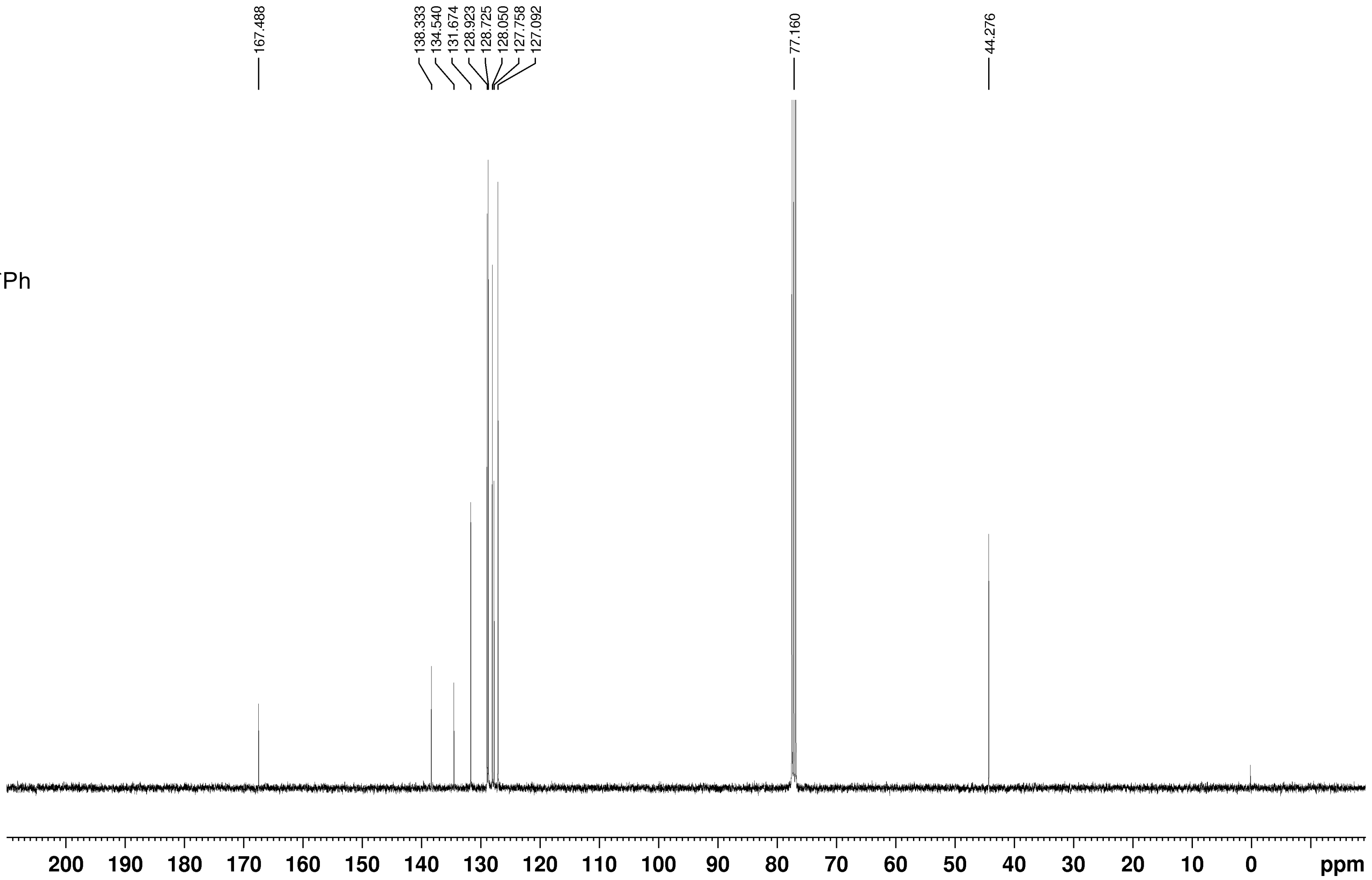

Supplementary Figure 54. ^{1}H NMR (400 MHz, $CDCl_3$) *N*-benzoylmorpholine (3ae)

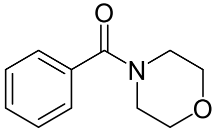

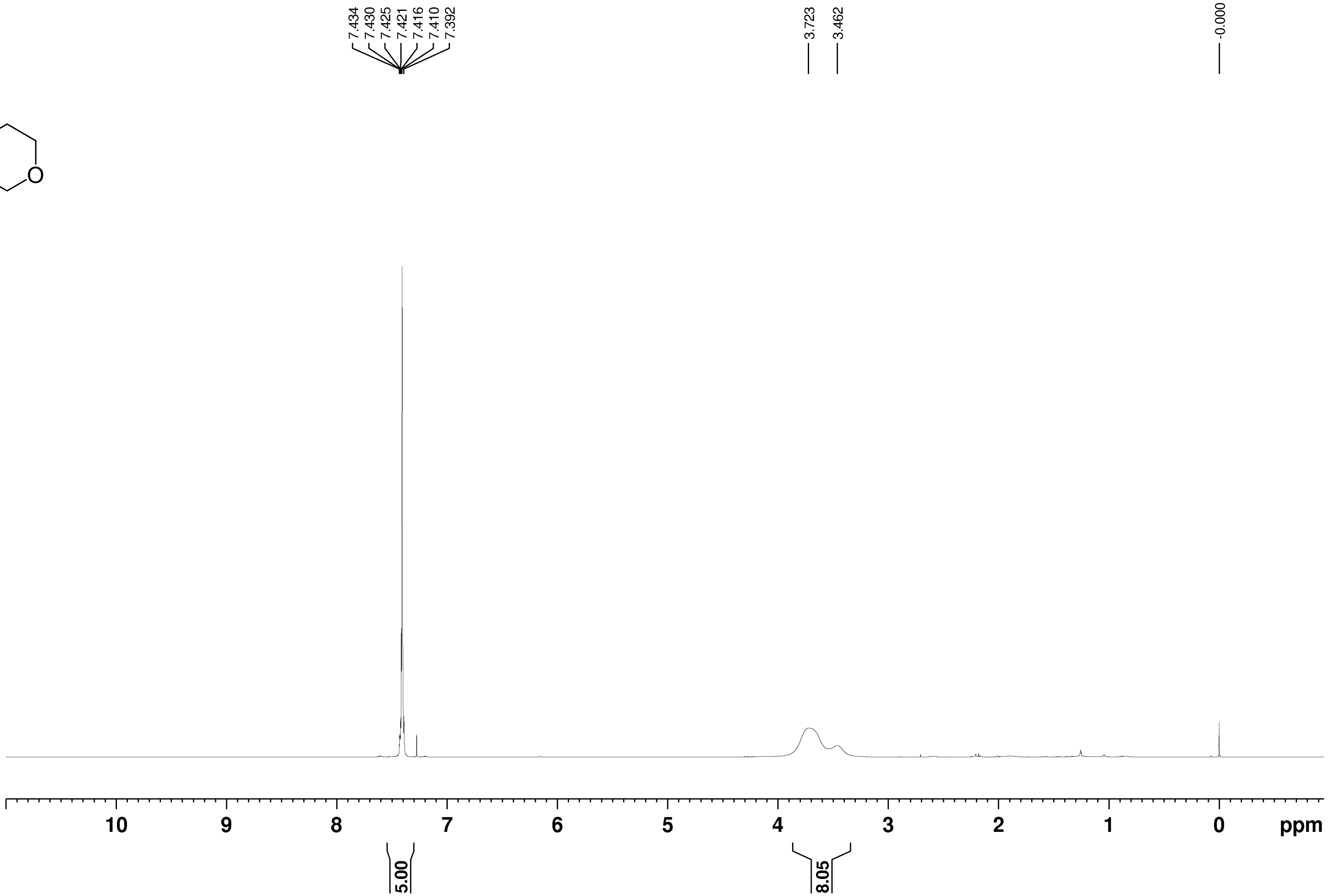

Supplementary Figure 55. ^{13}C NMR ($CDCl_3$, 100 MHz) *N*-benzoylmorpholine (3ae)

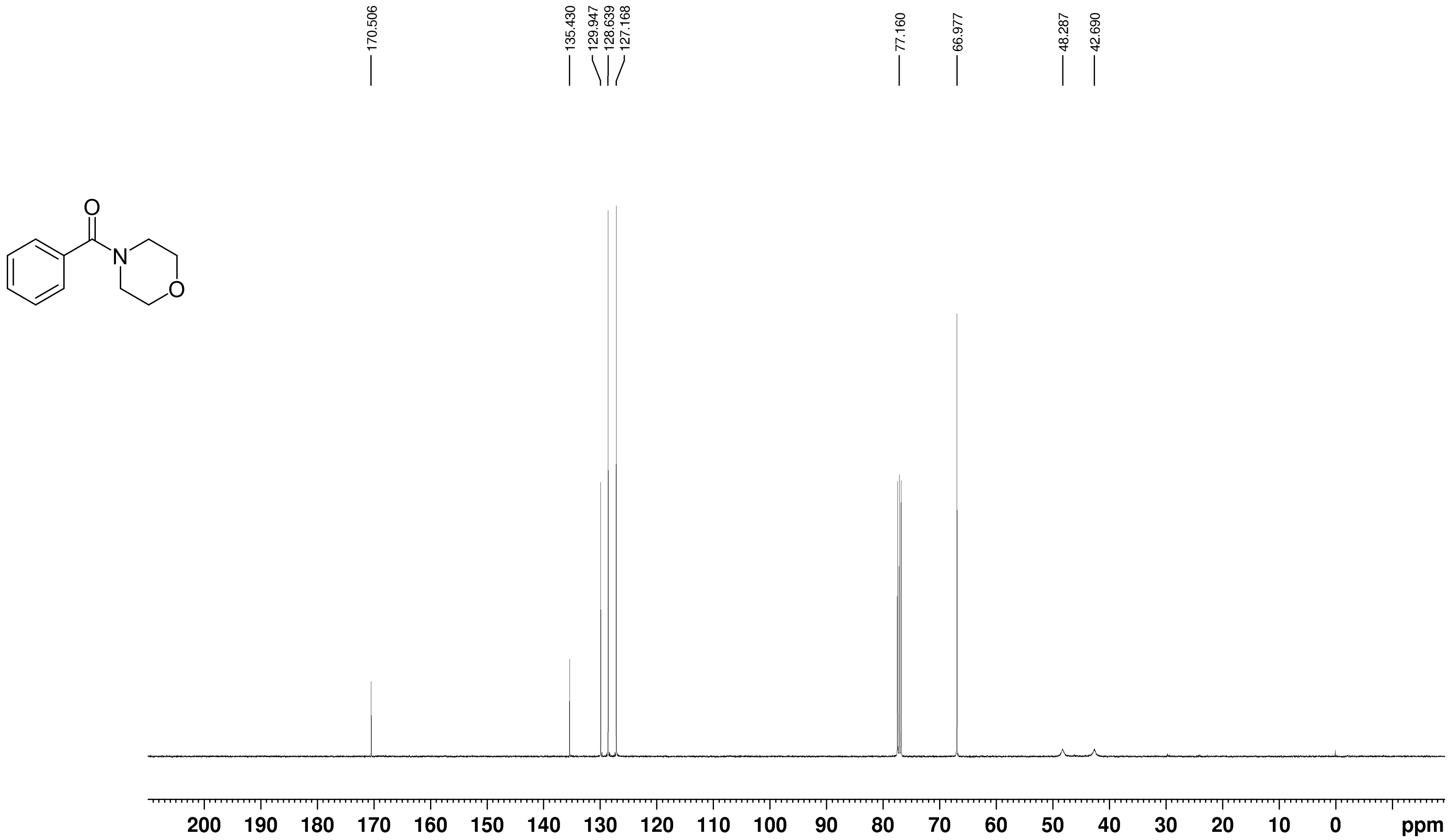

Supplementary Figure 56. ^{1}H NMR (400 MHz, $CDCl_3$) 1-(3-chlorotetrahydrofuran-2-yl)pyrrolidine-2,5-dione (5aa)

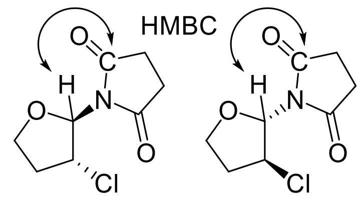

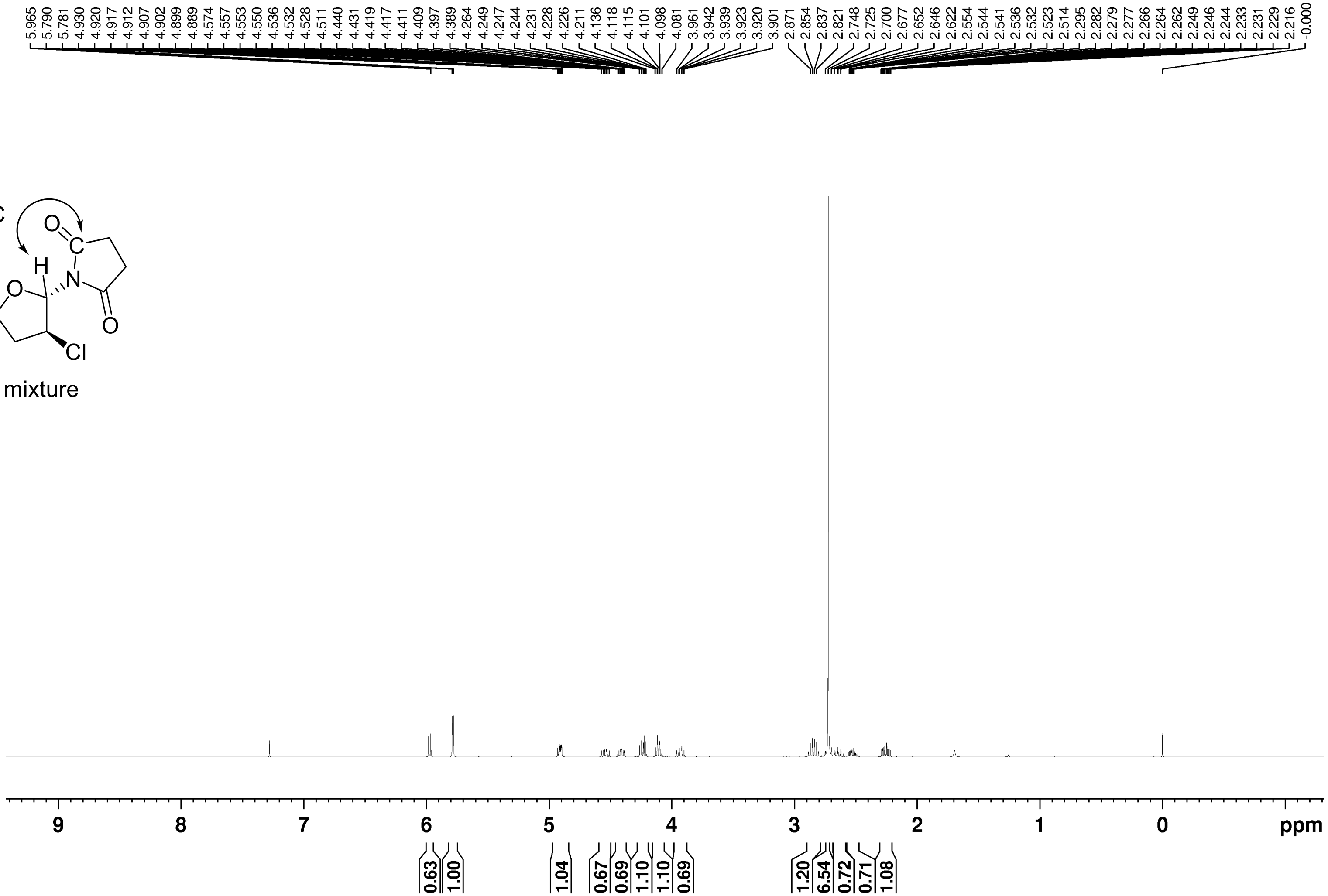

Supplementary Figure 57. ^{13}C NMR ($CDCl_3$, 100 MHz) 1-(3-chlorotetrahydrofuran-2-yl)pyrrolidine-2,5-dione (5aa)

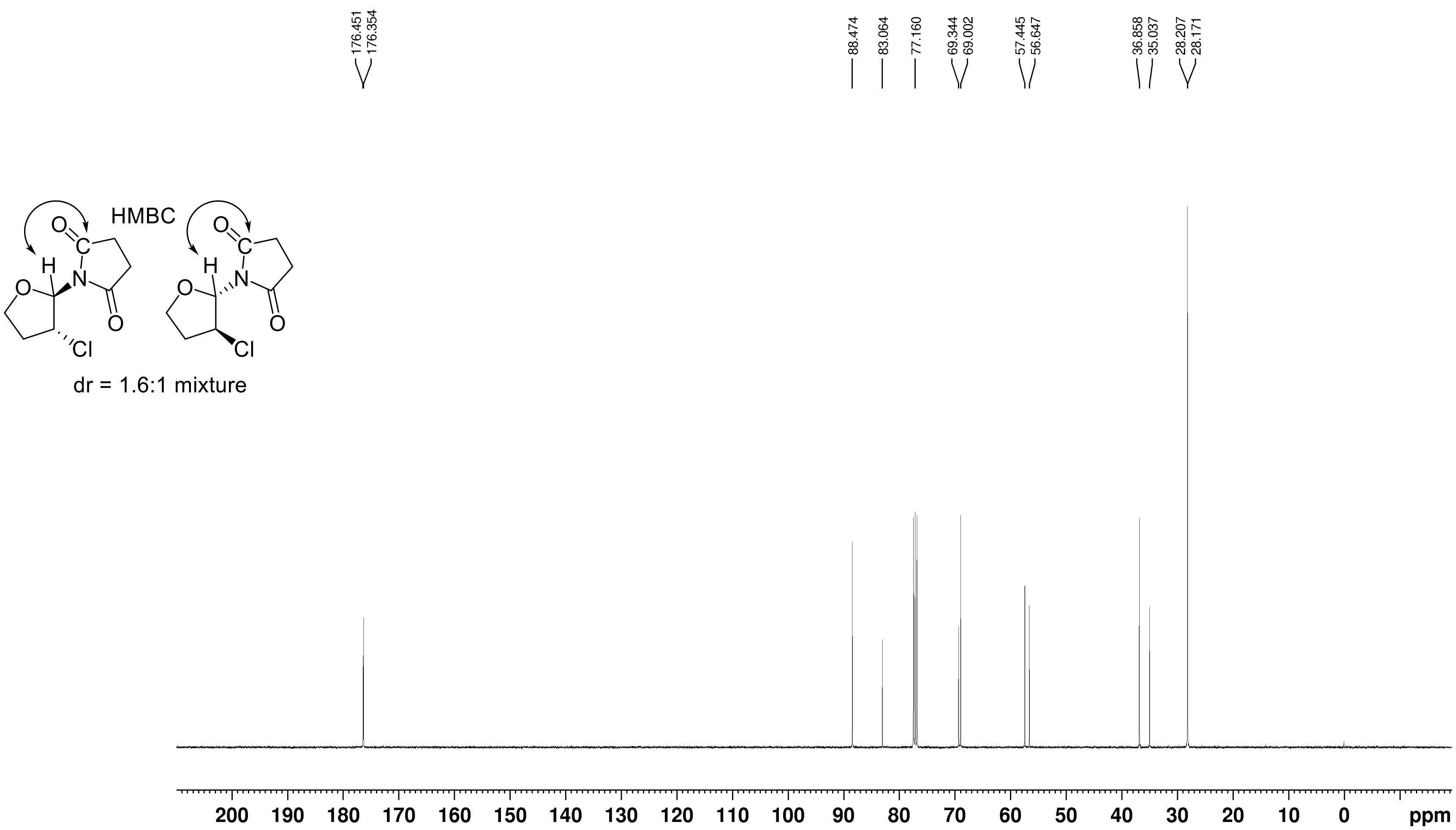

Supplementary Figure 58. ^{1}H NMR (400 MHz, $CDCl_3$) 1-((1-chlorocyclohexyl)methyl)pyrrolidine-2,5-dione (5ba)

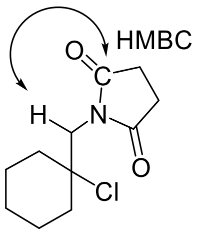

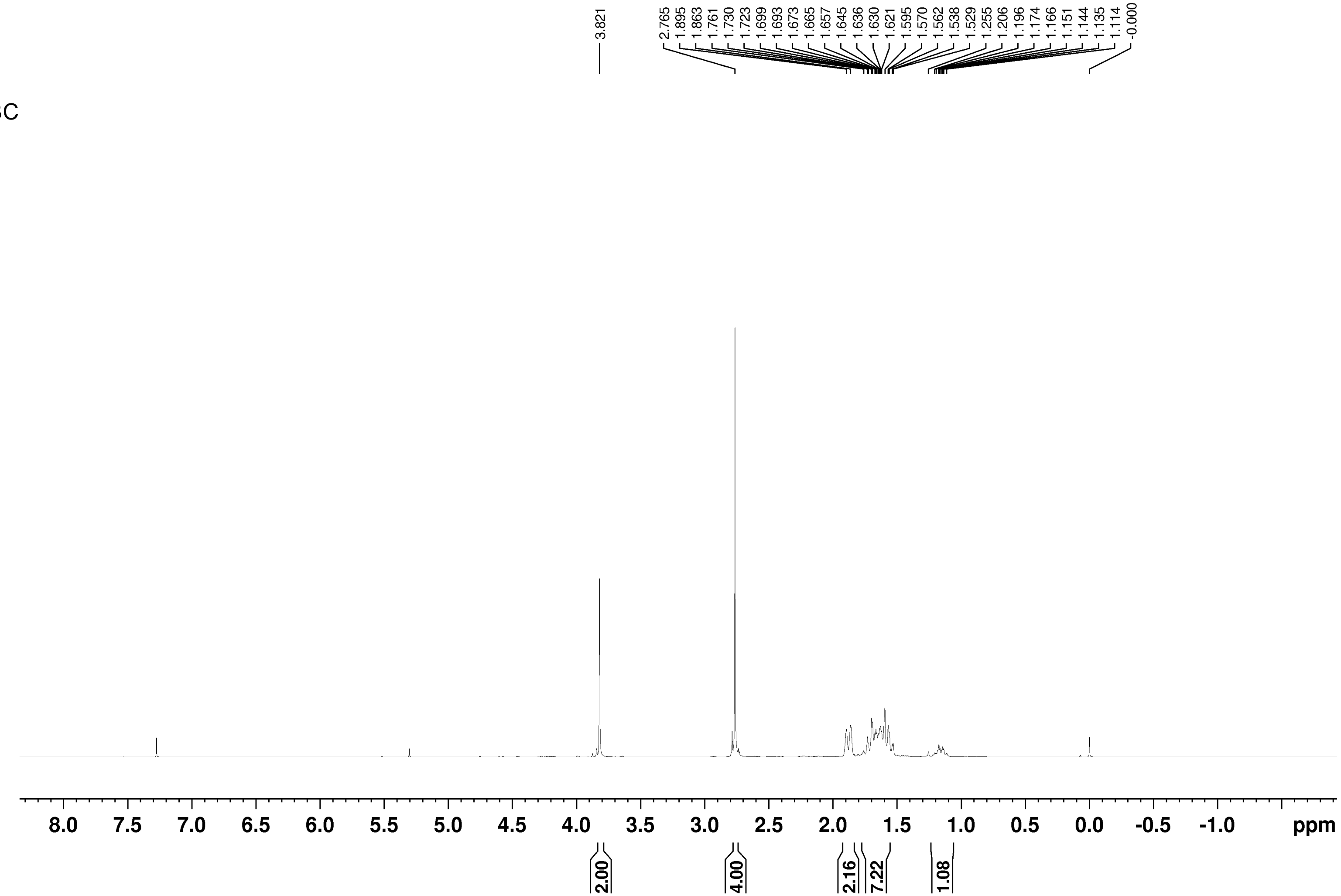

Supplementary Figure 59. ^{13}C NMR ($CDCl_3$, 100 MHz) 1-((1-chlorocyclohexyl)methyl)pyrrolidine-2,5-dione (5ba)

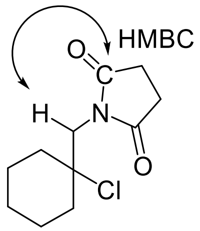

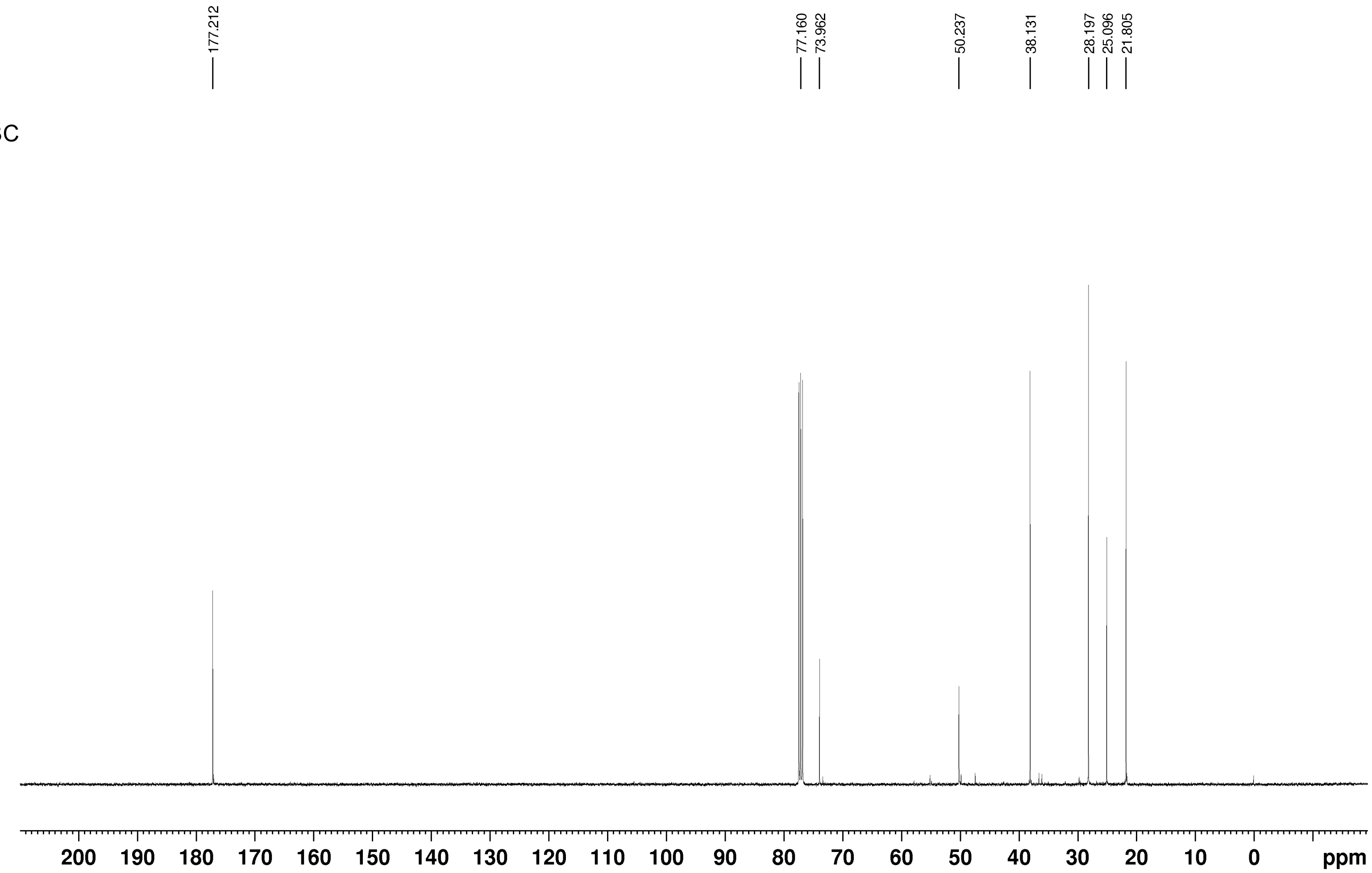

Supplementary Figure 60. ¹H NMR (400 MHz, $CDCl_3$) Methyl 9-chloro-10-(2,5-dioxopyrrolidin-1-yl)decanoate (5ca)

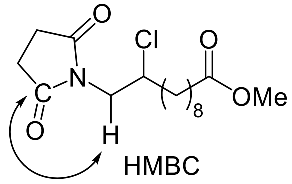

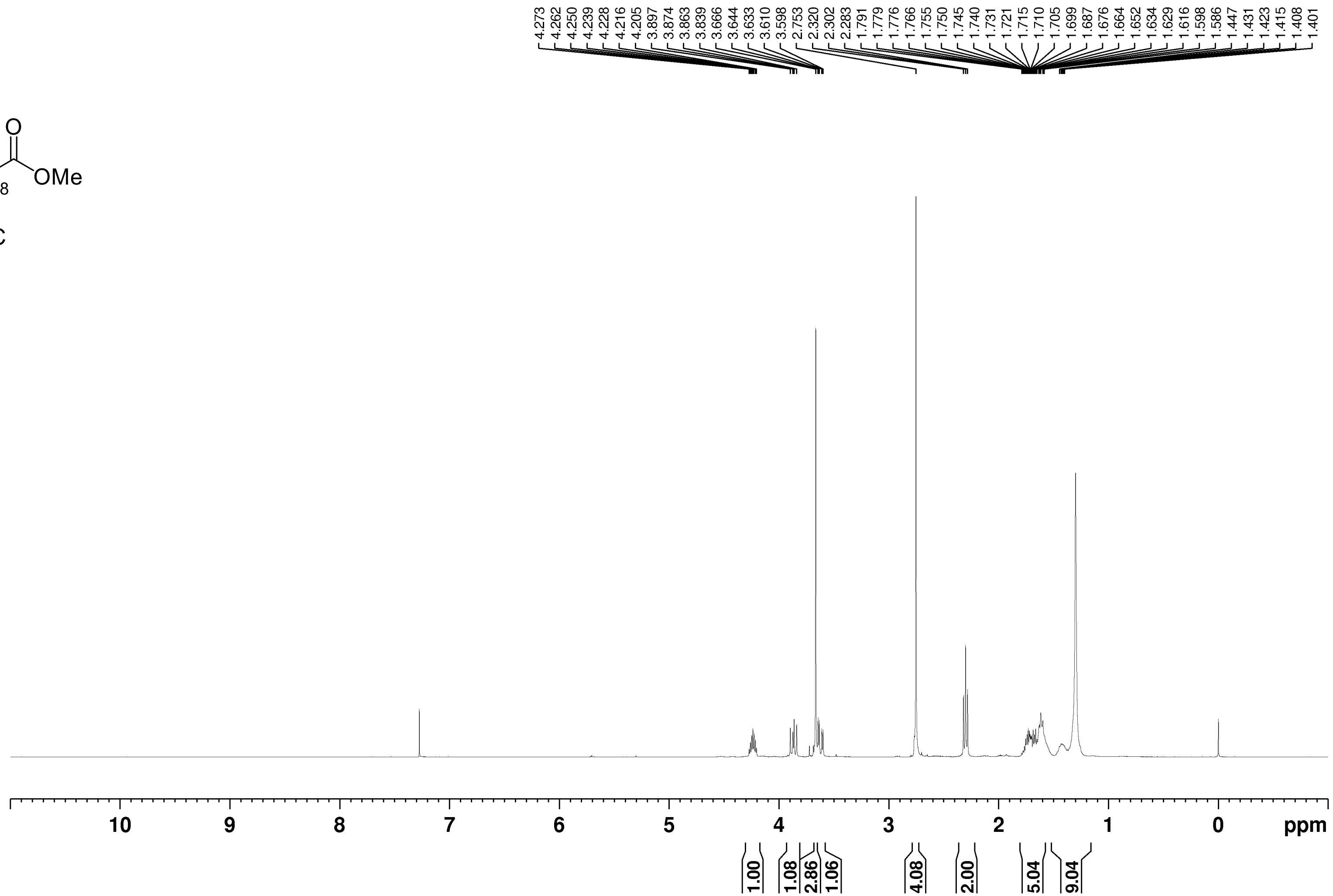

Supplementary Figure 61. ^{13}C NMR ($CDCl_3$, 100 MHz) Methyl 9-chloro-10-(2,5-dioxopyrrolidin-1-yl)decanoate (5ca)

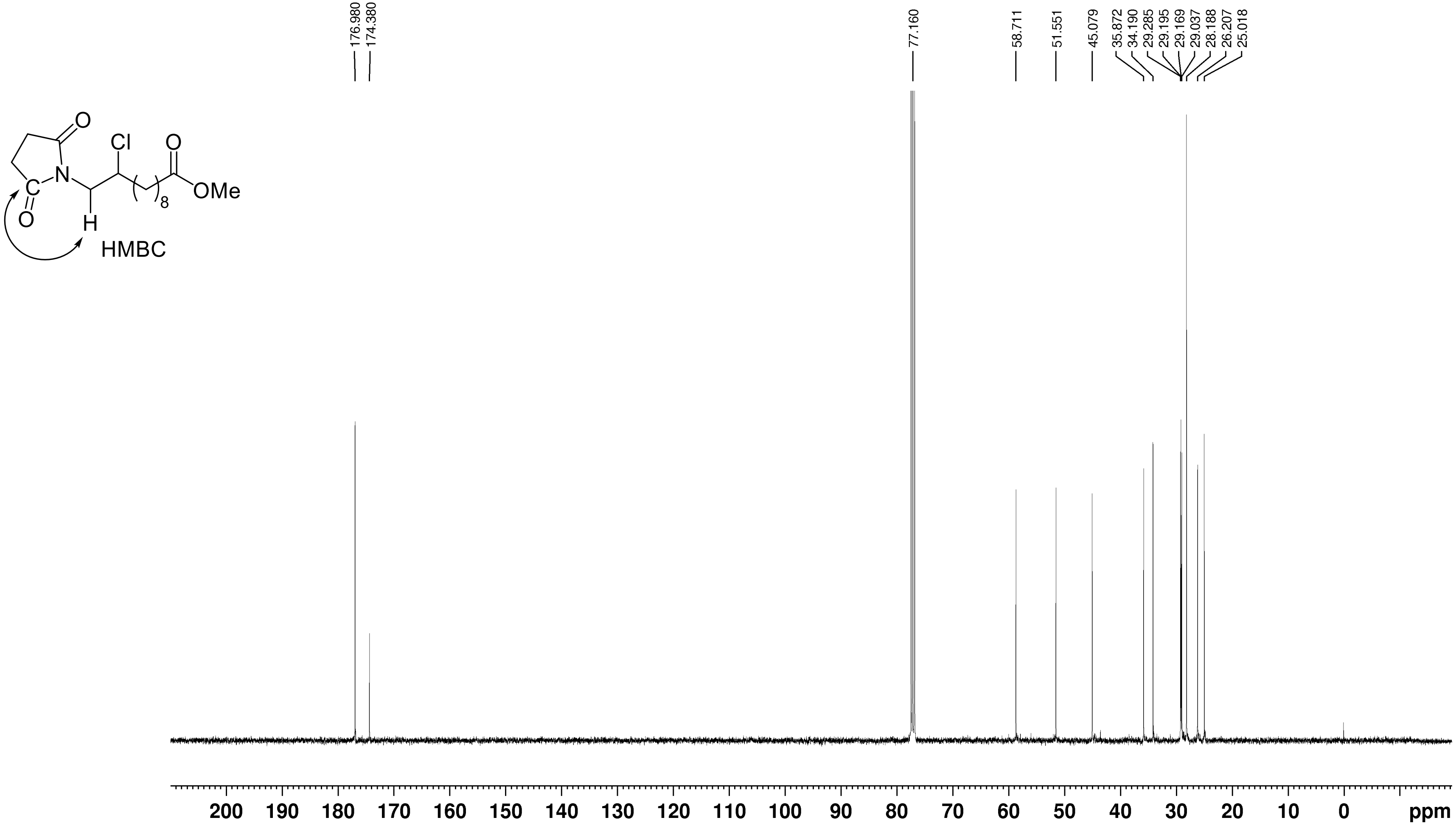

Supplementary Figure 62. ^{1}H NMR (400 MHz, $CDCl_3$) 2-(2-chlorocyclohexyl)benzo[*d*]isothiazol-3(2*H*)-one 1,1-dioxide (5dc)

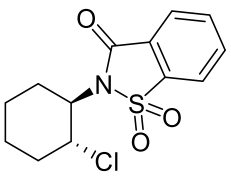

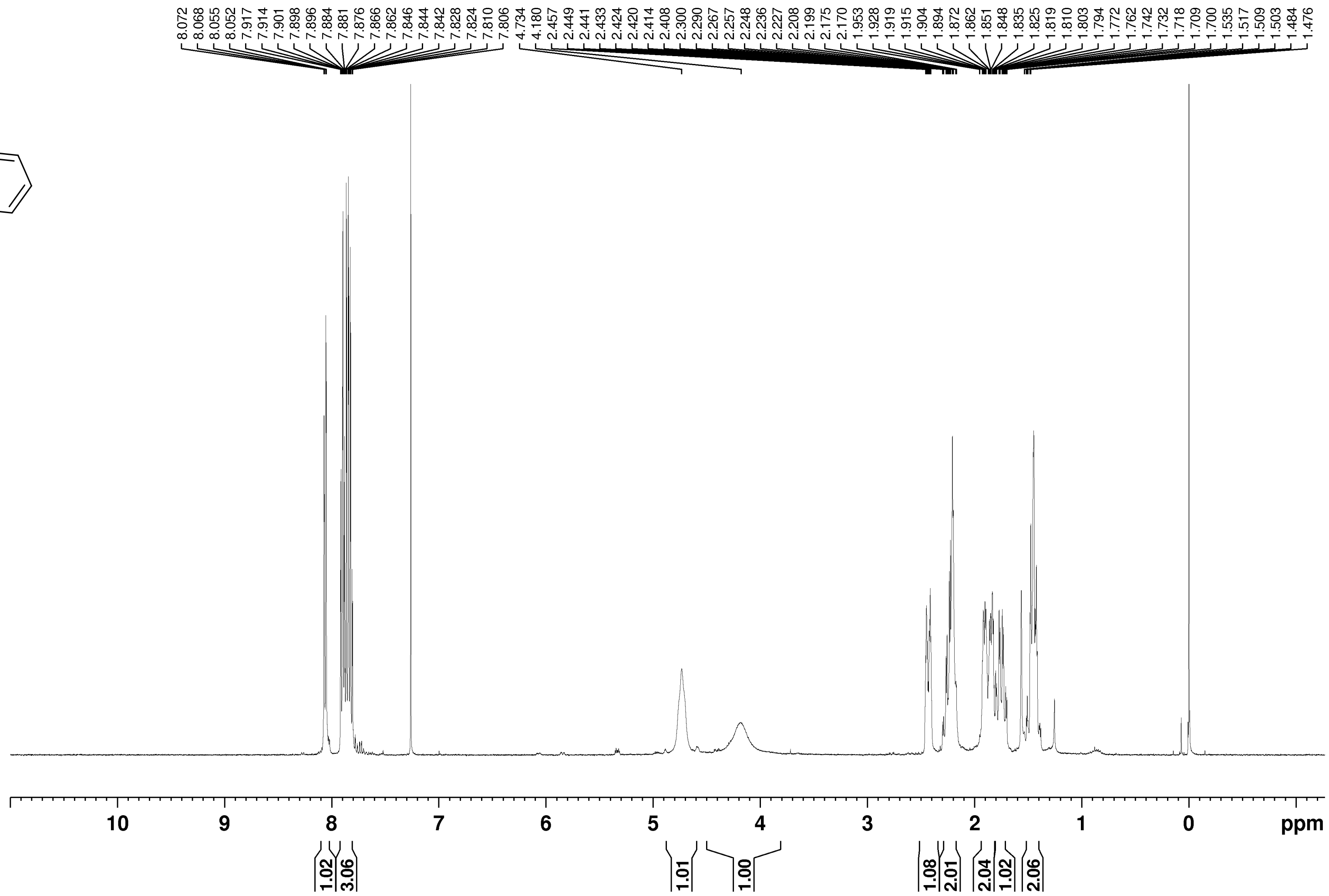

Supplementary Figure 63. ^{13}C NMR ($CDCl_3$, 100 MHz) 2-(2-chlorocyclohexyl)benzo[*d*]isothiazol-3(2*H*)-one 1,1-dioxide (5dc)

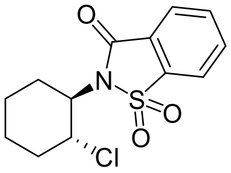

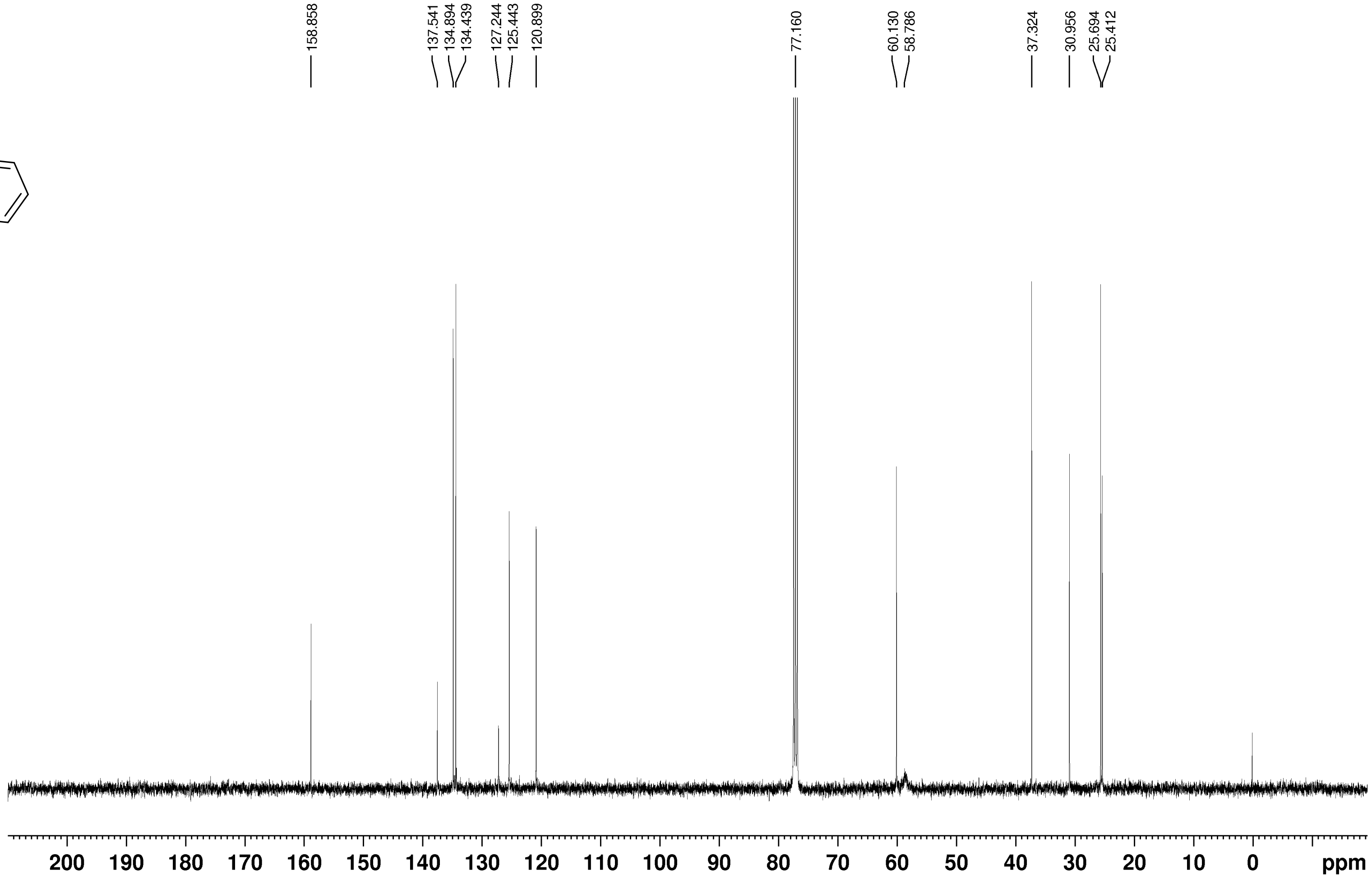

Supplementary Figure 64. ^{1}H NMR (400 MHz, $CDCl_3$) 2-(2-chloro-1-phenylethyl)benzo[*d*]isothiazol-3(2*H*)-one 1,1-dioxide (5ec)

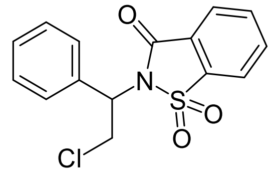

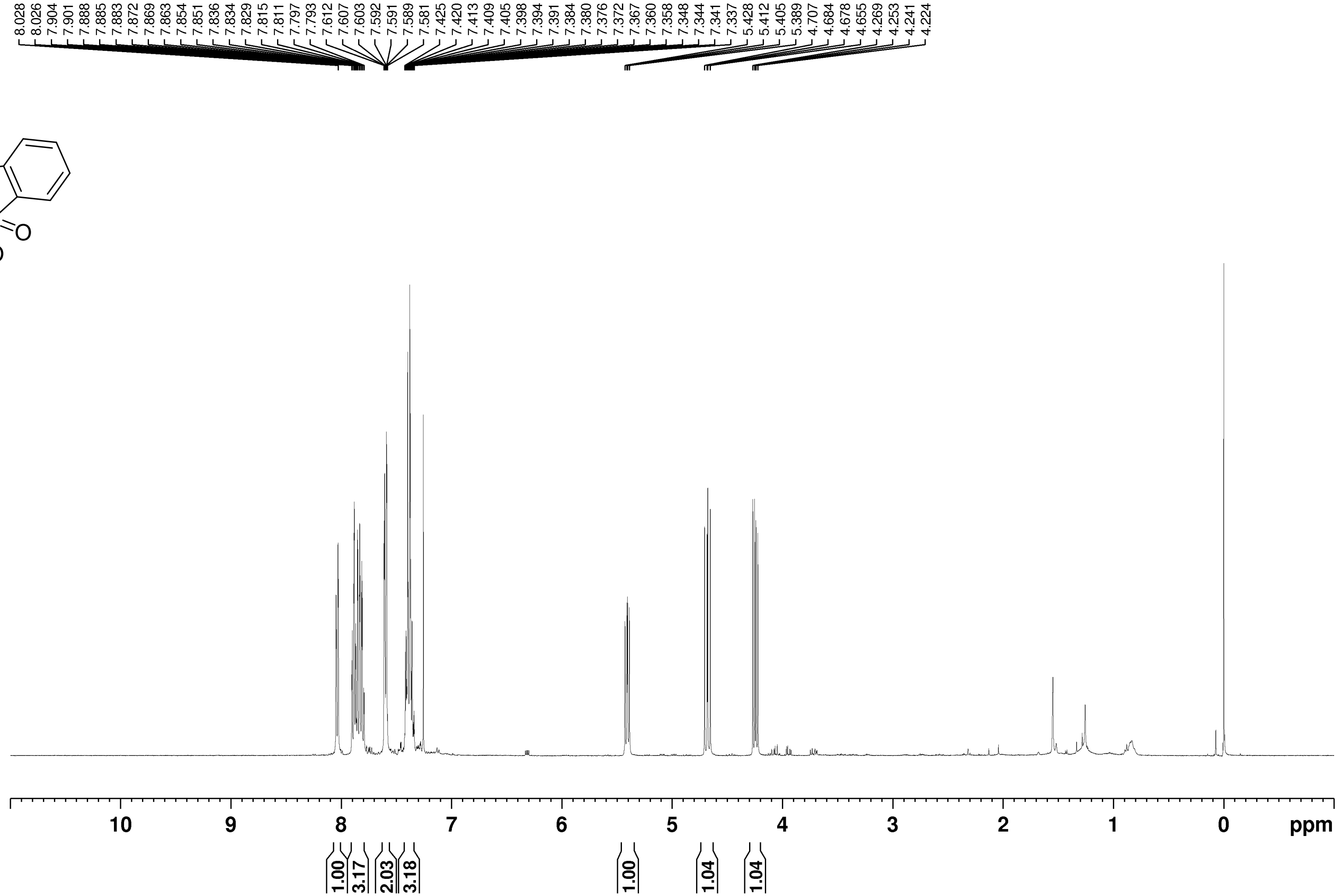

Supplementary Figure 65. ^{13}C NMR ($CDCl_3$, 100 MHz) 2-(2-chloro-1-phenylethyl)benzo[*d*]isothiazol-3(2*H*)-one 1,1-dioxide (5ec)

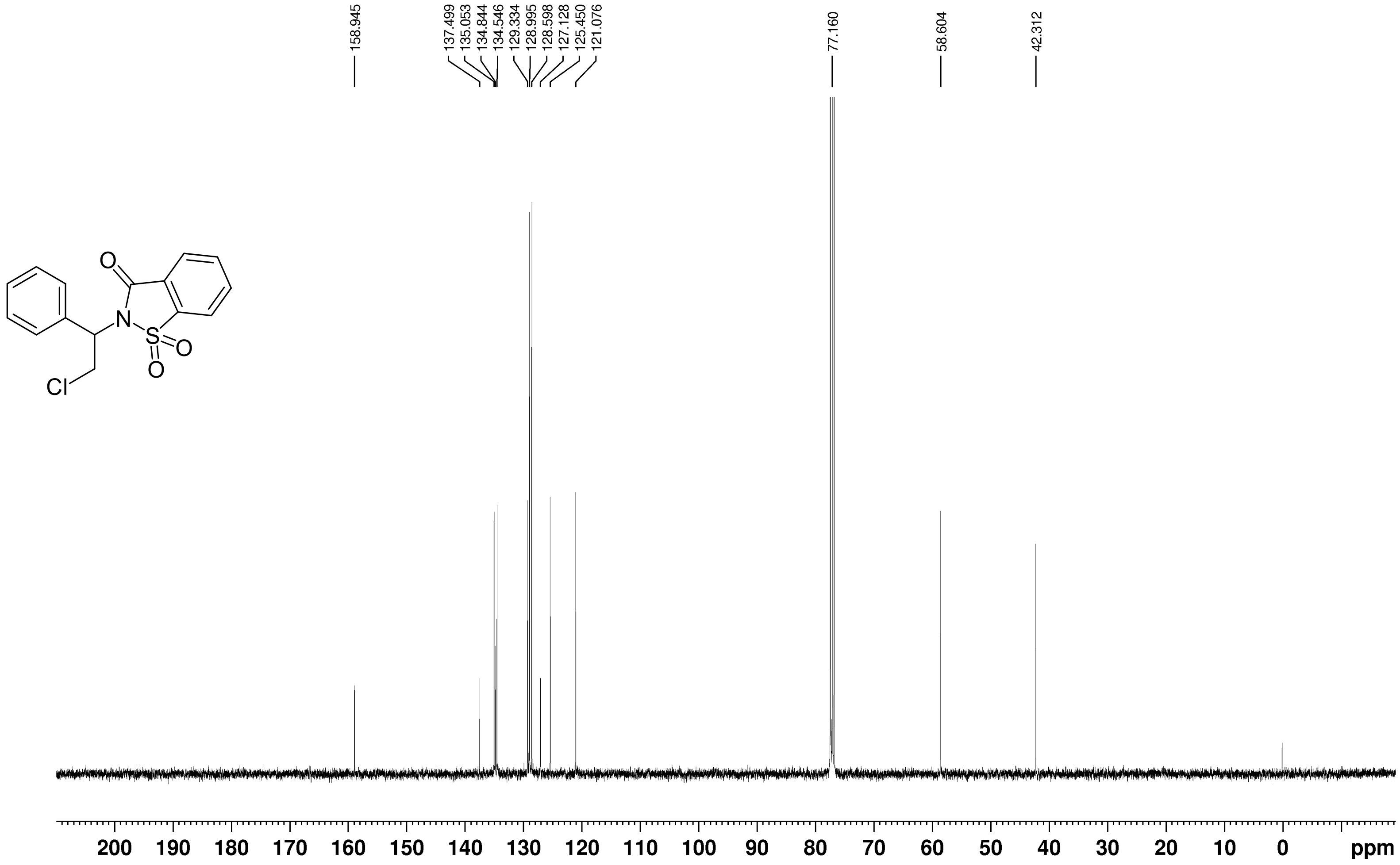

Supplementary Figure 66. ¹H NMR (400 MHz, $CDCl_3$) 2-(2-chloro-1-(*p*-tolyl)ethyl)benzo[*d*]isothiazol-3(2*H*)-one 1,1-dioxide (5fc)

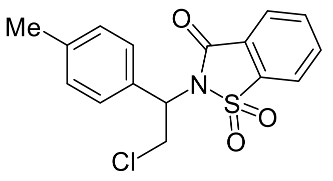

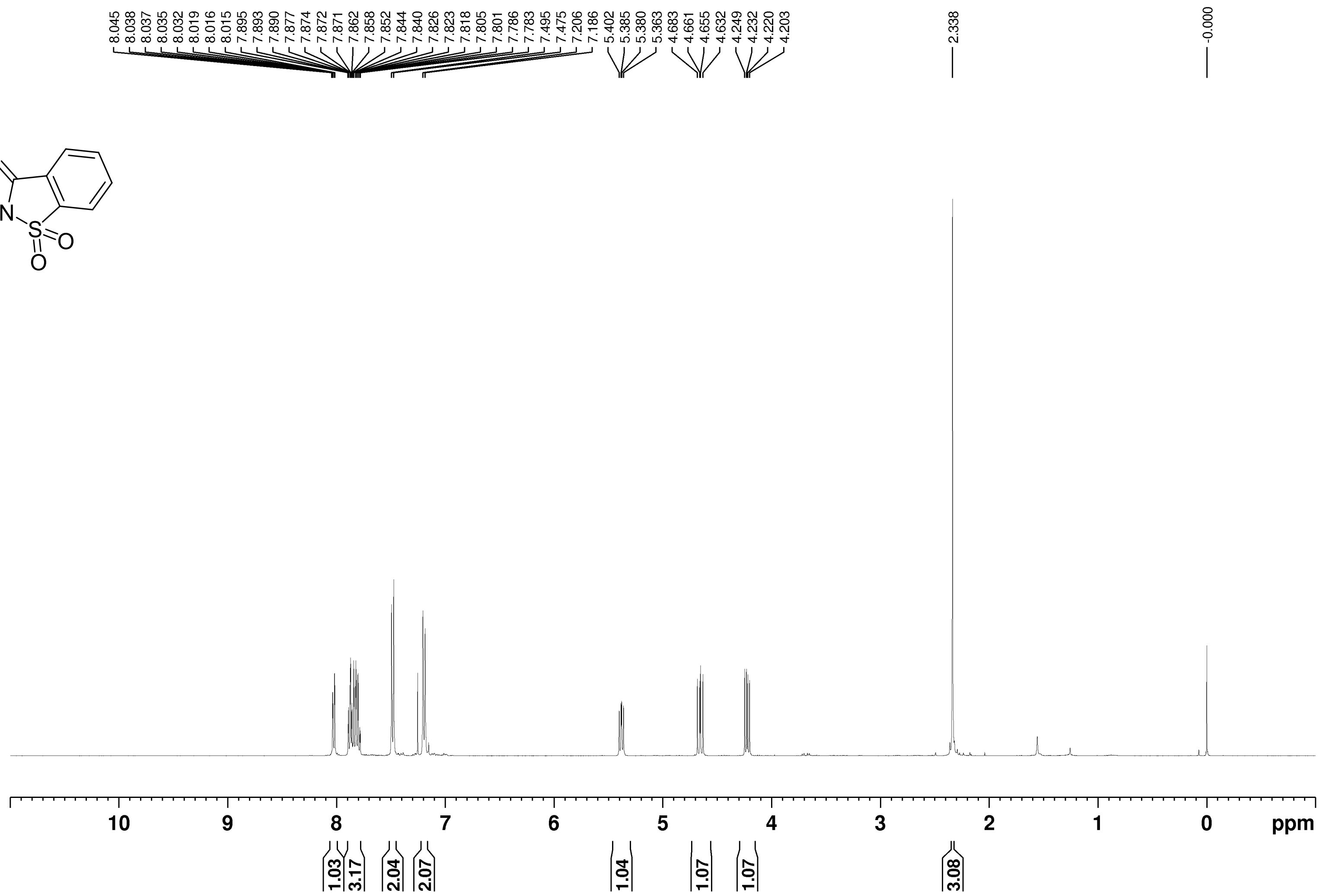

Supplementary Figure 67. ^{13}C NMR ($CDCl_3$, 100 MHz) 2-(2-chloro-1-(*p*-tolyl)ethyl)benzo[*d*]isothiazol-3(2*H*)-one 1,1-dioxide (5fc)

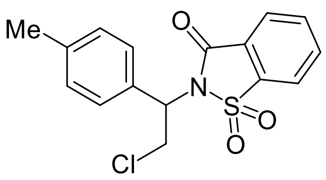

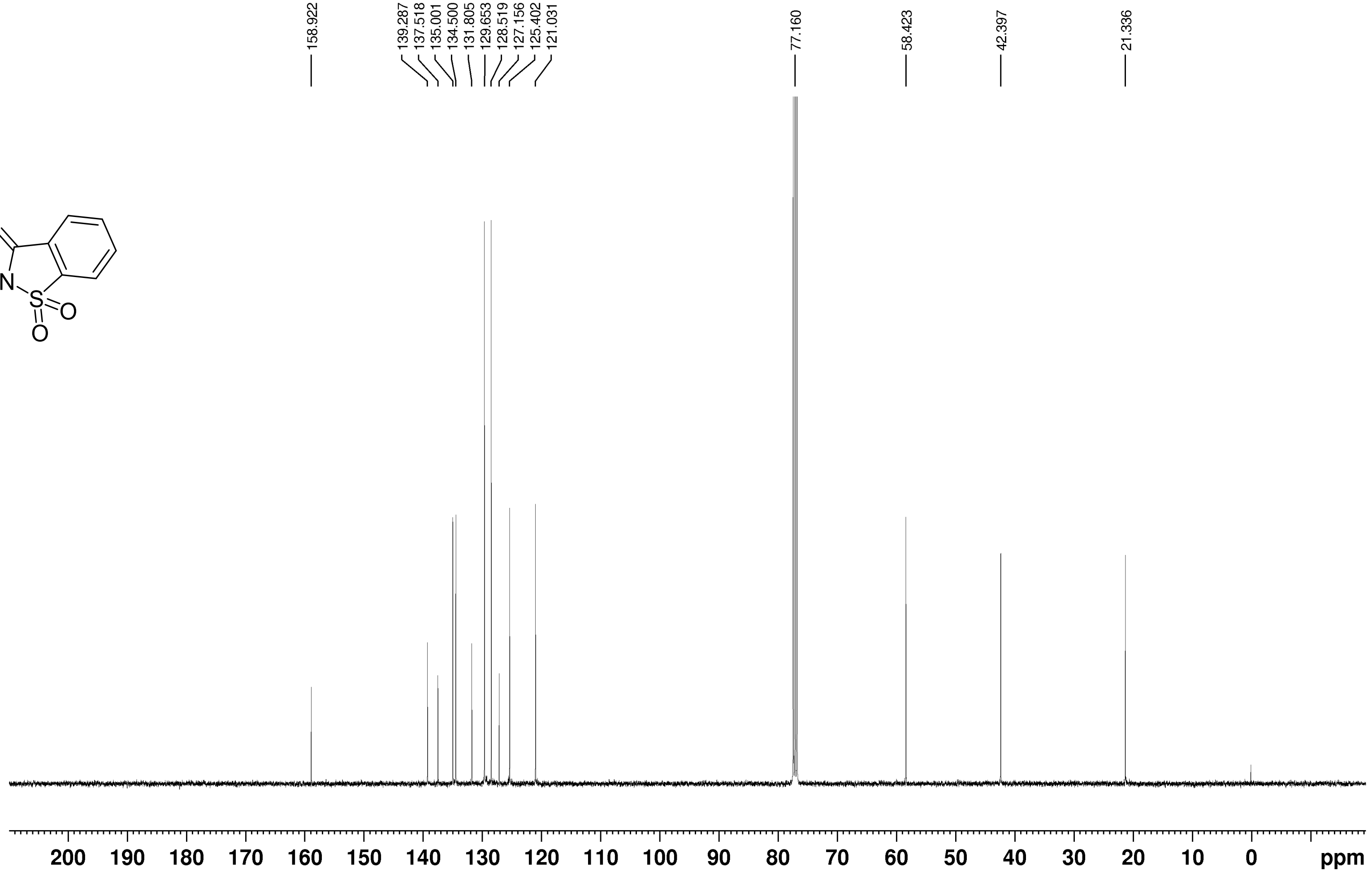

Supplementary Figure 68. ^{1}H NMR (400 MHz, $CDCl_3$) Benzoyl fluoride (6a)

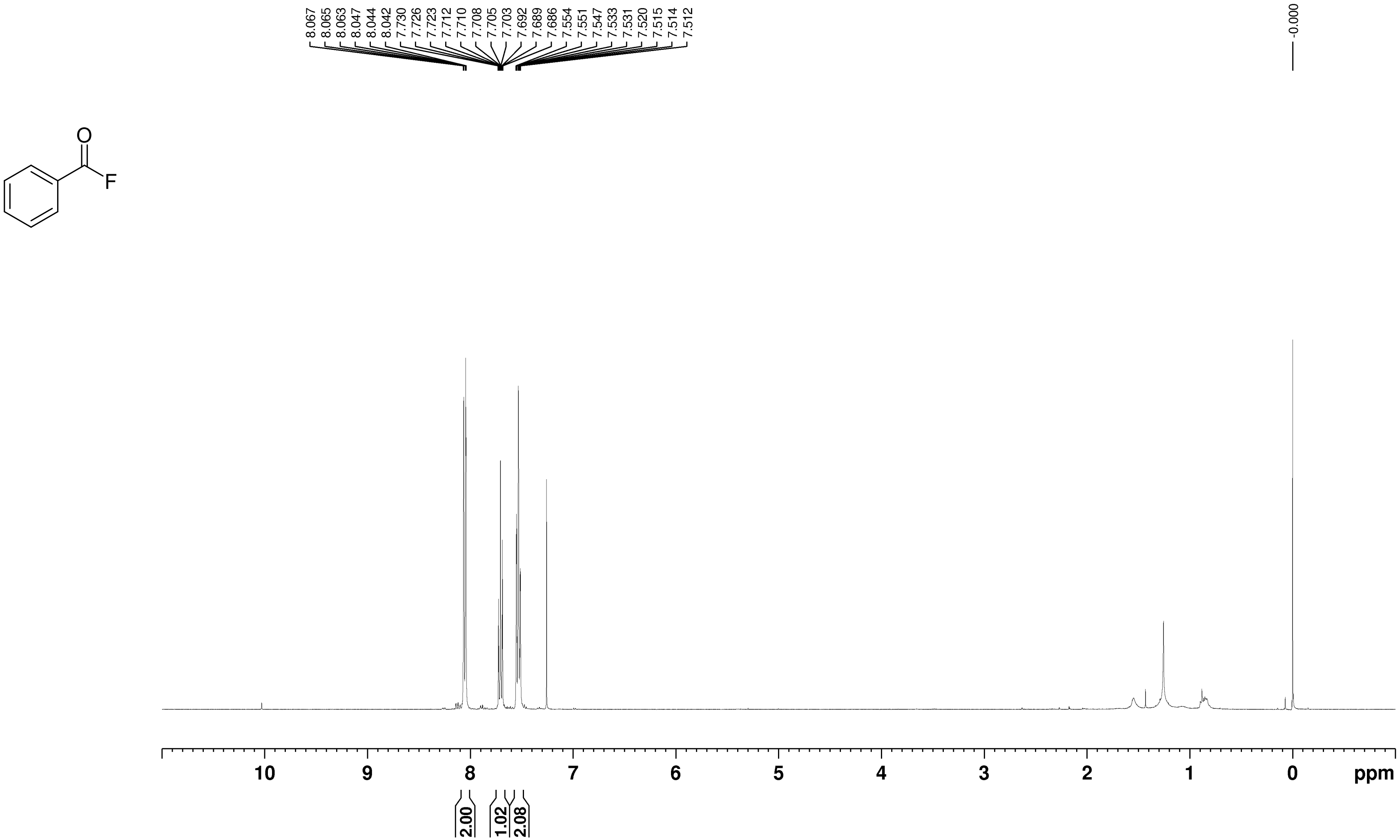

Supplementary Figure 69. ^{13}C NMR ($CDCl_3$, 100 MHz) Benzoyl fluoride (6a)

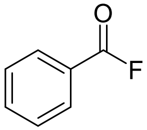

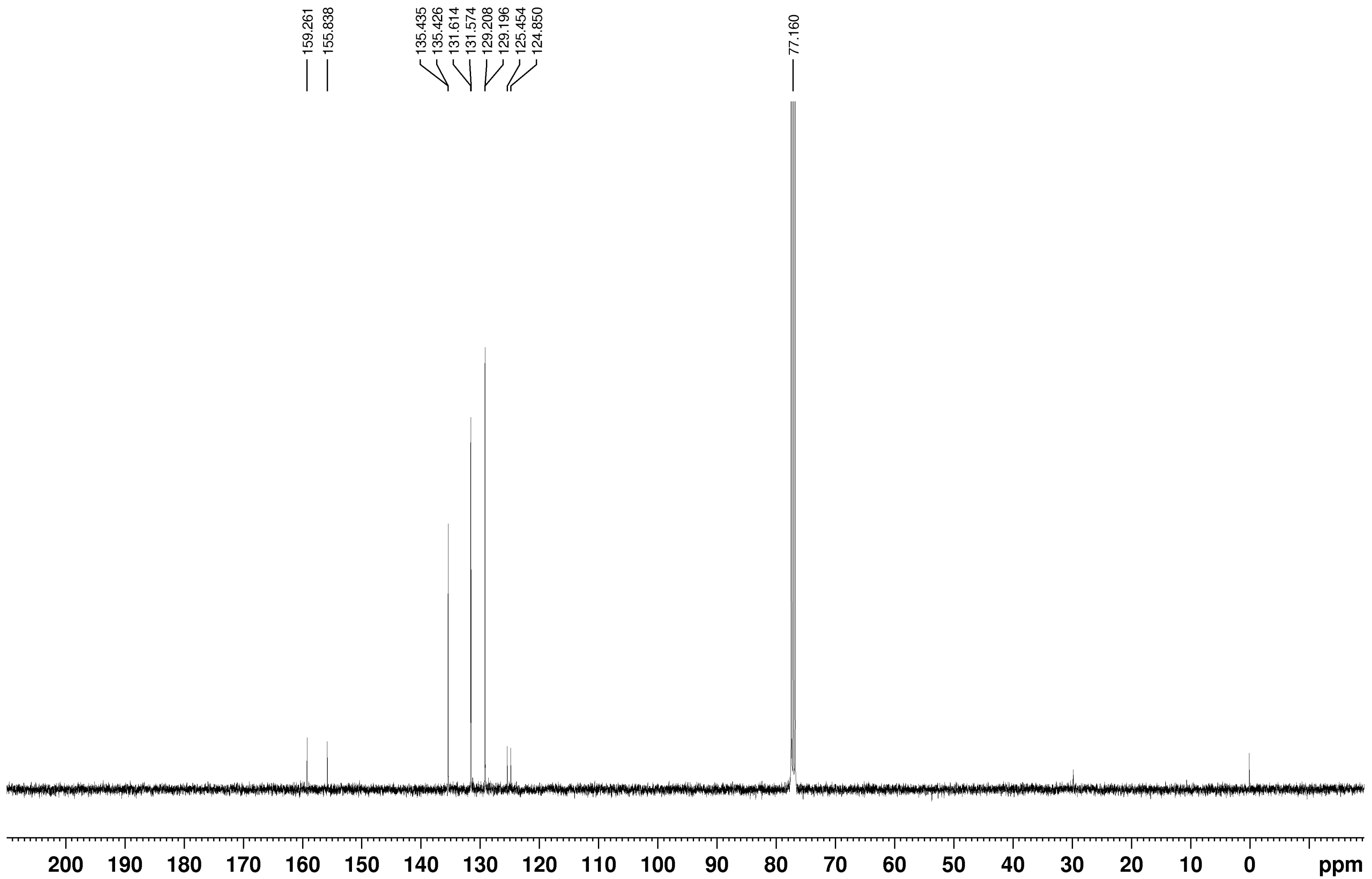

Supplementary Figure 70. ^{19}F NMR (CDCl$_3$, 376 MHz) Benzoyl fluoride (6a)

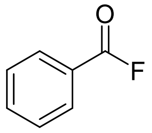

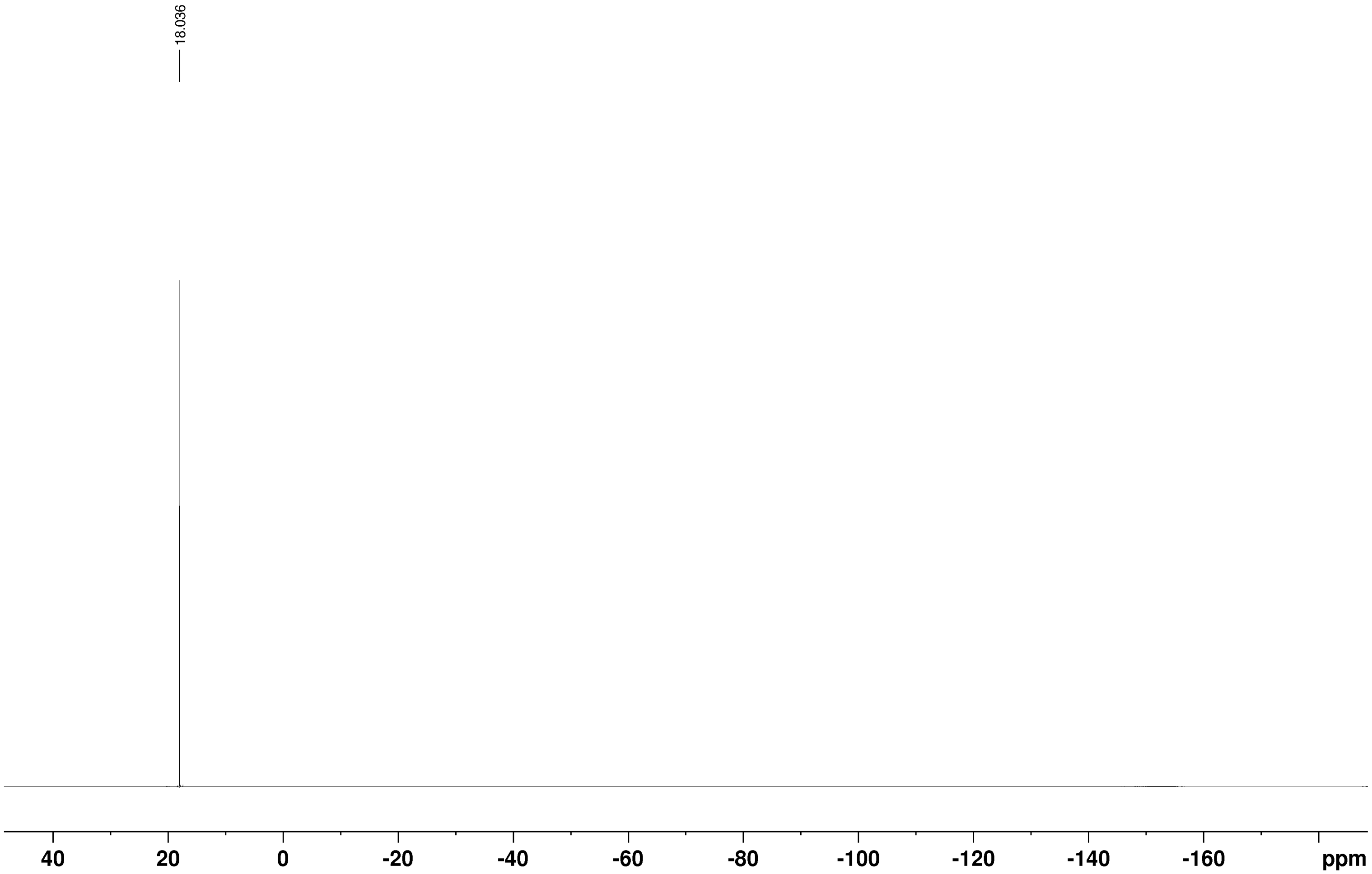

Supplementary Figure 71. ^{1}H NMR (400 MHz, $CDCl_3$) 4-chlorobenzoyl fluoride (6d)

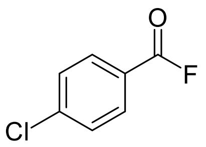

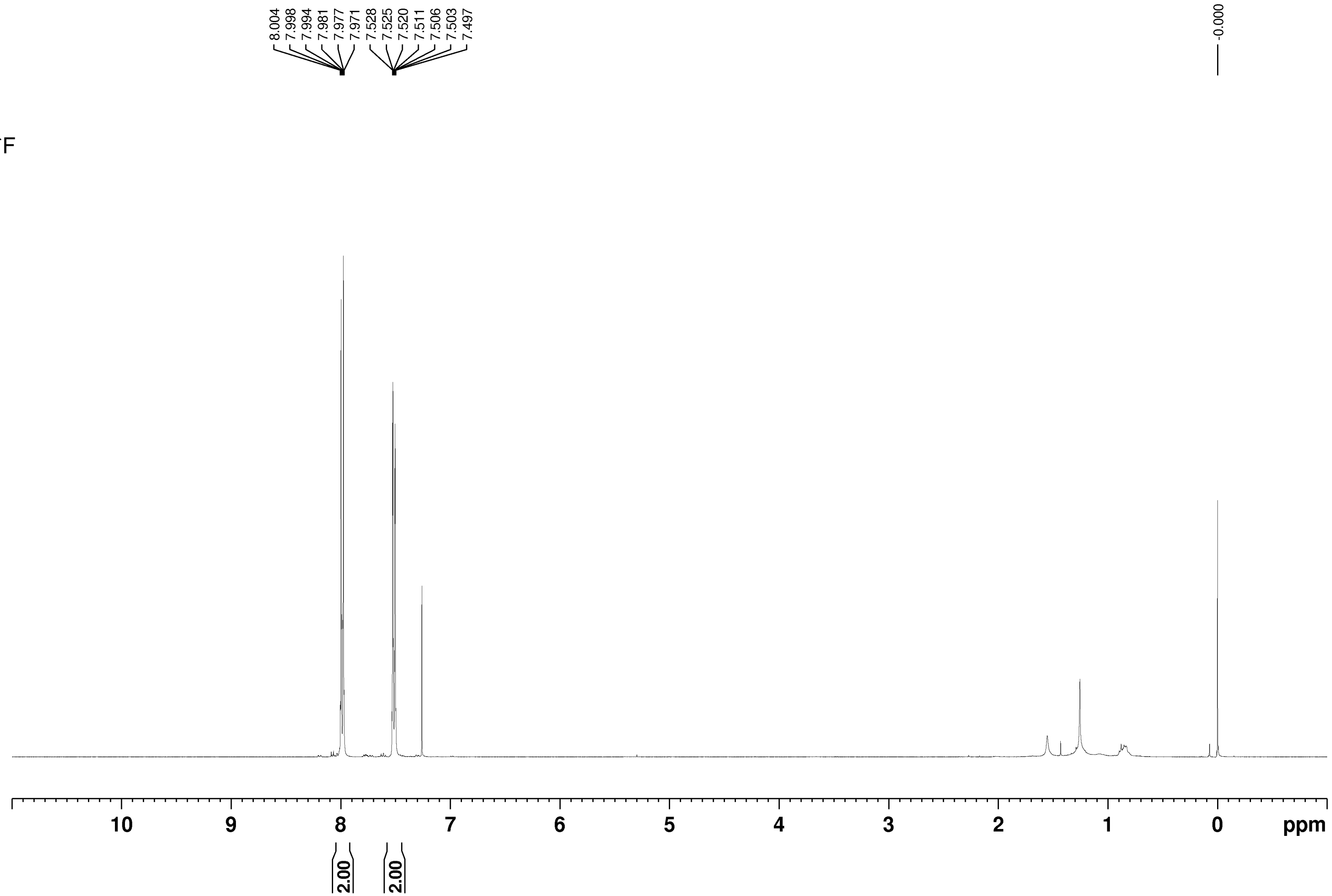

Supplementary Figure 72. ^{13}C NMR ($CDCl_3$, 100 MHz) 4-chlorobenzoyl fluoride (6d)

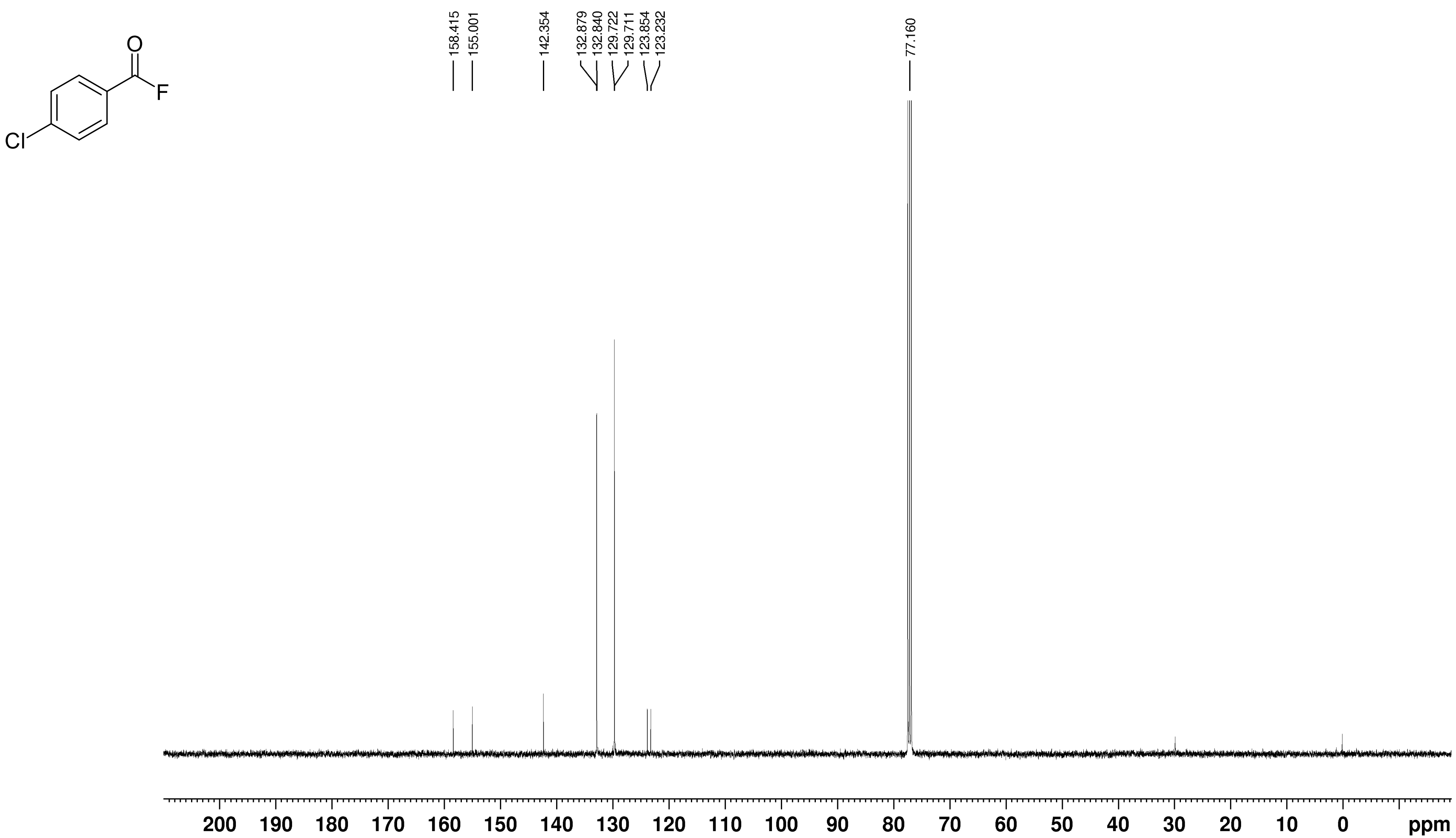

Supplementary Figure 73. ^{19}F NMR ($CDCl_3$, 376 MHz) 4-chlorobenzoyl fluoride (6d)

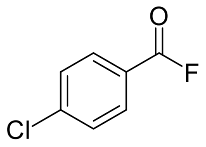

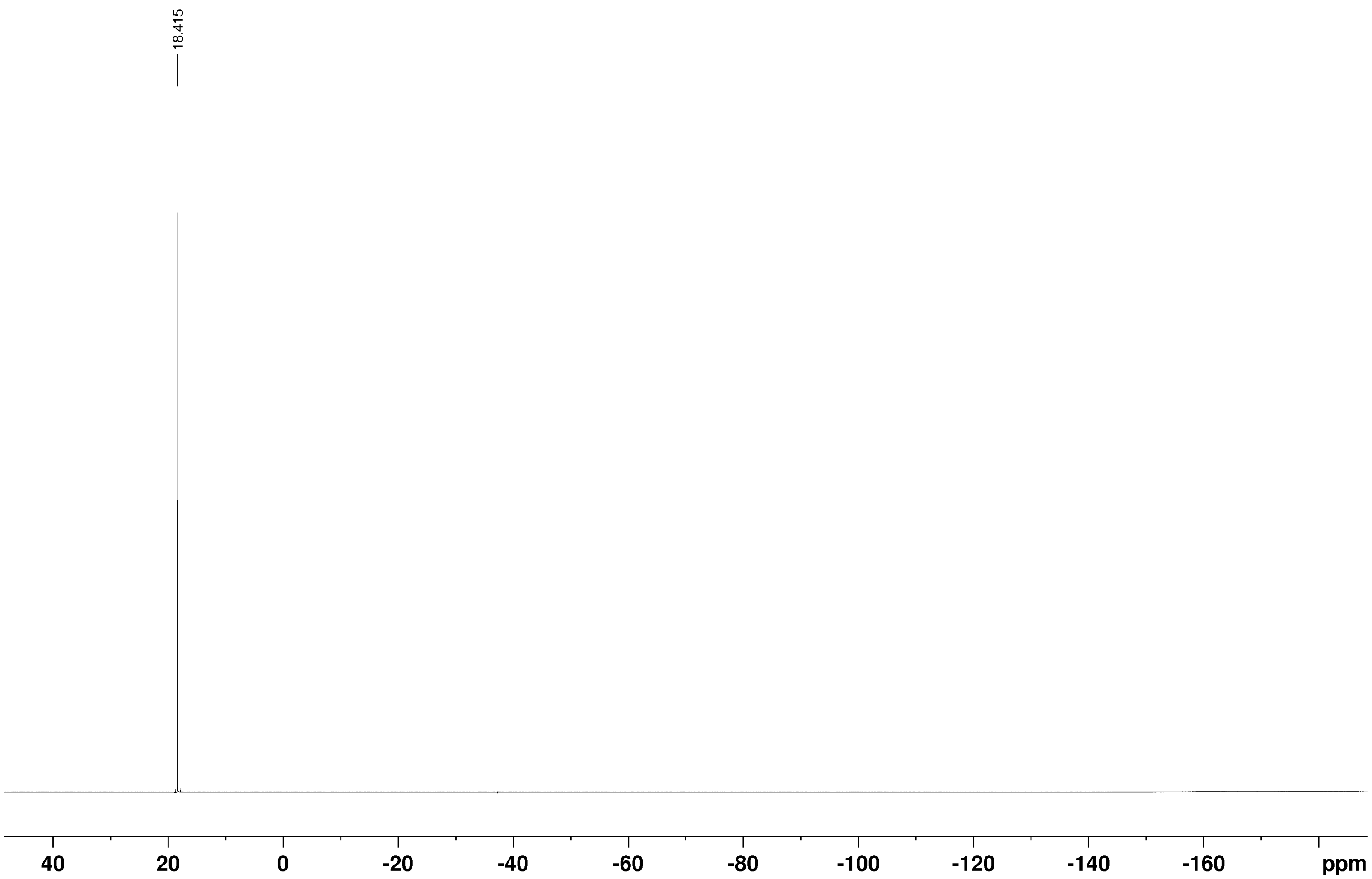

Supplementary Figure 74. ^1H NMR (400 MHz, $CDCl_3$) *N*-benzyl-4-methylbenzamide (3ld)

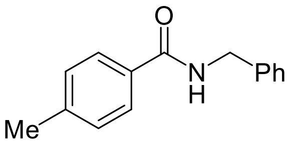

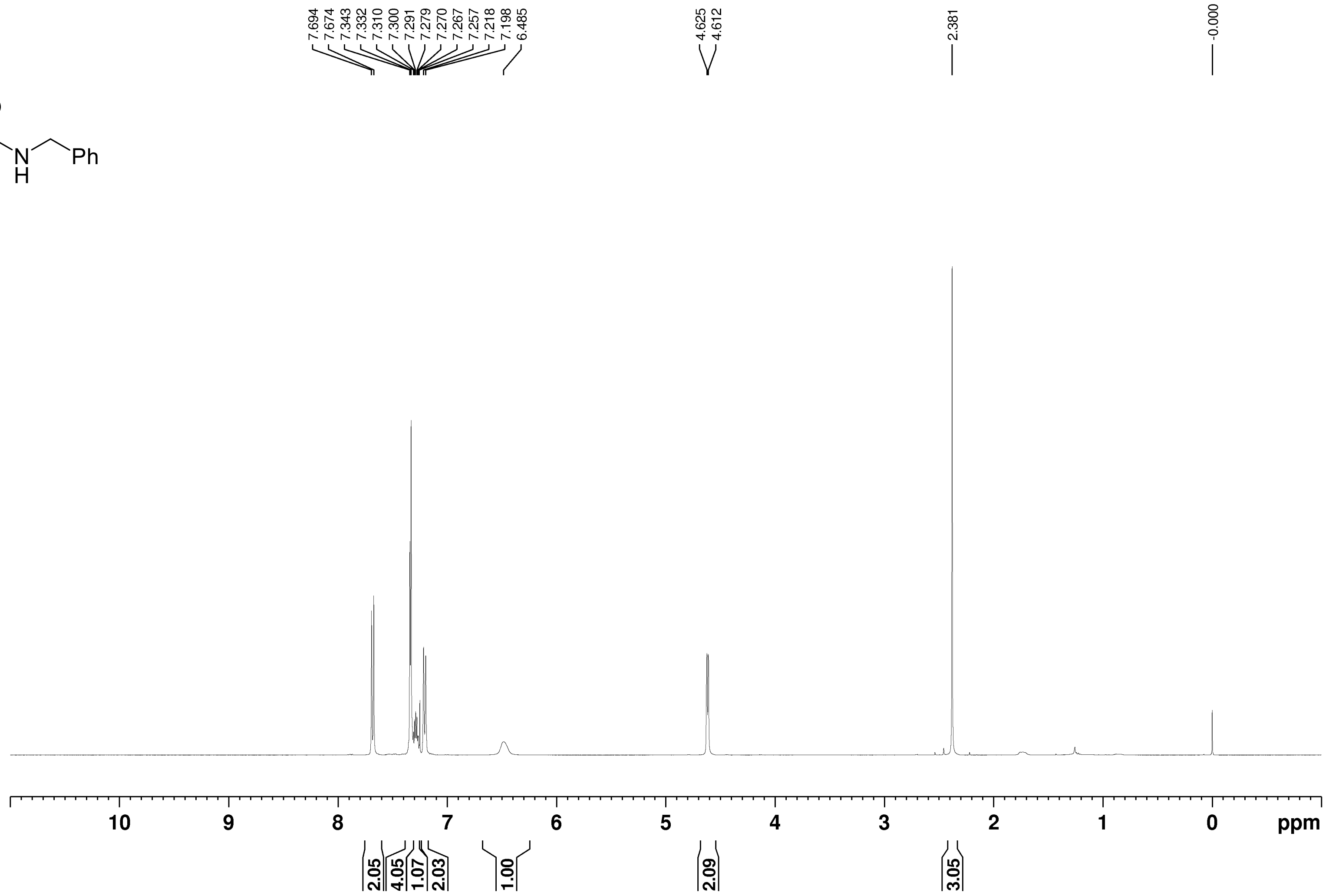

Supplementary Figure 75. ^{13}C NMR ($CDCl_3$, 100 MHz) *N*-benzyl-4-methylbenzamide (3ld)

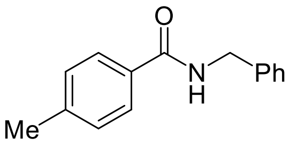

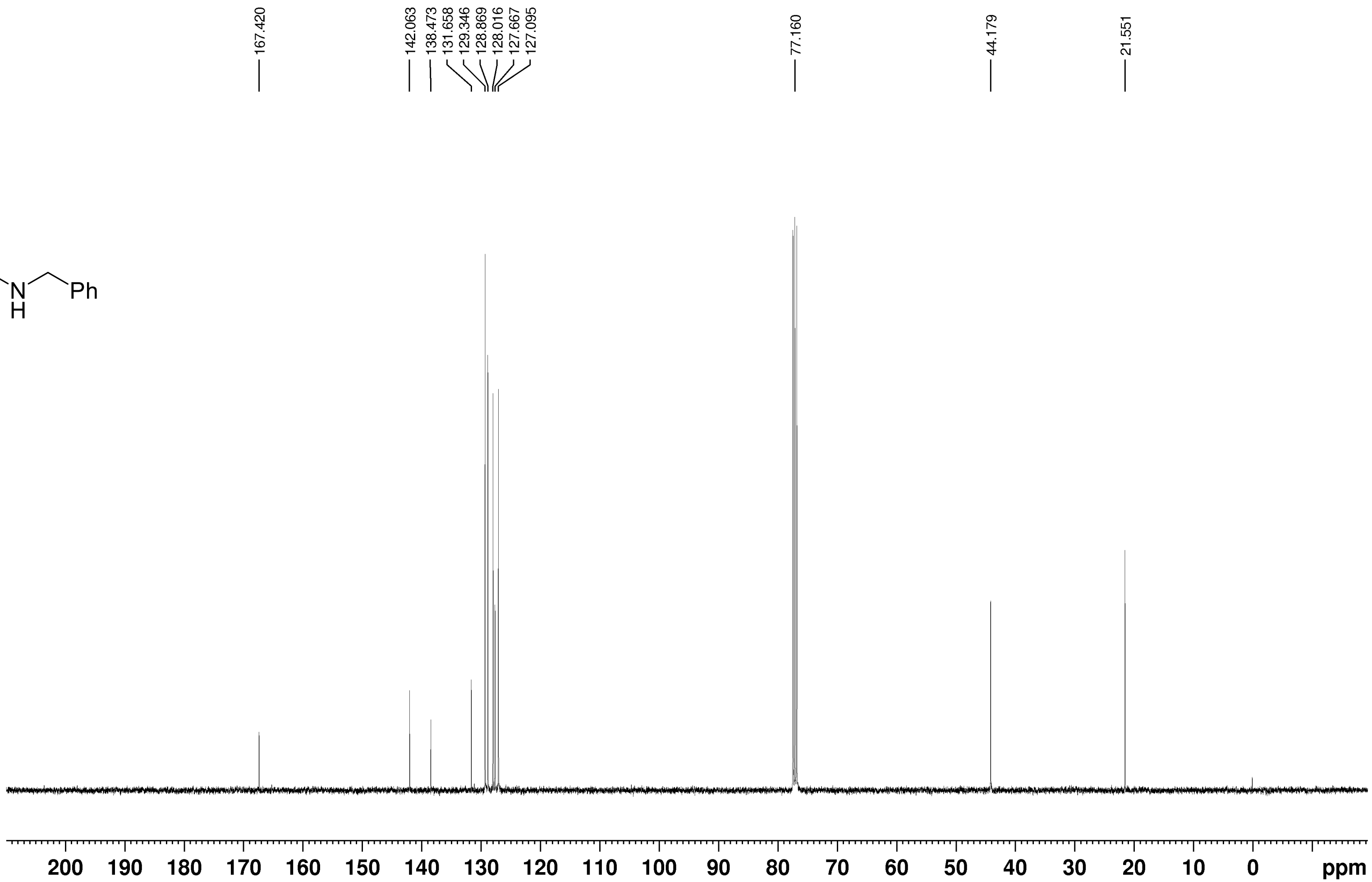

Supplementary Figure 76. ^{1}H NMR (400 MHz, $CDCl_3$) 3-phenylpropanoyl fluoride (6s)

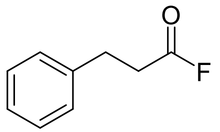

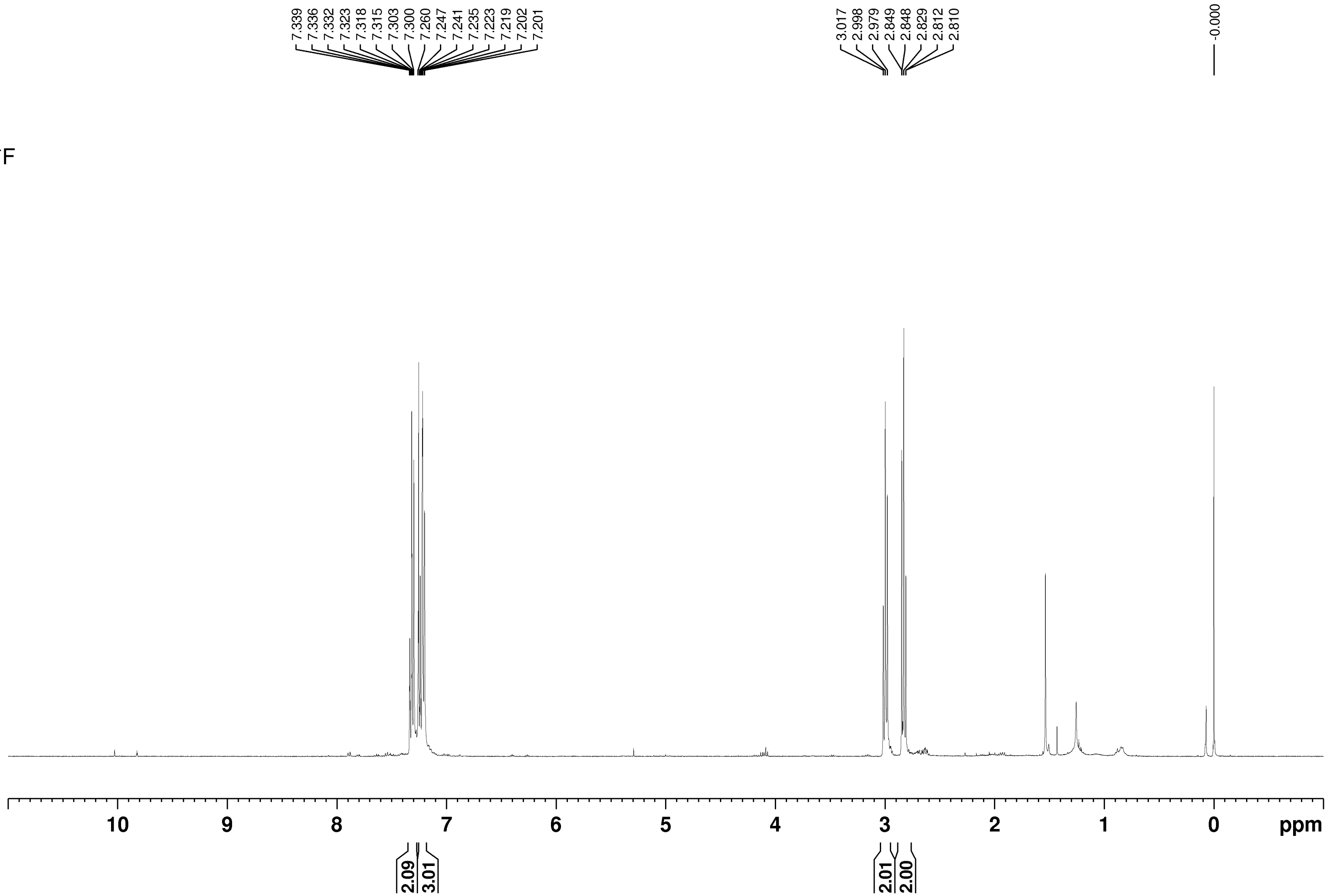

Supplementary Figure 77. ^{13}C NMR ($CDCl_3$, 100 MHz) 3-phenylpropanoyl fluoride (6s)

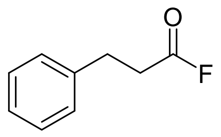

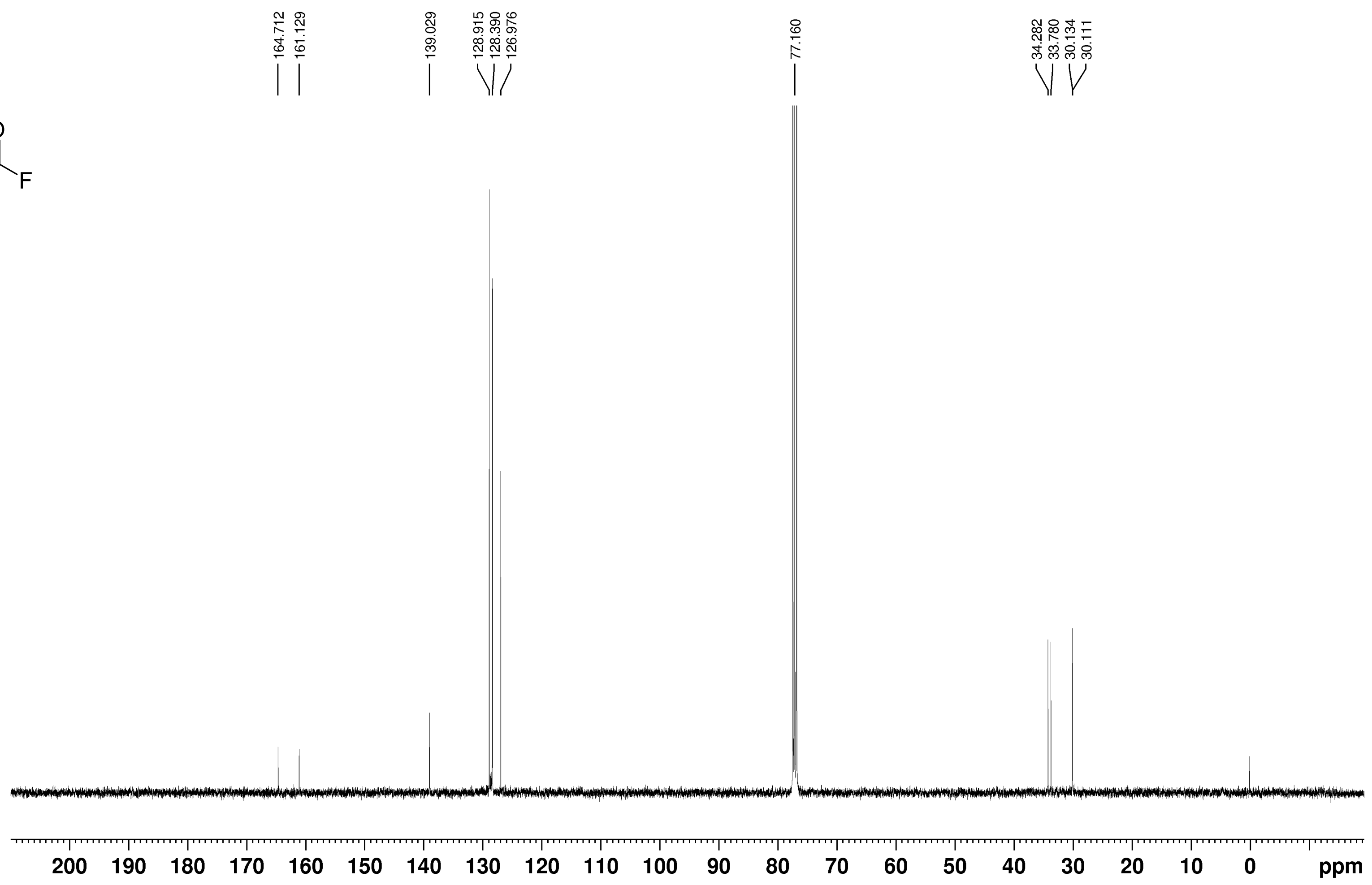

Supplementary Figure 78. ^{19}**F NMR (CDCl$_3$, 376 MHz) 3-phenylpropanoyl fluoride (6s)**

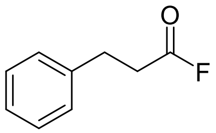

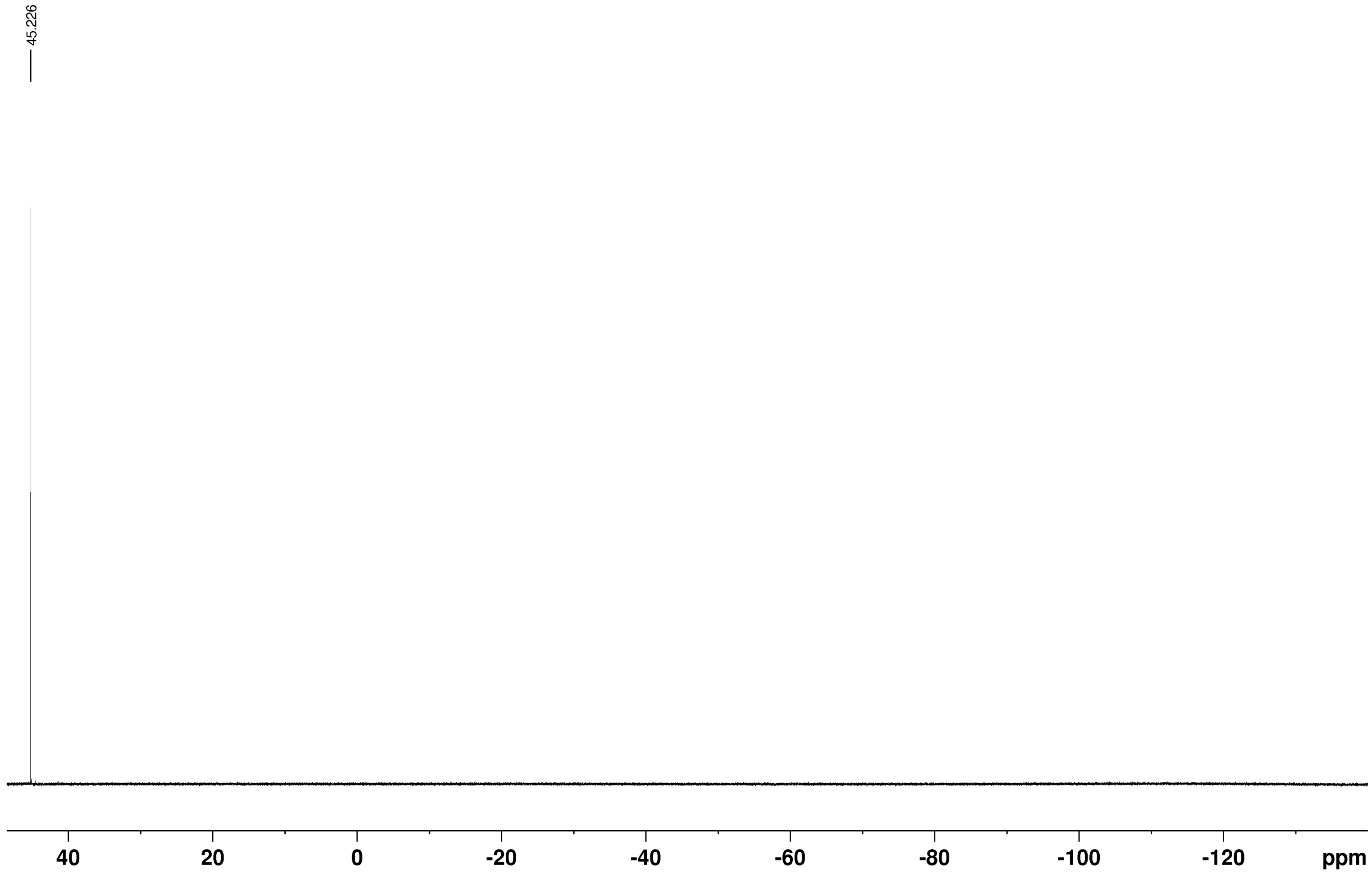

Supplementary Figure 79. ^{1}H NMR (400 MHz, $CDCl_3$) *N*-(cyclohex-2-en-1-yl)aniline (8d)

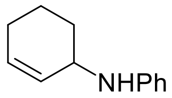

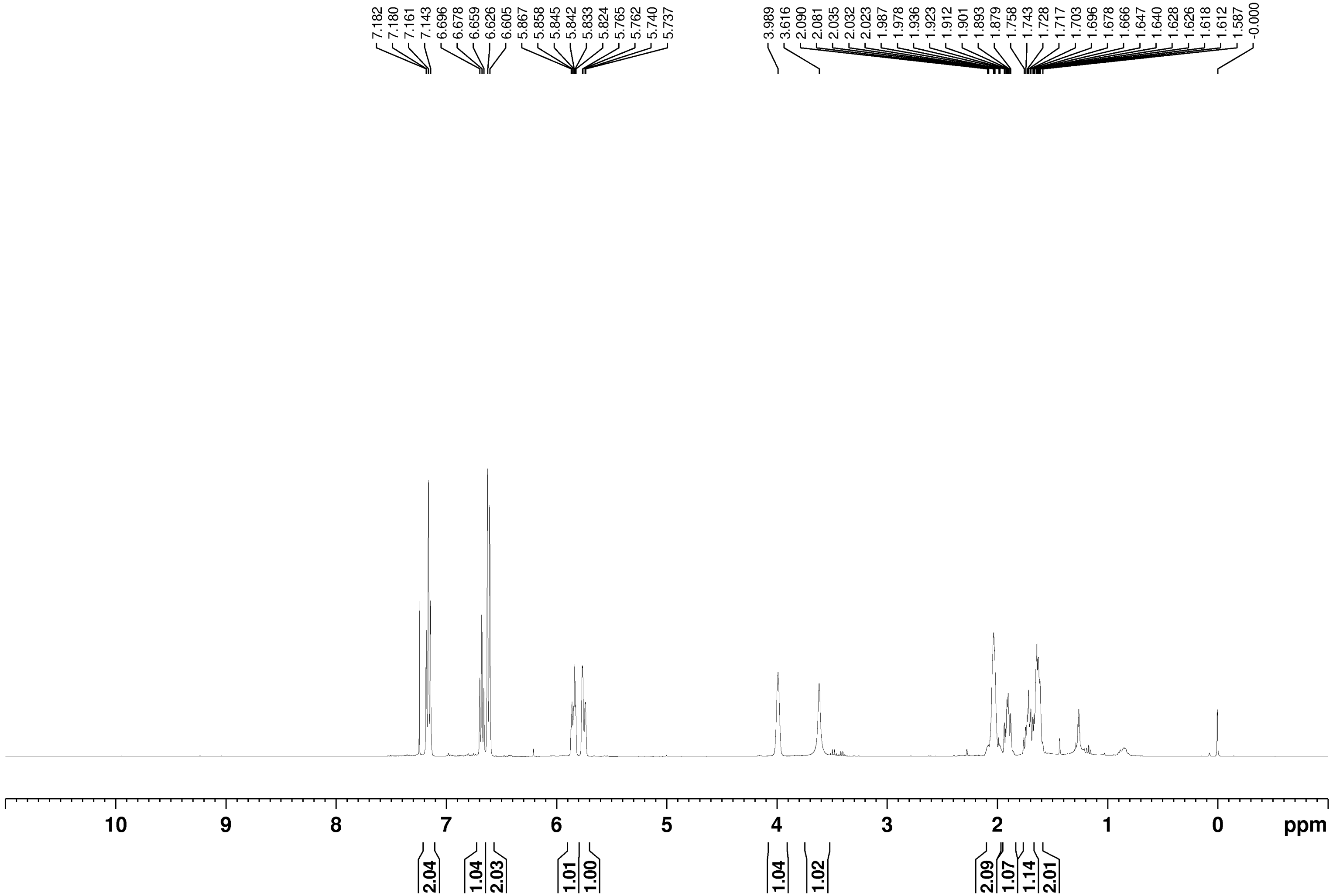

Supplementary Figure 80. ^{13}C NMR ($CDCl_3$, 100 MHz) *N*-(cyclohex-2-en-1-yl)aniline (8d)

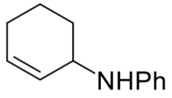

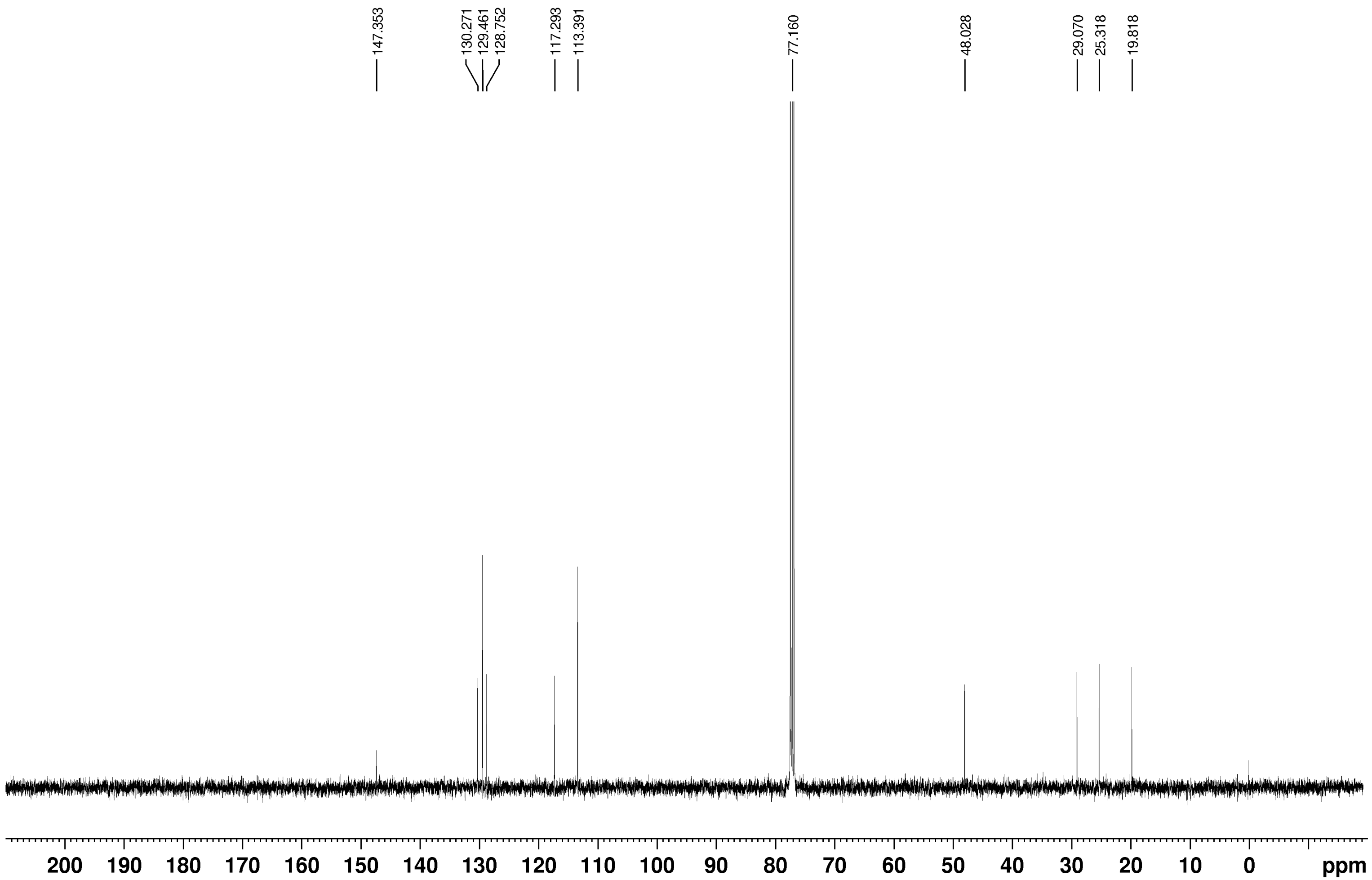

Supplement: Supplementary file 4 — Supplementary Data 2 [file 42004_2024_1208_MOESM4_ESM.pdf]
